# The *FTZ-F1* gene encodes two functionally distinct nuclear receptor isoforms in the ectoparasitic copepod salmon louse (*Lepeophtheirus salmonis*)

**DOI:** 10.1371/journal.pone.0251575

**Published:** 2021-05-20

**Authors:** Joakim Brunet, Christiane Eichner, Rune Male

**Affiliations:** Department of Biological Sciences, SLRC-Sea Lice Research Center, University of Bergen, Bergen, Norway; Laboratoire de Biologie du Développement de Villefranche-sur-Mer, FRANCE

## Abstract

The salmon louse, *Lepeophtheirus salmonis*, is an ectoparasitic crustacean that annually inflicts substantial losses to the aquaculture industry in the northern hemisphere and poses a threat to the wild populations of salmonids. The salmon louse life cycle consists of eight developmental stages each separated by a molt. *Fushi Tarazu Factor-1 (FTZ-F1)* is an ecdysteroid-regulated gene that encodes a member of the NR5A family of nuclear receptors that is shown to play a crucial regulatory role in molting in insects and nematodes. Characterization of an *FTZ-F1* orthologue in the salmon louse gave two isoforms named *αFTZ-F1* and *βFTZ-F1*, which are identical except for the presence of a unique N-terminal domain (A/B domain). A comparison suggest conservation of the *FTZ-F1* gene structure among ecdysozoans, with the exception of nematodes, to produce isoforms with unique N-terminal domains through alternative transcription start and splicing. The two isoforms of the salmon louse *FTZ-F1* were expressed in different amounts in the same tissues and showed a distinct cyclical expression pattern through the molting cycle with *βFTZ-F1* being the highest expressed isoform. While RNA interference knockdown of *βFTZ-F1* in nauplius larvae and in pre-adult males lead to molting arrest, knockdown of *βFTZ-F1* in pre-adult II female lice caused disruption of oocyte maturation at the vitellogenic stage. No apparent phenotype could be observed in *αFTZ-F1* knockdown larvae, or in their development to adults, and no genes were found to be differentially expressed in the nauplii larvae following *αFTZ-F1* knockdown. *βFTZ-F1* knockdown in nauplii larvae caused both down and upregulation of genes associated with proteolysis and chitin binding and affected a large number of genes which are in normal salmon louse development expressed in a cyclical pattern. This is the first description of *FTZ-F1* gene function in copepod crustaceans and provides a foundation to expand the understanding of the molecular mechanisms of molting in the salmon louse and other copepods.

## Introduction

The salmon louse, *Lepeophtheirus salmonis*, is an ectoparasitic copepod that lives of salmonids by feeding on mucus, skin, and blood. The salmon louse life cycle consists of eight developmental stages each separated by a molt: two planktonic nauplius stages followed by an infective copepodid stage, two immobile chalimus stages, two mobile pre-adult stages and finally the mobile reproductive adult stage [1–3]. The parasite is a threat to the welfare of both wild and farmed salmonids [4], and commercially important as it is responsible for significant financial losses in the salmon farming industry [5]. Better knowledge of the ecdysone pathway in salmon lice will help in understanding the initiation and regulation of the complex process of molting. To combat the parasite with new drugs, knowledge of endocrine regulatory mechanisms is indispensable with molt and reproduction as the main target processes. Molting is necessary for growth and development of the parasite and may represent a step where it is specifically vulnerable towards attack.

Ecdysteroid hormones are used to regulate various aspects of arthropod development, including molting and reproduction [6]. The hormone signals are typically mediated by a nuclear receptor dimer, the most prominent example is the ecdysteroid receptor which consists of a dimer of the ecdysone receptor (EcR) and Ultraspiracle (Usp) [7–9]. In the well-studied *Drosophila melanogaster* molting model, the liganded EcR/Usp dimer controls a transcriptional cascade of ecdysteroid regulated genes, consisting mainly of other nuclear receptors that subsequently regulate metamorphosis and timing of molting [10]. The specific temporal and spatial expression of ecdysteroid inducible nuclear receptors such as ecdysone inducible factor 75 (E75), hormone receptor 3 (HR3), hormone receptor 4 (HR4) and Fushi-Tarazu Factor 1 (FTZ-F1) are crucial for the correct transition through a molt cycle [11].

The ecdysone receptor has been identified in crustaceans belonging to the decapod and branchiopod order, reviewed in Nakagawa and Henrich [12]. However, the functional role of the ecdysone receptor and other putative members of the ecdysone regulatory cascade are not as explored in crustaceans compared to insects, with only a few recorded knockdown studies conducted on crustaceans [13–15]. In the salmon louse, separate knockdown of EcR [16, 17] and Usp [18] affected growth and reproduction in larvae and pre-adult females, while dual knockdown of EcR/Usp resulted in molting arrest at the second nauplius stage [17].

FTZ-F1, a member of the NR5A class of the nuclear receptor superfamily, is an ecdysteroid regulated nuclear receptor. In *D*. *melanogaster*, alternative transcription starts and splicing in the *FTZ-F1* gene generates two protein isoforms named αFTZ-F1 and βFTZ-F1. They contain identical DNA and ligand-binding domains, but unique N-terminal A/B domains [19]. In other investigated insect species, only one FTZ-F1 isoform has been reported [20–25]. However, a similar mechanism of alternative transcription, as seen in *Drosophila FTZ-F1*, has been reported in the beetle *Leptinotarsa decemlineata* [26] and the branchiopod crustacean *Daphnia magna* [14]. In *Drosophila*, *αFTZ-F1* transcript is maternally deposited and the protein is required for pair-rule segmentation in early embryogenesis by interacting with the homeobox domain protein FTZ and activating the transcription of *Engrailed* [27]. βFTZ-F1 is required for all stage transitions, and its expression is induced mid-to-late embryogenesis, prior to all larval-to-larval and larval-to-pupa ecdysis, and eclosion from pupa to adult [28]. *βFTZ-F1* mutants display cuticular abnormalities and fail to shed cuticle during molting throughout *Drosophila* development [29, 30].

The importance of the *FTZ-F1* gene in molting has been shown in different ecdysozoans. In holometabolous [11], hemimetabolous insects (*Blattella germanica*) [23], and the nematode *Caenorhabditis elegans* [31, 32], depletion of FTZ-F1 led to abnormal cuticle development and molting arrest. There are only few reports on FTZ-F1 orthologues in crustaceans. In the crab, *Eriocheir sinensis*, FTZ-F1 is involved in the regulation of vitellogenin expression [33], and in *Daphnia magna*, knockdown of *FTZ-F1* transcripts in embryos resulted in hatching failure.

The aim of the present study was (I) to identify *FTZ-F1* orthologues in the salmon louse, (II) to elucidate its molecular structure, and (III) to functionally characterize its isoforms through gene expression and knockdown studies.

## Materials and methods

### Animal culture

A laboratory strain (LsGulen) of salmon louse was propagated on Atlantic salmon (*Salmo salar*) as described in Hamre et al. [34]. The salmon were fed a commercial diet and kept in sea water with a salinity of 34.5 ppt and temperature of 10°C. Adult gravid females were collected with forceps from salmon anesthetized with a mixture of metomidate (5 mg/l) and benzocaine (60 mg/l). Eggstrings were removed with forceps and laid for hatching in flow-through hatching wells as described in Hamre *et al*. [34]. Hatched larvae were kept in the same hatching wells. Fish infection was done as described in Hamre *et al*. [34]. All experiments were performed according to Norwegian animal welfare regulations with the approval of the governmental Norwegian Animal Research Authority (ID7704, no 2010/245410).

### Sampling of sea lice and dissection of tissue

For qPCR measurements of embryonal development, egg string pairs from individual lice were divided, one egg string was put on RNA later, while the other eggstring was incubated further to observe the hatching time point. For time series of nauplii I and nauplii II larvae development, the time point for hatching was registered.

Individual differences between lice in developmental pace have been explored in Eichner *et al*. [35]. Lice are not of exact comparable instar age, even when sampled at same time point after infection. Therefore, preadult lice were photographed at sampling and sorted based on the ratio between the length of the cephalothorax (CT) and the total length (TL) of the animal. Since the growth pattern of the cephalothorax and abdominal segment are different, with more growth of the abdominal segment compared to the cephalothorax within the same stage, this ratio can be used as a proxy for age [35], with the range going from younger (higher ratio) to older (lower ratio).

Tissues were extracted from adult lice. Dissection of brain, testis, spermatophore, oocytes and ovaries were performed by removing the ventral exoskeleton with a scalpel and removing organs with as little surrounding tissue as possible. Subcuticular tissue was obtained by cutting the outer sides of the animals containing just subcuticular tissue and cuticular glands. Intestine was obtained by pulling it out of the animal. All samples with the exclusion of sexual organs contained a mixture of female and male tissue.

### RNA extraction and cDNA synthesis

#### Salmon louse larvae

Larvae (15–25 larvae per sample) were homogenized (30s x 4 at 6.0 m/s) with a FastPrep™ machine in 300 μl TRI Reagent® (Sigma) using 1.4 mm zirconium oxide beads (Bertin). RNA was extracted using Direct-Zol RNA micro kit (Zymo Research) with on-column DNase digestion according to manufacturer’s protocol.

#### Pre-adult and adult salmon lice

Individual pre-adult and adult salmon lice were homogenized in 1 ml TRI Reagent® (Sigma) using a 5 mm steel bead (Qiagen). RNA was extracted according to suppliers’ protocol with the following change: for phase separation 200 μl 24:1 chloroform/isoamylalcohol was added. The RNA was dissolved in 20–40 μl of RNase free water, treated with DNase I of Amplification Grade (Invitrogen) as described by the manufacturer. RNA was either used directly in cDNA synthesis or stored at -80°C until use.

### Reverse transcription

AffinityScript qPCR cDNA synthesis kit (Agilent Technologies) was used according to suppliers’ protocol with 300 ng total RNA from larvae or 200 ng of total RNA from pre-adult salmon lice in a 10 μl reaction. The cDNA was diluted ten-fold in nuclease free water and stored at -20°C until use.

### Molecular cloning and sequencing

Full-length sequence of *FTZ-F1* was obtained with SMARTer™ RACE cDNA Amplification Kit (TaKaRa Bio). Reverse transcription of DNase treated total RNA from larvae or adult female was done using SMARTscribe according to the manufacturer’s protocol. 5’ and 3’ RACE on larval and adult female RACE-ready cDNA was done with universal and gene specific primers (S1 Table) in a first and nested PCR reaction using conditions specified by the manufacturer. PCR products were purified from agarose gels using NucleoSpin® Gel and PCR Clean-up kit (Macherey-Nagel), and cloned in a pCR^®^4-TOPO TA^®^ vector (Invitrogen) in TOP10 *Escherichia coli* cells. Colony PCR was performed using MOD M13-primers with the following reaction conditions; 5 min denaturation at 95°C, 30 cycles of 30 seconds denaturation at 95°C, 30 seconds annealing at 55°C, and elongation at 72°C for 1 minute. Clones were grown o/n in 5 ml LB medium containing 100 μg/ml ampicillin and purified using NucleoSpin^®^ Plasmid Purification kit (Macherey-Nagel). Clones were sequenced using a BigDye^®^ Terminator v3.1 Cycle sequencing kit (Applied Biosystems). Sequences were analyzed and assembled using Gap4 from the Staden Package [36].

### Gene structure comparison

Orthologous sequences were found using the “orthologues” function in Ensembl Metazoa (Metazoa.ensembl.org), and through default pBLAST search against the ecdysozoa superphylum (hexapoda, chelicerata, crustacea, myriapoda, tardigrada, nematoda, priapulida) with FTZ-F1 as a query sequence (https://blast.ncbi.nlm.nih.gov/Blast.cgi). For predicted *αFTZ-F1* and *βFTZ-F1* sequences, Splign [37] was used for comparison to the genomic sequence (taken from NCBI genome or Ensembl Metazoa) and predict gene structure. All sequences found were verified by blasting the sequence against the Sequence Read Archive (SRA) at NCBI at default settings. The accession numbers for *FTZ-F1* for all species investigated are listed in Table 1.

**Table 1:** Sequences used in gene structure comparison.

	Accession no.		Comments
Species	*αFTZ-F1*	*βFTZ-F1*	
**Insects**			
*D*. *melanogaster*	NM_079419	NM_168775	
*A*. *mellifera*	XP_016766345.1 + XM_006557392.2	XM_006557392.2	
*B*. *mori*	AF426830.1	AB649122.1	
*S*. *litura*	HQ260326.1	XM_022976553.1	
*M*. *sexta*	XM_030168105.1	AF288089.1	
*B*. *germanica*	FM163377.1	*-*	*βFTZ-F1*: Start of ORF from genomic sequence upstream of DBD encoding exon in *αFTZ-F1*
*C*. *felis*	XM_026619028.1	*-*	*βFTZ-F1*: Start of ORF from genomic sequence upstream of DBD encoding exon in *αFTZ-F1*
*A*. *pisum*	XP_029344120.1		*βFTZ-F1*: Start of ORF from genomic sequence upstream of DBD encoding exon in *αFTZ-F1*
*F*. *occidentalis*	XM_026421392.1	*-*	*βFTZ-F1*: Start of ORF from genomic sequence upstream of DBD encoding exon in *αFTZ-F1*
*T*. *castaneum*	XM_008193153.2	XM_008193151.2	
**Myriapoda**			
*S*. *maritima*			*αFTZ-F1*: Constructed from two Ensembl genes (SMAR006163 + SMAR006161), *αFTZ-F1* start missing *βFTZ-F1*: Start of ORF from genomic sequence upstream of DBD encoding exon in *αFTZ-F1*
**Chelicerata**			
*T*. *urticae*	XM_015929659.2	*-*	*βFTZ-F1*: Start of ORF from genomic sequence upstream of DBD encoding exon in *αFTZ-F1*
*P*. *tepidarorum*	XM_016053025.2	*-*	*βFTZ-F1*: Start of ORF from genomic sequence upstream of DBD encoding exon in *αFTZ-F1*
*C*. *sculpturatus*	XM_023378248.1	XM_023378247.1	
*V*. *destructor*	XM_022789294.1	*-*	*βFTZ-F1*: Start of ORF from genomic sequence upstream of DBD encoding exon in *αFTZ-F1*
**Crustacea**			
*H*. *azteca*	XM_018152684.1	*-*	*βFTZ-F1*: Start of ORF from genomic sequence upstream of DBD encoding exon in *αFTZ-F1*
*L*. *salmonis*	MT150277	MT150276	
*D*. *magna*	LC105700.1	LC105701.1	Ishak *et al*. [14]
**Tardigrada**			
*H*. *dujardini*	OQV18443.1	*-*	*βFTZ-F1*: Start of ORF from genomic sequence upstream of DBD encoding exon in *αFTZ-F1*
**Nematoda**	***NHR-25***		
*C*. *elegans*	NM_001029379/ WBGene00003623		Gissendanner and Sluder [31]
*S*. *carpocapsae*	TKR80278.1		
*T*. *trichiura*	CDW52832		

### Gene expression measurements

**Fig 1:**
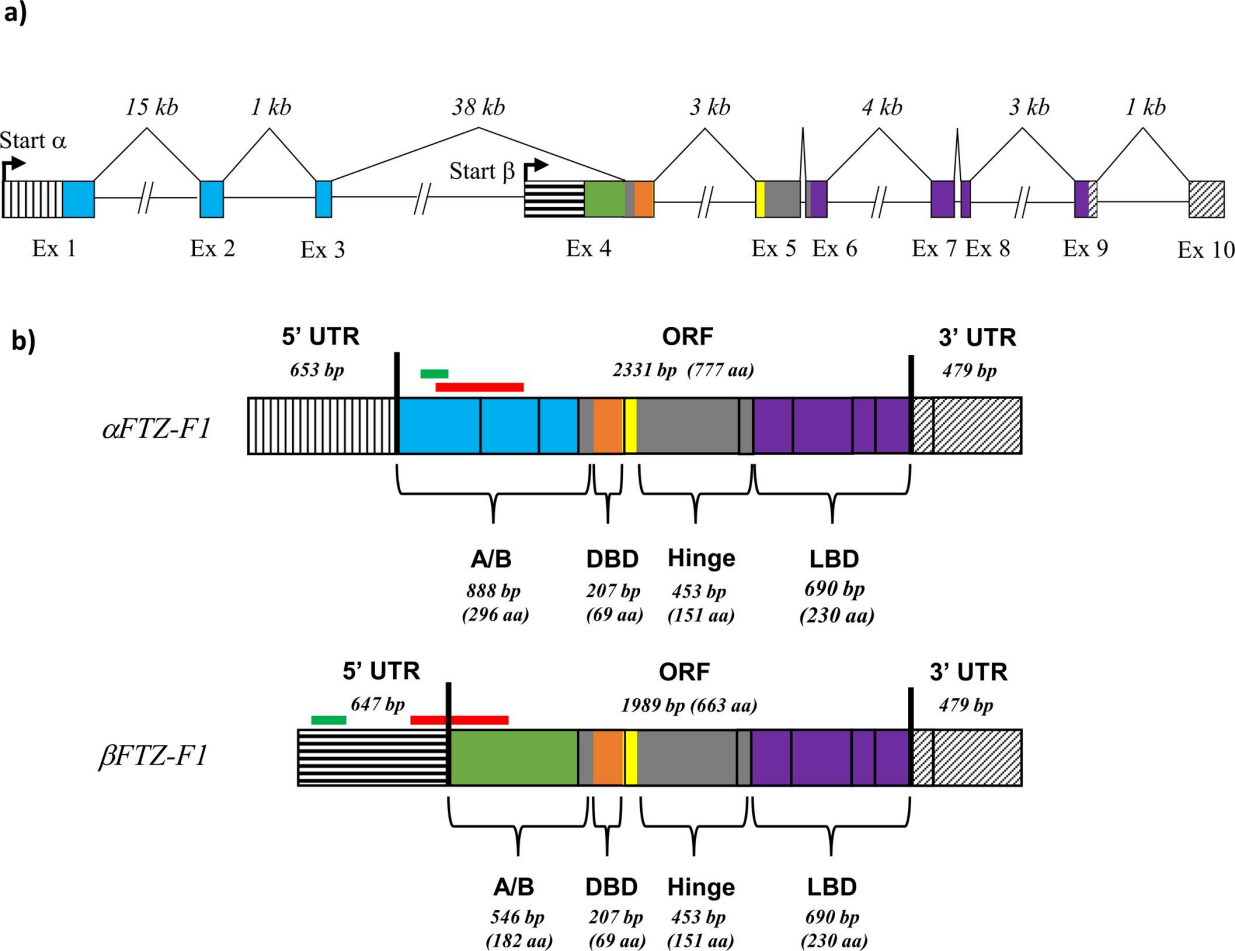
Genomic structure of the *FTZ-F1* gene, and the transcripts produced through alternative transcription. **a)** Schematic representation of the *FTZ-F1* gene in the salmon louse. Two transcript variants of *FTZ-F1* were found. The start of transcription for the *α*-isoform and β-isoform are highlighted with black arrows, with the 5’ UTR for the two variants highlighted with vertical and horizontal lines, respectively. The shared 3’ UTR is highlighted with diagonal lines. Exons and short introns up to 1 kb are drawn in scale. **b)** Schematic representations of the mRNA of *αFTZ-F1* and *βFTZ-F1*. Black lines represent exon borders, while the open reading frame (ORF) of the transcripts are marked between two thick extended lines. The DBD, LBD and N-terminal A/B domain are highlighted, as are the unique N-terminal domains of the *α* and β-isoform. The position of the dsRNA probes for RNA interference is highlighted by a red bar above the transcripts, and the qPCR amplicons are highlighted by green bars.

Expression of *FTZ-F1* was quantified by RT-qPCR carried out on a LightCycler 480^®^ using LightCycler 480^®^ SYBR Green 1 Master kit (Roche Diagnostics) with the following reaction settings: Pre-incubation at 95°C for 10 min, 42 cycles of amplification (95°C, 10s, ramp rate 4.4°C/s; 55°C, 10s, ramp rate 2.2°C/s; 72°C, 20s, 4.4°C/s). Each sample was measured in triplicates in 20 μl reactions according to manufacturer’s protocol using 2 μl cDNA and a primer concentration of 0.5 μM. Melting point analysis was performed after the final amplification cycle. Primer sequences are shown in S1 Table, and the position of the FTZ-F1 primers are highlighted in Fig 1. The primer efficiencies were determined by creating a standard curve using 1:10 serial dilutions of the PCR product as template. The housekeeping gene *Elongation Factor 1α* was used as the internal reference [38].

Measurements of expression following RNA interference experiments were analyzed using the 2^^-ΔΔCt^ method [39]. T-test was used to determine significant difference between experimental groups with a p-value of <0.05 as threshold. Expression profiles in both egg, larvae and pre-adults, expression levels are presented as E^-ΔCt^.

RNA sequencing data from a former study were re-analyzed based on the sequences obtained in this study [40]. The time series data contain samples of chalimus I and II as well as pre-adult I, divided into different instar ages called young (directly after molt), middle, and old (directly before molt) using the ratio between the cephalothorax and total length as a proxy for age.

### RNA interference

#### Synthesis of double stranded RNA probes

PCR fragments for both *FTZ-F1* isoforms, and a control fragment from the trypsin gene in Atlantic cod (XM_030370867.1) [41], were generated using 5x FirePol® 7.5 mM MgCl_2_ Ready to Load PCR Master Mix (Solis Biodyne) with primers flanked by promotor sites for T7 RNA polymerase (S1 Table). The length of the resulting products were 405 and 468 bp for *αFTZ-F1* and *βFTZ-F1*, respectively (Fig 1) and 849 bp for the control fragment. Position of the fragments were in the unique 5’ regions, covering residue number 109–244 for αFTZ-F1 and the last 180 bp for the 5’ UTR and residues 1–93 for βFTZ-F1. The following amplification program was run using plasmid DNA as template in a 50 μl reaction: initial denaturation at 94°C for 5 min, 30 cycles (94°C for 30 s; 60°C for 30 s; 72°C for 1 min) and a final elongation step of 72°C for 7 min. The PCR products were run on an ethidium bromide stained 1% agarose gel, and the PCR product was purified with NucleoSpin® Gel and PCR Clean-up kit (Macherey-Nagel) using DEPC treated water to elute the PCR template. The dsRNA was generated using the MegaScript RNAi kit (Ambion) according to manufacturer’s protocol with 1 μg of DNA template.

#### RNAi of *βFTZ-F1* and *αFTZ-F1* mRNA in salmon louse larvae

RNA interference in larvae was performed as described in Eichner *et al*. [42]. Approximately 300 nauplius I larvae were treated in 150 μl sea water containing 1500 ng dsRNA per fragment, including the control group, which was treated with the control fragment CPY185 [41]. After overnight incubation with dsRNA, larvae were transferred to flow-through incubators and incubated at 8.8 ± 0.1°C for 48 hours before half of the larvae were sampled, placed in RNAlater™ (Ambion) and stored at -20. The remaining larvae were kept in flow through wells to observe further development until the lice from the control group had molted to the copepodid stage. Larvae were followed in the microscope (Olympus SZX 0.5 and 1.6x Olympus objective) and photographed (Canon EOS 600D camera). The larvae were collected and stored in Karnovsky’s fixative for histology. Copepodids from the control and *αFTZ-F1* group were used to infect 3 fish each and kept in single fish tanks. Approximately 50 copepodids were used per fish. Larvae were left to develop to reproductive adults until the females had produced their second eggstring. The lice were removed with forceps from anesthetized salmon. The untreated females were screened for attached spermatophores and eggstrings were placed into hatching wells to investigate hatching success.

#### RNAi of βFTZ-*F1* in pre-adult 1 males

dsRNA was diluted to 600 ng/μl and 1% saturated bromophenol blue was added. Pre-adult 1 males were removed with forceps from anesthetized salmon and placed in a petri dish lined with wet Whatman-paper. dsRNA was injected in the animals as described in Dalvin *et al*. [41], with a borosilicate glass needle pulled using the P-2000 laser-based micropipette puller system (Sutter Instrument). The dsRNA solution was added to the needle using a microloader tip, and then coupled to a HI-7 injection holder (Narishige). After the injection, the lice were kept in sea water for 2–3 hours at 10°C to recover. The injected males were placed upside-down on wet paper together with untreated females in a 2:1 ratio, the paper was subsequently placed on the anaesthetized salmon to attach animals to the host. Untreated females were co-infected with the knockdown male lice, as a lack of females might encourage male lice to abandon the host. 60 males in the pre-adult I stage, were injected with *βFTZ-F1* dsRNA and placed out on 3 fish kept in individual tanks with seawater. 120 males were injected with control fragment and distributed on 6 fish kept in individual tanks. The lice from 1 fish from the *βFTZ-F1* knockdown group and 3 fish from the control group were collected 6 days after injection to assess gene expression and phenotype in the pre-adult II stage. The remaining lice were collected 35 days post infection (2 fish with βFTZ-F1 knockdown lice and 3 fish with control lice) when lice from control group had progressed to the adult stage. The experiment was repeated, but each group contained 60 injected pre-adult I males divided on 5 fish kept in a common tank. 6 days post infection, lice in the pre-adult II stage were collected from 3 fish from each tank to assess downregulation. Male lice were imaged as described in section 2.8.2 and fixed in RNAlater™ (Ambion) for qPCR analysis or in Karnovsky’s fixative for histology. Females were also imaged and investigated for attached spermatophores.

#### RNAi of *αFTZ-F1* and *βFTZ-F1* in pre-adult 2 females

The experiment was performed as described for pre-adult I males in the above section. 30 dsRNA injected pre-adult II females were divided to 3 fish with untreated males in a 2:1 ratio. The fish were kept in individual tanks. The animals were collected from the fish 35 days post injection. The adult female lice were imaged as previously described and placed in RNAlater™ (Ambion) for qPCR analysis, or Karnovsky’s fixative for histology.

### Histology

Salmon lice fixed in Karnovsky’s fixative at 4°C were washed in phosphate buffered saline (PBS) before being dehydrated in a graded ethanol series. The animals were subsequently pre-infiltrated for two hours in 50/50 Technovit/ethanol (Technovit 7100, Heraeus Kulzer Technique), and then infiltrated with Technovit and hardener overnight prior to embedding. Animals were cut in two-micrometer sections using a Leica RM 2165 microtome and stained with toluidine blue (1% in 2% borax). Sections were mounted using DPX Mountant for histology (Sigma). Microscopy and imaging were done using an Axio Scope A1 light microscope connected to an Axiocam 105 color camera (Zeiss International).

### RNA sequencing

Nauplii I larvae originating from the same pair of eggstrings were divided into three groups and treated with 2500 ng dsRNA of either *αFTZ-F1*, *βFTZ-F1* or *CPY185* (control). About 50 larvae from each group were transferred to RNAlater™ (Ambion) 48 hours after molting from nauplius I to nauplius II, and the remaining larvae were left to molt again and develop to copepodids. This experiment was repeated three times, producing three biological parallels per dsRNA treatment. Total RNA extracted from dsRNA treated larvae were sequenced at the Genomics Core Facility, University of Bergen. Sequencing libraries were prepared from 400 ng total RNA using Illumina® TruSeq® mRNA Stranded Sample Preparation kit. Samples were barcoded, pooled together and sequenced in a single lane using the Illumina HiSeq4000 (Illumina, Inc., San Diego, CA, USA), producing 2x75 base pair (bp) paired end reads. Image analysis and base calling were performed using Illumina’s RTA software version 2.4.11, and the data was converted to fastQ format using bcl2fastq version 2.1.7.1.14.

### Data processing of RNA sequencing

The sequences were quality controlled using FastQC v.0.11.9 and summarized using MultiQC v.1.7. The reference genome utilized was a combination of the Ensembl Metazoa reference assembly of the nuclear genome (LSalAtl2s, *Lepeophtheirus salmonis*) and the mitochondrial genome RefSeq sequence NC_007215 [43]. The gene models from Ensembl Metazoa were further enhanced with the inclusion of gene models of *FTZ-F1* isoforms derived from sequences obtained through rapid amplification of cDNA ends (RACE). RNA-seq reads were aligned using RNA STAR with default settings in Galaxy under the Norwegian e-infrastructure for Life sciences (NeLs) platform [44, 45]. Differential expression analysis was done with DESeq2 [46] on counts produced from the FeatureCounts tool. All genes with a p-adjusted value of < 0.05 were included in the list of differentially expressed genes. GO annotation enrichment analysis was performed using the GOEnrichment tool in the public European Galaxy server [44, 47]. In order to get accurate counts for the two *FTZ-F1* isoforms, we performed a Kallisto quantification using the database of all salmon louse cDNA from Ensembl Metazoa edited to contain the correct full-length transcript of the *FTZ-F1* isoforms [48]. A DESeq2 analysis was performed on the Kallisto quantification in the same manner as described above. The hierarchical clustering was made with data from the time series study from Eichner *et al*. [40] using J-Express [49].

## Results

### *FTZ-F1* alternative transcription start sites and splicing are conserved among ecdysozoans

pBLAST search in LiceBase.org against all salmon louse protein entries gave a hit with the salmon louse gene EMLSAG00000008902. The 5’ and 3’ Rapid Amplification of cDNA Ends (RACE) on RNA from nauplius II and adult female produced two cDNAs, later identified as *αFTZ-F1* (accession number: MT150277) and *βFTZ-F1* (accession number: MT150276) extended over 3460 and 3112 nucleotides (nt), respectively. The two *FTZ-F1* transcripts share sequence with the exception of a unique 5’ end of 1431 (*α*) and 1022 (*β*) nt. The two predicted ORFs of 777 and 663 aa, includes unique N-terminal ends of 259 and 145 aa, and share DNA-binding domain (DBD) and ligand-binding domain (LBD). The predicted genome organization of the two *FTZ-F1* transcripts are shown in Fig 1. The two transcripts are generated through an alternative transcription start and splicing. The isoforms were named *αFTZ-F1* and *βFTZ-F1* due to the similarity in gene structure to the *Drosophila melanogaster* orthologues. The 3460-nucleotide long *αFTZ-F1* stretches over exon 1–9, while the transcription of the 3112 base pair long *βFTZ-F1* starts in an alternative start exon that extends into exon 4 of *αFTZ-F1*.

**Fig 2:**
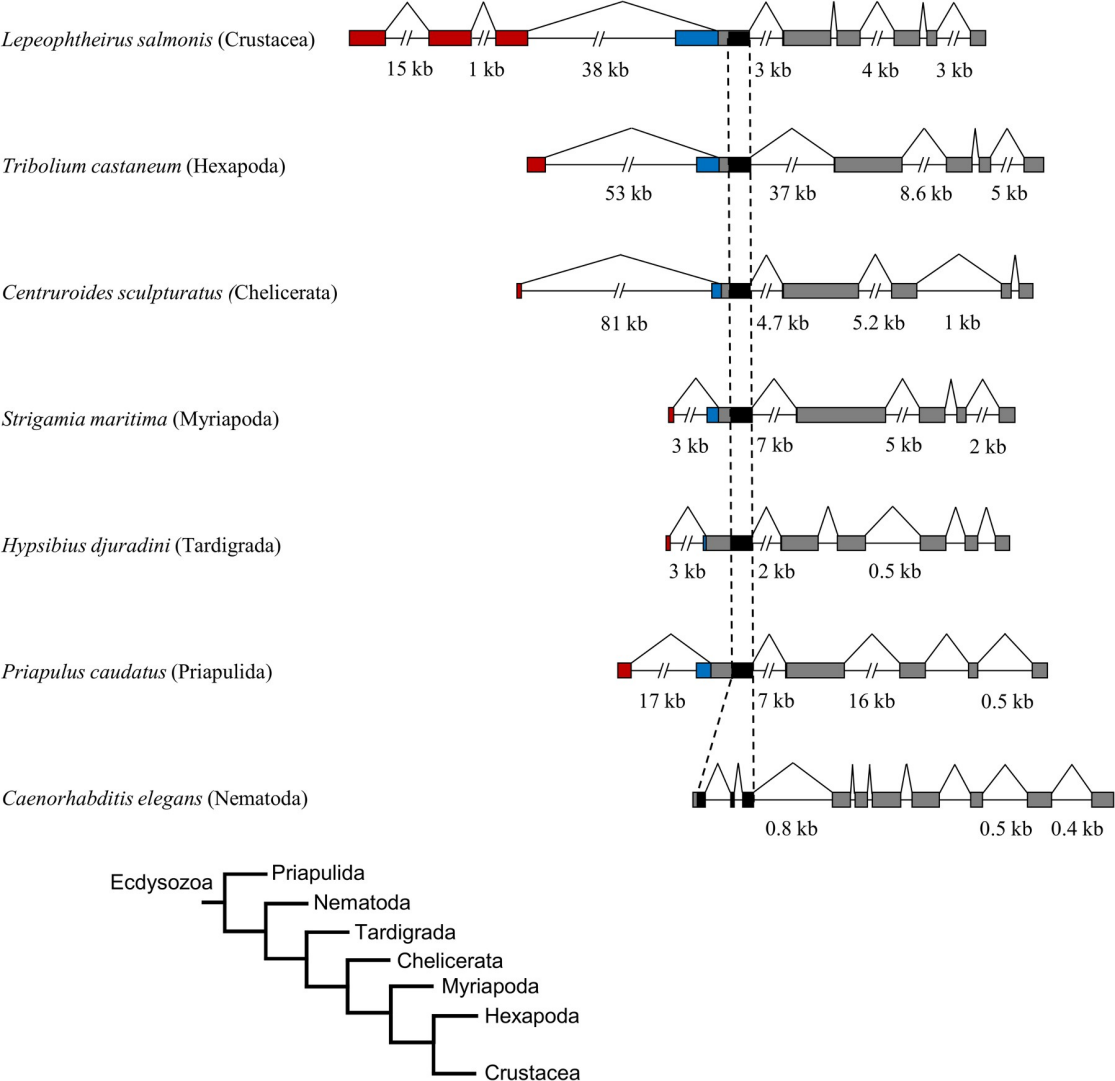
Comparison of *FTZ-F1* gene structure between ecdysozoans. The open reading frame of *FTZ-F1* genes from different ecdysozoans organisms are aligned through the area of the gene coding for the DNA-binding domain (black), with the stippled lines highlighting this alignment. The area coding for the isoform specific parts of the N-terminal domain is highlighted by red (*αFTZ-F1*) and blue (*βFTZ-F1*). Exons are represented by boxes, and introns and splicing patterns are shown with lines. Exons and introns of 1kb or lower are in scale, with sizes of selected introns given in kilobases (kb). The evolutionary relationship between the different ecdysozoans are shown in the schematic tree chart in the bottom [50, 51]. For accession IDs see Table 1. All sequences and structure are available in S1 File.

*FTZ-F1* in *Drosophila melanogaster* and *Lepeophtheirus salmonis* have similar structural organization, as do *Daphnia magna* [14], where *αFTZ-F1* and *βFTZ-F1* are generated through alternative transcription start and splicing (S1 Fig). In all three species, *βFTZ-F1* transcription originates in an intron of *αFTZ-F1* upstream of the DBD encoding exon, generating a transcript with an alternative 5’ end. Available gene sequences of *FTZ-F1* in organisms from the different subphyla of the ecdysozoan superphylum (Table 1) were investigated to explore potential conservation of the FTZ-F1 gene structure. A selection of 8 hexapod, 2 crustacea, 4 chelicerate, 1 myriapod, 1 tardigrade and 1 priapulid species were investigated, and showed a potential upstream ORF in the extension of the DBD coding exon that may include an alternative transcription start to generate a putative *βFTZ-F1*. It appears to be a general feature that the arthropod *FTZ-F1* gene encodes two transcripts that generates isoforms with unique N-terminal parts. The size of the predicted N-terminal within the α and β isoforms varied substantially between species, as well as the length of the unique part of the α and β N-terminal parts within the same species. A comparison of the predicted gene structures based on cDNA sequences from representative species selected from different subphyla is shown in Fig 2. The *FTZ-F1* gene of three nematode species investigated showed a structure that differed from the other ecdysozoan species, with the conserved DBD being split into three or two exons. The two isoforms reported in C. elegans are generated through an alternative splicing that results in a new initiation of translation that produces an isoform lacking a DBD [31, 32]. All transcript structures are depicted in S1 File along with the corresponding sequences, in addition to reads discovered in the NCBI sequence read archive (SRA).

### Expression of *αFTZ-F1* and *βFTZ-F1* mRNA

**Fig 3:**
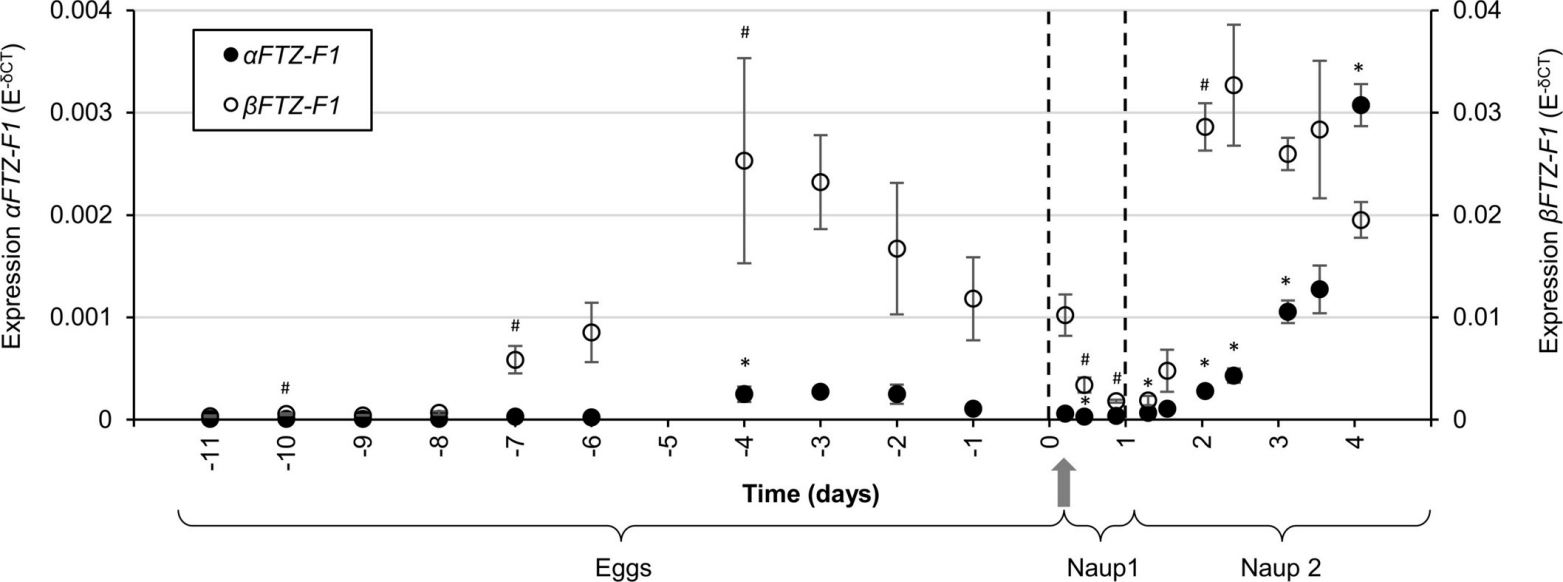
Expression of *αFTZ-F1* and *βFTZ* from early fertilized egg developmental to late nauplius II. The expression of *αFTZ-F1* and *βFTZ-F1* was measured by RT-qPCR against Elongation Factor 1α as internal reference in eggs, nauplii (Naup) 1 and 2. Every point represents the mean of three biological parallels (eggs = 1 eggstring each, larvae = 10–25 individuals each). Error bars indicate standard deviation. The stippled lines highlight stage transitions and the gray arrow indicates time of hatching. * marks groups significantly different (T-test: p value < 0.05) in *βFTZ-F1* expression compared to sample time point before.

RT-qPCR quantification of both *FTZ-F1* isoforms revealed that *αFTZ-F1* and *βFTZ-F1* have a similar expression pattern during early larval development until hatching, and from hatching to second molting, but are present at significantly different levels (Fig 3). In eggs shortly after fertilization, both transcripts are expressed at their lowest levels, with *αFTZ-F1* levels close to the detection limit, roughly 40 times lower compared to *βFTZ-F1*. 7 days before hatching, the expression of both transcripts increase, followed by another marked increase 4 days before hatching. Expression of *βFTZ-F1* decreases steadily through the nauplius I stage and increases again at 11 hours old nauplius II with the highest rise between 11 and 23 hours. *βFTZ-F1* levels are reduced by 40% from the nauplius II peak to the last measurement prior to molt. The *αFTZ-F1* mRNA expression also decreases at the beginning of the nauplius I stage, but appears to start increasing late in nauplius I and continues to rise at a steady rate throughout the nauplius II stage with a 95-fold increase from the lowest expression level in nauplius I to the last measurements in nauplius II prior to molt.

**Fig 4:**
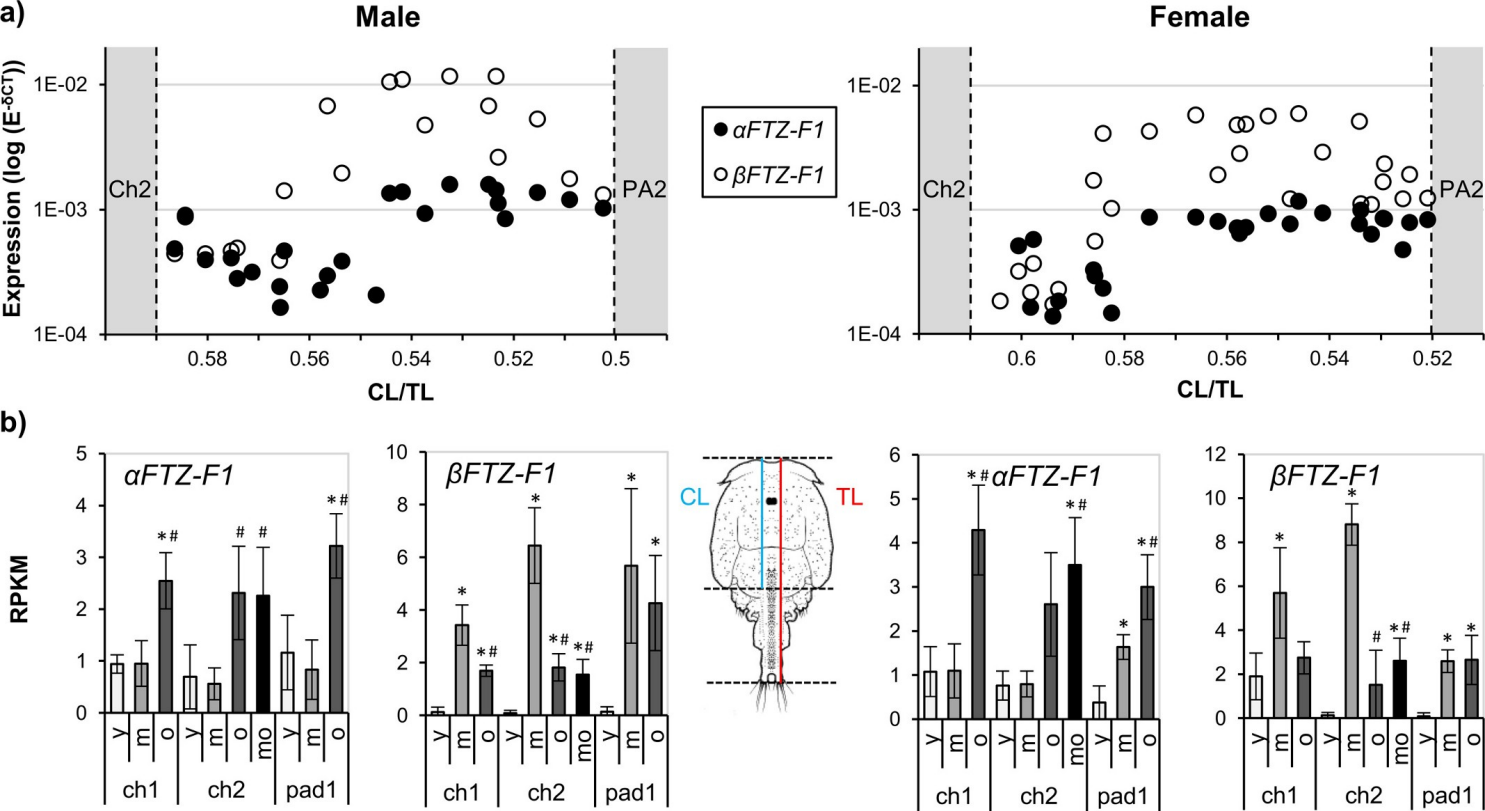
*αFTZ-F1* and *βFTZ-F1* expression during the molt cycle of male and female salmon lice. **a)** Expression of *αFTZ-F1* and *βFTZ-F1* during the molt cycle of pre adult 1 male and female lice. Each point represents the expression of *αFTZ-F1* or *βFTZ-F1* in one individual. The lice have been sorted by their individual instar age based on the ratio between the length of the cephalothorax (CL) and the total length (TL) of the animal (See materials and methods) with the youngest lice having the highest CL/TL. The stippled lines represent boundaries to the previous life stage, chalimus 2 (Ch2), and the next life stage, pre-adult 2 (PA2). **b)** Expression of *FTZ-F1* transcripts during the molt cycle in chalimus (Ch) 1 and 2 and pre-adult 1 (Pad). Expression data are taken from a time series study measured by RNA sequencing [40] for alpha and beta unique transcript parts only. Values are shown as reads per kilo base per million mapped reads (RPKM). For each stage the gene expression in lice of different instar age of the 3 to 4 categories; young (y), middle (m), old (o) and molting (mo) sorted by use of CL and TL ratios is shown. Significantly different sample groups (T-test <0.05) are marked with * for difference to young and with # for difference to middle. A significant difference (T-test <0.05) between male and female expression of the different sample groups could be only seen for Ch1 y. For details of the composition of lice see [40].

The overall expression patterns of *αFTZ-F1* and *βFTZ-F1* during the pre-adult I stage for both sexes are similar to the patterns observed during the nauplius II stage and egg development (Fig 4A). However, unlike during egg development and the nauplius stages, the two isoforms are expressed at similar levels at the start of the pre-adult I stage for both sexes. *βFTZ-F1* expression then increases 13-fold for males and 22-fold for females, while the increase of *αFTZ-F1* is slightly delayed and lower compared to *βFTZ-F1* in both sexes. The expression of *αFTZ-F1* remains at a constant level throughout the pre-adult I stage, while peak *βFTZ-F1* expression is followed by a decline of 80–90% in both sexes at the end of the instar. A similar shift in the expression levels of *αFTZ-F1* and *βFTZ-F1*, with *βFTZ-F1* earlier upregulated than *αFTZ-F1* can also be seen in RNA sequencing data from a time series study done on chalimus and pre-adult lice divided into different intra-instar ages (Fig 4B).

**Fig 5:**
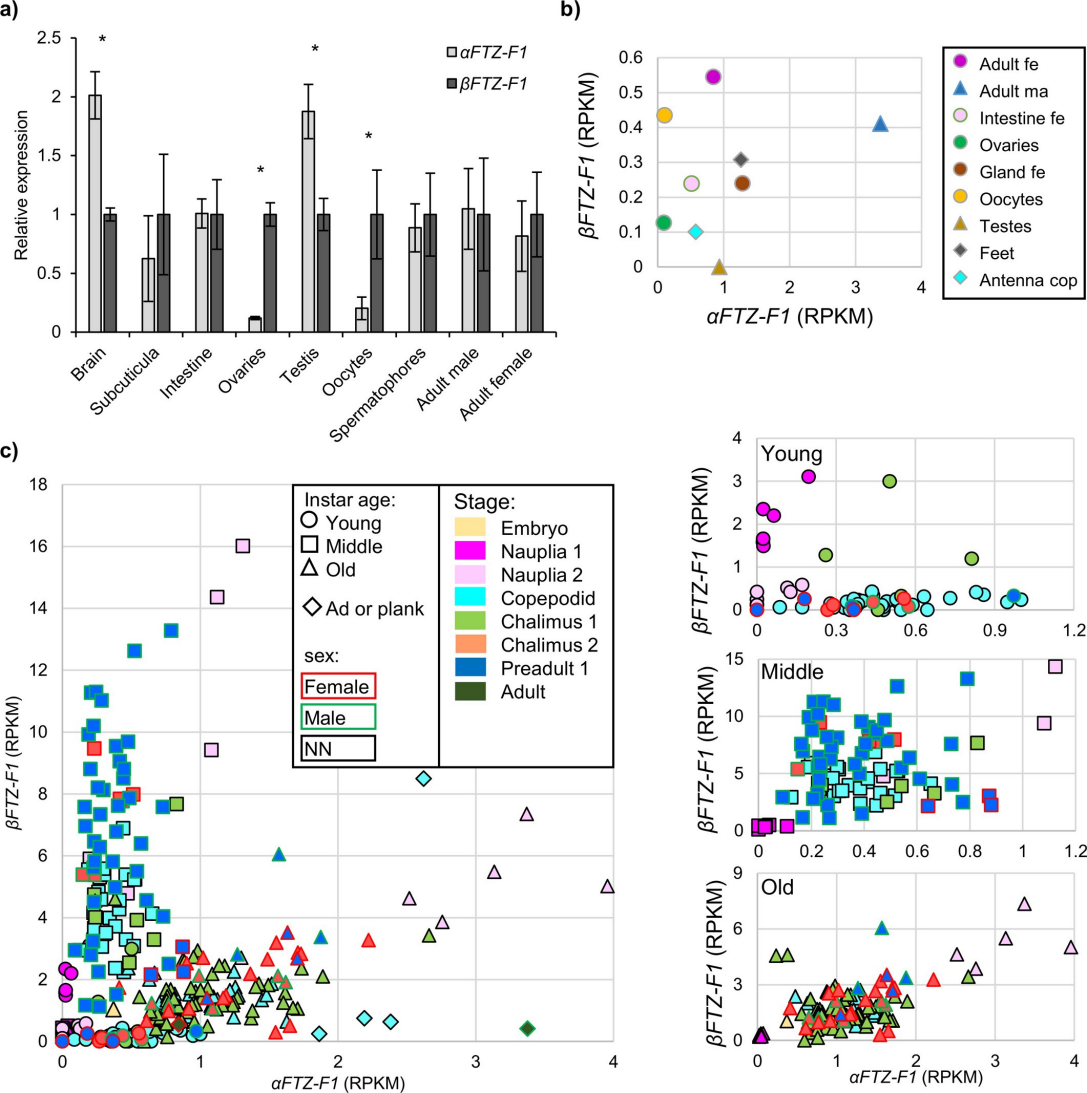
Relative expression of *αFTZ-F1* and *βFTZ-F1* in different adult salmon louse tissues and stages. a) The relative expression of the two *FTZ-F1* isoforms was measured in various adult salmon louse tissues by RT-qPCR. Samples from the intestine, brain and subcuticular contains tissue from both sexes. The expression of *βFTZ-F1* was used as a calibrator, with expression set to 1. Error bars show the standard deviation. N = 3 for each tissue type. * shows significant difference (T-test p value<0.05) between the two isoforms. The expression of the two isoforms measured by RNA sequencing is shown in b) and c). Data are taken from LiceBase.org and from [40] and were analysed for alpha and beta unique transcript parts only. Values are shown as reads per kilo base per million mapped reads (RPKM). b) Expression in different tissues as well as in adult lice; c) different stages and instar ages. For better clarity are data for the different instar ages (young, middle, old) shown separately in the right panel.

Expression of *αFTZ-F1* and *βFTZ-F1* was also measured in different tissues dissected from adult individuals of both sexes (Fig 5). Both isoforms were expressed in all tissues. In the brain and testis, *αFTZ-F1* expression was approximately 2-fold higher compared to *βFTZ-F1*, while *βFTZ-F1* expression was approximately 7-fold higher compared to *αFTZ-F1* in ovaries and 4-fold higher in oocytes. In the other tissues as well as in whole lice the two isoforms were not expressed significantly different from each other. The expression of *αFTZ-F1* and *βFTZ-F1* (individual part only) was also investigated in available RNA sequencing data. Expression of each isoform in different tissues is shown in Fig 5B and of larvae of different instar age in Fig 5C. For ovaries, oocytes and testis a similar trend can be seen with *αFTZ-F1* lowly expressed in ovaries and oocytes and *βFTZ-F1* lowly expressed in testis. In adult male lice, *αFTZ-F1* was much higher expressed than *βFTZ-F1*. The lowest *αFTZ-F1* to *βFTZ-F1* ratio was found in larvae of middle instar age, the highest in young larvae. Nauplii 1 shows a different trend with a low ratio in young larvae comparable with measurements shown in Fig 3. Male and female larvae do not differentiate from each other.

### RNAi mediated knockdown of *βFTZ-F1* in salmon louse larvae and pre-adult male caused molting arrest

**Fig 6:**
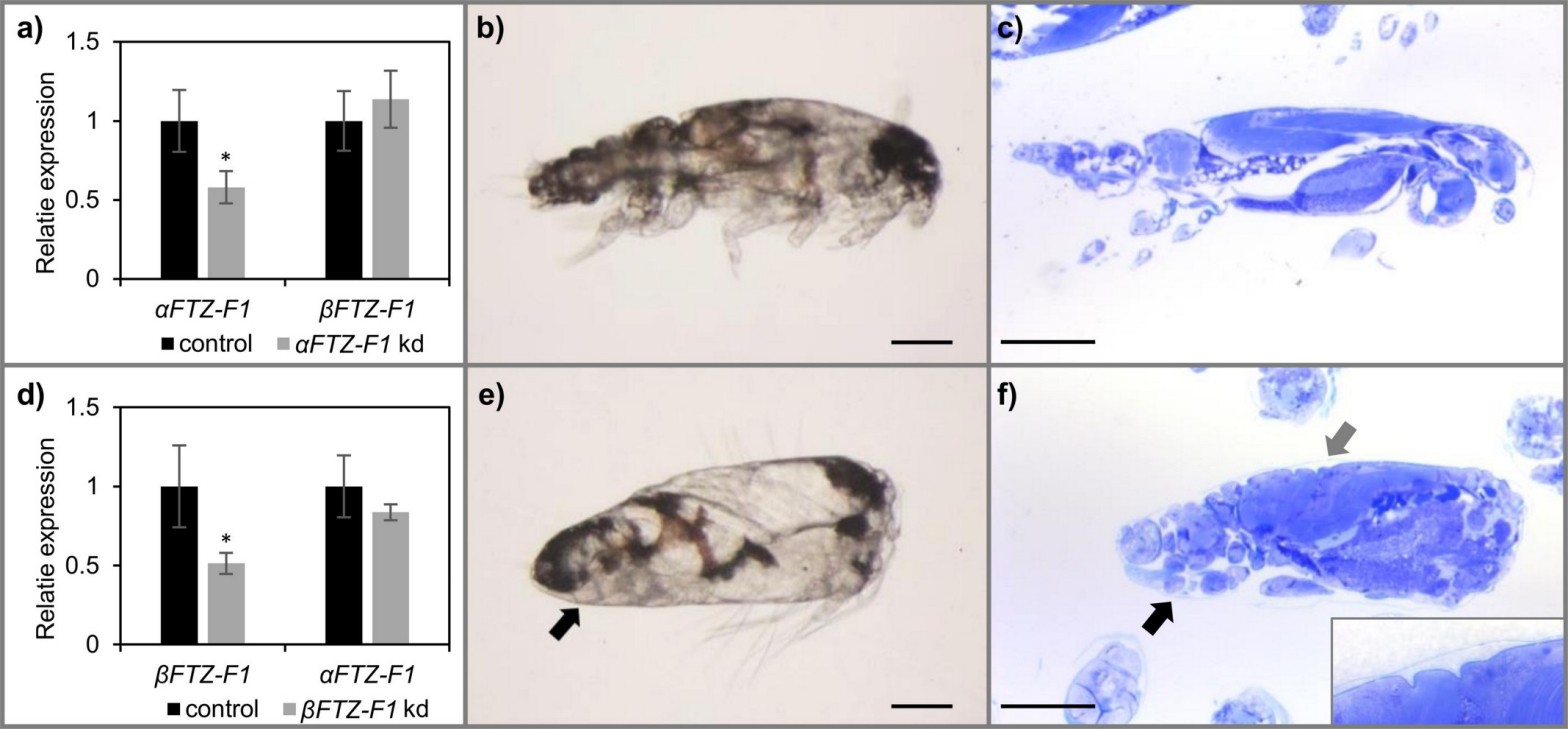
RNAi mediated knockdown of *βFTZ-F1* in nauplius I causes molting arrest at the nauplius II stage. Nauplius I larvae were treated with either dsRNA for *βFTZ-F1*, *αFTZ-F1* or control fragment. **a)** Relative expression of *αFTZ-F1* and *βFTZ-F1* in control (N = 6) and *αFTZ-F1* dsRNA (N = 6) in nauplius II larvae 48 hours after treatment. * indicates significant differences between control and knockdown group (T-test p-value < 0.05) **b)** Larvae treated with dsRNA targeting *αFTZ-F1* molted to the copepodid stage as control **c)** Also, histological sections of *αFTZ-F1* knockdown copepodids showed apparent normal phenotype as the control. **d)** Relative expression of *αFTZ-F1* and *βFTZ-F1* in control (N = 6) and *βFTZ-F1* nauplius II larvae 48 hours after treatment (N = 4). **e)** At the time of molting from nauplius II to copepodid, *βFTZ-F1* knockdown larvae stayed in the bottom of the incubation well and remained trapped in the cuticle. **f)** Sections of molting arrested larvae treated with *βFTZ-F1* dsRNA revealed the development of limbs and segments typical for copepodids (black arrow), and the separation of the new and old cuticle (gray arrow). Scale = 0.1mm.

Knockdown efficacy of the two transcript isoforms in nauplius I larvae 48 hours after treatment was 42% and 49% for *αFTZ-F1* and *βFTZ-F1*, respectively, and no significant difference was detected between the control group and the non-targeted isoform in their respective experimental group (Fig 6A and 6D). When the animals in both the control group and the two *FTZ-F1* knockdown groups approached molting from the nauplius II to copepodid stage, they appeared normal and sank to the bottom of their incubation well. The *αFTZ-F1* knockdown animals and the *CPY185* control group molted successfully to the copepodid stage (Fig 6B), however animals treated with dsRNA targeting *βFTZ-F1* remained at the bottom of the incubator motionless and alive, but unable to molt (Fig 6E). Closer inspection of these animals revealed the presence of limbs and segments like in the copepodid, also seen in the histological sections (Fig 6F). Histology also revealed the presence of two cuticular layers, showing the synthesis of the new cuticle and breakdown/recycling of the old cuticle. After settlement on its host, the *αFTZ-F1* dsRNA treated larvae developed to reproductively functional adults, and their offspring molted successfully to copepodids with no apparent phenotype.

**Fig 7:**
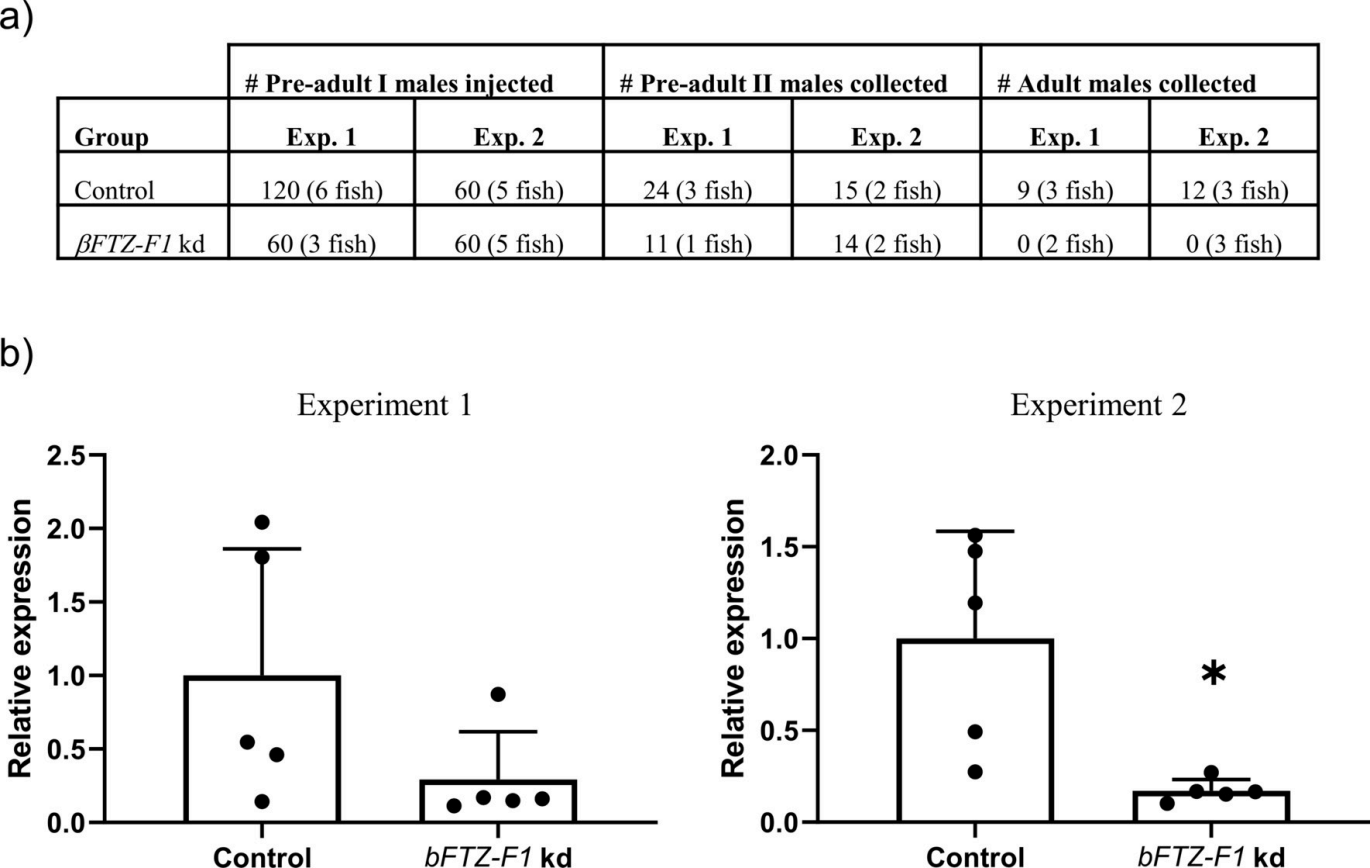
RNAi mediated knockdown of *βFTZ-F1* in pre-adult I salmon louse inhibits development into adults. a) Collective number of lice injected with *βFTZ-F1* dsRNA and collected at the two sampling time points. The number of fish at start and terminated at each time point is listed as well. Pre-adult I male lice injected with *βFTZ-F1* dsRNA developed into pre-adult II lice but failed to molt to adult lice across identical experiments. **b)** The relative expression of *βFTZ-F1* in control (n = 5) and knockdown (kd) (n = 5) group across two parallel RNAi experiments. Each dot represents a pre-adult II male louse. * indicates statistically significant knockdown of *βFTZ-F1* in knockdown group compared to control (T-test: P-value ≤ 0.05).

Knockdown of *βFTZ-F1* in pre-adult I males also caused developmental defects. Initially, the pre-adult I knockdown males developed normally to the pre-adult II stage without any apparent phenotype, but 35 days after injection when terminating the experiment, no adult males were found on the fish remaining in the *βFTZ-F1* knockdown group (Fig 7). It appeared that no *βFTZ-F1* knockdown males had been able to molt from the pre-adult II stage to the adult stage, and subsequently were fallen from the host either to be flushed out of the tank or eaten by fish. Untreated females that were co-infected with *βFTZ-F1* knockdown males did not have any spermatophores attached to the genital segment at the end of the experiment, supporting the idea that the *βFTZ-F1* knockdown males failed to develop past the pre-adult II stage. The knockdown efficacy was only significant in the second experiment, due to the high variability in expression of *βFTZ-F1* between lice in the control groups (Fig 7).

### RNAi mediated knockdown of *βFTZ-F1* in pre-adult II females disrupts oogenesis during the vitellogenic stage in adults

**Fig 8:**
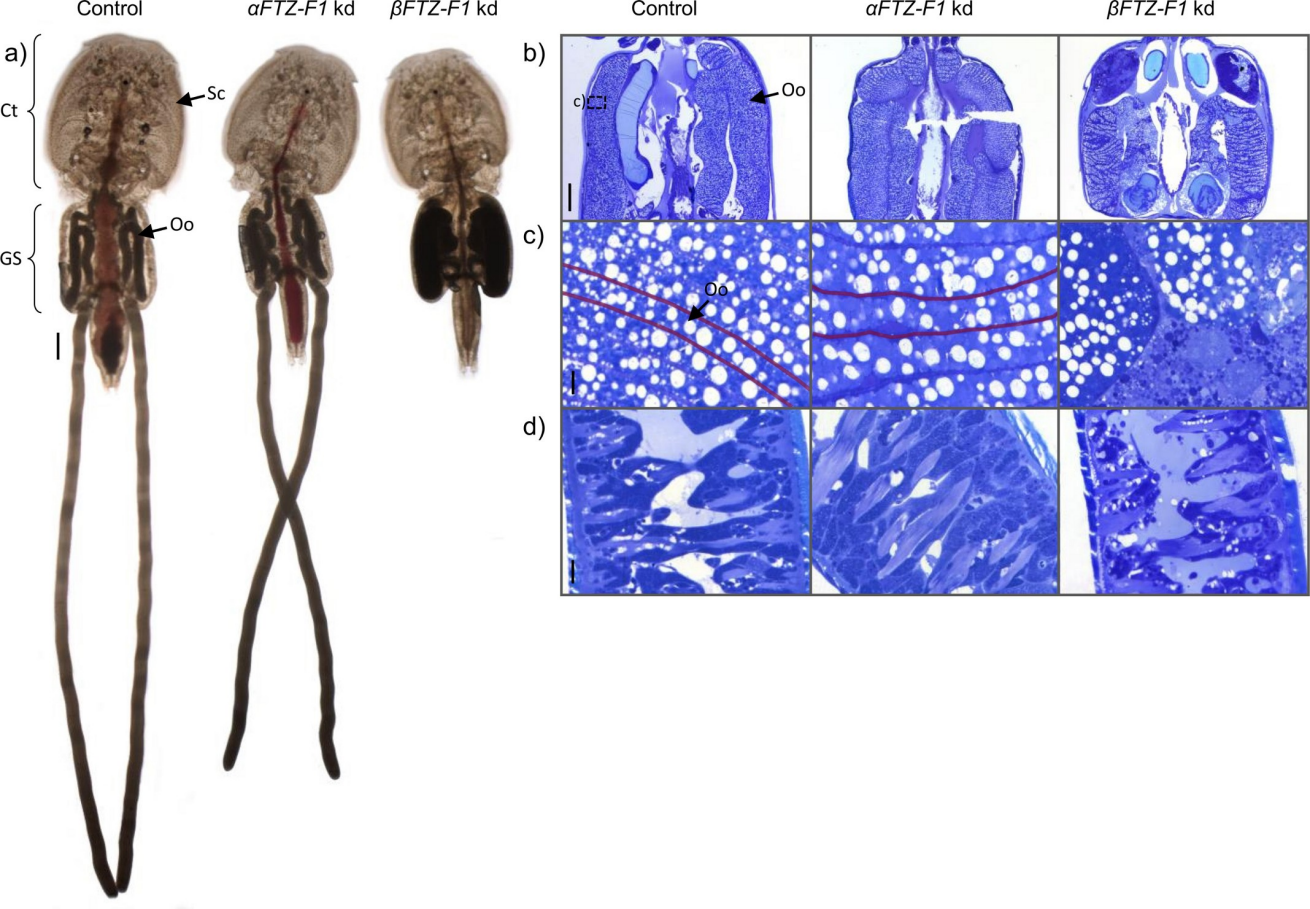
RNAi mediated knockdown effect of *αFTZ-F1* and *βFTZ-F1* in adult female lice. a) photographs of typical adult female lice at sampling for the three groups: control lice, αFTZ-F1 and knock-down of βFTZ-F1. Ct = Cephalothorax, GS = Genital segment, Sc = subcuticular tissue, Oo = oocytes, scale bar = 1mm. Toluidine blue dyed sections of lice from the same groups are shown in b-c. b) genital segment, scale bar = 500μm; c) magnification of the oocytes marked by a square in b). The outline of one oocyte in the genital segment is drawn in red in c), scale bar = 10μm; d) subcuticular tissue of the cephalothorax, scale bar = 10μm.

We attempted to selectively knock down both *αFTZ-F1* and *βFTZ-F1* in pre-adult II females to investigate the isoforms potential role during sexual maturation and reproduction (Fig 8). After undergoing sexual maturity, both the control and *αFTZ-F1* knockdown were able to produce viable eggstrings and offspring, and there was no visible phenotype in *αFTZ-F1* knockdown lice. Measurement of eggstring length did not show a significant difference between *αFTZ-F1* knockdown and control group. Females treated with *βFTZ-F1* dsRNA were able to molt to adults but had little intestinal blood and produced no eggstrings (Fig 8A). Oocytes were disorganized in the vitellogenic stage in the genital segment or appeared to be broken apart (Fig 8B and 8C). Ovaries and oocytes in the oviduct did not vary from control lice. The subcuticular tissue of the cephalothorax in the *βFTZ-F1* knockdown lice contained less and smaller cells than that of the control lice (Fig 8D) while the subcuticular tissue of the *αFTZ-F1* knockdown lice seemed to be unchanged. The experiment was performed three times producing the same result. No significant knockdown was detected for either isoform in their respective group.

### Transcriptome sequencing

The effect of RNAi knockdown of *αFTZ-F1* and *βFTZ-F1* on the salmon louse transcriptome was investigated by Illumina® mRNA sequencing of dsRNA treated nauplius II larvae. The sequencing of each sample produced on average 39.5 million reads. Out of all the reads, 92.1% mapped uniquely to the salmon louse reference genome, and 4.1% mapped to multiple loci. On average between all samples, 76.2% of all reads overlapped with exon regions in the annotated salmon louse gene models. Sequencing data are available at the NCBI SRA read archive (BioProject: PRJNA687532).

#### *βFTZ-F1* knockdown exhibits strong effects on overall gene expression compared to *αFTZ-F1* knockdown

**Fig 9:**
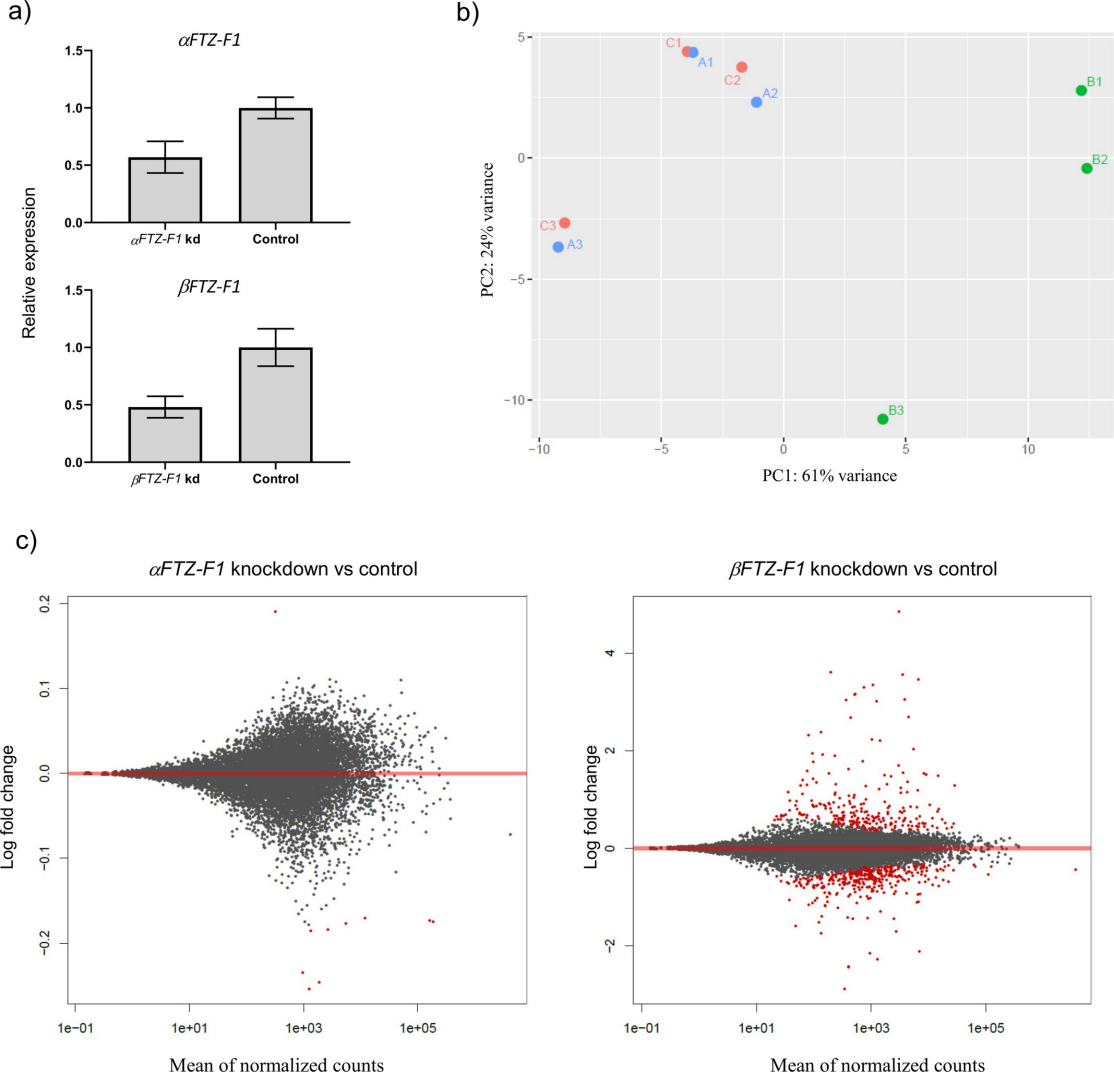
Principal Component Analysis (PCA) and MA plot of the RNAi sample transcriptomes. a) RT-qPCR measurements of *αFTZ-*F1 and *βFTZ-F1* in the mRNA sequencing samples, N = 3. b) PC1 and PC2 represent the two top dimensions of the differentially expressed genes among the three dsRNA treated groups and their three parallels. A = *αFTZ-F1* dsRNA treated larvae, B = *βFTZ-F1* dsRNA treated larvae, C = control dsRNA treated larvae. **c)** MA-plots for DESeq2 comparing the expression of genes in *αFTZ-F1* and *βFTZ-F1* knockdown samples compared to control. The average binary logarithm of the expression across all samples is shown on the x-axis and the binary logarithm of fold change is shown on the y-axis (note different y-axis). Red dots indicate differentially expressed genes (P-adjusted ≤ 0.05), while grey dots represent genes that are not differentially expressed between the two groups.

Knockdown of *αFTZ-F1* and *βFTZ-F1* was verified by RT-qPCR prior to mRNA sequencing and amounted to 42% and 51%, respectively (Fig 9A). The principle component analysis (PCA) revealed that larvae treated with *αFTZ-F1* dsRNA displayed little difference in overall gene expression compared to the control samples, while larvae treated with *βFTZ-F1* dsRNA displayed a large difference compared to the same control samples (Fig 9B). Differential gene expression (DGE) analysis identified a total of 217 upregulated and 278 downregulated genes following *βFTZ-F1* knockdown (Fig 9C). DGE analysis on *αFTZ-F1* knockdown samples identified only 10 differentially expressed genes compared to control, with differences (log2 fold change) ranging from only 0.17 to 0.25 (Fig 9C). The results from both DE analysis as well as the normalized gene counts are listed in S2 Table. In an attempt to verify the findings from the DESeq2 analysis of the *αFTZ-F1* knockdown, we performed RT-qPCR on 4 of these 10 differentially expressed genes on the same samples as well as on samples from a previous *αFTZ-F1* RNAi experiment (S3 Fig). RT-qPCR measurements on the same samples confirmed a similar fold change in all four genes, but we did not observe any difference in expression of these genes between control and knockdown group in the samples from the previous RNAi experiment shown in Fig 6.

#### *βFTZ-F1* downregulation disrupts the cyclical expression of genes, and affects genes associated with proteolysis and chitin-binding

There was a high number of differentially expressed genes following *βFTZ-F1* knockdown with no annotations regarding potential functions or homology to other genes. Of the 217 upregulated genes, 109 (50%) were completely without annotations, as the genes had no BLAST hits, or matched hypothetical/uncharacterized genes in other species, and had no known Pfam domains. 75 of the 278 downregulated genes (27%) were also without any annotations. Gene ontology enrichment analysis revealed only 2 enriched molecular function (MF) and biological process (BP) terms among the upregulated genes (S4 Fig). The MF terms enriched were inward rectifier potassium channel activity and chitin binding. Among the downregulated genes, the terms with the highest significant enrichment across both MF and BP categories were associated with proteolysis, organo-nitrogen compound metabolic process and chitin binding (S4b Fig). No terms in the cellular compartment (CP) category was enriched among the differentially expressed genes. Enrichment analysis data is shown in S3 Table.

**Fig 10:**
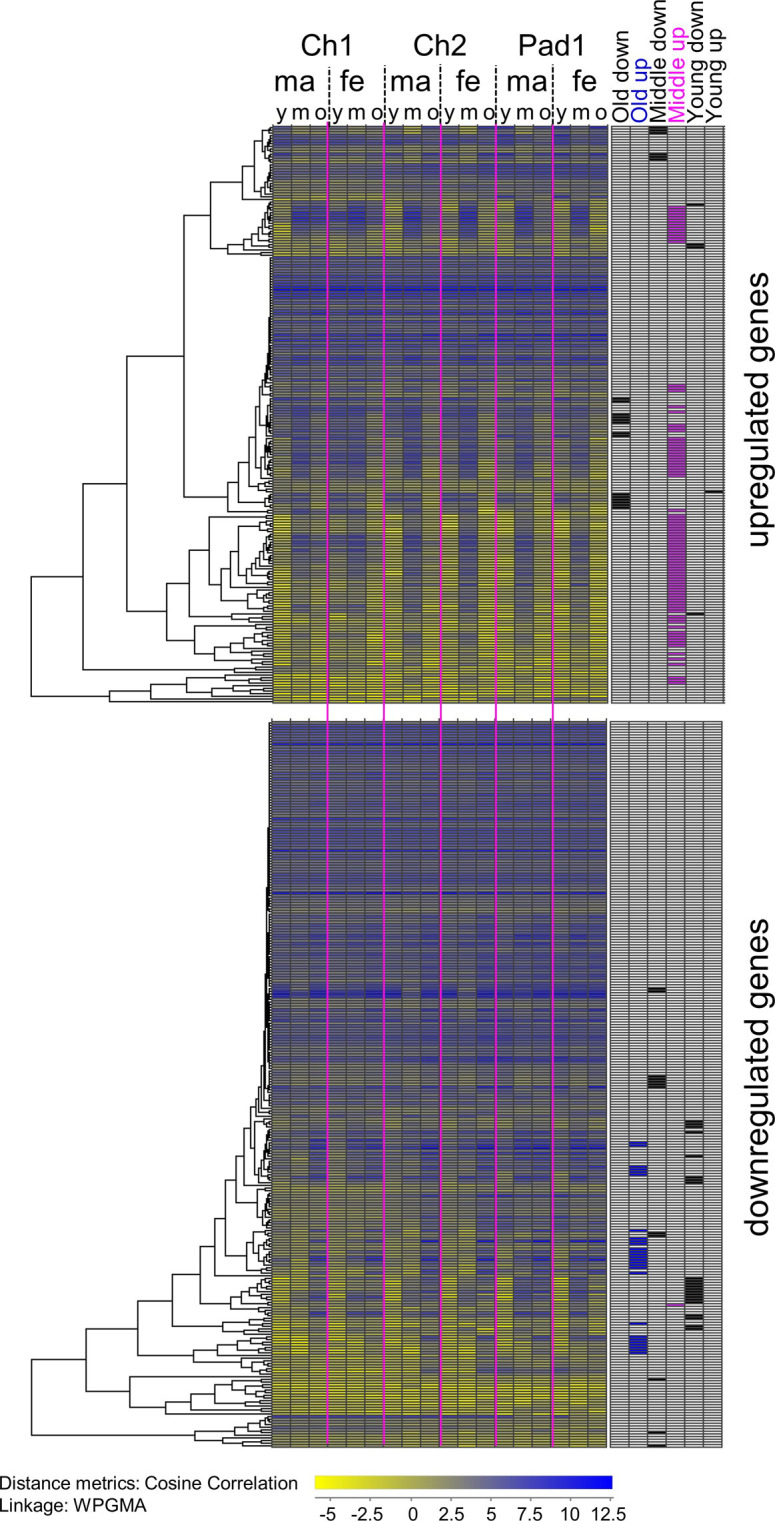
Gene expression up and downregulated after *βFTZ-F1* knockdown during the development of chalimus larvae into the preadult 1 stage. Data are taken from Eichner *et al*. [40] and show the expression profiles of the differentially expressed genes in chalimus stage 1 and 2, and preadult 1 lice (mean values) divided into different instar age with respect to molting ((young (y): directly after molting, middle (m): in the middle of the stage and old (o): directly before the molt to next stage)). The cyclically expressed genes (old, middle and young up or down respectively) described in Eichner *et al*. [40] are marked.

In a time-series study by Eichner *et al*. [40], the expression of 707 cyclically expressed genes were divided into 6 categories based on their expression pattern within an instar in chalimus and pre-adult I lice. Of the 495 differentially expressed genes following β*FTZ-F1* knockdown, 178 belonged to cyclically expressed genes, 116 upregulated and 62 under the downregulated category. The DE genes and whether or not it belongs to one of the genes described as cyclically expressed during development through chalimus and pre-adult I stages are shown in Fig 10 [40]. Strikingly many of the genes (43%) upregulated following β*FTZ-F1* knockdown belong to the genes that were found to be cyclically upregulated in the middle of the stage (pink marked). A small cluster, comprising unknown genes, however, shows the opposite pattern with lowest expression in larvae in the middle of the stage. Under the genes downregulated following *βFTZ-F1* knockdown, less cyclically expressed genes were generally found and most of these were upregulated in larvae prior to next molt (old up). The genes strongest upregulated after knock-down of *βFTZ-F1* (at least 5 times on average, 29 genes) are mainly unknown genes (10 genes without any annotation or Pfam domain as well as 15 uncharacterized or hypothetical proteins). All but 2 of these are upregulated in the middle of the stage compared to expression during normal development. Under the strongest downregulated genes (at least 5 times, 8 genes) are proteases, a cuticle protein-encoding gene and two unknown genes without annotations.

## Discussion

The revealed structure of the *FTZ-F1* gene of *L*. *salmonis* with different N-terminal isoforms generated through alternative promoter usage and splicing are not uncommon among nuclear receptors, and have been described in human ROR-alpha [52], human HNF4-alpha [53], fruit fly E75 [54], fruit fly EcR [55], and murine thyroid hormone receptor beta [56]. FTZ-F1 has been characterized in several ecdysozoan species, and N-terminal isoforms have been reported in *D*. *melanogaster* [19], *L*. *Decemlineata* [26], and *D*. *magna* [14] (Fig 2). FTZ-F1 has also been described in the arthropods *Metapenaeus ensis* [57], *Eriocheir sinensis* [33], *Aedes aegypti* [25], *Tribolium castaneum* [21], *Blattella germanica* [23], *Manduca sexta* [24], *Bombyx mori* [58], *Spodoptera litura* [22], and *Apis mellifera* [20], but in these studies, only one isoform of FTZ-F1 was reported. That isoforms were not detected in the former studies could have technical reasons (e.g. that rapid amplification of cDNA ends (RACE) and northern blots were performed on only one developmental stage, tissue or cell type, or that the antibodies and RT-PCR primers were specific to the N-terminal domain of only one potential isoform). To discover if the conserved organization of the *FTZ-F1* gene found in *D*. *melanogaster*, *L*. *salmonis* and *D*. *magna* could also be found in the different subphyla of the ecdysozoa we investigated available sequences of ecdysozoan organisms with only a predicted α*FTZ-F1* sequence. By re-analyzing the sequences in light of this study we could show that this structure is a conserved feature among most ecdysozoans, but not among nematodes. The nematode sequences currently available display a different splicing pattern of the DBD domain encoding exon compared to the other ecdysozoans to produce a DBD lacking isoform [31]. No isoforms with unique N-terminal domains has been described in nematodes thus far (Fig 3). The number of species investigated outside of the hexapods were limited, mainly due to few available sequenced genomes from these subphyla. However, summary of available data favor that the animals of the group called panarthropoda have two FTZ-F1 isoforms.

In *D*. *melanogaster*, the two *FTZ-F1* isoforms are expressed at different stages during development, with *αFTZ-F1* being expressed only during early embryogenesis, prior to any *βFTZ-F1* expression [19]. In the salmon louse however, expression of *βFTZ-F1* and *αFTZ-F1* is not spatially or stage separated as in the fruit fly, but both isoforms can be found in all stages and are found in all investigated tissues. Expression height varies within the stage and also the ratio between the isoforms. The ratios vary also between different tissues. (Figs 3–5). Interestingly, in the study by Yussa *et al*. [59], it was revealed that pair-rule segmentation in *Drosophila αFTZ-F1* knockouts could be rescued with βFTZ-F1 protein expression. The variable N-terminal domain did not affect the function of FTZ-F1, which was further demonstrated by the fact that the mouse orthologue SF-1, which does not have an N-terminal domain, was also able to rescue the fruit fly *α*FTZ-F1 knockouts. A study by Ohno *et al*. [60] also demonstrated that the two fruit fly isoforms compete for the same response elements when co-transfected into mammalian cells. The ability of the two *Drosophila FTZ-F1* isoforms to interact with the same transcriptional partners and bind the same DNA binding sites when regulating gene expression, raises interesting questions in regards to isoform function and specificity since the two isoforms are expressed in the same tissues at the same time in the salmon louse (Fig 5). A similar observation has also been reported in *L*. *decemlineata* and *D*. *magna* [14, 26]. The mRNA knockdown efficacy in larvae for *αFTZ-F1* was similar to the efficacy for *βFTZ-F1* (Figs 6 and 9A), no apparent phenotype was detected in the *αFTZ-F1* knockdown group. The exact function of *αFTZ-F1* thus remains unknown, as the knockdown might not have been sufficient to reduce the number of αFTZ-F1 proteins below the threshold for function. However, the expression of both isoforms concurrently in the same tissues raises the possibility that the different N-terminal domains of the two salmon louse *FTZ-F1* receptors confer a specificity in function, which could explain the difference in phenotype we observed when the two transcript variants were subjected to selective knockdown through RNA interference (Fig 6). There are no studies on the function of the N-terminal A/B domain in the class V (NR5A) family of nuclear receptors. A major reason for this is likely the lack of an A/B domain in the mammalian NR5A orthologues SF-1 and LRH-1. Studies on N-terminal function in other nuclear receptor families provide some ideas as to how the N-terminal isoforms could provide specificity of function. N-terminal isoforms have been shown to have different transcriptional output when regulating the same target genes [61], and a different response to ligand activation [62]. However, since the two FTZ-F1 isoforms in the salmon louse are expressed in the same tissues, it is possible that the isoforms have different affinities to DNA response elements and regulate different gene targets [52, 63]. The different N-terminals could also have different affinities to transcriptional partners and cofactors [64]. These affinities shown to be affected by intra domain interactions between the A/B domain and the LBD [65, 66], which in turn can confer specificity in regulation of downstream gene targets. Knockdown of *βFTZ-F1* caused downregulation of three G-protein-coupled inwardly-rectifying potassium channel (S4 Fig), proteins commonly found in the nervous system [67]. Interestingly, *αFTZ-F1* is the predominant isoform in brain tissue (Fig 5). This viewed in context with the potential competitive nature of the two receptor isoforms mentioned previously, raises the possibility that the ratio between the two receptor isoforms could be what is necessary for proper regulation of gene targets, and that a shift in dosage between the two receptors could affect the normal function of one or both receptors. The molecular mechanics of N-terminal function in FTZ-F1 are areas of interest for future in-vitro studies.

mRNA sequencing on larvae 48 hours after treatment with dsRNA, but prior to development of a lethal phenotype, revealed that 495 genes were differentially expressed as a result of *βFTZ-F1* downregulation (Fig 9). In particular, a significant number of genes associated with proteolysis and chitin binding were affected by *βFTZ-F1* knockdown (S4 Fig). This together with the presence of two cuticular layers and the late onset of phenotype at the moment of stage transition (Fig 6), suggests that the molting arrest is due to complications with complete breakdown and detachment of the old cuticle, leaving the nauplii trapped in their own cuticle and unable to proceed with ecdysis. Many of the genes upregulated after β*FTZ-F1* knockdown belonged to a group which during normal development are higher expressed in the middle of the stage, while the ones downregulated after knock-down are usually upregulated directly before the molt (Fig 10). This also confirms that these genes are important for the molting process itself. Involvement in the molt process was also evident when pre-adult I males were subjected to *βFTZ-F1* knockdown. The lice were able to develop to the pre-adult II stage but were unable to molt to the adult stage (Fig 7). No molt arrest occurred at the pre-adult I to pre-adult II molt, which was expected as injection of *βFTZ-F1* dsRNA likely happened at a time point where most of the lice had already developed past the point of peak *βFTZ-F1* expression in the pre-adult I stage. However, the effect of *βFTZ-F1* knockdown on the molt cycle became clear when the lice had to undergo a full molt cycle following the pre-adult I to pre-adult II molt, as no adult males were found 35 days post infection. Our findings provide the first description in crustaceans of FTZ-F1 gene function in molting, and together with known observations of FTZ-F1 function in insects and nematodes, this supports the already established idea of FTZ-F1 as a conserved regulator of molting across the ecdysozoan superphylum. There is high variability in expression of *βFTZ-F1* between individuals in the control group (Fig 7), which is likely due to differences in the instar age (See Fig 4), caused by individual differences in the development rate [35, 68]. Therefore, it was difficult to document the knockdown efficacy in pre-adult II lice.

Injection of dsRNA selectively targeting *βFTZ-F1* in pre-adult II lice as expected did not inhibit molting to the next developmental stage, similar to the observations made when injecting pre-adult I lice. However, the adult females failed to produce viable oocytes with treatment resulting in disorganized and ruptured oocytes in the genital segment during the vitellogenic stage (Fig 8). Also, the cells of the subcuticular tissue seemed to be less and smaller compared to control lice. The subcuticular tissue was shown to be the production area for vitellogenins [69] and the yolk-associated protein, LsYAP [41]. A similar phenotype was observed in a study by Sandlund *et al*. [16] when knocking down EcR transcripts in the same developmental stage, indicating that the putative ecdysone regulatory cascade, like in insects, play a crucial role in the reproductive development in crustaceans. FTZ-F1 appears to be a conserved regulator of oocyte maturation and reproduction among ecdysozoans as in addition to our findings it also plays a vital role in vitellogenesis in crabs [33], mosquitos [25] and honey bees [70], and is shown to be crucial for somatic gonad development in C. elegans [31, 32]. In the aforementioned species, with the exception of nematodes, only one *FTZ-F1* transcript was studied. In the current study, we demonstrated that the *βFTZ-F1* isoform is crucial for oocyte maturation, while injection of dsRNA targeting the *αFTZ-F1* isoform had no effect on oocyte maturation or embryonal development. This observation fits well with the tissue expression analysis of the isoforms, with *βFTZ-F1* being the most predominant isoform in both ovaries and oocytes (Fig 5). No downregulation compared to control lice was detected for either isoform 35 days post injection. This was also seen in the study by Sandlund *et al*. [16], where *EcR* mRNA knockdown was only detectable 2- and 4-days post injection. However, *FTZ-F1* isoform expression fluctuates within a molt cycle (see Fig 4), which makes it difficult to accurately determine downregulation in the pre-adult stages. Downregulation of *αFTZ-F1* was detectable at the larval stage when soaking the lice with dsRNA during the first molt, showing that the fragment is effective. The injection of both dsRNA in pre-adult lice was done with the same dosage and the experiment repeated three times producing the same outcome. Based on this, it appears that only βFTZ-F1 plays an important role during oocyte maturation and female sexual development in the salmon louse. However, if downregulation of *βFTZ-F1* would have an impact on male development, could not be answered by these experiments, since the defect in molting interfered with development to adult male lice.

Knockdown of *αFTZ-F1* did not produce many changes on the overall gene expression (Fig 9B). Analyzing differential expression between the control and the *αFTZ-F1* knockdown larvae revealed only 9 genes, all with a minor fold change (Fig 9C). To verify the results for 4 of the highest differentially expressed genes, RT-qPCR were run for both the sequenced samples and samples from another RNAi experiment. The minor difference in expression could only be verified in the samples subjected to mRNA-sequencing, and not in a repeated RNAi experiment (S2 Fig), and is therefore probably caused by individual biological differences of single animals. We measured similar knockdown efficacy of both *FTZ-F1* isoforms with RT-qPCR (Fig 9A), however, mRNA sequencing did not show the same amount of downregulation. Only reads representing the different N-termini could be counted, resulting in low numbers of reads, and uncertain quantification [71]. A different quantification method (Kallisto) improved the counts for both isoforms compared to FeatureCounts (S3 Fig). However, only *βFTZ-F1* showed a significant difference this way. RT-qPCR is a more accurate method compared to mRNA sequencing when quantifying isoform expression. For *αFTZ-F1*, RNAi in larvae resulted in no visible phenotype and no effect on the transcriptome. There is an uncertainty over how persistent the RNAi effect is during development to adults following soaking at the larval stage. For two other salmon louse genes knocked down, the knockdown effect wore off as the animals grew [17, 72], likely influenced by factors pertaining to mRNA stability, protein turnover and dilution of the dsRNA probe. *αFTZ-F1* knockdown lice placed on hosts developed normally to reproductive adults, suggesting that the downregulation in larvae was insufficient to reduce the amount of *αFTZ-F1* proteins below the threshold for function in the larval stage and in the subsequent stages, or that it has no vital functions during development from larvae to adults. The higher ratio of *αFTZ-F1* expression in the male gonads could suggest a potential role in male reproduction. The investigation of the role of *αFTZ-F1* in male reproduction would have been strengthened by treatment at the pre-adult II stage. RNAseq analysis was only performed on knockdown larvae, so any changes in the transcriptome passed the nauplius II stage was not investigated. In the fruit fly, *αFTZ-F1* is required for pair-rule segmentation during early embryogenesis. A significant rise of transcript levels was observed in late embryo development of *L*. *salmonis* at 4 days up until hatching (Fig 3). Since there is no method to knock down genes in the eggstrings themselves, we are not able to investigate the effect of *αFTZ-F1* knockdown on the embryonal development.

## Conclusion

Here we propose the hypothesis of a structural conservation of the *FTZ-F1* gene in ecdysozoans to produce two isoforms with different N-terminal domains, a feature not found in nematodes. Our findings raise questions around the mechanism of specificity in function provided by N-terminal domains in class V nuclear receptors, and other nuclear receptors in general. We demonstrated that *βFTZ-F1* is a major regulator of molting and oocyte maturation in the salmon louse. This is the first description of *FTZ-F1* gene function in copepod crustaceans. Our findings provide a foundation to expand the understanding of the molecular mechanisms of molting in the salmon louse and other copepods.

## Supporting information

S1 Fig The generation of two isoforms of FTZ-F1 through alternative transcription is conserved in the fruit fly, salmon louse and water flea. A comparison of the FTZ-F1 gene structure of the fruit fly (*Drosophila melanogaster*), the salmon louse (*Lepeophtheirus salmonis*) and the water flea (*Daphnia magna*). The sizes of the primary transcripts and some introns are given in kilobases (kb). Exons are represented as boxes, and introns and splicing patterns as lines. The starts of transcription for α*FTZ-F1* (α) and β*FTZ-F1* (β) are marked with a curved arrow. The 5’ UTR of the α*FTZ-F1* and β*FTZ-F1* transcripts are highlighted yellow and green, respectively. The area of the gene coding for the N-terminal is highlighted red for α*FTZ-F1*, and blue for β*FTZ-F*. The area coding for the shared DBD domain for both transcript variants is colored black. The 3’ UTR is colored light gray. Exons and short introns are in scale.
(DOCX)

S2 Fig RT-qPCR verification of four DESeq2 differentially expressed genes following *Ls*α*FTZ-F1* knockdown. **a)** RT-qPCr measurements of the genes EMLSAG00000008331, EMLSAG00000011833, EMLSAG00000010679, and EMLSAG00000007107 in the samples analysed with mRNA sequencing from both *Ls*α*FTZ-F1* knockdown (kd) and control. **b)** RT-qPCR measurements of the same genes in samples from a previous RNAi experiments, n = 3.
(DOCX)

S3 Fig Counts of *LsFTZ-F1* transcripts from mRNA sequencing with FeatureCounts and Kallisto quantification. **a)** Normalized counts from FeatureCounts of *FTZ-F1* regions in genome annotation file; whole transcript (*αFTZ-F1 +* common region), *LsαFTZ-F1* specific region, *LsβFTZ-F1* specific region. **b)** Counts of *LsαFTZ-F1* and *LsβFTZ-F1* full-length transcripts from Kallisto quantification. Star indicates significant difference compared to control (P-adjusted value ≤ 0.05).
(DOCX)

S4 Fig Gene ontology enrichment analysis of *βFTZ-F1* knockdown animals. GO terms enriched under the genes downregulated (a) or upregulated (b) after *βFTZ-F1* knockdown. Shown are all terms with a q-value <0.05 for the categories biological process (BP) and molecular function (MF). No terms for cellular compartment (CC) met these conditions. Bars show the frequency of the given GO term under the selected genes (study frequency) and in the whole genome (population frequency). The enrichment compared to occurrence in the whole genome is shown as a number on top of each bar. Terms are sorted according to its q-values for each category.
(DOCX)

S1 File Overview of structures, sequences and accession numbers of FTZ-F1 sequences in ecdysozoan species investigated. (DOCX)

S1 Table RT-qPCR primers used in this study and their application. (DOCX)

S2 Table Differential expression analysis (DeSeq2) of salmon louse genes. (XLSX)

S3 Table Data from GOEnrichment analysis. (XLSX)
