# Supplementary material for: The FTZ-F1 gene encodes two functionally distinct nuclear receptor isoforms in the ectoparasitic copepod salmon louse (Lepeophtheirus salmonis)
Source: PLoS One. 2021 May 20;16(5):e0251575. doi: 10.1371/journal.pone.0251575 (PMC8136749; doi:10.1371/journal.pone.0251575)
Supplement: S1 Fig — A comparison of the FTZ-F1 gene structure of the fruit fly (Drosophila melanogaster), the salmon louse (Lepeophtheirus salmonis) and the water flea (Daphnia magna). The sizes of the primary transcripts and some introns are given in kilobases (kb). Exons are represented as boxes, and introns and splicing patterns as lines. The starts of transcription for αFTZ-F1 (α) and βFTZ-F1 (β) are marked with a curved arrow. The 5’ UTR of the αFTZ-F1 and βFTZ-F1 transcripts are highlighted yellow and green, respectively. The area of the gene coding for the N-terminal is highlighted red for αFTZ-F1, and blue for βFTZ-F. The area coding for the shared DBD domain for both transcript variants is colored black. The 3’ UTR is colored light gray. Exons and short introns are in scale. (DOCX) [file pone.0251575.s001.docx]

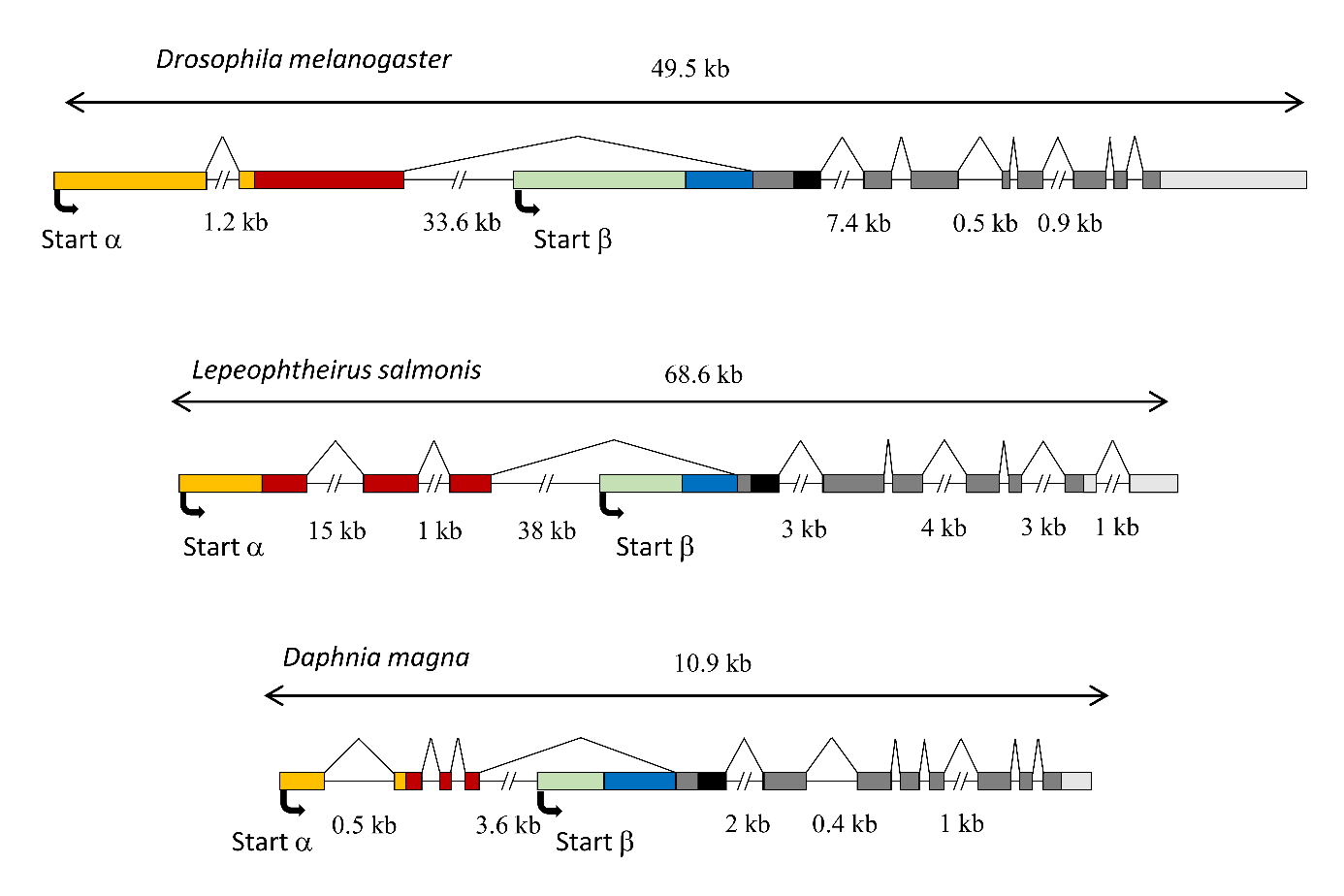
**S1 Fig.** **The generation of two isoforms of FTZ-F1 through alternative transcription is conserved in the fruit fly, salmon louse and water flea.** A comparison of the FTZ-F1 gene structure of the fruit fly (*Drosophila melanogaster*), the salmon louse (*Lepeophtheirus salmonis*) and the water flea (*Daphnia magna*). The sizes of the primary transcripts and some introns are given in kilobases (kb). Exons are represented as boxes, and introns and splicing patterns as lines. The starts of transcription for *αFTZ-F1* (α) and *βFTZ-F1* (β) are marked with a curved arrow. The 5’ UTR of the *αFTZ-F1* and *βFTZ-F1* transcripts are highlighted yellow and green, respectively. The area of the gene coding for the N-terminal is highlighted red for *αFTZ-F1*, and blue for *βFTZ-F*. The area coding for the shared DBD domain for both transcript variants is colored black. The 3’ UTR is colored light gray. Exons and short introns are in scale.
