# Supplementary material for: The FTZ-F1 gene encodes two functionally distinct nuclear receptor isoforms in the ectoparasitic copepod salmon louse (Lepeophtheirus salmonis)
Source: PLoS One. 2021 May 20;16(5):e0251575. doi: 10.1371/journal.pone.0251575 (PMC8136749; doi:10.1371/journal.pone.0251575)
Supplement: S2 Fig — a) RT-qPCr measurements of the genes EMLSAG00000008331, EMLSAG00000011833, EMLSAG00000010679, and EMLSAG00000007107 in the samples analysed with mRNA sequencing from both LsαFTZ-F1 knockdown (kd) and control. b) RT-qPCR measurements of the same genes in samples from a previous RNAi experiments, n = 3. (DOCX) [file pone.0251575.s002.docx]

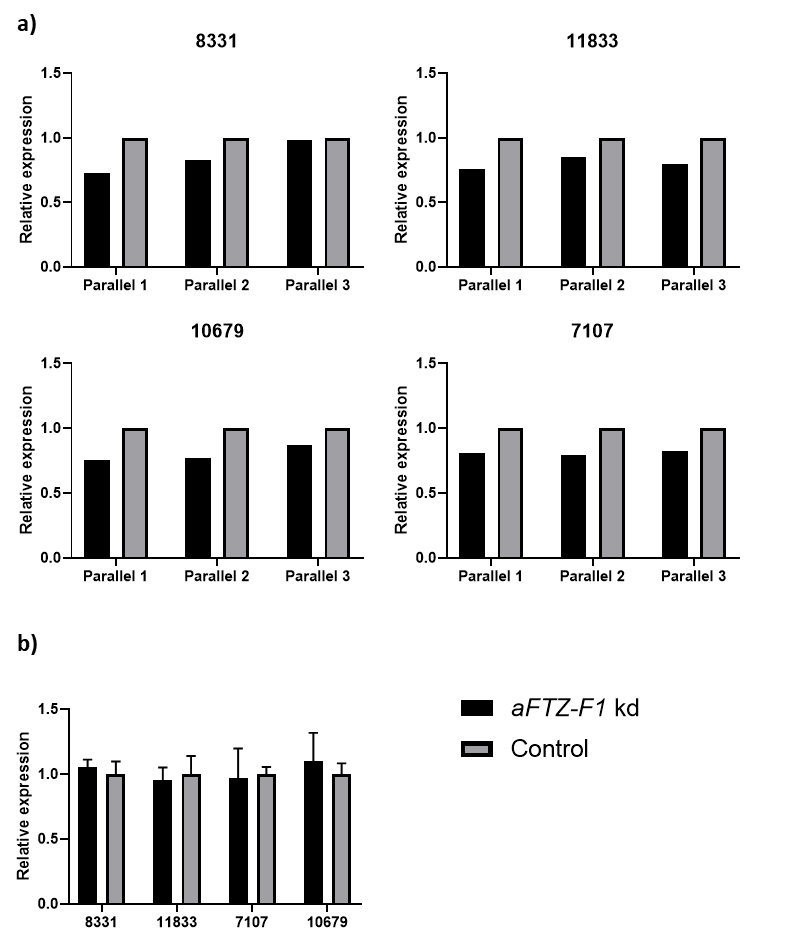


**S2 Fig. RT-qPCR verification of four DESeq2 differentially expressed genes following *LsαFTZ-F1* knockdown**. **a)** RT-qPCr measurements of the genes EMLSAG00000008331, EMLSAG00000011833, EMLSAG00000010679, and EMLSAG00000007107 in the samples analysed with mRNA sequencing from both *LsαFTZ-F1* knockdown (kd) and control. **b)** RT-qPCR measurements of the same genes in samples from a previous RNAi experiments, n=3.
