# Supplementary material for: The FTZ-F1 gene encodes two functionally distinct nuclear receptor isoforms in the ectoparasitic copepod salmon louse (Lepeophtheirus salmonis)
Source: PLoS One. 2021 May 20;16(5):e0251575. doi: 10.1371/journal.pone.0251575 (PMC8136749; doi:10.1371/journal.pone.0251575)
Supplement: S3 Fig — a) Normalized counts from FeatureCounts of FTZ-F1 regions in genome annotation file; whole transcript (αFTZ-F1 + common region), LsαFTZ-F1 specific region, LsβFTZ-F1 specific region. b) Counts of LsαFTZ-F1 and LsβFTZ-F1 full-length transcripts from Kallisto quantification. Star indicates significant difference compared to control (P-adjusted value ≤ 0.05). (DOCX) [file pone.0251575.s003.docx]

**
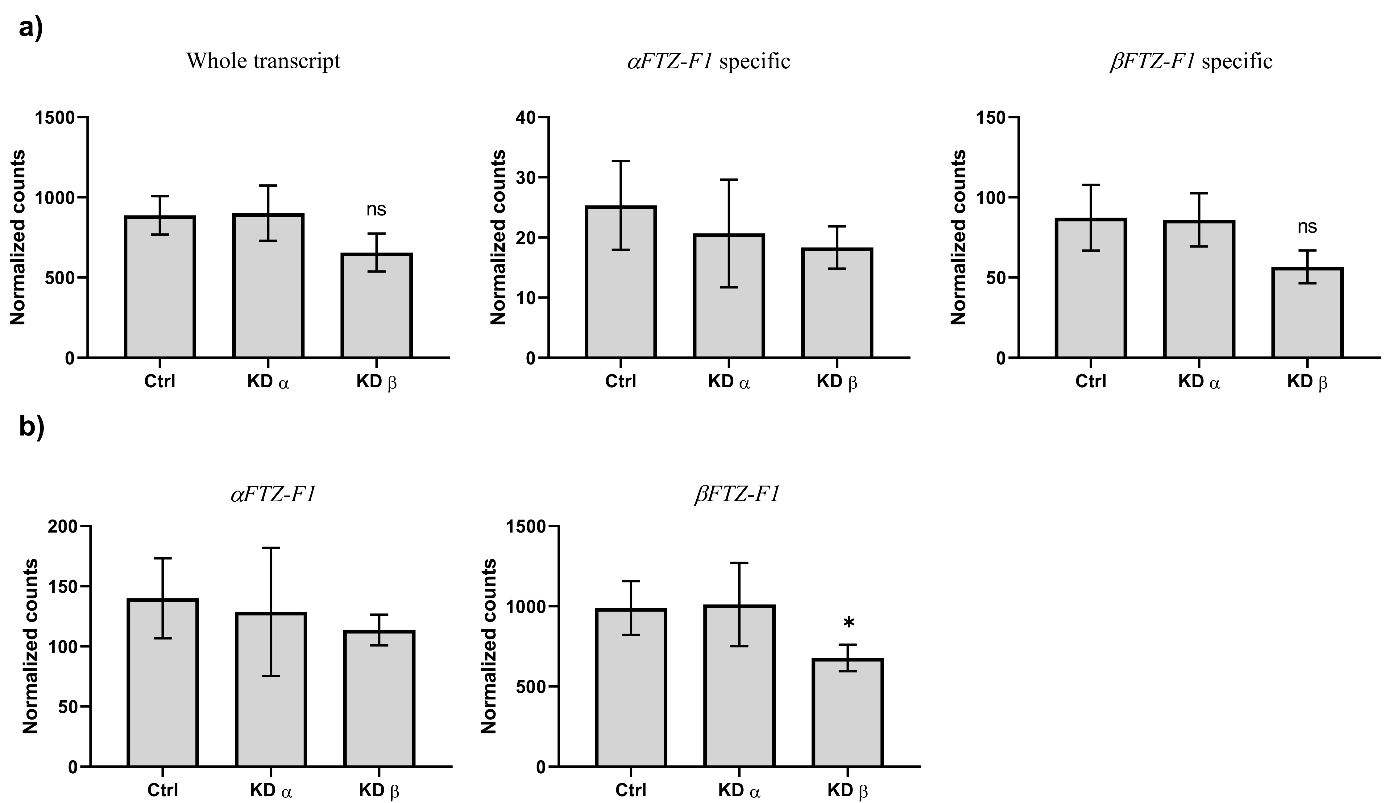
S3 Fig. Counts of *LsFTZ-F1* transcripts from mRNA sequencing with FeatureCounts and Kallisto quantification**. **a)** Normalized counts from FeatureCounts of *FTZ-F1* regions in genome annotation file; whole transcript (*αFTZ-F1 +* common region), *LsαFTZ-F1* specific region, *LsβFTZ-F1* specific region. **b)** Counts of *LsαFTZ-F1* and *LsβFTZ-F1* full-length transcripts from Kallisto quantification. Star indicates significant difference compared to control (P-adjusted value ≤ 0.05).
