# Supplementary material for: The FTZ-F1 gene encodes two functionally distinct nuclear receptor isoforms in the ectoparasitic copepod salmon louse (Lepeophtheirus salmonis)
Source: PLoS One. 2021 May 20;16(5):e0251575. doi: 10.1371/journal.pone.0251575 (PMC8136749; doi:10.1371/journal.pone.0251575)
Supplement: S4 Fig — GO terms enriched under the genes downregulated (a) or upregulated (b) after βFTZ-F1 knockdown. Shown are all terms with a q-value <0.05 for the categories biological process (BP) and molecular function (MF). No terms for cellular compartment (CC) met these conditions. Bars show the frequency of the given GO term under the selected genes (study frequency) and in the whole genome (population frequency). The enrichment compared to occurrence in the whole genome is shown as a number on top of each bar. Terms are sorted according to its q-values for each category. (DOCX) [file pone.0251575.s004.docx]

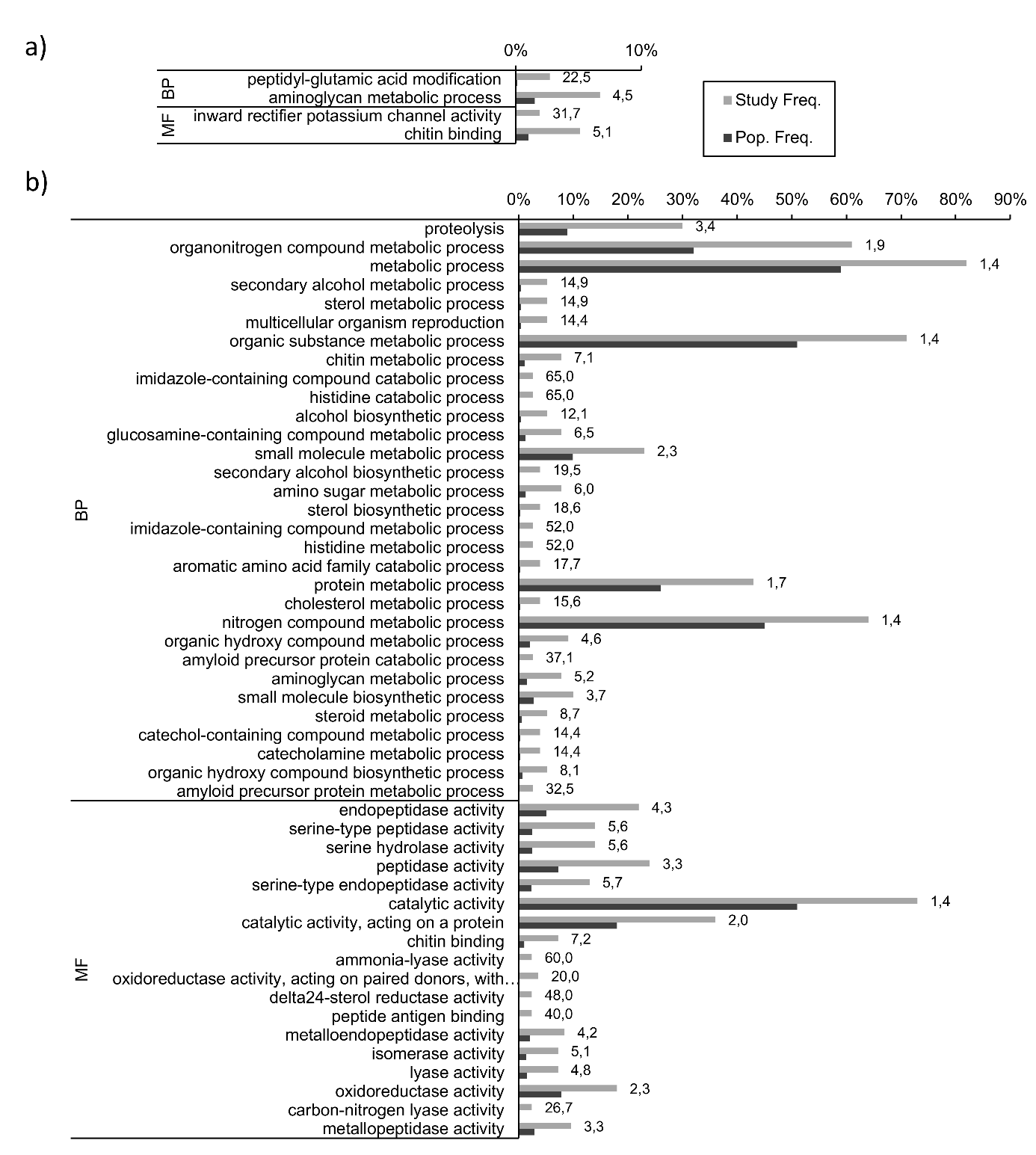


**S4 Fig. Gene ontology enrichment analysis of *βFTZ-F1* knockdown animals.** GO terms enriched under the genes downregulated (a) or upregulated (b) after *βFTZ-F1* knockdown. Shown are all terms with a q-value <0.05 for the categories biological process (BP) and molecular function (MF). No terms for cellular compartment (CC) met these conditions. Bars show the frequency of the given GO term under the selected genes (study frequency) and in the whole genome (population frequency). The enrichment compared to occurrence in the whole genome is shown as a number on top of each bar. Terms are sorted according to its q-values for each category.
