# Supplementary material for: The FTZ-F1 gene encodes two functionally distinct nuclear receptor isoforms in the ectoparasitic copepod salmon louse (Lepeophtheirus salmonis)
Source: PLoS One. 2021 May 20;16(5):e0251575. doi: 10.1371/journal.pone.0251575 (PMC8136749; doi:10.1371/journal.pone.0251575)

S1 File. Overview of structures, sequences and SRA reads of FTZ-F1 orthologues

[Insects 2](#_Toc60872903)

[Diptera (Drosophila melanogaster) 2](#_Toc60872904)

[Hemiptera (Acyrthosiphon pisum) 4](#_Toc60872905)

[Siphonaptera (Ctenocephalides felis) 5](#_Toc60872906)

[Lepidoptera (Bombyx mori) 7](#_Toc60872907)

[Lepidoptera (Manduca sexta) 8](file:///C:\Temp\Joakim\to%20send%20to%20PlosOne\S2_compact.docx#_Toc60872908)

[Lepidoptera (Spodoptera litura) 10](#_Toc60872909)

[Hymenoptera (*Apis mellifera*) 12](#_Toc60872910)

[Blattodea (Blattella germanica) 13](#_Toc60872911)

[Thysanoptera (Frankliniella occidentalis) 14](#_Toc60872912)

[Coleoptera (Tribolium castaneum) 16](#_Toc60872913)

[Myriapoda 18](#_Toc60872914)

[Myriapoda (Strigamia maritima) 18](#_Toc60872915)

[Chelicerata 20](#_Toc60872916)

[Acariformes (Tetranychus urticae) 20](#_Toc60872917)

[Araneae (Parasteatoda tepidarorum) 21](#_Toc60872918)

[Scorpion (Centruroides sculpturatus) 23](#_Toc60872919)

[Acariformes (Varroa destructor) 25](#_Toc60872920)

[Crustacea 27](#_Toc60872921)

[Branchiopoda (*Daphnia magna*) 27](#_Toc60872922)

[Maxillopoda (Lepeophtheirus salmonis) 28](#_Toc60872923)

[Amphipoda (Hyalella azteca) 28](#_Toc60872924)

[Priapulida 31](#_Toc60872925)

[Priapulida (Priapulus Caudatus) 31](#_Toc60872926)

[Tardigrade 33](#_Toc60872927)

[Tardigrada (Hypsibius djuradini) 33](#_Toc60872928)

[Nematoda 34](#_Toc60872929)

[Nematoda (Caenorhabditis elegans) 34](#_Toc60872930)

[Nematoda (Trichuris trichiura (CDW52832.1) 34](#_Toc60872931)

[Nematoda (Steinernema carpocapsae (TKR80278.1) 34](#_Toc60872932)

## Insects

### Diptera (Drosophila melanogaster)


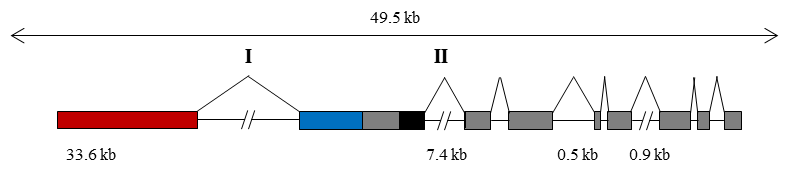


##### >NM_079419

ACTGTGTGTTGTGTTCCCGTTCGAGTTCGACCTAATTTCGCTCGAGACCTAATTTTTGCGTGCGGAATAGTTTTTCCCGAAAAAGTGAAGCCAGCGAATAAACAATAGCTGAAAGTGAGCGATAGCCAAGAAAGTAAGAGTGAAAGTAAACAAAAGCGAAAAGAAGAACAGCTCTGGCGTTAATTATGTGTGTGTACATACGTATATATTTAATATTTCCGATGCGTCCATAACGGGAGTGTGTTCTGGTTTCCAATCGTCGCCCGCGCCCCCGTCATTGCCTATCCGTTTACCAAACCCCAATTTTGGTGTGCAATTGTGTATAAAAATTAACACAAAGTGGGAAGATAGCGTTGTGTCATAGAATTGGAGCAGAAGCAAAAACAAGGCAAAATTAGCAAAAGCCAAGCGGAAAAACAACAACACAGAGTGCCCAGTGGAACCCCAGTACATGTGTGTATATATGTATGTGCGGTGCGAGCGTGTCAGTGTGTGCGTAGCAGGAATTGCAATTTATAGTTGTTGTTTCTGAGAGCTTTCGCTTGTTGCGCGCGTGCGACGATTGTGCAAGAAGTTCTTGGTGTGCGTTCGAAATCGGGAAGAGGAAATCGCCGAGAATAGAGGAAGAGTAAATGGCAATAAATGCAAAATAAATAAAAGAAATAAATAAAAAAAAATATAAATAAATTGCCAAATGCGTTTGGCGTCGCGTGTGTGTGCGTGTTCTGAAATATGCTGAAAGAGCTTTCGGAGAGCGAGAGCAAAAGAGAACGAGACGCCGAGAGATGCGATCGGGAGAAATAGCAACAACAACAACAACGGCAACAGCGACTACGATCGGCGAAATGAATCACACACAAGCTTTGTTTTGCGAATTATAAATTCGCGTTCCGTTCTCCACACATGCGAATTCCCACACTGAGAGCGAGACAGAGACAGCGAGAGAGAGGGAGACCAGCTACGGCTACAAAAACAGAAACAGCAAGAAGACCAAGACATAATAATAGAACTTTTGCTTTCGCTTCCAACTGGTTTTTGTATACATAAAAAGCGAGAGGGGAAGAGACGGAGAAAGAGAGAGACGGACAGCTTCCAGCGCTTCTTCTTCTTCCGCGATCTTCGTCTTGGAATTCATGGAACCGGGCGCATGCGCAACACACAGAACTACAAAAGGAAAAACAACGACGTCAGCTGCATGAAAGGTCTTTAAAGCAATTATAAACTTACAACTACTATCGTTACCTGCCATTAAGAATCAACAAAACAAATACCACACATCACAACGAAACGTACAAAAAATAAAAGTTAAATTATATTATTCATAAAATGGATACCTTCAATGTACCTATGCTGGCGGAGAGCAGCAACACCAACTATGCAACAGAAGCTACCAGCAATCATCACCACCTGCAACATCAGCATCAGCAGCAACATTCACATCAGCAGCAGCAGCAGCAGCAACTCCTAATGCCCCATCATCACAAGGATCAAATGCTGGCGGCGGGCAGTTCGCCCATGCTGCCATTCTATTCACACTTGCAGTTGCAACAGAAGGATGCAACTGCAACAATAGGACCTGCAGCAGCAGCAGCAGCAGTTGAGGCGGCAACAACATCGGCAAATGCCGATAACTTCAGTTCATTGCAAACGATCGATGCCAGTCAACTGGATGGAGGCATCTCCTTGAGCGGCTTGTGCGATCGTTTCTTTGTGGCCAGTCCAAATCCCCACAGCAATAGCAACATGACACTAATGGGCACTGCCACAGCTGCAACAACTACCACTACCAACAACAACAATAATAACAACACTAACAATAACAATAACAACAACGTGGAGGCTAAGACAGTGAGGCCATCGAACGGCAATTCGGTGATCATCGAATCTGTGACGATGCCATCGTTTGCCAATATTCTATTTCCCACCCATCGCAGCGCCAACGAGTGCATTGATCCCGCCCTGTTGCAAAAGAATCCCCAAAATCCAAATGGCAACAACAGTAGCATAATTGTGCCGCCCGTCGAATATCATCAATTGAAACCACTTGAAGTCAATTCGTCGACTTCCGTTTCCACCAGCAACTTCCTGTCCTCCACAACGGCCCAATTGTTGGACTTTGAGGTGCAGGTGGGCAAGGATGATGGCCACATTAGCACCACTACGACCACAGGGCCGGGCTCTGGATCGGCATCGGGTTCAGGATCGGGATCGGGATCGGGATCAGGATCGATTGCACGCACAATAGGCACTGCAACGCCCACAACGACAACATCCATGAGCAACACTGCCAATCCGACGAGGAGCTCATTGCACAGTATTGAGGAGCTGGCCGCCAGTTCCTGTGCCCCGAGAGCAGCGTCTCCCAACAGCAACCACACTAGCAGTGCCAGCACCACTCCGCAGCAGCAGCAGCAGCAGCAGCATCACATGCAAAGCGGCAACCACAGTGGCTCCAATCTCAGCAGCGACGATGAGTCCATGTCCGAGGATGAGTTCGGTCTGGAAATCGACGATAACGGAGGCTACCAGGACACCACCTCCTCACACTCGCAACAGAGCGGAGGAGGCGGTGGCGGCGGCGGTGGCAACCTGCTAAACGGCAGCTCCGGCGGCAGCTCCGCCGGCGGTGGCTACATGCTGCTCCCCCAGGCGGCCAGCTCCAGTGGCAATAATGGCAATCCGAATGCCGGCCACATGTCCTCCGGTTCCGTGGGCAATGGCAGCGGAGGCGCTGGCAATGGCGGAGCGGGCGGCAACTCCGGTCCCGGCAATCCCATGGGCGGTACGAGCGCCACGCCGGGACACGGCGGCGAGGTGATCGACTTCAAGCACCTGTTCGAGGAGCTTTGCCCCGTGTGTGGCGACAAGGTGAGCGGCTACCACTACGGCCTGCTCACCTGCGAGTCCTGCAAGGGATTCTTCAAGCGCACCGTGCAGAACAAGAAGGTCTACACCTGCGTGGCGGAGCGGTCGTGCCACATCGACAAGACGCAGCGCAAGCGGTGTCCCTACTGCCGATTCCAGAAGTGCCTCGAGGTGGGCATGAAGCTAGAGGCTGTTCGAGCGGATAGAATGCGTGGTGGACGCAACAAATTCGGACCCATGTACAAACGGGATCGCGCGCGGAAGTTGCAAGTGATGCGGCAGCGGCAGTTGGCGCTGCAAGCGCTGCGCAACTCGATGGGTCCGGACATCAAGCCAACGCCGATCTCGCCGGGCTACCAGCAAGCATATCCAAATATGAACATTAAGCAGGAAATTCAAATACCTCAGGTATCCTCACTCACCCAATCTCCGGACTCGTCGCCCAGCCCCATAGCAATTGCGTTGGGACAGGTGAACGCGAGCACGGGCGGTGTTATAGCCACGCCCATGAACGCCGGCACTGGCGGCAGTGGGGGCGGTGGTCTGAACGGACCAAGTTCCGTGGGCAACGGCAATAGCAGCAACGGCAGCAGCAACGGCAACAACAACAGCAGCACGGGCAACGGAACGTCCGGAGGAGGAGGTGGCAATAATGCGGGCGGCGGAGGAGGAGGAACCAATTCCAACGATGGCCTGCATCGCAACGGCGGCAATGGCAACAGCAGTTGCCACGAGGCTGGAATAGGATCTCTGCAGAACACGGCCGACTCGAAATTGTGCTTCGATTCTGGCACACATCCATCGAGCACAGCCGACGCGCTAATCGAGCCATTAAGAGTCTCACCGATGATTCGTGAATTTGTGCAATCTATTGACGATCGGGAATGGCAGACGCAACTGTTTGCCCTGCTGCAGAAGCAAACCTACAACCAGGTGGAAGTGGATCTCTTCGAGCTGATGTGCAAAGTGCTCGACCAGAATTTGTTCTCGCAAGTAGACTGGGCACGGAACACCGTCTTCTTCAAGGATCTGAAGGTCGACGACCAAATGAAGCTGCTGCAGCATTCCTGGTCGGACATGCTTGTTCTGGATCACCTGCATCATCGAATCCATAACGGCCTGCCCGACGAGACGCAACTGAACAATGGTCAGGTGTTCAATCTGATGAGTCTGGGTTTGTTGGGAGTGCCACAGCTGGGCGATTACTTCAACGAGCTGCAGAACAAGCTGCAGGACCTGAAATTCGATATGGGCGACTATGTCTGCATGAAATTCCTAATCCTGTTGAATCCAAGTGTACGGGGTATTGTCAACCGGAAGACCGTCTCCGAGGGACATGATAATGTGCAAGCCGCTTTGCTGGACTACACCCTCACCTGCTATCCGTCAGTGAATGACAAATTCAGAGGGCTAGTTAACATCTTACCGGAAATCCATGCCATGGCCGTTCGCGGCGAGGATCACCTGTACACCAAGCACTGTGCCGGCAGTGCGCCCACCCAAACGCTGCTCATGGAGATGCTGCACGCCAAGCGCAAGGGATAGAGGCCGGGAGAACGTGACACGGAATACTTAATCATTTATGAAATGTAAATAACAAGGCGGGAAGGCCCTCGGGGCAACCGGGTCATGGAAGGCGAACGAAGGATACAGCAGAATTCCGTATTATGAATATGGGAATGCATCATCACTACTACCACCAACTATCACACCTATACACACACATGCACACATTTGTTGATTCAATGTTAATTATTATTACGTTTACGGTTAGGTCTAGTTTACGTTTAACTAATTAATTAATTTGTCTTAAATTAATTCGTGTTTTATTTGTAGTCCCTGATAAAGCAATTTTAAAACACTTGAACCTAAACGAGAATATGTAGTAGATGTATGGATTTAAATTTAAATACGGCAAGGAGAAACACACTTTTTTAGGCATTACAAAACAAAAGAAGCATGAGAAATTTTATTTTTATATACCTATATGAATACGATACTTATGGATACAAATCTATATATATTTTTATGTAAATTGGCGTACTTTTAGCGTCCTACATATTTTTTAATTAGAATTTGGTTATACTATAGTTTTGAAATTAGTATCGTTCCCACTTGAAGATCGATTCTTGTATTTTTTTGCGCCAAGTGTCTTGCATAGTATTTGCGTCTAATCTAATGGCAACAAAAAAAATATTGGAAAATCCATACAAAGAAAATGAAAACAAAGCAAATTTAGGTGTTCATGGTATGAATGTATGTGTATATTATAATTGTAATTTCATCTAAGTGTAAGAAAACAATGCAAACAACTACCTACAACAAGATAATGAAGAGCAAGAAATTATATAAATTAATAAAGGTCGTGTTAAAAACTATATAGAAAATATATACTTACATGTATATTTAAATTTAGCAGCGAGTTTCAACTATTTGTAAATTTCAACCAAGATGTAATTGTTTCAATGTCAACAACAAACAAATTGTAAAGCTTATATTTAAAATACAAAATATATTTTGTGCAAGGAAAGCGATAAAATCC

##### >NM_168775

GGTTTAGTCAATCGAGATACGTGGTTGATGTTGGTCGTGTTTTTCGCATAGCTATCCACTTATAAATAAATATATATATATATACATGCAACAACAACAACACACACATAGATGACAAAGCGTTACACCTAACGAAATATTGTTAACAACAAATTGCGCGTGTGAGTGACCCAAATATCGATCAAAATAAACAAAAGAGCCATTGCGAAATAGATAAAAAGGCAATCGAATTGCATGCACCGAATACAATACAAATACAAATACAAGCAAAATTGAAGTCAAACCCAATTGCACCTCACCCACACCAGCAGAACAGCCCAAGGATTATGTTCTCTTGCTGCGTTGCGTGAGAGTGTGTGTGTGTCTCAGTGCAACAGTGCATCAGTGTGTGTGCAATGCAAATTGTTTAAACAACAAATCCGATTCCCACATATAGTGCAATAAATTCGTGACCCACTTTCAATCAAAATACAAAAGTGAAAATATACTAAGCAAGCCAAAAAGTGTTTATACATCTCGTTTCAATTCAACGCACACAATCAAGCTTATATATAAAGTATATAAAAAAGTCCTACCTCTATCCAGGCAAACCAACCAAAAGTTTATAAAGAAATCATCCTTTTTGCTGTCTACTCTACATATAAATAAATATACATACATATATCTCACTCACCTCGTCAAAGTACCTGCAAAAAAAAGTAACAATTTAAGCAAAAGTAAAATTAAAAATAAACAAAGCAAAAAGGAAAACATCTTATCAAATGCAAAATAAATGTCAAGGAACACACAGCGAGTTTGTTAAATGATTTTCTTGTAAAGGAAATCGATCAATTAACAATAAAAAATTACACATCTTCTAAAGAACCAAACAATTCCAAACATTTATAAAATACATTTCTTTCAAAGAAAGTAAAACATAAATAGTTGTAAAAGCAAGTAAAACACCATTAAATTACTGTGTTAAACGAATATTAAATATGTCAAGATATTACGATACATTTTAACTATCGACGTTGAAATCAAATACAAGCAAATACAATTTGTTGTCAGAACCCCAAATATTCTGTTCAACAATTGGCCTTAACAATGTTGCAAATAACAAATCTGCAAAAATTAAGTGCAAAAACCAGACCACCGTATGTTAACCGCAACCTATTTTACTACCAAATTTAAACCAACTCTGCAACACAAGTGCTGGTAATTGAGCCAACTAAAGCGAAAAACAATTTAACCTTCCGTCCAGGAAATAAAACAACAAAGGATAACTCTTCGGTCTTCTAAAAACGGAAGTTTAAAACCGAAACCGAAAAAACCAAAATCACTCGCCTTGAATTCTACGCAAAATAAAACGTACATGAAATGTTATTAGAAATGGATCAGCAACAGGCGACCGTACAGTTTATATCGTCGCTGAATATATCGCCGTTCAGCATGCAGCTGGAGCAGCAGCAGCAGCCCTCCAGTCCCGCTCTGGCCGCCGGTGGCAACAGCAGCAACAACGCGGCCAGCGGTAGCAACAACAACAGCGCCAGCGGCAACAACACCAGCAGCAGCAGCAACAACAACAACAACAATAACAACGACAATGATGCACACGTTCTAACGAAATTCGAGCACGAATACAATGCCTACACGTTGCAGTTGGCCGGAGGCGGTGGGAGTGGCAGCGGCAATCAGCAGCACCACAGCAACCACAGCAACCACGGCAACCACCACCAGCAGCAGCAGCAACAACAGCAACAGCAGCAGCAACATCAGCAGCAGCAGCAAGAACACTACCAGCAGCAACAGCAACAGAATATCGCCAACAATGCCAATCAATTCAACTCCTCGTCCTACTCGTATATATACAATTTCGATTCACAGTATATATTCCCGACAGGCTACCAGGACACCACCTCCTCACACTCGCAACAGAGCGGAGGAGGCGGTGGCGGCGGCGGTGGCAACCTGCTAAACGGCAGCTCCGGCGGCAGCTCCGCCGGCGGTGGCTACATGCTGCTCCCCCAGGCGGCCAGCTCCAGTGGCAATAATGGCAATCCGAATGCCGGCCACATGTCCTCCGGTTCCGTGGGCAATGGCAGCGGAGGCGCTGGCAATGGCGGAGCGGGCGGCAACTCCGGTCCCGGCAATCCCATGGGCGGTACGAGCGCCACGCCGGGACACGGCGGCGAGGTGATCGACTTCAAGCACCTGTTCGAGGAGCTTTGCCCCGTGTGTGGCGACAAGGTGAGCGGCTACCACTACGGCCTGCTCACCTGCGAGTCCTGCAAGGGATTCTTCAAGCGCACCGTGCAGAACAAGAAGGTCTACACCTGCGTGGCGGAGCGGTCGTGCCACATCGACAAGACGCAGCGCAAGCGGTGTCCCTACTGCCGATTCCAGAAGTGCCTCGAGGTGGGCATGAAGCTAGAGGCTGTTCGAGCGGATAGAATGCGTGGTGGACGCAACAAATTCGGACCCATGTACAAACGGGATCGCGCGCGGAAGTTGCAAGTGATGCGGCAGCGGCAGTTGGCGCTGCAAGCGCTGCGCAACTCGATGGGTCCGGACATCAAGCCAACGCCGATCTCGCCGGGCTACCAGCAAGCATATCCAAATATGAACATTAAGCAGGAAATTCAAATACCTCAGGTATCCTCACTCACCCAATCTCCGGACTCGTCGCCCAGCCCCATAGCAATTGCGTTGGGACAGGTGAACGCGAGCACGGGCGGTGTTATAGCCACGCCCATGAACGCCGGCACTGGCGGCAGTGGGGGCGGTGGTCTGAACGGACCAAGTTCCGTGGGCAACGGCAATAGCAGCAACGGCAGCAGCAACGGCAACAACAACAGCAGCACGGGCAACGGAACGTCCGGAGGAGGAGGTGGCAATAATGCGGGCGGCGGAGGAGGAGGAACCAATTCCAACGATGGCCTGCATCGCAACGGCGGCAATGGCAACAGCAGTTGCCACGAGGCTGGAATAGGATCTCTGCAGAACACGGCCGACTCGAAATTGTGGTATTTAGCCCAAAAGCCAGAACAAAACTCAATGAATATTCGAACTCAAACCAAATTAAACGACAAACGAACTTCAACAAACATTGAAAAGAAACTTTCGTTACATAAGCATATTTGTGGAAAAGGATTCAACCTGATCATGCAGCAAGTCTTCGATTCTGGCACACATCCATCGAGCACAGCCGACGCGCTAATCGAGCCATTAAGAGTCTCACCGATGATTCGTGAATTTGTGCAATCTATTGACGATCGGGAATGGCAGACGCAACTGTTTGCCCTGCTGCAGAAGCAAACCTACAACCAGGTGGAAGTGGATCTCTTCGAGCTGATGTGCAAAGTGCTCGACCAGAATTTGTTCTCGCAAGTAGACTGGGCACGGAACACCGTCTTCTTCAAGGATCTGAAGGTCGACGACCAAATGAAGCTGCTGCAGCATTCCTGGTCGGACATGCTTGTTCTGGATCACCTGCATCATCGAATCCATAACGGCCTGCCCGACGAGACGCAACTGAACAATGGTCAGGTGTTCAATCTGATGAGTCTGGGTTTGTTGGGAGTGCCACAGCTGGGCGATTACTTCAACGAGCTGCAGAACAAGCTGCAGGACCTGAAATTCGATATGGGCGACTATGTCTGCATGAAATTCCTAATCCTGTTGAATCCAAGTGTACGGGGTATTGTCAACCGGAAGACCGTCTCCGAGGGACATGATAATGTGCAAGCCGCTTTGCTGGACTACACCCTCACCTGCTATCCGTCAGTGAATGACAAATTCAGAGGGCTAGTTAACATCTTACCGGAAATCCATGCCATGGCCGTTCGCGGCGAGGATCACCTGTACACCAAGCACTGTGCCGGCAGTGCGCCCACCCAAACGCTGCTCATGGAGATGCTGCACGCCAAGCGCAAGGGATAGAGGCCGGGAGAACGTGACACGGAATACTTAATCATTTATGAAATGTAAATAACAAGGCGGGAAGGCCCTCGGGGCAACCGGGTCATGGAAGGCGAACGAAGGATACAGCAGAATTCCGTATTATGAATATGGGAATGCATCATCACTACTACCACCAACTATCACACCTATACACACACATGCACACATTTGTTGATTCAATGTTAATTATTATTACGTTTACGGTTAGGTCTAGTTTACGTTTAACTAATTAATTAATTTGTCTTAAATTAATTCGTGTTTTATTTGTAGTCCCTGATAAAGCAATTTTAAAACACTTGAACCTAAACGAGAATATGTAGTAGATGTATGGATTTAAATTTAAATACGGCAAGGAGAAACACACTTTTTTAGGCATTACAAAACAAAAGAAGCATGAGAAATTTTATTTTTATATACCTATATGAATACGATACTTATGGATACAAATCTATATATATTTTTATGTAAATTGGCGTACTTTTAGCGTCCTACATATTTTTTAATTAGAATTTGGTTATACTATAGTTTTGAAATTAGTATCGTTCCCACTTGAAGATCGATTCTTGTATTTTTTTGCGCCAAGTGTCTTGCATAGTATTTGCGTCTAATCTAATGGCAACAAAAAAAATATTGGAAAATCCATACAAAGAAAATGAAAACAAAGCAAATTTAGGTGTTCATGGTATGAATGTATGTGTATATTATAATTGTAATTTCATCTAAGTGTAAGAAAACAATGCAAACAACTACCTACAACAAGATAATGAAGAGCAAGAAATTATATAAATTAATAAAGGTCGTGTTAAAAACTATATAGAAAATATATACTTACATGTATATTTAAATTTAGCAGCGAGTTTCAACTATTTGTAAATTTCAACCAAGATGTAATTGTTTCAATGTCAACAACAAACAAATTGTAAAGCTTATATTTAAAATACAAAATATATTTTGTGCAAGGAAAGCGATAAAATCCAATTATAACAATTTAAAAAAAGCTGTATAACACTTTAAAACTAAAAGTAAAAGATGTTTAGTCATGAATTTGGGCAATGAGGCGGAAAAGAAATCGAATTAAAGAAGAACTTGAATATTGTAAAAATCAAAATCGGAATAAACAAACCAAATAAACTATTTTTAATGAATATCTATTAATGGGTCAACTTTTAATTCGAATATTAAGTTACCTGGCAAAGGATTCAAAAATTGTTCAATTAGTTAATGTAATGACCCAAAAGCTTCTCGGAATTTCAAAATTTGCATTTAATTTGTTCGGATCTAATCATTGCCTTAATTCTCGCCTAGGCATTGCGCTTAAACAAAACGTACCTTATAAAATTAAAAAAAAGTTTAAGCTCTAAATTCAGTAAACAGAAACAAATTACCACACACATTTCAAATTTAAAGGCAGCGTGTAAAAAAAAACAAACGTTTAACGAGGTGTAAAAAATCAGAGGTAGCAGAAAGATTCACGAAAATAATATAAAATTCTGGCAGACGCAAGTTTAGTAGGGGACAAAAAATACAAAAAACGAAAGCACGACAACATCTATATAGGTTTGCCTAGGTATATAATATATATTTAGGTCTCTAGCTGGTATGTCTAACTTCTATATGTATTATAAAGTGTAAAAAAGTAAAGAAAATATGGGTAAATAATGTAAAAGTTGGCAATGCGAGTTGGCTAGATAGTGGATCAGTTATATTTAATGATTATAAGTAAAGGGCAAAATTGTTGACGTCTAACGTAACGCTGGCGGCATGTTGTTGAAAACACGTTAAAAAAAAAAGGGAGGAGATGAAGGGATCGATATACACATACAAATATATAAATGTTTATTCAGGTATGAATGGCGTTAGGTATGTTTATTGTAAAATTGATTACGATGTGAACCTGTTATTGCAAATGAAAAGTGATTTTAACTATTAAGAAATCTATATATATACTATAAACCCATAAAAAGTAAAAGGATCTAACAGTTATACATTAAATGATTAATATAAATGCAAGCAACACACACACTTTAAGTTTGAAAAACTGTTAAAAATGCAAAAAAAAAAAATCATTTAACTAGAAACAAAACTACCTAAAACTACAATGCATATTTGATCAATTGA

### Hemiptera (Acyrthosiphon pisum)


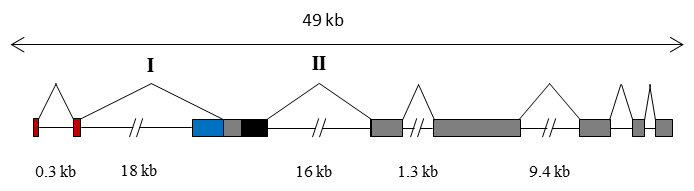


Confirmed reads in RNA seq embryo SRX6630196

Query 60 CCTCAGTAATTTGAACGAAGATGATCAAACATTCATTGAAGATTCGAAGTGCATGGACTT 119

||||||||||||||||||||||||||||||||||||||||||||||||||||||||||||

Sbjct 100 CCTCAGTAATTTGAACGAAGATGATCAAACATTCATTGAAGATTCGAAGTGCATGGACTT 41

Query 120 GAGTCATTTGAGTGACGATGATCCTAATTTCATTGAAGGT 159

||||||||||||||||||||||||||||||||||||||||

Sbjct 40 GAGTCATTTGAGTGACGATGATCCTAATTTCATTGAAGGT 1

##### >XM_008185073.2_Acyrthosiphon_pisum_aFTZF1_X2

AATGGGTATCTATATATTATATCATAGTATTTACATTTTTCAGATCAAAAGTGCATGGACCTCAGTAATTTGAACGAAGATGATCAAACATTCATTGAAGATTCGAAGTGCATGGACTTGAGTCATTTGAGTGACGATGATCCTAATTTCATTGAAGGTGGAAACGGCGGCGGCGGAGGAGGAGGCGGAGTGTCCGGCTCCATGGACGGCCTCCAGCAGTACGTGGCCGTCGGCGGAAACGGCGGCGGTACGCCCGTGTCCGCGGCCGCAGCCGCCGTCAACCCGTCGGCCACTGGTTCCGAGCTGCCCGACACTAAAGAGGGCATCGAAGAACTGTGTCCCGTGTGTGGTGACAAGGTGTCCGGTTACCATTACGGTCTGCTTACGTGTGAGTCATGCAAAGGCTTTTTCAAGCGGACCGTGCAAAACAAAAAGGTGTACACGTGCGTGGCCGAGCGGTCGTGTCACATCGACAAGACCCAAAGGAAGAGGTGTCCATTCTGCCGATTCCAGAAATGTCTCGAAGTCGGCATGAAACTCGAAGCCGTCCGAGCGGACAGGATGCGAGGAGGCCGAAACAAATTCGGGCCCATGTACAAACGGGACAGGGCGAGAAAGCTGCAGATGATGCGTCAGCGACAGATGGCCGTGCAAACGTTGCGCGGCGGCGTGGTCACGTCGGCCGGGGCCAACAACCACCACCAATTCGCCATGTTGGCCGGCGACGCGGTCACGCTAGGCCAGTACCCTGGTTCTCCCATCACATCCTCGGTGTCCAGTTTGCACATCAAACAGGAAATTCAGATTCCCCAGGTGTCTTCGTTGACATCCTCGCCCGACTCGTCTCCCAGCCCGATCGCCGTGGCTCTGGGCCAGGCCGGACTCGGGTCTCTGACCACGGGCGCCGGCGGCACCACGTGTGTGGTCAACCAACACTCACAGATGACGCTGCAAATCGTGGCTTCGGCCAACGGCAACAACAATAATAACAACACTAGCAGCAGTAACAACAACAACAATAGCACGAGCGTGACCAACATGACCGGTACAAACGGTGGAGTCCCGCCGTCGCAACACCAGCAACATCACCAGCAGGCACAACAACAGCAACAGCAGCAGCAACAGCAGTCGGTAGTACCGGCCTCGAACTTGTTGTCCGTAAACAAAATGTGGTCGCCACCGTCGTCGCCTAAGAACTACCTGTTGTCTACGCACCAGCAGCAACAGCAGCAACACCACGTGCCACAGCAGCAACAGCAGCAACAACAGCAACAGCAGATGGTGGTGGACGGTGGCGGTAGCTCGTCGACCGCCGCGACTGCTAATGGTGGCGGCAGCGGCGTCAAAGGCATGTCGCCCATGATCCGGGACTTCGTGCAGGGACTGGACGACCGGGAGTGGCAGAGTTCACTGTTTGGCCTGTTGCAAAACCAGACGTACAACCAGTGCGAGGTCGATCTCTTCGAACTCATGTGCAAGGTGCTCGACCAGAATCTATTCTCGCAGGTGGACTGGGCGCGGAATTCGGTGTTCTTCAAAGATTTGAAGGTGGACGACCAGATGAAACTGTTGCAGCACTCGTGGTCGGACATGCTGGTGCTGGATCACATCCACCAGCACATGCACAACAACCTGGCCAACGAGATCATGTTGCACAACGGGCAAAAGTTCGATCTACTCGCTCTTGGTCTACTTGGCGTCACCACGATGGCTGATTGTTTCGCCCAGGTCATCGATAGGTTACAGGAGCTCAAGTTTGACGTTGGCGATTACATCTGCTTAAAGTTCCTGCTGCTGTTGAACCCCGATGTCAAAGGGATTGGCAACAGTAAACACGTTCACGAAGGTCACGAGCAAGTGTTGAAGGCTCTCTTGGACTATTGCATCACAGCCAACTCTCAAGTACAGGACAAATTTACCAAATTGTTGTCCGTACTACCCGAAATCCATCACATAGCCACCAGGGGCGAGGAGAGTCTGTACTTGAAGCACTGTAGTGGCGGCGCTCCCACGCAGACGTTGCTCATGGAGATGCTGCACGCCAAACGAAAATAACAAAAATCACACAAGACACACATACACACACACTACTCACCACCAAAATATTATATATTTTATAACAATAATAGCGTATATACTATATACTGCTGTTATTATTATTATAATAATGACAATATATTATTATTATTATTATTTTATTTAAAATATATATATTTAGATGTCCACACACAACACACCATCACCACCACTACCACCCCTTACACACACACACACACACACACACACACTCATGCCCACAAAGTCAAATCGACATCATCTGCAATGCGAAGAACAGACACCACACCGCACGGCACTCCTCGCACTCGTTCCACGGTTGGCCAAATATTTTAAAAGACAGACCCCGTCAACGTCTATTTGAGCGACAGCCACGTTACCATGACAACTAAATAAATTTTGAGTCATTACAGAGTTACCAACTGATCTCGTCGACAGCGTCATTACACACGTACGCGATGTTGGTCAAAACTACTCGTACACACCACATACACTGATGTACACAACATACACCAATACACACACATACACAAATACACACCACATAATTATTAATATTATTTATTATTAATTATTTAACTTATGTTTTTTTTTATATATATTATATTATAAATGTAAAAAAAGTAATCCTCCCGATGACTAATGAAATAAGAGTGCACTATGTGCGCCCAAAAAATCCGATTATTATTATTATTATTATTAAGTACATATGTTATGTTTATATAGTGCATATTATATATTATAGCATAATGCGTATGTATACATATATTTTTATTATGAGCGATATAGTTTGTATATATATTATTATAATGATTTTAATGCTTAGCTTAGGTATTTAATGTTTGGTTACCAAATCTCAAAAGGCCAGTAAAAGGAGATGTGCTCAAGATTTAAAAATGTTTAAAAAAGATTATATTATCTACAATATAATACAAGCCGTTCAGTTCCTAAACACTGTTCCAATTTTAAATAAGATATTAATCCGCTATGTTTGGTTTTCTCCTTCTTTGTATTAATTGCAGATATTTTGTTCATGAAAGAGATATATAAGTGAGACCCATATGCAAAGAAAGAATGACTACAATAAATGTG

Confirmed reads in SRX7004795

Query 920 GTGGGTGCCCCTCCGGGACGCCCTGTACGTACATCATATGTTAGCAGCCGGCATGGATCA 979

||||||||||||||||||||||||||||||||||||||||||||||||||||||||||||

Sbjct 75 GTGGGTGCCCCTCCGGGACGCCCTGTACGTACATCATATGTTAGCAGCCGGCATGGATCA 16

Query 980 CTCTGCCAGTTATCT 994

|||||||||||||||

Sbjct 15 CTCTGCCAGTTATCT 1

##### >XM_008185072.2

AGCCGTTTCGTCGTGCCGTCCGTGTCAGTGTGTCTGTGTGTGTGGTATCCGATCTTACGGCCACGGCTAACCTGACTCTGCCGCGAAGTTTCGTACATGTGTTCGTCGAGCGCGCTCGATTCGTTGTAAAATAAAATACATCGTCTCGCGGTACTTGTAAAATTTTTTTTTTCAAAAATTGTATCGCTTTATTTTACGCTTAAAATATTTTTTATTTATATTTTTAATTTTCGAGTTAGTGTGTATATTATACGATTATAATATAATTCGGAAAAATCGTTTTTATAATATTAATAATAAATAGCCGTTTATTTATTACTTTAATAATTTTCGGAATTTATTCGTGTTTGGTCACCGCCGTTGTCGTCGTCGTCGTCGTACAGTGTCCGCGCGCGCCAGTAGTGTGTCGGTCCCCCACCGGTCCCGGTGCGTGTAGCGTGTAGCGCGCGCCCGTGTGTTGTGTGTTCGTGTAGCGCGCGCGCGCCCGTGTGTGTGTGTGTGTGCGCCGAGCCGCGCAACGATTGCGTTTCGTCGTCGTTGTATAGTCGTCGTCGCCCGATTCAGTCTCACAGTGACGAAATCGGACCGGGCGCGTTGTTGTGCAAACGAGTCTCGCCAACCTGCAGTGCAATAGCCATATTATAGATATGACGAAAGCCGACAGCTAGCCGCCCCCCGCGTCCGCGCGTGGCCAACAACTATATACAGCGACGACATTCCAGCGCGACACCGTCGCAACGGCCGTCGTTGTACCAACAAACCCGCCGAAGAACTTTTTCCGGGTATTGTAAGGTCTTTTTCGTGCCTGACGATCACCCCATAACCACTACTCCCCAACCAACACCCTGCAATAGTACATTAGTAAAATATGCTCTCGAGCAGCCCTTATTCGTGACGAAAAGTGCGATCCGCGTGTCAAGTGGGTGCCCCTCCGGGACGCCCTGTACGTACATCATATGTTAGCAGCCGGCATGGATCACTCTGCCAGTTATCTGTCCCATTTCGGGCTCAGCCCGCCACAGGCGTCCGGCGATGACGGCGGCGACGATTCGCTGTACCAGCCGCCCGCCTCATCGCCGCCCCTGTACCAGTACATGCCGCCCCAGCACCAAACGCCCTCATCCAGCGCGGCCGCCGCGTACTCGATGATGGGTGGCAGTAACCAAGGCTGTTATCAGCTCGGTAATGTGGACACAGCGTATTTGTTCGGTTCCACAGGTGGAAACGGCGGCGGCGGAGGAGGAGGCGGAGTGTCCGGCTCCATGGACGGCCTCCAGCAGTACGTGGCCGTCGGCGGAAACGGCGGCGGTACGCCCGTGTCCGCGGCCGCAGCCGCCGTCAACCCGTCGGCCACTGGTTCCGAGCTGCCCGACACTAAAGAGGGCATCGAAGAACTGTGTCCCGTGTGTGGTGACAAGGTGTCCGGTTACCATTACGGTCTGCTTACGTGTGAGTCATGCAAAGGCTTTTTCAAGCGGACCGTGCAAAACAAAAAGGTGTACACGTGCGTGGCCGAGCGGTCGTGTCACATCGACAAGACCCAAAGGAAGAGGTGTCCATTCTGCCGATTCCAGAAATGTCTCGAAGTCGGCATGAAACTCGAAGCCGTCCGAGCGGACAGGATGCGAGGAGGCCGAAACAAATTCGGGCCCATGTACAAACGGGACAGGGCGAGAAAGCTGCAGATGATGCGTCAGCGACAGATGGCCGTGCAAACGTTGCGCGGCGGCGTGGTCACGTCGGCCGGGGCCAACAACCACCACCAATTCGCCATGTTGGCCGGCGACGCGGTCACGCTAGGCCAGTACCCTGGTTCTCCCATCACATCCTCGGTGTCCAGTTTGCACATCAAACAGGAAATTCAGATTCCCCAGGTGTCTTCGTTGACATCCTCGCCCGACTCGTCTCCCAGCCCGATCGCCGTGGCTCTGGGCCAGGCCGGACTCGGGTCTCTGACCACGGGCGCCGGCGGCACCACGTGTGTGGTCAACCAACACTCACAGATGACGCTGCAAATCGTGGCTTCGGCCAACGGCAACAACAATAATAACAACACTAGCAGCAGTAACAACAACAACAATAGCACGAGCGTGACCAACATGACCGGTACAAACGGTGGAGTCCCGCCGTCGCAACACCAGCAACATCACCAGCAGGCACAACAACAGCAACAGCAGCAGCAACAGCAGTCGGTAGTACCGGCCTCGAACTTGTTGTCCGTAAACAAAATGTGGTCGCCACCGTCGTCGCCTAAGAACTACCTGTTGTCTACGCACCAGCAGCAACAGCAGCAACACCACGTGCCACAGCAGCAACAGCAGCAACAACAGCAACAGCAGATGGTGGTGGACGGTGGCGGTAGCTCGTCGACCGCCGCGACTGCTAATGGTGGCGGCAGCGGCGTCAAAGGCATGTCGCCCATGATCCGGGACTTCGTGCAGGGACTGGACGACCGGGAGTGGCAGAGTTCACTGTTTGGCCTGTTGCAAAACCAGACGTACAACCAGTGCGAGGTCGATCTCTTCGAACTCATGTGCAAGGTGCTCGACCAGAATCTATTCTCGCAGGTGGACTGGGCGCGGAATTCGGTGTTCTTCAAAGATTTGAAGGTGGACGACCAGATGAAACTGTTGCAGCACTCGTGGTCGGACATGCTGGTGCTGGATCACATCCACCAGCACATGCACAACAACCTGGCCAACGAGATCATGTTGCACAACGGGCAAAAGTTCGATCTACTCGCTCTTGGTCTACTTGGCGTCACCACGATGGCTGATTGTTTCGCCCAGGTCATCGATAGGTTACAGGAGCTCAAGTTTGACGTTGGCGATTACATCTGCTTAAAGTTCCTGCTGCTGTTGAACCCCGATGTCAAAGGGATTGGCAACAGTAAACACGTTCACGAAGGTCACGAGCAAGTGTTGAAGGCTCTCTTGGACTATTGCATCACAGCCAACTCTCAAGTACAGGACAAATTTACCAAATTGTTGTCCGTACTACCCGAAATCCATCACATAGCCACCAGGGGCGAGGAGAGTCTGTACTTGAAGCACTGTAGTGGCGGCGCTCCCACGCAGACGTTGCTCATGGAGATGCTGCACGCCAAACGAAAATAACAAAAATCACACAAGACACACATACACACACACTACTCACCACCAAAATATTATATATTTTATAACAATAATAGCGTATATACTATATACTGCTGTTATTATTATTATAATAATGACAATATATTATTATTATTATTATTTTATTTAAAATATATATATTTAGATGTCCACACACAACACACCATCACCACCACTACCACCCCTTACACACACACACACACACACACACACACTCATGCCCACAAAGTCAAATCGACATCATCTGCAATGCGAAGAACAGACACCACACCGCACGGCACTCCTCGCACTCGTTCCACGGTTGGCCAAATATTTTAAAAGACAGACCCCGTCAACGTCTATTTGAGCGACAGCCACGTTACCATGACAACTAAATAAATTTTGAGTCATTACAGAGTTACCAACTGATCTCGTCGACAGCGTCATTACACACGTACGCGATGTTGGTCAAAACTACTCGTACACACCACATACACTGATGTACACAACATACACCAATACACACACATACACAAATACACACCACATAATTATTAATATTATTTATTATTAATTATTTAACTTATGTTTTTTTTTATATATATTATATTATAAATGTAAAAAAAGTAATCCTCCCGATGACTAATGAAATAAGAGTGCACTATGTGCGCCCAAAAAATCCGATTATTATTATTATTATTATTAAGTACATATGTTATGTTTATATAGTGCATATTATATATTATAGCATAATGCGTATGTATACATATATTTTTATTATGAGCGATATAGTTTGTATATATATTATTATAATGATTTTAATGCTTAGCTTAGGTATTTAATGTTTGGTTACCAAATCTCAAAAGGCCAGTAAAAGGAGATGTGCTCAAGATTTAAAAATGTTTAAAAAAGATTATATTATCTACAATATAATACAAGCCGTTCAGTTCCTAAACACTGTTCCAATTTTAAATAAGATATTAATCCGCTATGTTTGGTTTTCTCCTTCTTTGTATTAATTGCAGATATTTTGTTCATGAAAGAGATATATAAGTGAGACCCATATGCAAAGAAAGAATGACTACAATAAATGTG

### Siphonaptera (Ctenocephalides felis)


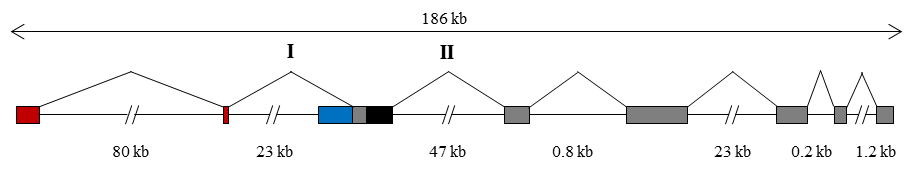


Reads found in SRX4519023

READ ID:SRA:SRR7657391.10724067.1

Query 385 GCATGAGGAGGCTAAAGGAATGAGCGTTCCGAACTCTGTCGCCAGTTCCACAAGTCAGCA 444

||||||||||||||||||||||||||||||||||||||||||||||||||||||||||||

Sbjct 1 GCATGAGGAGGCTAAAGGAATGAGCGTTCCGAACTCTGTCGCCAGTTCCACAAGTCAGCA 60

Query 445 AGACGCTGGGGCTGCGGTGACGGTAGCCTCCGATGAGACCT 485

|||||||||||||||||||||||||||||||||||||||||

Sbjct 61 AGACGCTGGGGCTGCGGTGACGGTAGCCTCCGATGAGACCT 101

##### >XM_026619028.1

TAACATTTGTCATTCAAGTGATTGATTATCGATATAGATTTAAATAAATTGTATTTATTTAGAATTTCGAATTAAATTTTTTATTAGGTTATCCTAAAATCCTGTGATGTAGCTTGTATAACATGACATATATTTATATAGCACACACACAACTATCCCAATGTCAGCTCATGCTCGCAGTCGCTGGCTTGACTTTGTAGTCATGTATGTAAAGTGTAAGTGAGTGGCCTACAGACCTACTTTTCCAAAACGTTCATTTTTATATAACACATAATATTTTTGTAAAGGACCTTGGCACAAAAAACTTCATAATACATCTGTAGTTGTATCAAAGAGCTGGATTTAATGAACAAAGTGCGTGGTGTTTATAATTGTGTGAAATATGCATGAGGAGGCTAAAGGAATGAGCGTTCCGAACTCTGTCGCCAGTTCCACAAGTCAGCAAGACGCTGGGGCTGCGGTGACGGTAGCCTCCGATGAGACCTCGATATCGGCCGTAGATCCCCTTTTGGGAGACGGGGTCCCGGACCAGGCAGACTTAGAAATAAAAATGGCATTCGAGGAGAGCTCGACAGGTGGGGCAGTTGTGATTATTACCAAAGATGAAAGCGATGGAGCTGCTGGTTTCCCGATGGAGGGCGCGGGTTCTGGTAGCGGCCCGCCTGGATACATGGGCCTGCTGCCTGGAACGCCGGCCACCACTGCGCAATGCGATCAGACGGAAATGAAAGACGTCATCGAGGAGTTATGTCCCGTATGCGGTGACAAAGTATCGGGATACCATTACGGTCTCCTAACATGTGAATCCTGTAAAGGGTTCTTCAAGCGAACTGTACAAAACAAAAAGGTCTACACGTGCGTTGCAGAACGCAGTTGTCATATCGACAAAACGCAAAGGAAAAGGTGTCCGTTCTGTAGGTTCCAGAAATGCCTTGAAGTCGGCATGAAACTTGAAGCCGTCCGAGCAGATCGTATGCGAGGTGGTCGAAATAAATTTGGGCCAATGTACAAACGAGATAGGGCCCGAAAACTACAGATAATGAGGCAACGACAGCTTGCGATACAAGCGATCAGAGGATCATTAGGAGGCCCAGGAGATGTTTACGGCCAAGGAAGTACATCACCTTATGCAAACCTGCACATCAAACAAGAAATACAAATACCACAGGTGTCCTCATTAACGTCATCTCCCGATTCATCACCCAGTCCCATCGCGGTCGCCCTCGGTCAGGTCGGGCAGACGACGATAACGGCCGCGCCGAACATCGGCGGTATAGGACCTCCATCCACGACCCACCACCACAATAACAGTGGAGCACCTGCCGGCAGCACGCCTCAACAGCCAACGAGCGCCTCTCAATCCGATTCAAAACACTGGAATGCGGCGAATTCGACGACGCATTCACTCTCACCTAAACAGTACTCATATGACCCACCTGCAGCAGGAACAGAATCCGCCAATTCAACGACGGAACCTCTCCGAGTATCACCTATGATTAGGGAATTTGTACAATCGGTCGATGATCGAGAATGGCAGAATTCACTGTATGGACTTCTACAAAACCAAACTTATAATCAATGTGAAGTTGATTTGTTTGAGCTGATGTGCAAGGTGTTAGACCAGAATTTATTCTCGCAAGTCGATTGGGCGAGGAATACGGTGTTCTTTAAAGATCTCAAGGTGGACGATCAGATGAAACTGCTGCAACACTCGTGGTCCGATATGTTAGTCCTGGACCACATGCACCAGCGCATGCACAACTCCCTGCCGGACGAGATGTCACTGCCTAATGGACAGAAGTTCGATCTTCTCTGCCTTGGGCTACTGGGCGTTCCGGCACTGGCAGCGCCCTTCAACGAATTACAAGCCAAACTGCATGAACTTAAATTTGACGTCGGAGATTACATTTGCGTCAAATTCCTGCTTCTCCTCAATCCTGATGTCCGCGGTATAACGAACAGGAAGCATGTGGTGGAAGGTTACGAGACTGTTCAAGCAGCCCTACTAGACTACACATTGACATGTTATCCATCTGTAACGGATAAATTCACAAAATTGGTTTCGATGGTACCTGAAATTCATGCGATGGCCACCATGGGCGAGGATCACCTGTACCTGAAACATTGCGCTGGCAGCGCGCCCACGCAGACGCTGCTCATGGAAATGTTACATGCGAAACGAAAATGAGGCGTTTCTCATCTGACATCCGACTCTGTCGGACATGGACAAGTTCTGCAGGAGTGATTGTCAACCCAATTCGCCCATTAAGCGCCATTCGATGGGCGTCCTTAAAAGCGGATTGAGGTATTTGGCTAAACTGCATAAATGTAAATAAATTTACTCGAATCCCGATACAGAAGAGCCCCAGTGTTGTGTTCAAGAAAACAGAAAATCTAACTAAACCGTAATTATGATATGACAAATTAATGTTATGTGTAAAGAATAAAAATAAATCGCGGGCGCGATCAGGACCACGCGGTCGGTATATTATGTTGTGTGAACTAAAGGACAGTTTAAAGACATTTTCGGACTAGTTTCTTTAATTAGTAGGTTCATTAATTTATAGGTTTCCAGTGTATGTTAATATATAATAGATAACACTAGATTAAGTAAGGAGGCACTTATTTTTTTATTAACAATGGTTACCAAACTGCTCAGTGTAAAACACCTTAAAACAGCGTCGAACAAAAATTAACTTATGTCTGCCTAATTCCTATCTGTTAATTTATTGTTACAATGCGCTGGAACAAGAGATATATAATGTTACCAAGAGTGTGTATAGTCCACGTTGTTGTACGTGACATTTAT

Reads found in SRX4519021

READ ID:SRA:SRR7657393.9970018.1

Query 143 CTGCCGGCTCAATGCCTCCGCCTCAATATGAAGCGTATGCGCCCTGTCAGCAGGAGCAGC 202

||||||||||||||||||||||||||||||||||||||||||||||||||||||||||||

Sbjct 1 CTGCCGGCTCAATGCCTCCGCCTCAATATGAAGCGTATGCGCCCTGTCAGCAGGAGCAGC 60

Query 203 AGGCTCCTGTAGCGCAGGCACCTTCTTATGGCATGTGTAAC 243

|||||||||||||||||||||||||||||||||||||||||

Sbjct 61 AGGCTCCTGTAGCGCAGGCACCTTCTTATGGCATGTGTAAC 101

##### >bFTZF1_putative_Ctenocephalides

ATGTTATTAGACATGGAGCATCACCATTCGGCACTCATGTCTTTGAACATGTCCCCGTTTAGTCTGAGTCCTACTGACAGTCCGGGGGCTGCTCCTCAACAGCATTCCGGTGCCGCCAATGACAATAATAATTCGGGTGCCTCTGCCGGCTCAATGCCTCCGCCTCAATATGAAGCGTATGCGCCCTGTCAGCAGGAGCAGCAGGCTCCTGTAGCGCAGGCACCTTCTTATGGCATGTGTAACATGGATGTTTCCTACTTGTTTTCTTCAGGAGCTGCTGGTTTCCCGATGGAGGGCGCGGGTTCTGGTAGCGGCCCGCCTGGATACATGGGCCTGCTGCCTGGAACGCCGGCCACCACTGCGCAATGCGATCAGACGGAAATGAAAGACGTCATCGAGGAGTTATGTCCCGTATGCGGTGACAAAGTATCGGGATACCATTACGGTCTCCTAACATGTGAATCCTGTAAAGGGTTCTTCAAGCGAACTGTACAAAACAAAAAGGTCTACACGTGCGTTGCAGAACGCAGTTGTCATATCGACAAAACGCAAAGGAAAAGGTGTCCGTTCTGTAGGTTCCAGAAATGCCTTGAAGTCGGCATGAAACTTGAAGCCGTCCGAGCAGATCGTATGCGAGGTGGTCGAAATAAATTTGGGCCAATGTACAAACGAGATAGGGCCCGAAAACTACAGATAATGAGGCAACGACAGCTTGCGATACAAGCGATCAGAGGATCATTAGGAGGCCCAGGAGATGTTTACGGCCAAGGAAGTACATCACCTTATGCAAACCTGCACATCAAACAAGAAATACAAATACCACAGGTGTCCTCATTAACGTCATCTCCCGATTCATCACCCAGTCCCATCGCGGTCGCCCTCGGTCAGGTCGGGCAGACGACGATAACGGCCGCGCCGAACATCGGCGGTATAGGACCTCCATCCACGACCCACCACCACAATAACAGTGGAGCACCTGCCGGCAGCACGCCTCAACAGCCAACGAGCGCCTCTCAATCCGATTCAAAACACTGGAATGCGGCGAATTCGACGACGCATTCACTCTCACCTAAACAGTACTCATATGACCCACCTGCAGCAGGAACAGAATCCGCCAATTCAACGACGGAACCTCTCCGAGTATCACCTATGATTAGGGAATTTGTACAATCGGTCGATGATCGAGAATGGCAGAATTCACTGTATGGACTTCTACAAAACCAAACTTATAATCAATGTGAAGTTGATTTGTTTGAGCTGATGTGCAAGGTGTTAGACCAGAATTTATTCTCGCAAGTCGATTGGGCGAGGAATACGGTGTTCTTTAAAGATCTCAAGGTGGACGATCAGATGAAACTGCTGCAACACTCGTGGTCCGATATGTTAGTCCTGGACCACATGCACCAGCGCATGCACAACTCCCTGCCGGACGAGATGTCACTGCCTAATGGACAGAAGTTCGATCTTCTCTGCCTTGGGCTACTGGGCGTTCCGGCACTGGCAGCGCCCTTCAACGAATTACAAGCCAAACTGCATGAACTTAAATTTGACGTCGGAGATTACATTTGCGTCAAATTCCTGCTTCTCCTCAATCCTGATGTCCGCGGTATAACGAACAGGAAGCATGTGGTGGAAGGTTACGAGACTGTTCAAGCAGCCCTACTAGACTACACATTGACATGTTATCCATCTGTAACGGATAAATTCACAAAATTGGTTTCGATGGTACCTGAAATTCATGCGATGGCCACCATGGGCGAGGATCACCTGTACCTGAAACATTGCGCTGGCAGCGCGCCCACGCAGACGCTGCTCATGGAAATGTTACATGCGAAACGAAAATGAGGCGTTTCTCATCTGACATCCGACTCTGTCGGACATGGACAAGTTCTGCAGGAGTGATTGTCAACCCAATTCGCCCATTAAGCGCCATTCGATGGGCGTCCTTAAAAGCGGATTGAGGTATTTGGCTAAACTGCATAAATGTAAATAAATTTACTCGAATCCCGATACAGAAGAGCCCCAGTGTTGTGTTCAAGAAAACAGAAAATCTAACTAAACCGTAATTATGATATGACAAATTAATGTTATGTGTAAAGAATAAAAATAAATCGCGGGCGCGATCAGGACCACGCGGTCGGTATATTATGTTGTGTGAACTAAAGGACAGTTTAAAGACATTTTCGGACTAGTTTCTTTAATTAGTAGGTTCATTAATTTATAGGTTTCCAGTGTATGTTAATATATAATAGATAACACTAGATTAAGTAAGGAGGCACTTATTTTTTTATTAACAATGGTTACCAAACTGCTCAGTGTAAAACACCTTAAAACAGCGTCGAACAAAAATTAACTTATGTCTGCCTAATTCCTATCTGTTAATTTATTGTTACAATGCGCTGGAACAAGAGATATATAATGTTACCAAGAGTGTGTATAGTCCACGTTGTTGTACGTGACATTTAT

### Lepidoptera (Bombyx mori)


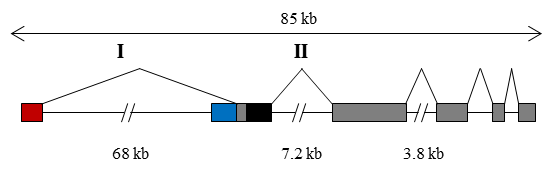


Read found in SRX7508645 and **SRX7508644** embryo

READ ID:**SRA:SRR10836807.9883031.2**

READ ID:**SRA:SRR10836808.11414170.1**

Query 36 GCGCAAAGTCTGGCCGCCTCCACGAGCCAGCCAAAAGGTGATATCGTTACAGAAATTCCC 95

||||||||||||||||||||||||||||||||||||||||||||||||||||||||||||

Sbjct 150 GCGCAAAGTCTGGCCGCCTCCACGAGCCAGCCAAAAGGTGATATCGTTACAGAAATTCCC 91

Query 96 CTGGAATTCGCCATGAGCTCTATGGAGACAAAATCTATCGAAACAACCAACGTGGAGTTG 155

||||||||||||||||||||||||||||||||||||||||||||||||||||||||||||

Sbjct 90 CTGGAATTCGCCATGAGCTCTATGGAGACAAAATCTATCGAAACAACCAACGTGGAGTTG 31

Query 156 AAAATTACCTACGTAGATCCTACAACTGGC 185

||||||||||||||||||||||||||||||

Sbjct 30 AAAATTACCTACGTAGATCCTACAACTGGC 1

##### >aFTZF1_bombyx­_AF426830.1

CTATAATGCACGAAGACGCTCCAAAAATGAGTATAGCGCAAAGTCTGGCCGCCTCCACGAGCCAGCCAAAAGGTGATATCGTTACAGAAATTCCCCTGGAATTCGCCATGAGCTCTATGGAGACAAAATCTATCGAAACAACCAACGTGGAGTTGAAAATTACCTACGTAGATCCTACAACTGGCACTGGAGGTGAACCAGGAGCGTATCTGCCGACAGCAGGAACAGTTTGCGACCAAACTGATACCAAGGATGTAATAGAAGAATTGTGTCCCGTCTGTGGAGACAAAGTCAGCGGCTACCACTATGGATTGCTGACGTGCGAATCCTGCAAAGGTTTCTTCAAAAGAACCGTTCAGAACAAGAAGGTTTATACATGCGTCGCCGAAAGAGCCTGCCACATAGACAAAACTCAACGGAAACGCTGTCCCTTTTGCCGTTTCCAGAAATGTCTTGATGTGGGCATGAAACTTGAAGCGGTTCGAGCAGATCGTATGCGCGGTGGTCGCAATAAATTTGGCCCTATGTACAAACGAGACCGTGCCCGTAAACTACAAATGATGCGTCAGCGACAAATCGCCGTTCAGACTCTGCGCGGTTCTCTAGGGGACGGTGGATTAGTCCTTGGTTTTGGTTCTCCGTACACAGCTGTATCCGTTAAACAAGAGATACAGATTCCGCAAGTATCATCATTGACGTCCTCGCCTGAGTCGTCGCCGGGACCAGCGCTCCTTGGGGCTCAGCCACAGCCGCCGCAGCCACCTCCACCACCAACTCACGACAAGTGGGAAGCCCACTCACCACACTCGGCGTCGCCGGATGCTTTCACGTTCGATACACAATCGAACACCGCCGCTACACCATCCAGCACAGCCGAAGCTACTAGCACTGAAACTTTACGAGTTTCTCCAATGATCAGAGAATTCGTACAAACCGTCGATGACCGCGAGTGGCAGAATGCACTGTTCGGACTCTTACAAAGCCAAACATATAACCAGTGCGAAGTAGATCTCTTCGAGTTAATGTGCAAAGTGCTGGACCAAAATTTATTCTCTCAAGTGGATTGGGCAAGAAACACAGTGTTCTTTAAGTATTTAAAGGTTGATGACCAAATGAAACTTCTACAGGACTCATGGTCTGTTATGCTGGTTTTGGATCATTTACACCAGAGAATGCACAATGGTCTGCCAGACGAGACCACACTCCACAACGGGCAGAAGTTTGACCTGCTCTGTTTGGGGCTACTTGGAGTTCCTTCATTAGCCGACCACTTCAATGAATTACAGAATAAACTAGCAGAATTGAAATTCGACGTTCCAGATTACATATGCGTTAAATTCATGCTTCTTCTCAATCCCGAGGTTAGGGGTATCGTAAACGTGAAGTGCGTTCGTGAAGGTTACCAAACAGTACAAGCCGCCCTTCTTGACTACACTCTTACCTGCTATCCAACGATACAGGATAAGTTTGGAAAACTTGTAATGGTAGTGCCAGAGATACACGCTTTAGCGGCTCGGGGAGAAGAGCACCTGTACCAGCGGCATTGTGCAGGCCAGGCACCTACCCAGACTCTTCTCATGGAAATGCTGCACGCAAAACGCAAATCTTGAAGTACCAGTGACGAATAAAGTTGAAGAGCTGAGAAGCGCCAAGCGCAGACGACACCACAACAAATAGAACATAATTAATTATTACTTATTAATTAGTTTTAGTTACTAGCTTGGTGTGTGATTAAATTAAATAAGTTATTGAGCCGAGCGCCGGCCGGCGTTGCCGCGCGTGCCTAGTCCCAATACAGAT

Reads found in SRX7570839 wing disc

Reads found in SRX7570837 epidermis

Query 292 CGGTTGAAAGCCGCTCAGGATTTACAATAAAAACGGCCGCATCGTGCGGCTGAGCATGAC 351

||||||||||||||||||||||||||||||||||||||||||||||||||||||||||||

Sbjct 1 CGGTTGAAAGCCGCTCAGGATTTACAATAAAAACGGCCGCATCGTGCGGCTGAGCATGAC 60

Query 352 GATGGACCAGCAAACAAGCCTCATGTCCCTTAATATGTCCCCATTTGATTTAAGTCCTGG 411

||||||||||||||||||||||||||||||||||||||||||||||||||||||||||||

Sbjct 61 GATGGACCAGCAAACAAGCCTCATGTCCCTTAATATGTCCCCATTTGATTTAAGTCCTGG 120

Query 412 TCCAGAAGGATCGGCTTCGGGTGGTGGACC 441

||||||||||||||||||||||||||||||

Sbjct 121 TCCAGAAGGATCGGCTTCGGGTGGTGGACC 150

##### >bFTZF1_bombyx_AB649122.1

AGTCGATCGTGGTACGTCTACTGTGAGGTACGCGTGCTACCGTCAGTCAAATCTGCTCGTCGCGATCGTAACTAACCCCGCGCACAGTTGAAGACTTGTGCGATAGGTCGTTATAGTTCCAATAGTGACTGATATTAGCGCGAAGCGCTATTATTTTCACACAATTGTTATAGGACAAAATGTGCTGGATTTCCGTGCCTACTTTAGTGCTTGAACAGGTGCAGCTCGCAAGCGGCAGCTAGTGTTGCGTCCAGACCGCTAGTGAACTCGGTGACTGTGTGAACGTTTTTGCGGTTGAAAGCCGCTCAGGATTTACAATAAAAACGGCCGCATCGTGCGGCTGAGCATGACGATGGACCAGCAAACAAGCCTCATGTCCCTTAATATGTCCCCATTTGATTTAAGTCCTGGTCCAGAAGGATCGGCTTCGGGTGGTGGACCTTCCAGTGCTTCCCAACAATATGTGCCTCAAGGCGCAGCATACCAATGCCCCCCTGAACAACAATCATTTGGATATGCCAATCTGGATGCTTCATATCTATTTCCGACAGGCACTGGAGGTGAACCAGGAGCGTATCTGCCGACAGCAGGAACAGTTTGCGACCAAACTGATACCAAGGATGTAATAGAAGAATTGTGTCCCGTCTGTGGAGACAAAGTCAGCGGCTACCACTATGGATTGCTGACGTGCGAATCCTGCAAAGGTTTCTTCAAAAGAACCGTTCAGAACAAGAAGGTTTATACATGCGTCGCCGAAAGAGCCTGCCACATAGACAAAACTCAACGGAAACGCTGTCCCTTTTGCCGTTTCCAGAAATGTCTTGATGTGGGCATGAAACTTGAAGCGGTTCGAGCAGATCGTATGCGCGGTGGTCGCAATAAATTTGGCCCTATGTACAAACGAGACCGTGCCCGTAAACTACAAATGATGCGTCAGCGACAAATCGCCGTTCAGACTCTGCGCGGTTCTCTAGGGGACGGTGGATTAGTCCTTGGTTTTGGTTCTCCGTACACAGCTGTATCCGTTAAACAAGAGATACAGATTCCGCAAGTATCATCATTGACGTCCTCGCCTGAGTCGTCGCCGGGACCAGCGCTCCTTGGGGCTCAGCCACAGCCGCCGCAGCCACCTCCACCACCAACTCACGACAAGTGGGAAGCCCACTCACCACACTCGGCGTCGCCGGATGCTTTCACGTTCGATACACAATCGAACACCGCCGCTACACCATCCAGCACAGCCGAAGCTACTAGCACTGAAACTTTACGAGTTTCTCCAATGATCAGAGAATTCGTACAAACCGTCGATGACCGCGAGTGGCAGAATGCACTGTTCGGACTCTTACAAAGCCAAACATATAACCAGTGCGAAGTAGATCTCTTCGAGTTAATGTGCAAAGTGCTGGACCAAAATTTATTCTCTCAAGTGGATTGGGCAAGAAACACAGTGTTCTTTAAGTATTTAAAGGTTGATGACCAAATGAAACTTCTACAGGACTCATGGTCTGTTATGCTGGTTTTGGATCATTTACACCAGAGAATGCACAATGGTCTGCCAGACGAGACCACACTCCACAACGGGCAGAAGTTTGACCTGCTCTGTTTGGGGCTACTTGGAGTTCCTTCATTAGCCGACCACTTCAATGAATTACAGAATAAACTAGCAGAATTGAAATTCGACGTTCCAGATTACATATGCGTTAAATTCATGCTTCTTCTCAATCCCGAGGTTAGGGGTATCGTAAACGTGAAGTGCGTTCGTGAAGGTTACCAAACAGTACAAGCCGCCCTTCTTGACTACACTCTTACCTGCTATCCAACGATACAGGATAAGTTTGGAAAACTTGTAATGGTAGTGCCAGAGATACACGCTTTAGCGGCTCGGGGAGAAGAGCACCTGTACCAGCGGCATTGTGCAGGCCAGGCACCTACCCAGACTCTTCTCATGGAAATGCTGCACGCAAAACGCAAATCTTGAAGTACCAGTGACGAATAAAGTTGAAGAGCTGAGAAGCGCCAAGCGCAGACGACACCACAACAAATAGAACATAATTAATTATTACTTATTAATTAGTTTTAGTTACTAGCTTGGTGTGTGATTAAATTAAATAAGTTATTGAGCCGAGCGCCGGCCGGCGTTGCCGCGCGTGCCTAGTCCCAATACAGAT

### Lepidoptera (Manduca sexta)


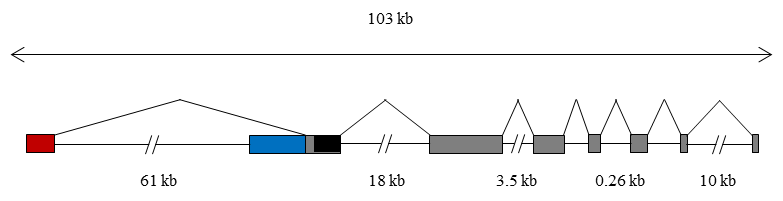


Reads found in SRX7724678

Read ID: : SRA:SRR11085580.10192016.1

Query 169 CGGCAACGGTGATATATTACATAATCTGTAGGCGGCATAATACGGGCGCCTCGTCTCAAT 228

||||||||||||||||||||||||||||||||||||||||||||||||||||||||||||

Sbjct 125 CGGCAACGGTGATATATTACATAATCTGTAGGCGGCATAATACGGGCGCCTCGTCTCAAT 66

Query 229 ATTCGTGTATGATGCACGAAGACGCTCCAAAAATGAGTGTAGCACAAAGTCTGGCGGCCT 288

||||||||||||||||||||||||||||||||||||||||||||||||||||||||||||

Sbjct 65 ATTCGTGTATGATGCACGAAGACGCTCCAAAAATGAGTGTAGCACAAAGTCTGGCGGCCT 6

Query 289 CCACG 293

|||||

Sbjct 5 CCACG 1

##### >XM_030168105.1

TACCGACTTATTTCAATGTCAGTCATGAGTACGGTAGCTTCATTGAACCCGAGTTATGTCGGTCGGAATATCAAAGTGTAAGTGAAGTGGCCTAGAGACCTACATTTTGTTGTTTAAACGCATAATCGCATTTAATGGGTGATTTTTGAAATAGTATATTACCCGAAGCGGCAACGGTGATATATTACATAATCTGTAGGCGGCATAATACGGGCGCCTCGTCTCAATATTCGTGTATGATGCACGAAGACGCTCCAAAAATGAGTGTAGCACAAAGTCTGGCGGCCTCCACGAGCCAGGCGAAGAGTGAACTTGAAACTGGGGTTCCTTTGGAATACGACTTGAGTTCTACGGAGCCAAAACCGACAGAAAATATAGATATGGAATTGAAAATTGCTTATGTGGACCCCACTGGCGGCCCTGGTGGTGAGGCCGGAGCCTATCTGCCCGCAGCCGGTGCCGTGTGCGACCAGACTGACACCAAGGACGTGATCGAGGAACTGTGTCCTGTTTGCGGAGACAAAGTCAGCGGCTACCACTACGGCCTGCTCACATGCGAATCATGCAAAGGTTTCTTCAAGAGAACCGTTCAAAACAAGAAAGTTTACACGTGTGTAGCTGAGAGAGCCTGCCACATAGATAAAACACAAAGGAAGCGATGTCCCTTTTGCCGATTCCAAAAGTGCCTCGATGTCGGCATGAAGCTTGAAGCCGTACGAGCCGACCGCATGCGTGGAGGCCGGAATAAGTTTGGTCCCATGTATAAACGCGACCGCGCTCGCAAACTTCAAATGATGCGACAAAGACAAATAGCAGTGCAAACGCTACGCGGCTCTCTCGGTGATGGTGGTCTCGTGCTCGGTTTCGGCTCACCTTACGCAGCCGTGCCCGTGAAGCAAGAAATCCAGATCCCGCAGGTGTCGTCGCTAACGTCGTCGCCGGAGTCATCACCGGGCCCGGCGCTGCTGGGCACGCAGCCGCAGCCGCCGCAGCCACCGCCGCCGCCTGCGCACGACAAGTGGGAGGCGCACTCGCCGCATTCGCCGGACGCGTTTGCGTTCGACGCGCCAACCAACGCAGCAGCTACGCCATCCAGCACCGCTGAACCCACAAGCACGGAAACCCTTCGAGTTTCACCTATGATCCGCGAATTTGTTCAAACTATCGACGATCGCGAGTGGCAAAATTCGCTGTTCGGACTCTTACAGAGCCAAACCTACAATCAATGTGAGGTGGATCTCTTCGAATTAATGTGCAAAGTGCTGGACCAAAACTTGTTCTCTCAAGTGGACTGGGCAAGAAATACAGTGTTCTTTAAGTATCTAAAGGTTGACGATCAGATGAAACTCCTGCAGCACTCGTGGTCCGACATGTTGGTGTTAGATCATCTGCATCAGCGGATGCACAACGGTCTGCCGGATGAAACGACGCTCCATAACGGACAAAAGTTCGACCTGCTTTGTTTAGGCCTCTTGGGCGTACCGGCTCTGGCGGACCACTTCAACGAGCTTCAGAACAAACTGGCCGAGTTGAAGTTTGACGTTCCAGATTATATCTGTGTTAAATTTTTGCTTCTCCTAAATCCTGAGGTGAGAGGCATCGTAAATGTGAAGTGCGTACGAGACGGTTACCAAACAGTGCAGGCTGCGCTTCTAGACTACACTCTAACCTGTTATCCTACAGTTCAGGACAAATTCGGTAAACTGGTGATGGTGGTGCCAGAGATCCACGCGCTGGCGGCGCGGGGGGAGGAGCACCTGTACCAGCGGCACTGCGCCGGCCAGGCGCCCACGCAGACCCTCCTAATGGAGATGCTACACGCTAAACGCAAGCCAAACGGAGGCGAAATGGTTAACCGGAGTGCCGAGCACACGTCAACTCTAGACAGATTGTGCGGTTTCCTGCCAAGTGACACGATTGAGCCGCATTCTCCAATATAGAATCTTGAAAACCCAGTGAAGAATAAAGAGAACACTGCATACGCGCCAAGCGCTCCCGACATCACGTCAGTTAGAATATTATATTTAAATAAAACTTATTAATTAGTTTTAGTTTATAACTTGGTGTGTGATTAAATATAATAAGTTATTGCGCCGAGTGCCGGCCGGCGGTGCCGCGCGTGCCTAGTCCGAATACAGATAAAACAAATATTTATCTATCTAATTTCTGTAAAGTAATGTTATACAAATATTGTACACCAGTCTTGAGTAGCTACGATTAACTTATTTAGCATTCTCATCACGTTATTCAAAATTGAGTGTGGTATCTACTTTTTAAACTAGGTTTTCTGTATCTCCGGTACGTAGGTGAAGAAGAATTACAATACATATTTTAAGAAAGCGTCTGTGATTATAATAAATGGAGAAATTCGATGAAAACCGTTAGATTCATTACGTATAGTATAAAATGATTGCTAGTTTTTGTTTTGTCTAGCAGCATGTTAAGTTCGACGTGTCACGTGGCAGGCCGCAGACTGGGCGCCTGCCCGCTCTTAATTTCGGCGTTACTTACTATACATACGATATACTGTTTATGTTGTTTTTAAGAGTTTGGAGCACCTACGCGGTACGTGTAATGTTTAACCAGTCTTCACAGATATACGAGTTGTGTTCTGTAGCTTACATGTATTTGCAACGTAAAGTGTAAAAAGTATGCGTGTGTAATGCGTATCTAATGCAATAAGTTTGTTAACTAGTGTGTGCGGCAACTTGCAATTGGCGCAGTTTCTATATTTGGCTTGATTGAGATGTTTAAAATTTTTATCAAATGTTGTCATTTGAAAAATAAAATTGTTTAAATATATTATAATAGTAAGGACAAAATATAAATCATGTTTAAAAATCACAGTTATTCACTATAGATATTTAACTGAGTGTTGTTGTAAGAAAATATTCGATTATTATTATTGGCCATACCAGTACAAAATCAAATGATCCTTGTATATAACATAGCACATTTACAGTGATTTATTCTACTTAAATTGTAATTATGTAAAATATTTCTCAACATACGTGCAAGAAGCCTTAAAAATCATAGATTTTTAAAAAATATGGGTACTAGCGTCTACCGGCTAATGGCCGAGGGAGATGATGTATTTTTATATAAAACATATTGTTCTTTATTAACATATATAATATGTTAACTGAAAGTACATAATTTCGTATTTTTCTAACAATTAAATATAATAGTGTTCGTGAGCATGTGTAATAATCTACTTAAAAAGTGGAATTTATTATACATAATAAATATGAAATTAATCTAAAATTATCATTCGTGTGTAGAGTTCTATAAAATGACGTTACAAATTTGATTTGAAAACGTTTTATTTTTGTAATGTTTTGGCGTAAATTGTTTGTGGTCAACAAAGCCCATTTCATAAACGGTAGCCAAGCATGCGCCAGCACTCGCATTACGTACATTGAGACCGAAGTCAGAACATGGCCAGGCACCAGTGTTGTGGAATGGGCGCTCCAAGTACATTATTTGTGACGATTAATCTTAGTTGATTGAAAAATGTGATTGACAGTAGGTATGTGTTTTCAAGTGAATGTTATATTTTATTACTATATAAATATTTTTATCATGTCGTAGCACCAAGTGCGAATGTATCGATAGTAGGCTGCATCAACGAGTAGTAAGTATAGTAATGACGAGGTGATGAGTTATAAATTTTGTTTAGCTTAGTGATTCTCACATAAGGTCTACGTGTACAAAATTATGGCACCGCGTTTATTCTATAATACCGTCCAATCAACCGAGTAATTTTATTGTCATGACATAATGTTTTAGGGATTCGATTAATCTTTGTTGCAATTGTAAATTGATCGGACACGCCGTACGAGGTAATGCTAGCTTGATAGAGTGAGGTTAACTACTAAGTTATATCAAATTCACGAAATAGGCGACAATAGAACGGAATGTAATTACCTGGTCTAATGAAATAATAGTTACATATTATAGTAGCGATCAACTTAAACGAAGTTCCTTAAAATGTTGTTGTGGTACAATTTTTAAAATATACAAATGTATGCGGATTCGTTTAAATCATAGAAATTGAGCAATTTTATAAAATTTTACTTAAATAAGTATTGACGAGTTTACTGAATTACTTTGCAAATGTTTTGTATGTAATTATTATTTACATGTTATATAAAGGACGACGATTCAGCACAACTCTGCATATGTAGATACAGTTTTAACAGTTAGAAACCCCAATCGTGTATGTCTTGCTTGTCAATTTTATATTGTCGAAATAGTAATACCAATTATATCTGAATAAAGTGACGACTTAGTAAGATTTTTTGTAAATTAAGAATATGTCCATCGATATAATAGATATTTATTAAATTTTTATAAATCAGATTAATCGATTAGTGTAAAGGGACTTTGAAGTTTGGTTATTAATAACTTTCTTAATTCACAGGAATCTGTACTAACGTAACGATCGATCACGGCACATTTACTACTAAGTTGATGTTGCTGATTACACTACAAATTTAAACGCTTTATCTGGGCAAAGCTCATCTGTGATGGTATGACATAGTTAGGCTGTGTGGATCGTATATTTTCTCTATTTTAAGGCACATTTTTAGACTGTGAACACACCCTAATAAAATACCACTATCTTAATACTACGCTACTGTTACAAAGTTTTCTTCTACTTAATGTGCCGCGGGTATTTATATAAAATCAGTCTTTCCTAACGGAAGAAACAATTTAATATCACAATTACTATCGAAATAATTTATTGACTTGACAAACACGAACTTATACTTGGCATCGCTAAGCAACAGTTTTATTTAATACAGTATTATTTATATGCTAATATCACATTTATCACACTATAATTCGTATATGAAAATAAATATTTGCATTGCATTAACTTATGTGATAGCAATACGGATGTTATGGTTTTACCCAAAATATCAGACCGATTGACAGTACTTGTTTATAACTGCAAGAAAACGCAAATAGATTTTCGATGATTTCATATTATGTACTACAAATTCACTTGACTCGCTCGACCAATCGACCGCTCTGCCCCTCTAAACCAATCGGCACAGAGTAACTAGCACGAATTTTTGTATTATAGTATATTACGTGAGTTATGGTTTCGTTTAAGTAATTTTTTGTACGGAAATGTATTTTGTACGGCACCCCGGTGAGCGTATTTGCAACTAAAATACGACAGCGTGAACATTGATCGCGTCTAAACTTGGAATAGTAAATCGTGTAACCAAGGTAGTAGTAAATTCATAACCTATGGGAACACTCAGCACGAATCTCTTTGCCCAAAGACTATAAAAATACTTAAACGTTAACTTACTCGATACTTGGAGCAATTCGTGCTCAGACGCTGTCACCACGCGGCACCAATTAGATTTTACTGTGACATCGATAATCAATAAGCGCAAGACTATTCGCTATTCTATCACGGTGTGAGATACATACAGTCTGTCTTCATTGAAGTCTAGTTCGGTGCTCTGTGCTATGCAGTATGCTGTAGGCGATATGGGGGATCAAGACTTTCAATATAATGCTTTACAAATATGTTGATGGTTCTCAATGGTTTTGCTTCCAAAATTGAAGTCTCGGCTATAGAGGCCACACAGTATCTAGCAACTACCAGTACAATTTTATTTTTATACATTACTACGAATGCGCCAGACAGGTTCAAATTAAAAAAAAAATGTTACTCAATGGAAAGTTCTATAATCAGTAGGTAGTTAGTAAACAATTATAAATTACATTTATAGGTTTTTATAAACCTTGTCACAAAGAATAATCCTTAATTTGATACTTTAAATACATTCATATTTTATATACTTACCTCATTTTTTATAATAAATAAATATTAACCTTTAAAAGTTATAGAATCTTATGAGTTTTCGTTATTTATGACGTAACCTAAGCAATTTTTTGATTACATATGTTAAAAATATATATTTGTTTCAAATCGTAAACTTCAAATATTTCTATCATTTTTTAAATGACCCTGAATCGCCTATGACTACTGTATGGTATAAAAAATGATTGGTATTTACGCGAGTCTTTTAGTTTAGCGATACATTTACAAGCTGCTGATAAAAAATAGTACGAACATGTCTAAGAATTAGTCAAGTAATTACTACCGCAACATATCTAAGAACTATTTTGAAAAAATAAGTCATAAATTTATTCATCACGTGGGTAACTCGCGCACTTCTGACATAATTATCAGCTGAAAATTCTTGTTGTTATAAGCAACACCAGAAATATTCACAGTATTTATTTTCCTATATTTTTGGTACTGGGAAATAACCTGAGTTCAACGTATACCTTATTTACGCATGTAACGTAACTTAAGAGCTCGGATGTGTACTGCACCATCAGCTTAAACGACATATATTGTGATATATGATATTTTATACCACGGTCGAGCAATATCACTGTTTAACAATCTGCGTAGGATTAAAATGACTCAGAATATTTATTATTAAATAGTTAGATTTTCATGTACGTCTAATTCGATTAAACTCAATGCGTATATATATATAATTATTCTTGCATTCATTTCAATTTAGTAATAGAATACTAAAAATAACCCCTTGATGTGAATACGTACACTGGTATCAGTATTTCCCGACTATAAAACACGTTGTTTACTATGTGATTGTTAAATTATGGTACTATTGCTCGTTTGCAAGATATATTTGGGTTACTTATGTTTTGGGTAAATCTGTACTTGAGTGTGAGAGAGAACGATATCTTTTACGCCTAATATGCTTATATATTTTAGATACATACAATGGTGAATAACTTGCAGGTGTGACTTTTGTATTTATTAGGTAACACAAGTAGAGCGTTATAATATTATCTACTTTACTTTCATAAACAAAACGCTTGTAGTCACAGTTCATTGTTCATAATAATTTATTTATATTTATGTAAGTATATAATTTACTTTGATGTCTATGTCAACTGAGGGATCATTTTCCAATCATCAGATAAAATATATTCATAGGTTAAGTACAACACAAAGGAATTAAAAAATGAAAGTTGTATTATAAAATTGTTAATTTGTCTCGCGTTGTATACAGTGCAGTTATATTTTATTCGATGGATAAGTTTTATATGACGATTGAAAAATTGGCCCTAAGTAAGGTAACGTTTAGCAAGTATATCTATATTTTCAATCATTATGACACATTAGAAAGTCAGTGAATATGAACAACCACTACTTATGCAATTTAAGTATCTTTATTATAGACCTTAATAATACGACGTACAATCGCAATGGCTTGTGATCGTAAAAGCAATAGATACAGCAATTAATTTGTCCCGTATTTTAAAATTTAATACCTGCTTAGCTTGCTAAGTTTACCAATTAGCTTAAATTCGTTATTTAGTGTCTTTTCATAAGTCTTTATATTATCTCGACTACTGTAATCACTGTTACTTTGTATTTAGCATTATAATTTGCAATCCTTCAGCAGGGGTTTTGCAAAATTCGCAACATGCATTTCATGAACTAATATAATATTTACCAGTTTTATAAATCCTTATTTGGTTAAATATGAACTCATTTAATTTGGCAAGGGCACAAAGTCCATCCCGTAACTGCTTACGATGTAGTTTCGTGTGGGTGTGGTATACATCATGTACATTTGTCGCGGTCGATATTACACCGTAATTTAGACAATTATTTAATTATATTATTCAATTTAAGGTGCCAGTGTTCATAATTATTAATTTCAGTTAGTCGAATAAATTGCTTTCATAATCTTAATCATTTGCTTCGTTTTCCATGAATCAACTATACCTTTCATTTTAATCTTTTATTCAATATATAAAATTTAAACCTACTATTTACTTTTAAGAGTAGATTGTAATGTACATGATAAAGTTGTATGTTAGGGCTTATGTAAGGAATTTCTATATTTATGAATTAAAACGATAAGTATTTTTAAATGTAAGACACATCATGAGTGTTCTATGTAAATGTACATTTTCAGTTCAATTAGGTTAAGTGTTAGGTAGTTTTACGAACTGCGCCGAGTCGAGCGATGCCTTCGTGTTGCCGTGTCGCACTTGCGACTCGGAGCATATTGTCCTGCCGTTCGGGCACTGTTACTTCGCTCAGATTCGCTCGCAGTGCAACGTAAATACATCGCCGTCGTAACATGTCATGGCAGTTACAGAGGGCCATATTTCAGACGCGGATAAGCACGTATTTCGACACTCTTATAATAGCGCTACGCTTGCATGGGTGAGGGGCGGGGTGCCTAGATGAGTCGATGCATTGTTCGCGTTTCGCGTTTAAATGTTACCCGTTGTCATAGGTAGGAAATTGAGGATTAAAATGATATTTTATAAAGCCAATTTTAAAAACTTTTAATAATTATCTGTTATGCAAATCTTTTATATACTCCACAGCAATCGTAATAAATGTATATACTTAAATCGAATATGATATGCGCCAAATTAGCCCATGCCTGTTTATAAAAATATCACAATATTTCTCATTGAGTATCTTTACATGTTTGTCGCTTATTTAAGACAAAGGGCTACAGTCGCTACATGCATGAATTTGCACAGTGTGTGTGATCATGGGGGCGTGCAAAAACGAGCTTGTCGTGTCACCATTAGTTCAGGTCTGTAAACGCTGCAAGGGACGTGAGCTCGCTTCTGGTAAACTATTCTTCTATCCCAGTATGGCTGGCTGCTGACAGTTGCCCGTGCCCGCGTGATCCATGCTCATAGCTCACCTGGTCGCACGGTCGCGGCCACCGGGGTGAAAGAGTACGTACGCGTCGCACACATTCCAAAATAACACTAAAAGTAACGCTATGTGACGCGCGTCGACGAAAGCGTTGCAATTTGTAAGTTTTAACCACTTTGTTGTTCATTAACGATAAGTTAGGTTTAATCTCTGTCGATGTTGTTAAATTTAGTCTGTGTATAGTATTCTTTTGGTTAAGTTTATGTGAGGCGTGAATTTGAGCGAATCGACAGGCCTAAGCCTGATGGGTTCACACTAGCTGGTAATAAGTAACCGAGCGATAATTTCCGTTGCATCTAACTTTATTTCGCGAGCTGTGTTAATTTTCTTTTAATTGTCAATTGTACATATAGGTAAGAAGACGATAAACAGAGTTTTCATTCAGTCTTAAATGGTTGTTGTTGAATATTAAATGATGGGGCTGGCAAATACCAATGCAATTTAGCATTTGATGTTGATAATTTGAATAAAGCTTTTGTCTGATATGGTAAATGTATGGCTAATTAGTTCTTACTATTTCGTAAACAGTGATAAGGTGAAGACCCTGAACTCGGCTATTTTTGTAGCTGTTTTAAATTACTGTATCAACGTTCAGACACATAATTTATTTACAAGTAAAATTAAGGCAGTCCACACAAACCCATCTCCGATACGTGTCTAATTTGGGGTTCCATACTCCTTAATATACACACCGCGCGCGGAACACATCCCTCGCATCTCTCGGTTTAGTATATTTTTTGTAATATAATAAGAGTAAAATACACCTTAAGTTCAGCCACAAAGGCTTGTGAGGTTGCTACCGTTAAAACTGAACGCGATACATTTTAACATCATTATTTTTAAATAAATACGCTACATCACTGTTCACATCACTAATTTAATATGTGAAACAAACACATTTTTATGATTTTAATTATTATAAATTTTACAATATAATATTATCAATGAATAAAAAAAAATCGTTAGATTGATTATCTTTACACGATATATTTTAGACATCAGTTTAAACGGAGCAACTGACGTTATCAACCACAGCCACATTTGTACCATGTCATTACCTCACATTACGTCTTTTATACATAATAAATAATAGTTATTGTTCTAAATCTTTAAACTTATTATTATTGTATTGTTTAGAGGTGTCATTAAGTTTAAATTTGTAAACACGATGGTCTATTTTTAGATTACAAACACAGATACATGTATGACGGTGTAAGGTCGCAAGTACACCGTCGATGTATTTATTTTCTTGGTGATCAATGAATATTGTGAGACGCACGTACACGCCGGCGAGCGGCGGTGAGAGCGCACGCTCGCTCCGTCGCCCGTTATTGTAGAAATAAATGATATTTTCGTTTAAATAA

Reads found in SRX702733

Read ID:  SRA:SRR1577022.21365140.2

Query 256 GCGGCCGAGCATGACGATGGACCAGCAGACAAACCTCATGTCCCTCAACATGTCCCCGTT 315

||||||||||||||||||||||||||||||||||||||||||||||||||||||||||||

Sbjct 1 GCGGCCGAGCATGACGATGGACCAGCAGACAAACCTCATGTCCCTCAACATGTCCCCGTT 60

Query 316 CGATCTGAGTCCCGGTCCCGAAGGATCGGGATCGGGCGGA 355

||||||||||||||||||||||||||||||||||||||||

Sbjct 61 CGATCTGAGTCCCGGTCCCGAAGGATCGGGATCGGGCGGA 100

##### >AF288089.1

GGACGGTTCTCTGTGGTTGGTGGTCGTAACTATCGACGGTGTCTGACATCGGCGCTTTATGCCCGTATGATTTTCACAACTTAATCATAGAACAAAATGTGCTGGATTTACGTGCCTCCTACAATAGTGCTTGAACAGGTGCAGCTCGCGAGCGGCAGCTAGTGTTGCGGTGAGACCGCTAGTGAACTCGGTGACTGTGTGAACGTTGGGGCGGTCAGTAACCGCTAGGGATTTATAATAAATTTGCCGCTCGGTGCGGCCGAGCATGACGATGGACCAGCAGACAAACCTCATGTCCCTCAACATGTCCCCGTTCGATCTGAGTCCCGGTCCCGAAGGATCGGGATCGGGCGGAGGTCCCTCTGGAGCCTCGCAACAATACGTGCCACAAGGCGCCGCGTATCAATGTCCACCCGAACAGCAATCCTTTGGCTATGCCAACCTGGACGCCTCATATTTATTTCCAACAGGCCCTGGTGGTGAGGCCGGAGCCTATCTGCCCGCAGCCGGTGCCGTGTGCGACCAGACTGACACCAAGGACGTGATCGAGGAACTGTGTCCTGTTTGCGGAGACAAAGTCAGCGGCTACCACTACGGCCTGCTCACATGCGAATCATGCAAAGGTTTCTTCAAGAGAACCGTTCAAAACAAGAAAGTTTACACGTGTGTAGCTGAGAGAGCCTGCCACATAGATAAAACACAAAGGAAGCGATGTCCCTTTTGCCGATTCCAAAAGTGCCTCGATGTCGGCATGAAGCTTGAAGCCGTACGAGCCGACCGCATGCGTGGAGGCCGGAATAAGTTTGGTCCCATGTATAAACGCGACCGCGCTCGCAAACTTCAAATGATGCGACAAAGACAAATAGCAGTGCAAACGCTACGCGGCTCTCTCGGTGATGGTGGTCTCGTGCTCGGTTTCGGCTCACCTTACGCAGCCGTGCCCGTGAAGCAAGAAATCCAGATCCCGCAGGTGTCGTCGCTAACGTCGTCGCCGGAGTCATCACCGGGCCCGGCGCTGCTGGGCACGCAGCCGCAGCCGCCGCAGCCACCGCCGCCGCCTGCGCACGACAAGTGGGAGGCGCACTCGCCGCATTCGCCGGACGCGTTTGCGTTCGACGCGCCAACCAACGCAGCAGCTACGCCATCCAGCACCGCTGAACCCACAAGCACGGAAACCCTTCGAGTTTCACCTATGATCCGCGAATTTGTTCAAACTATCGACGATCGCGAGTGGCAAAATTCGCTGTTCGGACTCTTACAGAGCCAAACCTACAATCAATGTGAGGTGGATCTCTTCGAATTAATGTGCAAAGTGCTGGACCAAAACTTGTTCTCTCAAGTGGACTGGGCAAGAAATACAGTGTTCTTTAAGTATCTAAAGGTTGACGATCAGATGAAACTCCTGCAGCACTCGTGGTCCGACATGTTGGTGTTAGATCATCTGCATCAGCGGATGCACAACGGTCTGCCGGATGAAACGACGCTCCATAACGGACAAAAGTTCGACCTGCTTTGTTTAGGCCTCTTGGGCGTACCGGCTCTGGCGGACCACTTCAACGAGCTTCAGAACAAACTGGCCGAGTTGAAGTTTGACGTTCCAGATTATATCTGTGTTAAATTTTTGCTTCTCCTAAATCCTGAGGTGAGAGGCATCGTAAATGTGAAGTGCGTACGAGACGGTTACCAAACAGTGCAGGCTGCGCTTCTAGACTACACTCTAACCTGTTATCCTACAGTTCAGGACAAATTCGGTAAACTGGTGATGGTGGTGCCAGAGATCCACGCGCTGGCGGCGCGGGGGGAGGAGCACCTGTACCAGCGGCACTGCGCCGGCCAGGCGCCCACGCAGACCCTTCTAATGGAGATGCTACACGCTAAACGCAAGCCAAACGGAGGCGAAATGGTTAACCGGAGTGCCGAGCACACGTCAACTCTAACAGATTATCTTGAAACCCATGAAAATAAAGAAACACTGATACCGCCAGCGCTCCGCATACGTAGTAGATATTATTAATAACTATAATAGTTTAGTATACTGGGGGATAAATATAGTATGC

### Lepidoptera (Spodoptera litura)


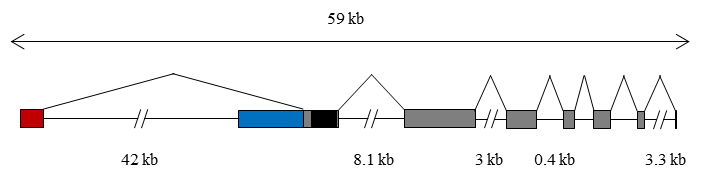


Read found in SRX5172374

Read ID: SRA:SRR8361847.9356792.1

Query 138 AATTATTTGTGTATAATGCACGAAGACGCTCCAACAATGAGTGTAGCACAAAGTCTACCC 197

|||||||||||||||||||||||||||||||||| |||||||||||||||||||||||||

Sbjct 1 AATTATTTGTGTATAATGCACGAAGACGCTCCAAAAATGAGTGTAGCACAAAGTCTACCC 60

Query 198 GCCTCCACGAGTCAGGCGAAGAGTGAGATCGTGACTGAGGTcccccccGTGGACTATGAA 257

||||||||||||||||||||||||||||||||||||||||||||||||||||||||||||

Sbjct 61 GCCTCCACGAGTCAGGCGAAGAGTGAGATCGTGACTGAGGTCCCCCCCGTGGACTATGAA 120

Query 258 TTGAACACTGTTGAACTAAAACCCTCTCAA 287

||||||||||||||||||||||||||||||

Sbjct 121 TTGAACACTGTTGAACTAAAACCCTCTCAA 150

##### >HQ260326.1

CGCGGGGAGTTATGTCGGTCGGAATATCAAAGTGTAAGTGAAGTGAAGCTATCCTTATACGGCGTAGGTGAAATACTTAAAGTTCTCTGGGCGACATTATCCTGTCGCCCTGTCCAATTATTTGTGTATCCCTGTCCAATTATTTGTGTATAATGCACGAAGACGCTCCAACAATGAGTGTAGCACAAAGTCTACCCGCCTCCACGAGTCAGGCGAAGAGTGAGATCGTGACTGAGGTCCCCCCCGTGGACTATGAATTGAACACTGTTGAACTAAAACCCTCTCAAACTATTGAAATGGAGTTAAAGCTTGCTTATGTGAACCCCTCCAGCGGCGCTGGTGGCGAACCAGGCGCTTATTTGCCAGCAGCCGGCACAGTATGCGACCAAACCGACACTAAAGATGTGATCGAAGAACTTTGTCCCGTTTGTGGAGACAAAGTCAGCGGTTACCACTACGGGCTGCTAACGTGCGAGTCTTGTAAGGGATTCTTCAAGAGGACCGTACAAAATAAGAAGGTGTACACGTGTGTCGCTGAGCGAGCCTGCCACATAGACAAAACACAGCGGAAGCGCTGCCCATTTTGCCGCTTCCAAAAGTGCCTCGACGTTGGCATGAAACTCGAAGCTGTTCGAGCTGATCGCATGCGAGGAGGTCGTAACAAGTTCGGTCCTATGTACAAAAGAGATCGTGCCCGCAAACTGCAAATGATGCGACAGAGGCAAATTGCTGTGCAGACTCTACGTGGATCTTTGGGAGACAGTGGTCTAGTGCTTGGCTTTGCCTCGCCTTACGCAGCGGTTCCAGTCAAGCAAGAGATACAGATCCCACAAGTATCATCTCTCACATCATCGCCAGAGTCTTCACCAGGCCCAGCTTTGCTGGGTGCGCAGCCGCAGCCGCCCCAGCCTCCGCCGCCACCCGCTCACGACAAGTGGGAAGCGCATTCGCCTCACTCCGCCTCCCCAGGCGCATTCGCGTTTGACGCACCAGCCACAGCAGCCGCAACGCCGTCGAGCACCGCCGAACCCACAAGCACTGAATCCCTACGAGTCTCACCCATGATACGCGAATTCGTACAAACTATTGATGACCGCGAGTGGCAGAATTCGCTATTTGGGCTCTTGCAGAGTCAGACTTACAATCAGTGTGAGGTCGATCTCTTCGAGTTAATGTGCAAAGTATTGGACCAAAACTTATTTTCACAAGTGGATTGGGCGAGAAACACCGTGTTCTTTAAGTATTTAAAGGTCGACGATCAAATGAAGCTGTTGCAGCACTCGTGGTCTGACATGTTGGTGTTAGATCACCTTCACCAGCGAATGCACAATGGCTTGCCTGATGAGACTACACTTCACAATGGCCAAAAGTTTGACCTCCTCTGCCTGGGTCTCCTGGGTGTACCATCGTTGGCTGACCACTTCAACGAGCTCCAGAACAAATTAGCAGAGCTGAAATTTGATGTCCCAGACTACATTTGCGTTAAATTTTTACTTCTATTAAACCCTGAAGTAAGAGGCATCGTTAATGTGAAGTGCGTCCGAGACGGTTACCAAACAGCCCTCCTTGACTATACGCTGTCCTGTTATCCAACGATACAGGACAAATTCGGGAAACTAGTAATGGTAGTTCCTGAAATACATGCGCTAGCCGCACGTGGCGAGGAGCACCTGTACCAGCGACACTGCGCTGGACAGGCACCTACACAGACTCTCCTCATGGAAATGCTGCACGCGAAGCGCAAGCCGAACGGAGGTGAAATGGTTAACCGGAATGCCGAGCACACGTCGACACTTGACCGATTATCTTAGTTTCAGTGAAGAGAAACAGACCACAAAAGCGCAAAGCGCTCCCGACACCGCATCAGAATAGAATATGCAACAAACTTTACTTATTAGTTAGTTTTAGTTTATAACTTGGTGTGTGATTTTCAAATTTAAGTTATTGCGCCGAGCGCCGGCCGGTGGTGCCGCGCGTGCCTAGTCCGATACAGATAAAACAAATATTTATCTACCTATTGTCTGTAAAATAATATACAAATATTATACACCAGTCTTGTGTAGCTATGGTTAATTTATTTAGCAGTTTATATCGTGTTACTTCAAAATTGAGTAAGTACATTATATAACTAGGTTCTCTTTTACACCGGTGCGTAAGCAAAGGGAAAAAAAAAAAAAAAAAAAAAAAAAAAAAA

Reads found in SRX6760545

Read ID:  SRA:SRR10023496.30476321.2

Query 254 AGTGAACTCGGTGACTGTGTGAACGTTGTTGCGGTTGAACGCCGCTGTAGGGATTCTTAA 313

||||||||||||||||||||||||||||||||||||||||||||||||||||||||||||

Sbjct 1 AGTGAACTCGGTGACTGTGTGAACGTTGTTGCGGTTGAACGCCGCTGTAGGGATTCTTAA 60

Query 314 TAAACAGCCGCACCACGCGGCCGAGTATGACGATGGACCAGCAAACAGGCCTCATGTCCC 373

||||||||||||||||||||||||||||||||||||||||||||||||||||||||||||

Sbjct 61 TAAACAGCCGCACCACGCGGCCGAGTATGACGATGGACCAGCAAACAGGCCTCATGTCCC 120

Query 374 TCAATATGTCCCCGTTTGATCTGAGCCCAG 403

||||||||||||||||||||||||||||||

Sbjct 121 TCAATATGTCCCCGTTTGATCTGAGCCCAG 150

##### >XM_022976553.1

GCACGTCTACCGAGCGGCACGCGTGCCGCTTGTTGTTAAAAAATACTCATTGCGATCGTAACATACCACGCGCTGTCAACACTTGTACGAACAGTAGTAATAATTACTTCGGTGACTGACACAGACGCTTAACGCTTGTTAAATTCTTAAACCGCTCGTATTGAACAAAATGTGTTGGATTTATGTGCTTCCTACAATAGTGCTTGAACAGGTGCAACCCACGAGCGGTAGCTAGTGTTGCGGCCAGACCGCTAGTGAACTCGGTGACTGTGTGAACGTTGTTGCGGTTGAACGCCGCTGTAGGGATTCTTAATAAACAGCCGCACCACGCGGCCGAGTATGACGATGGACCAGCAAACAGGCCTCATGTCCCTCAATATGTCCCCGTTTGATCTGAGCCCAGGGCCTGAAGGGTCTGGTTCAGGTGGGGGTCCCTCAGGTGCCTCGCAACAATACGTGCCGCAAGGCGCCGCGTATCAGTGCCCTCCTGAACAACAATCTTTCGGATATGCAAACTTGGACACATCCTATCTATTTCCAACAGGCGCTGGTGGCGAACCAGGCGCTTATTTGCCAGCAGCCGGCACAGTATGCGACCAAACCGACACTAAAGATGTGATCGAAGAACTTTGTCCCGTTTGTGGAGACAAAGTCAGCGGTTACCACTACGGGCTGCTAACGTGCGAGTCTTGTAAGGGATTCTTCAAGAGGACCGTACAAAATAAGAAGGTGTACACGTGTGTCGCTGAGCGAGCCTGCCACATAGACAAAACACAGCGGAAGCGCTGCCCATTTTGCCGCTTCCAAAAGTGCCTCGACGTTGGCATGAAACTCGAAGCTGTTCGAGCTGATCGCATGCGAGGAGGTCGTAACAAGTTCGGTCCTATGTACAAAAGAGATCGTGCCCGCAAACTGCAAATGATGCGACAGAGGCAAATTGCTGTGCAGACTTTACGTGGATCTTTGGGAGACAGTGGTCTAGTGCTTGGCTTTGCCTCGCCTTACGCAGCGGTTCCAGTCAAGCAAGAGATACAGATCCCACAAGTATCATCTCTCACATCATCGCCAGAGTCTTCACCAGGCCCAGCTTTGCTGGGTGCGCAGCCGCAGCCGCCCCAGCCTCCGCCGCCACCCGCTCACGACAAGTGGGAAGCGCATTCGCCTCACTCCGCCTCCCCAGACGCATTCGCGTTTGACGCACCAGCCACAGCAGCCGCAACGCCGTCGAGCACCGCCGAACCCACAAGCACTGAATCCCTACGAGTCTCACCCATGATACGCGAATTCGTACAAACTATTGATGACCGCGAGTGGCAGAATTCGCTATTTGGGCTCTTGCAGAGTCAGACTTACAATCAGTGTGAGGTCGATCTCTTCGAGTTAATGTGCAAAGTATTGGACCAAAACTTATTTTCACAAGTGGATTGGGCGAGAAACACCGTGTTCTTTAAGTATTTAAAGGTCGACGATCAAATGAAGCTGTTGCAGCACTCGTGGTCTGACATGTTGGTGTTAGATCACCTTCACCAGCGAATGCACAATGGCTTGCCTGATGAGACTACACTTCACAATGGCCAAAAGTTTGACCTCCTCTGCCTGGGTCTCCTGGGTGTACCATCGTTGGCTGACCACTTCAACGAGCTCCAGAACAAATTAGCAGAGCTGAAATTTGATGTCCCAGACTACATTTGCGTTAAATTTTTACTTCTATTAAACCCTGAAGTAAGAGGCATCGTTAATGTGAAGTGCGTCCGAGATGGTTATCAGACAGTTCAAGCTGCCCTCCTTGACTATACGCTGTCCTGTTATCCAACGATACAGGACAAATTCGGGAAACTAGTAATGGTAGTTCCTGAAATACATGCGCTAGCCGCACGTGGCGAGGAGCACCTGTACCAGCGACACTGCGCTGGACAGGCACCTACACAGACTCTCCTCATGGAAATGCTGCACGCGAAGCGCAAGCCGAACGGAGGTGAAATGGTTAACCGGAATGCCGAGCACACGTCGACACTTGACCGATTGTGCGGATTCCTGCCTAGCGACGCGATTGAGCAACAATCGCCAATATAGAATCTTGAAGTCTCAGTGAAGAGAAACAGACCACAAAAGCGCAAAGCGCTCCCGACACCGCATCAGAATAGAATATGCAACAAACTTTAC

### Hymenoptera (*Apis mellifera*)


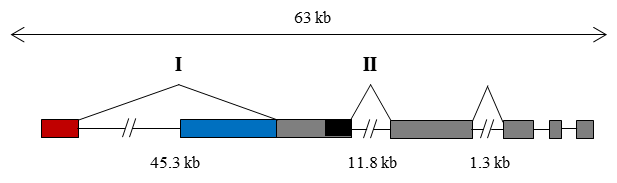


Reads found in SRX5544803

Read ID:  SRA:SRR8754042.18435690.2

Query 23 GTGGGGGCCATACGGCCCTCGAGAACACCACTGCGACGGCCCTTACGGAATCATCGTCAt 82

||||||||||||||||||||||||||||||||||||||||||||||||||||||||||||

Sbjct 1 GTGGGGGCCATACGGCCCTCGAGAACACCACTGCGACGGCCCTTACGGAATCATCGTCAT 60

Query 83 cctcttcctcctcttcctcctcttcctcctcttcctcctc 122

||||||||||||||||||||||||||||||||||||||||

Sbjct 61 CCTCTTCCTCCTCTTCCTCCTCTTCCTCCTCTTCCTCCTC 100

##### >aFTZF1_predicted_apis

ATGGCTACGGGGACAGGGGAAGGTGGGGGCCATACGGCCCTCGAGAACACCACTGCGACGGCCCTTACGGAATCATCGTCATCCTCTTCCTCCTCTTCCTCCTCTTCCTCCTCTTCCTCCTCCTCCTCCTCTTCATCCTCGGTACCAACTATCCTGACAGAAAACAACACGGCGATTAATCTGCCTTTGACTGAATCTGAACAAGAAACCCTTCATCAAGTACAACAGCATCAGGCTCAACAACAGCAGCAACAAACACATTCGCAATCATCGGTCAATTTGGTCACCGATCTGCAAACAAACGATACATCAGGAGCCGGAAGCGGCACTGGTGGCAGTGGAGGCAGCAATGGCGCGGCAAACGGTGCTGGAGGTGGTGGTTGCGGCACATCGACAGCCCTCGAGAGTAGCGGCAGCAATTATACCGGTGGCGGTGGTGCCGGTACCGGTGGTGGAGGAGGAACGGGTGGCGGCGGTGGTGCGGGTGCCGCTGGCGGTAGTGGGCCCACCGCAGGGAATGCCTCTGCTGGTGGTGGCGGTAGCGGTAGTGGTGCGGCCAGCGGTGCAAGTGGCGGCGGATCCGCTACGGTAAGCGGCAATGTCGCGATACCAACTACCACCTACTCATTACCAACTGGTACCCTCTGTCATCCCGGATTGGGTCAGGTCGGGGTTGGTGTCGTGACCGGCTCGATACCGTGTCCGTCCGAATTCCCCGATACCAAGGACATCATCATAGAAGAACTCTGTCCGGTCTGCGGCGACAAAGTTTCCGGATACCATTACGGGCTACTCACTTGTGAATCCTGCAAAGGTTTCTTCAAACGCACCGTCCAAAACAAAAAGGTCTACACGTGCGTCGCCGAGAGGTCCTGTCACATTGACAAAACGCAAAGAAAGCGGTGTCCCTACTGCCGTTTTCAGAAGTGCCTCGAAGTCGGCATGAAGCTTGAGGCCGTACGAGCGGATCGGATGAGAGGCGGTAGGAATAAATTTGGACCTATGTACAAGAGAGACCGAGCGCGGAAGCTACAAATGATGAGACAACGGCAGCTGGCACTGCAAACGATACGCGGCAGCCTCGGTGACCCATCGAACTATCCCTCCGCCGTAACGCCTTTCCTGCATATTAAACAGGAAATACAAATACCTCAGGTCTCGAGTCTAACCTCTTCTCCAGATTCGAGTCCATCCCCCGCAGCCGTGGCCGCTGGTTTGGTCACGACACAAGCTGGTAGCGGAGCTGGTCAGCATCAACTGATCGCACCGTCTTCCCAGCCAAACATTTCTGCTGGTAATCACCTGCACAATCTCAACCCTGGCCTGGATAGTAAGCTCTGGGCTGCCAATTCCACCACCCCGAGTCCGAAGGCCTTCAACTTCGGCGAACAGTCCACACAATCTCATGGAGCCACTGGCTCCGCACCCTCTACCGCCACGTTGAAAACTAGTCCGATGATAAGAGACTTTGTGCAAACAGTCGACGATCGCGAGTGGCAGGCATCGCTTTTTGGATTATTACAAAATCAAACGTACAATCAGTGTGAAGTGGACTTGTTCGAATTAATGTGCAAAGTGCTCGATCAAAATTTGTTCTCTCAGGTGGACTGGGCAAGGAATTCTGTATTCTTCAAGGATCTCAAGGTTGATGACCAAATGAAGTTGCTACAGCACTCCTGGTCAGATATGTTGGTGCTCGATCATCTTCATCAAAGGTTACACAATAATTTACCTGATGAGACTACGCTTCATAATGGTCAAAAGTTTGATCTCCTTTGTCTCGGCCTACTCGGGGTTCCTTCTTTGGCAGATCTTTTCAATGATTTATCGTCCAAACTTCAGGAACTCAAATTCGATCTTTCGGATTATATATGCATGAAATTCTTGATGCTGCTCAATCATGAAGTTCGTGGATTAGTTAACAAGAAACATGTACAAGAAGGTCACGAACAAGTTCAACAAGCTCTTTTGGATTATACTTTGACGTGTTATCCTTCTATACCGGATAAGTTTAACAAGCTGCTAGCAGTATTACCAGGAATCCACGTGGTGGCGAGCAGGGGAGAAGATCATCTTTATCAAAAACATTGTAGCGGTGGAGCACCAACTCAAACTCTTCTCATGGAAATGCTTCATGCTAAAAGAAAATGA

Reads found in SRX5544801

Read ID:  SRA:SRR8754044.9553356.2

Query 172 GATATGTTATTGGAGATGGAGCAGCATTCGGGTCTGATATCCCTGAATATGTCGCCGTTC 231

||||||||||||||||||||||||||||||||||||||||||||||||||||||||||||

Sbjct 100 GATATGTTATTGGAGATGGAGCAGCATTCGGGTCTGATATCCCTGAATATGTCGCCGTTC 41

Query 232 CATTTAAGCCCTCAGGGTAGTCCCCAAGGTGCGGCCAGTG 271

||||||||||||||||||||||||||||||||||||||||

Sbjct 40 CATTTAAGCCCTCAGGGTAGTCCCCAAGGTGCGGCCAGTG 1

##### >bFTZF1_Apis_mellifera_XM_006557392.2

AACCGTGGTTGTTCCGTCACATGACACGCCACTGGCAAATATAAAAAAGAAAAGAAACGGAAAACATTTATCATTTACCACAGTGGTTAAAATTAGAAGAATTTACAATCAATTCATATATGAGCCAATACGCCACAAGCACGTTCCTCCGTGCAGTATTCGCGCAAGGGTGATATGTTATTGGAGATGGAGCAGCATTCGGGTCTGATATCCCTGAATATGTCGCCGTTCCATTTAAGCCCTCAGGGTAGTCCCCAAGGTGCGGCCAGTGCTGGTCAGCAACAGCAACAAGCGCAACAACAACAACAACCACAATACGGTTCTTCGCCGTATGGGAATTGCGCTCAAAGTCCTCCTACAACAATGTGTCATCAATCACAGTCACAACAACAACCGCAACAACAGCAACAGTTGCCGGTACATAATCAGGCGCATTCGCAGGGTATATCGAATTTCGACGCCGCGTTTTTCGACTCCACCGCCCTTGAAGAACGGTGTCCCATGTGCGGGGACAAAATGTCTGGTTATCAGTATGGTCTGCTTACGTGCGAATCCTGTAAAGGTTTTTTCAAACGGAATAATTCGCCGTGTAGCAGTAAAAAAGTATATACGTGCCTATTCTCGCCAACGGGAGGTGGGGGAGGTGGGGGCAGCGGCGGAGGTGGTGGTGGCGGAGGTGGCGGTGGTGGAAATGGTAACAACGGTAACAACGGTGGAACCGGTAGCTGTGGCGGCGGCGCATCATCGGCACTCGAGATCGGATCTTGCGGCAACAAGAAGGTGTACACGTGTCTGTTCTCGCCAACAGGAGCCGGAAGCGGCACTGGTGGCAGTGGAGGCAGCAATGGCGCGGCAAACGGTGCTGGAGGTGGTGGTTGCGGCACATCGACAGCCCTCGAGAGTAGCGGCAGCAATTATACCGGTGGCGGTGGTGCCGGTACCGGTGGTGGAGGAGGAACGGGTGGCGGCGGTGGTGCGGGTGCCGCTGGCGGTAGTGGGCCCACCGCAGGGAATGCCTCTGCTGGTGGTGGCGGTAGCGGTAGTGGTGCGGCCAGCGGTGCAAGTGGCGGCGGATCCGCTACGGTAAGCGGCAATGTCGCGATACCAACTACCACCTACTCATTACCAACTGGTACCCTCTGTCATCCCGGATTGGGTCAGGTCGGGGTTGGTGTCGTGACCGGCTCGATACCGTGTCCGTCCGAATTCCCCGATACCAAGGACATCATCATAGAAGAACTCTGTCCGGTCTGCGGCGACAAAGTTTCCGGATACCATTACGGGCTACTCACTTGTGAATCCTGCAAAGGTTTCTTCAAACGCACCGTCCAAAACAAAAAGGTCTACACGTGCGTCGCCGAGAGGTCCTGTCACATTGACAAAACGCAAAGAAAGCGGTGTCCCTACTGCCGTTTTCAGAAGTGCCTCGAAGTCGGCATGAAGCTTGAGGCCGTACGAGCGGATCGGATGAGAGGCGGTAGGAATAAATTTGGACCTATGTACAAGAGAGACCGAGCGCGGAAGCTACAAATGATGAGACAACGGCAGCTGGCACTGCAAACGATACGCGGCAGCCTCGGTGACCCATCGAACTATCCCTCCGCCGTAACGCCTTTCCTGCATATTAAACAGGAAATACAAATACCTCAGGTCTCGAGTCTAACCTCTTCTCCAGATTCGAGTCCATCCCCCGCAGCCGTGGCCGCTGGTTTGGTCACGACACAAGCTGGTAGCGGAGCTGGTCAGCATCAACTGATCGCACCGTCTTCCCAGCCAAACATTTCTGCTGGTAATCACCTGCACAATCTCAACCCTGGCCTGGATAGTAAGCTCTGGGCTGCCAATTCCACCACCCCGAGTCCGAAGGCCTTCAACTTCGGCGAACAGTCCACACAATCTCATGGAGCCACTGGCTCCGCACCCTCTACCGCCACGTTGAAAACTAGTCCGATGATAAGAGACTTTGTGCAAACAGTCGACGATCGCGAGTGGCAGGCATCGCTTTTTGGATTATTACAAAATCAAACGTACAATCAGTGTGAAGTGGACTTGTTCGAATTAATGTGCAAAGTGCTCGATCAAAATTTGTTCTCTCAGGTGGACTGGGCAAGGAATTCTGTATTCTTCAAGGATCTCAAGGTTGATGACCAAATGAAGTTGCTACAGCACTCCTGGTCAGATATGTTGGTGCTCGATCATCTTCATCAAAGGTTACACAATAATTTACCTGATGAGACTACGCTTCATAATGGTCAAAAGTTTGATCTCCTTTGTCTCGGCCTACTCGGGGTTCCTTCTTTGGCAGATCTTTTCAATGATTTATCGTCCAAACTTCAGGAACTCAAATTCGATCTTTCGGATTATATATGCATGAAATTCTTGATGCTGCTCAATCATGAAGTTCGTGGATTAGTTAACAAGAAACATGTACAAGAAGGTCACGAACAAGTTCAACAAGCTCTTTTGGATTATACTTTGACGTGTTATCCTTCTATACCGGATAAGTTTAACAAGCTGCTAGCAGTATTACCAGGAATCCACGTGGTGGCGAGCAGGGGAGAAGATCATCTTTATCAAAAACATTGTAGCGGTGGAGCACCAACTCAAACTCTTCTCATGGAAATGCTTCATGCTAAAAGAAAATGAAACGATAAATTCATTTGGCACTTTTGCCATGAATTAGTCTATTGTTATATCTCAAAAATCAAGATAGAGCTCCTTATTGTCAATGTGGACCTCAAGTACCTATTAAGAGCTAATATACTACTGCTTAGGTGTTGGACCAAGTTAAGGAGCTCTGGTTCAAAAAATACCTATGCAAACGAATCAAGTATAGACGTACATAATTTACATATATACATAATATATGTATTATAAAATGTATCTAAATATATAAATATAATGACATATATTTTCTATATTTGATGTTGAAGTTTTAGAGATGTATCCATGAAGAATTATTACCCCTGTTGGTGGGTAAAAGCGCAGGGAAACAGTAAGCAAGATGCCTTGAAAACAGTACAAAGGGTGGTTAACAAAATATTGGTTAAAGGGCAGGCAATTTTTGTCTAAATAAGTAAAGCTATTTTTCTAATAATAGATGCGATTATTTTTAAGCACATACCACTGAAGCCTTGCTCTCATTTTAACAAAAAAATAATCCTAATGGTAACGAATGAAAAAAATTGTCGACTCTCCAGTCAATCGTGCTTCTGCAAGGGATTCAATGGGCATACTTGAATGACAGTAAAATTTCATGTGATATACTTATATATAACATATATACATACATATATGTCGCACGGCCACCTGGGCATCCTTAGAACTTCTTATAAAAAAAAAAAAATCTGCATTTTGTCACACTTGCACACGGCAGGGCTACTATATTGTAAATACTAGTTAACACATTCCAATTATGTTGTAAAGTGAATCATGTTAAATATGTAATTATAATATTTTTGTGTATAGTTTAATGAAATAAATATATTTCTGTTGCTTACTGAGAAAAAAAATCATTAGCTACTTAATGTCTTCACTAATTTAGACTTTTATTCATGTATTTCTTTTTATTATTTTTCAAATAGGTGGCGGAAAAAAATAACCACAGATTGCGATATTTTATTATTGTATATTGTATCGTAATAAAGATTCTGTTTAAGAAATAAAAGAATCATTGGAGCAAGGTTTATGTGGTCCTACATTATCTAGCGATTAATATTAAGTACATAAGATCAATATTGTCTTATCGCAGTTGTTAAATAGATAAATATTTACTGAATGTTAAGATAGTGATCGAATGTAGTAATTTTTCACTAATAGATTATTATTGTTTAGTCAATAGTCAGAAACTTTTCAGTGCTGTGAAGGTTGTTTTTTCGATGCCTGTGGTATCTAAGCCATCGGTAATTACACGTGCTAGATTCGAGGCTCAAGTGTGACAACAGGAACAGAAATCTTTGCTCGTACTGGTAGGCTGGTAGTACCACCCTTTGCAACTCCTACTGTTTATATCCTCTATCAAAGAAGTACCTGGGAAAAATGCTATTTTACTACTTTCACTTTTCCACCGACTTCGATATAAGATATTAAGTATAGATTTAAATAATTTGTTGATGTATGTAAAAAAAAAAAACTCGATGCCACATTAGATTAAGAATCGAGTGTTGAATCAAATCAGGAGGAGTAGCTGCGAGTATTACTACCGTGAAATATATATATATATATATTTATATATATATATATATATTTTCGAAAGCTTGCATGTACTTATCGTGTGAGTAAAGATTTATGTCCATGGTAAAATTTTTGAAATCTTCGATATAAATACAAATCATATATTGACATGTTCCCAAATTTTCAAATCGTCTGGTTCTTTTAATTGATTGATCAATTGAAAAGATACTTTACACTATTATTAACTTTTTAATGAATTAATTTTATTTAACTCGCATATAAATGTCTACCTCTTTGTAACTGTATTAAGTCTTACACCTTTACATTTTTAGTAAACCACATACCTCAGAATAGACACCCAGATAGCAAATTAAATGAAATATATGAATGTTACCTATGTAGACTTAAACTCTACAATTTCTATAATATGCGTATGGACATAATATAAAGGTTCCATTCAAGTATTGTACCCATTATATTTCCATGTTAATTATTTTAATTATTATTGTGTTTATTCCAAATTAGATTATATTTCTATATGATATATGCGATCGAAATTTAATAACTCAATGAAGTCTTTAAAATTTCTATTTATATTGTGTTATTGTTGGGTAAAGTCAATTAATATTTTATCTATTACATTTTCATTCAAATCTTTACTCAAGACTTCTTGTAGAACGTGTCGGTCTGATAATTTTGGAACAACTTACAGAAATCATAAATAAAATATTTCAAAAAACGAAAATGAAAATTGTCTCTTCTAAATATATGTCTCTAAACGATGAACTTTGTATGTATCCATCCTATTTAATCGTCCGCTCGATTCTGTTATAAATTGTTTATTAATAATTGTAATTAGGAAATATACATAATAATAATGGTGTTATAAGAATTGTGTTAAATATGTACAGTTACCAATTATAATTGGCGAACAATCTAATTTAATATCGATGTGATTTTTGATCCAACAAATAATTGGAATTAACATTAAATGATGAACTATTATGCGTTGTTAACACTGCCATTAGGAGTTGGCAATTGTAAATTATGTAGAAAAGAAATGATTAATTGTAAATATATCTTTATTATTAACTGA

### Blattodea (Blattella germanica)


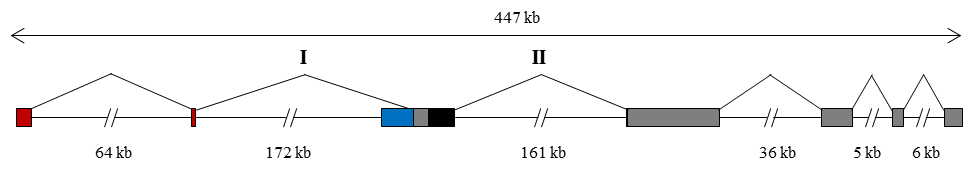


##### >FTZF1_ FM163377.1

TGGCCACGCGCCGRSGGGGGGGGGGGGGCBGAATGCAAAGTGTAAAGTGAATTGTCCTACAGACCTACATTTATACTGCGTTCATTTTTTTAAATATCACGTACAGAAGAAGATTTTTGTGAAGCCTTGGCACAGAGGAATAGTTCAGGGCTTTTTGAAGCTCTGAGTTGGAAGAATCGGGTTCAACATAGACGGATTACTGCCCAAAACATGCATGAGGAGTCAACCAGTAGGCCAATGAGCGTTCCCAGCTCGGTTGCCACTACCACCACTCAACCCACAGCTACAGACCACAATGCAAGCGAGCTACAAGTGTCATTCTCTTCTAGTTCTGCAGGTTCCGCCCTATATAGTCTACACAGCGAATCAGAGGAAGCCGGAGGTGGTGGTTCGACCATGGAGGTGGCCGCCGCGGGCAGCTATCAGGCGTCGCCGGGCGTCTCCGCCGCGACGGTGGCCGTCGTCACGGGCATGACAGGAGGAGACCTCCCGGACACGAAGGAGGGCATCGAAGAGCTCTGCCCCGTCTGCGGAGACAAGGTCTCGGGCTACCACTACGGCCTCCTCACGTGCGAGTCCTGCAAAGGATTCTTCAAGAGGACAGTGCAAAACAAGAAGGTCTACACATGCGTCGCCGAAAGGAGTTGTCACATCGACAAGACGCAGAGGAAGCGGTGTCCATATTGCCGCTTCCAGAAGTGCCTCGACGTCGGCATGAAACTTGAAGCTGTTCGCGCAGACCGCATGCGCGGCGGGAGAAACAAATTCGGCCCGATGTACAAGAGGGACAGAGCCCGCAAGTTGCAGATGATGCGGCAACGCCAGATAGCAGTGCAGACGCTCAGAGGATCGCACAGCCTCGGGGATAACGTGACCTTGAGCTACCCCCAGGCAGGAGGGGCGGGGACTTCCCCGTTCGCGAGTCTGCACATAAAGCAAGAGATCCAGATCCCGCAAGTATCGTCGCTGACGTCATCGCCCGACTCCTCCCCCAGCCCCATCGCCGTGGCCCTAGGCCAAGCGGGGGCGGCGTCCATCCCCGGAGCCTCGAGCGCCGGCCAGCAGGGCACTCTCCACATCATCGGGGGCGGGACACCGAGCACTGCCAACCCCACGGTCCTGAGCAGCGAGAGCAAACTGTGGACCGCGGCGTCGAACCCCACGACGCCCTCCCCGCACTCCCTGTCCCCGAAGACGTTCCACTTCGACAACGTGTTACCCAACGGAGGATCCACCCCCAGTGCGCCTTCCGCCCCCAACGCCAACGCCGGGGGCGGCACCGGCACCACCACCACCGGGCCCTCCTCCGTCAAGTATTCCCCCATCATTCGGGACTTTGTTCAAACTGTGGACGATCGCGAGTGGCAGAAGTCGTTGTTCGAACTCTTGCAGAACCAGACGTACAATCAGTGTGAGGTGGACCTGTTCGAACTGATGTGCAAAGTGCTGGACCAGAATTTGTTCTCTCAGGTCGACTGGGCGAGGAATTCCGTCTTCTTCAAGGACCTCAAGGTGGACGACCAGATGAAGCTGTTGCAGCATTCGTGGTCCGACATGTTGGTCCTGGACCACATGCACCAGCGCATGCACAACAACCTCCCTGACGAGACGACGCTGCCCAACGGACAGAAGTTCGACCTCCTGTGCCTCGGCCTCCTCGGGGTGCCCACCCTCGCAGACCACTTCAGCGACCTCATGGCCAAGTTGCAGGACCTCAAGTTCGACGTCACCGACTACATCTGCGTCAAGTTCCTGCTCCTTCTCAACCCCGAGGTTCGAGGCATCATGAACCGGAGGCACGTGCAGGAAGGCTACGATCAAGTGCAACAGGCACTCATGGACTACACAGTCAATTGCTACCCTCAAATCCAGGACAAGTTTACGAAACTACTGCTCGTGCTCCCAGAAATCCACCACATGGCCAGTAGAGGGGAGGAGCATCTGTACCACAAACACTGCAATGGTGGAGCCCCCACGCAAACACTGCTCATGGAGATGCTACATGCCAAACGGAAATAGCATCCGCACGCTCCAAGATCCAAGGCAACCGTTGTTAACTGAAATGAATTGTTGACAAAGTCCCKCGAGTCTAAGGCCGGTCCCTCAAATCTCGCGTTGGTTCTAATCAGTTCGTCCAGCCGCATCATCACGTAGGAGCTGCCACAGTGGTGATTGGTGTAGGCCTTTGGCAGCGGAGTAGGTGTTCAGGTGGTGCGTTGGTTACCAAGTGTGCGCGTAGTCAGTGTGGACAAGATGGGCATTATGCGAGTTACCTATAACGCAGCAAGTGCAATACAAAACGTCTGGCAAAACACATTGGCTTCTTGTACTTTAAAAAAAAAGAGAAACTCATAGCACAGTGAAAGACTACTGGAATGTCATATTTTATTATGCCTGTTGACTTTTTTTTAAAGAAAG

##### >bFTZF1_suggested

ATGTTATTAGAAATGGATCACCATCCAGGGCTCATGTCTCTCAATATGTCTCCTTTCAACCTGTCTCCCAACGGCAGCAATGGGAGTAACAGTGCTGTGACAATGACTTCCAGTGGAAGCCCCCACAATAACAATATGCCGCCCCCGTTGTACGGTTCGCCGCCCCTCGGCCAATACCCGATGGGGGGCGCAATGCAACACCAACCCCCACAGCAACAGCACATGATGGGTGGTCAGCACATGGGTAACCTGGATGCGTCGTATTTGTTCCCAGCCGGAGGTGGTGGTTCGACCATGGAGGTGGCCGCCGCGGGCAGCTATCAGGCGTCGCCGGGCGTCTCCGCCGCGACGGTGGCCGTCGTCACGGGCATGACAGGAGGAGACCTCCCGGACACGAAGGAGGGCATCGAAGAGCTCTGCCCCGTCTGCGGAGACAAGGTCTCGGGCTACCACTACGGCCTCCTCACGTGCGAGTCCTGCAAAGGATTCTTCAAGAGGACAGTGCAAAACAAGAAGGTCTACACATGCGTCGCCGAAAGGAGTTGTCACATCGACAAGACGCAGAGGAAGCGGTGTCCATATTGCCGCTTCCAGAAGTGCCTCGACGTCGGCATGAAACTTGAAGCTGTTCGCGCAGACCGCATGCGCGGCGGGAGAAACAAATTCGGCCCGATGTACAAGAGGGACAGAGCCCGCAAGTTGCAGATGATGCGGCAACGCCAGATAGCAGTGCAGACGCTCAGAGGATCGCACAGCCTCGGGGATAACGTGACCTTGAGCTACCCCCAGGCAGGAGGGGCGGGGACTTCCCCGTTCGCGAGTCTGCACATAAAGCAAGAGATCCAGATCCCGCAAGTATCGTCGCTGACGTCATCGCCCGACTCCTCCCCCAGCCCCATCGCCGTGGCCCTAGGCCAAGCGGGGGCGGCGTCCATCCCCGGAGCCTCGAGCGCCGGCCAGCAGGGCACTCTCCACATCATCGGGGGCGGGACACCGAGCACTGCCAACCCCACGGTCCTGAGCAGCGAGAGCAAACTGTGGACCGCGGCGTCGAACCCCACGACGCCCTCCCCGCACTCCCTGTCCCCGAAGACGTTCCACTTCGACAACGTGTTACCCAACGGAGGATCCACCCCCAGTGCGCCTTCCGCCCCCAACGCCAACGCCGGGGGCGGCACCGGCACCACCACCACCGGGCCCTCCTCCGTCAAGTATTCCCCCATCATTCGGGACTTTGTTCAAACTGTGGACGATCGCGAGTGGCAGAAGTCGTTGTTCGAACTCTTGCAGAACCAGACGTACAATCAGTGTGAGGTGGACCTGTTCGAACTGATGTGCAAAGTGCTGGACCAGAATTTGTTCTCTCAGGTCGACTGGGCGAGGAATTCCGTCTTCTTCAAGGACCTCAAGGTGGACGACCAGATGAAGCTGTTGCAGCATTCGTGGTCCGACATGTTGGTCCTGGACCACATGCACCAGCGCATGCACAACAACCTCCCTGACGAGACGACGCTGCCCAACGGACAGAAGTTCGACCTCCTGTGCCTCGGCCTCCTCGGGGTGCCCACCCTCGCAGACCACTTCAGCGACCTCATGGCCAAGTTGCAGGACCTCAAGTTCGACGTCACCGACTACATCTGCGTCAAGTTCCTGCTCCTTCTCAACCCCGAGGTTCGAGGCATCATGAACCGGAGGCACGTGCAGGAAGGCTACGATCAAGTGCAACAGGCACTCATGGACTACACAGTCAATTGCTACCCTCAAATCCAGGACAAGTTTACGAAACTACTGCTCGTGCTCCCAGAAATCCACCACATGGCCAGTAGAGGGGAGGAGCATCTGTACCACAAACACTGCAATGGTGGAGCCCCCACGCAAACACTGCTCATGGAGATGCTACATGCCAAACGGAAATAGCATCCGCACGCTCCAAGATCCAAGGCAACCGTTGTTAACTGAAATGAATTGTTGACAAAGTCCCKCGAGTCTAAGGCCGGTCCCTCAAATCTCGCGTTGGTTCTAATCAGTTCGTCCAGCCGCATCATCACGTAGGAGCTGCCACAGTGGTGATTGGTGTAGGCCTTTGGCAGCGGAGTAGGTGTTCAGGTGGTGCGTTGGTTACCAAGTGTGCGCGTAGTCAGTGTGGACAAGATGGGCATTATGCGAGTTACCTATAACGCAGCAAGTGCAATACAAAACGTCTGGCAAAACACATTGGCTTCTTGTACTTTAAAAAAAAAGAGAAACTCATAGCACAGTGAAAGACTACTGGAATGTCATATTTTATTATGCCTGTTGACTTTTTTTTAAAGAAAG

### Thysanoptera (Frankliniella occidentalis)


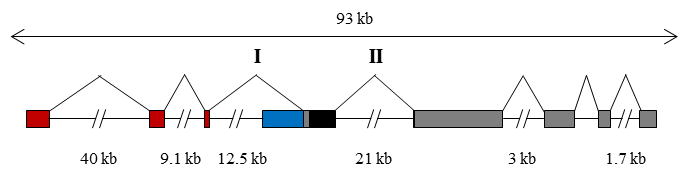


##### >XM_026421392.1_Frankliniella_occidentalis_FTZF1

GAAGTGTAAGTGAAGTGGCCTACAGACCTACATAATCCAATCGTTCAAATTTGTATTGCGTAACGGCGTAACGAAGGATCTGCGGCGAGTGCTGGACCTCAGAGGACGTGTGTGAAAGTGCGTCGATGTGGGGACGTGAAGGTCGCGGGGGAACAGTGCGTCTGCGTGGTGTAGGGACATGCACGAAGACGCCACCACGGCCAGGGGCAGCCAGGACAGCCTCGTCCGCAGCAGCAGCTACCACCGCCGCAGCAGCATGAGCTCAGCCGCCTCCCCCTCCGCCTCCCCGCTCGAGCCCGAGCTCGAGTCCGACCCAGCTGACACGGTCGAGCTCCAGGTTGCCTTCTCGTCGTCCAGCAACTCAGGCGACCAGAGCCGGGTGGAGCTGGGCGTCCCGGGCGGCGGCGCGGTGGGCCTGGAGCACGGCCTGGAGCACGACGACATCGAGCTGGAGGGCGACGAGCGCGTCCTGGAGCTCATCGTGGACTCAGACTTTGCCGAGAGCGATGCGGGCCAACCCTCCACTCAAGACTACCCGGCCGGCGCCGTGCGCCCCGACCTGCCGGACACCAAGGAGGGCATCGAGGAGCTGTGCCCCGTGTGCGGCGACAAAGTGTCTGGATACCACTACGGCCTGCTCACGTGCGAGTCGTGCAAGGGCTTCTTCAAGCGCACCGTCCAGAACAAGAAGGTGTACACGTGCGTGGCCGACCGGTCGTGCCACATCGACAAGACGCAGCGCAAGCGCTGTCCGTTCTGCCGCTTCCAGAAGTGCCTCGAGGTCGGCATGAAGCTGGAAGCGGTGCGAGCGGACCGGATGCGCGGCGGGAGGAACAAGTTCGGCCCCATGTACAAGCGGGACCGGGCGCGGAAACTGCAGATGATGAGGCAGCGGCAGATCGCCGTGCAGACCATCCGGGGCCACGGCAGCTCGCTGTCCAACTCGCTGTCCAGCCAGGGCAACCAGGGCCTGTCCGAGTCGGGGCTGTCGTACCCGCAGTCGTCGGCGCAGTCCTTCGCCTCGCTGCACATCAAGCAGGAGATCCAGATCCCCCAAGTGTCCTCGCTCACCTCCTCGCCCGACTCGTCGCCCAGCCCCATCGCTGTGGCGCTCGGTCAGGCGTCGGCGTCGGCGTCCGGCGGCGTCTCGGCGCCCCCGGGCCTCCAGCTCGCCGCCCCCACCTCCGCCCCCGGCTCAACGGCCGCCTCCACGGCCACCAGCCTGGCCGCCACCTCGGACCACAAGCTCTGGGGCGGCGGCACGGGCGGCGGCGGCACCGGCGGCGGCAACACGCCGTCCAGAGACCTCCTCGACGGGCTCATGGGCTCCACCTCGCCGACGGCGACCACCACCAAGATGACGCCGATGATGAGGGACCTGGTGCAGTCAGTGGATGACCGGGAGTGGCAGAACTCGCTGTTTGGTCTGCTTCAGAACCAGACTTACAACCAGTGCGAGGTGGACCTCTTTGAACTCATGTGCAAAGTGCTGGACCAGAACCTCTTCTCGCAAGTAGACTGGGCGAGAAATTCAGTCTTCTTCAAGGACCTCAAGGTTGATGACCAGATGAAACTACTCCAGTACTCATGGTCAGACATGCTGGTGCTAGATCATCTCCACCAACGGCTCCATAACCAACTCCCAGATGAGACAACGCTTCCCAATGGACAGAAGTTCGATCTGCTTTGTCTAGGCCTTCTGGGAGTGGCATCTTTGGCGGATCAGTTCGCTGCATTGGCGCACCAATTCGCTCTCCTCAAATTCGATATCTCGGATTACATCTGTTTGAAGTTTCTGCTTCTGCTCAACCCTGATGTCAGAGGGATAATGAATCGAAAGCACGTACAGGAGGGCCAGACCCAGGTGAAAACAGCACTCCTGGACTACACTATGACGAATTATCCACAACTTCAGGATAAGTTCAACAGACTACTACAGCTCCTACCAGACATTCATGAGATGGCAGCCCGAGGAGAGGACCACCTATATCAGAAGCATTGTTCGGGCGGAGCACCAACACAAACTCTACTAATGGAAATGCTACATGCGAAGCGCAAGTAGTGTTAATGTTACCAATTACCTGCCCAACACGAGAGAAGAGTGCACTGCCATGAGTTGTAGTGGCATGAGCTTGAGAGGGCTGGGCCCTCCAAATCCTGCCACACACCTCAATCCCACACCAAGGAAAGATGAGGACATCAGATTCGAAAAGCCTTGCAACATGTCATGGGCATCTTAGTCAACTTCAGGATGTTCCCATTTTATGTTAATACAGTCAAGTTATAGTTATAACTTTTAAAAGATTATTATTTATGTGACTTTAGTGATCATATTTTTTTTTTTTACTCAATTCTGCGGTGTATGGCATGTGCAGTCTAATGGTAAACATCACATAAAGTGCCCTGTAGAGAACTCTCGGATGTTGCAAATGTACCTAACACAGTGCGGCTCCTGGCCCACACATGTTTGTTACCTCTGTACAGCTTAACATAGTGAATAATATAAGTAAATTAGGACATTATTCTATTTTGTAAAAAGATTATCTATGCATTATCCTTGCACTTACTGTGAAATATTGTGAAATTATTAAGGCATGTTTTCTGTTGTTTTTTTTTTCTTTCCCAAAGGAACAAGTTATTATGCTATTTAAGAAAAGCTGTGGATTATGTGCAATATGTTAAAAATAGTTTGGTATCGAGTCCTATTTGTTAAGTAAAGTTTTGACAACACAGAAAATTCTATTTATTGGCTTGTTTTTGTTGCTGAAAATGTTAATACCTTTGTTAGTCAGTTGGTTGTGTCCATGTCTTGTGAAATACCTCTGTAAAAACCATGTTGAAATTTAAGAAGAGTGACCAATATTTTTGTTGATAGAAGATTAAATACTGGTTTAAGTTACACAATAATATATATATTTGTTTTATTTTTTTATGAATGTAAATTGGATACCTTCCTGTAATTCTTAATATTGTGACAATTAGCTGCCTTCAAAAAAGTACAAAACTGGAAAAGTGTGGTAGAATTTGCTACCTCAGAATGGTTCACTTCTTCACTATACATGTTATTATTGTTACTTAGATTTGGCACATTTTGTGTTGTATCTACACATTCCGGCGGCCGTCCCGCACAGTAATGATGTACATTTGGATGTATGGTTTGTACATACAGTGTATTCTGTGTGTACCTCACATTTTGTAAAGTTAGGGTTGCTTGATGCTTGTTTACAGATCAATATTTACAGGACACTTCTGTTCATATTAGTTAATTTAATTTTTTGATATTGTCTTTGTACGTAGCTGCATTTTTTTGCTTTTCTCATGCTTCTTGTATAGGCTCAAAATTTCAGTCAACCAGAAATTTTATTTATTTTTTCTTCCCTACATTCCATTCTTTCGCTTGATTTTGTTTGTTTCATTCCCAAAAGAGCTGCTGCCGTGTGCCGGTGTGGGAATCTTCCTTGACTAATGTATCTGAGAAGTATATAAAAGAGCTAACTTGTCTATTTTTGTTATTATAGTTAACTTCTTATAGAACAAAATCGGGCATGGTGATGGAATTAAATACTAAAAGATGTATAGTTTTGTAGAGCAACAGAGCATTGTGCCTACTTTGTTTATATCTACAATGTACTGTAGAATAGAGGTGTTGTGTAAACACATCACAGGAAGGAGGGCAGGAGGGATCAAACTTATGCATATGTTGTGCATGCAAGTGTTACCAGCCGGTTGGGCGTTGTGATAATCCCAGCCCAGCCTCCACACTGTACATACTATATTCATTGCGAAATGTTTCTCCCAGATTACGAAAAACCAGAACCTGACTTTGATGTGTTGATACCGAGACCGAGACTCACCATTTTTGTCAACATGAATGACTTGAAGTACAATTGATATTATTGGCTGTATAATTTTTTTCTTCATGTATTGAAGCACTGGTTCAATCAAATCACCTCTGAAATTTTATTAAACATTGCGCAATGGCGAGCTTCTCAAAATGAGCTGTGAAAGTGAATGGTCCAGACATTGATTTGTTTTATGGTTTAGATATACTTCTTTAAGGCAGCCTCTCATATGTTTTTCAGAACTAATGCCTTTACTGCCATTGAAGTTTCTCTTTTCGAGTAAATTTGACCTGATGGACCAGATTGGCCTTATGAGCATGGTGTTGAGGCTGTTGTTATTGTAGATTTTTGAAATGATGTGATGTGTGAAGTAGTGAGCAGCATCCAATGAGAGGCACAATGTGCTAAAATCTGCTGCTCCTACTCTCAATGCTCAAGATTGTAGCTTAGCTGGAAAAATGAGATTTGTATTGTTAGGTTTGAGTTTTGTACTGAGGGCACTGTCCCTTGCGCACACAAGGAACCAGTACAAATGAGCGCTGTGAAGGACCCAGGAATGGATCACTTGTTTTGCCTAAGAAGCTCCAACGAATTACCAATTTAATCATACGTTAAATTCATATTTTCATTCACCTATGGCTGTTCTTAATTTTGAAGAGGCTGTATTAATGTTAAATTTTTCTTTTGAAGGCTATTTTATGTATAGAAAGCAAATGTGTTGGTGTAGATTCTTTATGGTGGCTGTTGTACTACCAGTCATATGGCTGATAATACTACAGCCTCTATGCTCTTAATTAATGGTACTAAAGTTTACTTAAATTTCATTGTGTTCACTGCCAAACCCAAGTTTATGATTAGGCTGTTATATAGAAATGTAAAGTTTGAATGAACCGTTTGCCGGATTAACGACTCATTCATTATGTCTATTTTCCAGTGATTCTTAAAATAGCTTTGTATAAATCATAGAGGAAATATATTTGTTGTTGGCACTCAGAGCCACTATTTTGTAAAAAAAAGATTATTGAAAAGCTAATGTTAGAGTATGAAATTACTAAGAAAGGTGTATAAAATATTATTTTCCTCATCTATTAAAAATTGTGTACAGATAATATTGTCACGTGAAGTAAGTTTCATGGCAATTTGTTATTTTTGTTGTAATTCTCAAAACTTTGGCACAGTTATTTTAAAGACCCAAGTAAAAGTGTTTCACTGTTAATAGATATGCCTTCCACTCATTATACATCCTCATTTATTTGATCTGATTATAGCTATTTTTTTTTTTTTTAATTCATGGGACATTTCTTTCTTGAGCAAAAGTTTTCACTTTCTGAAGAGACATTTATGTTACCTCATTACAATCCACATCAAGTCACCTACACTCTGTGCTGTAATGTATATTGCTACATTTGTACAAAAAAAGCAGTATCCGTTGTAAGGATATCAACGTTTTAAATAAAAACATGTGTTGTTACATGTTGGATGTGATACAAACCCTCA

##### >bFTZF1_putative_Frankliniella

ATGTTATTAGACATGGAGCACCATAGCGCGCTGCTCTCCCTCAATATGAGCCCGTTCAACCTCAGCCCCGGCTCGGGCGGCGGCGGTACCCAGGGCGGTGGTGGTGGCGGCGGCGGTGGCGGCGGCGGCTCCGGGGTGACGGCGGCGCTGACGCCGTCCAGCCTGGCGCCGCCGTCGCTGTACGGGTCGCCGCCGCTGACGGGCTGGCAGCAGCCGCCCCAGCAGCAGCAGCAGCACCAGCAGCAGCAGCACCACCAGCAGCAGCAGCAGCACCACCAGGGCCACGGCGGCGTCGGCCACGGCATGCTCATGAGTAACCTGGACGCGTGTTTGTTCCCCGGCGCAGACTACCCGGCCGGCGCCGTGCGCCCCGACCTGCCGGACACCAAGGAGGGCATCGAGGAGCTGTGCCCCGTGTGCGGCGACAAAGTGTCTGGATACCACTACGGCCTGCTCACGTGCGAGTCGTGCAAGGGCTTCTTCAAGCGCACCGTCCAGAACAAGAAGGTGTACACGTGCGTGGCCGACCGGTCGTGCCACATCGACAAGACGCAGCGCAAGCGCTGTCCGTTCTGCCGCTTCCAGAAGTGCCTCGAGGTCGGCATGAAGCTGGAAGCGGTGCGAGCGGACCGGATGCGCGGCGGGAGGAACAAGTTCGGCCCCATGTACAAGCGGGACCGGGCGCGGAAACTGCAGATGATGAGGCAGCGGCAGATCGCCGTGCAGACCATCCGGGGCCACGGCAGCTCGCTGTCCAACTCGCTGTCCAGCCAGGGCAACCAGGGCCTGTCCGAGTCGGGGCTGTCGTACCCGCAGTCGTCGGCGCAGTCCTTCGCCTCGCTGCACATCAAGCAGGAGATCCAGATCCCCCAAGTGTCCTCGCTCACCTCCTCGCCCGACTCGTCGCCCAGCCCCATCGCTGTGGCGCTCGGTCAGGCGTCGGCGTCGGCGTCCGGCGGCGTCTCGGCGCCCCCGGGCCTCCAGCTCGCCGCCCCCACCTCCGCCCCCGGCTCAACGGCCGCCTCCACGGCCACCAGCCTGGCCGCCACCTCGGACCACAAGCTCTGGGGCGGCGGCACGGGCGGCGGCGGCACCGGCGGCGGCAACACGCCGTCCAGAGACCTCCTCGACGGGCTCATGGGCTCCACCTCGCCGACGGCGACCACCACCAAGATGACGCCGATGATGAGGGACCTGGTGCAGTCAGTGGATGACCGGGAGTGGCAGAACTCGCTGTTTGGTCTGCTTCAGAACCAGACTTACAACCAGTGCGAGGTGGACCTCTTTGAACTCATGTGCAAAGTGCTGGACCAGAACCTCTTCTCGCAAGTAGACTGGGCGAGAAATTCAGTCTTCTTCAAGGACCTCAAGGTTGATGACCAGATGAAACTACTCCAGTACTCATGGTCAGACATGCTGGTGCTAGATCATCTCCACCAACGGCTCCATAACCAACTCCCAGATGAGACAACGCTTCCCAATGGACAGAAGTTCGATCTGCTTTGTCTAGGCCTTCTGGGAGTGGCATCTTTGGCGGATCAGTTCGCTGCATTGGCGCACCAATTCGCTCTCCTCAAATTCGATATCTCGGATTACATCTGTTTGAAGTTTCTGCTTCTGCTCAACCCTGATGTCAGAGGGATAATGAATCGAAAGCACGTACAGGAGGGCCAGACCCAGGTGAAAACAGCACTCCTGGACTACACTATGACGAATTATCCACAACTTCAGGATAAGTTCAACAGACTACTACAGCTCCTACCAGACATTCATGAGATGGCAGCCCGAGGAGAGGACCACCTATATCAGAAGCATTGTTCGGGCGGAGCACCAACACAAACTCTACTAATGGAAATGCTACATGCGAAGCGCAAGTAGTGTTAATGTTACCAATTACCTGCCCAACACGAGAGAAGAGTGCACTGCCATGAGTTGTAGTGGCATGAGCTTGAGAGGGCTGGGCCCTCCAAATCCTGCCACACACCTCAATCCCACACCAAGGAAAGATGAGGACATCAGATTCGAAAAGCCTTGCAACATGTCATGGGCATCTTAGTCAACTTCAGGATGTTCCCATTTTATGTTAATACAGTCAAGTTATAGTTATAACTTTTAAAAGATTATTATTTATGTGACTTTAGTGATCATATTTTTTTTTTTTACTCAATTCTGCGGTGTATGGCATGTGCAGTCTAATGGTAAACATCACATAAAGTGCCCTGTAGAGAACTCTCGGATGTTGCAAATGTACCTAACACAGTGCGGCTCCTGGCCCACACATGTTTGTTACCTCTGTACAGCTTAACATAGTGAATAATATAAGTAAATTAGGACATTATTCTATTTTGTAAAAAGATTATCTATGCATTATCCTTGCACTTACTGTGAAATATTGTGAAATTATTAAGGCATGTTTTCTGTTGTTTTTTTTTTCTTTCCCAAAGGAACAAGTTATTATGCTATTTAAGAAAAGCTGTGGATTATGTGCAATATGTTAAAAATAGTTTGGTATCGAGTCCTATTTGTTAAGTAAAGTTTTGACAACACAGAAAATTCTATTTATTGGCTTGTTTTTGTTGCTGAAAATGTTAATACCTTTGTTAGTCAGTTGGTTGTGTCCATGTCTTGTGAAATACCTCTGTAAAAACCATGTTGAAATTTAAGAAGAGTGACCAATATTTTTGTTGATAGAAGATTAAATACTGGTTTAAGTTACACAATAATATATATATTTGTTTTATTTTTTTATGAATGTAAATTGGATACCTTCCTGTAATTCTTAATATTGTGACAATTAGCTGCCTTCAAAAAAGTACAAAACTGGAAAAGTGTGGTAGAATTTGCTACCTCAGAATGGTTCACTTCTTCACTATACATGTTATTATTGTTACTTAGATTTGGCACATTTTGTGTTGTATCTACACATTCCGGCGGCCGTCCCGCACAGTAATGATGTACATTTGGATGTATGGTTTGTACATACAGTGTATTCTGTGTGTACCTCACATTTTGTAAAGTTAGGGTTGCTTGATGCTTGTTTACAGATCAATATTTACAGGACACTTCTGTTCATATTAGTTAATTTAATTTTTTGATATTGTCTTTGTACGTAGCTGCATTTTTTTGCTTTTCTCATGCTTCTTGTATAGGCTCAAAATTTCAGTCAACCAGAAATTTTATTTATTTTTTCTTCCCTACATTCCATTCTTTCGCTTGATTTTGTTTGTTTCATTCCCAAAAGAGCTGCTGCCGTGTGCCGGTGTGGGAATCTTCCTTGACTAATGTATCTGAGAAGTATATAAAAGAGCTAACTTGTCTATTTTTGTTATTATAGTTAACTTCTTATAGAACAAAATCGGGCATGGTGATGGAATTAAATACTAAAAGATGTATAGTTTTGTAGAGCAACAGAGCATTGTGCCTACTTTGTTTATATCTACAATGTACTGTAGAATAGAGGTGTTGTGTAAACACATCACAGGAAGGAGGGCAGGAGGGATCAAACTTATGCATATGTTGTGCATGCAAGTGTTACCAGCCGGTTGGGCGTTGTGATAATCCCAGCCCAGCCTCCACACTGTACATACTATATTCATTGCGAAATGTTTCTCCCAGATTACGAAAAACCAGAACCTGACTTTGATGTGTTGATACCGAGACCGAGACTCACCATTTTTGTCAACATGAATGACTTGAAGTACAATTGATATTATTGGCTGTATAATTTTTTTCTTCATGTATTGAAGCACTGGTTCAATCAAATCACCTCTGAAATTTTATTAAACATTGCGCAATGGCGAGCTTCTCAAAATGAGCTGTGAAAGTGAATGGTCCAGACATTGATTTGTTTTATGGTTTAGATATACTTCTTTAAGGCAGCCTCTCATATGTTTTTCAGAACTAATGCCTTTACTGCCATTGAAGTTTCTCTTTTCGAGTAAATTTGACCTGATGGACCAGATTGGCCTTATGAGCATGGTGTTGAGGCTGTTGTTATTGTAGATTTTTGAAATGATGTGATGTGTGAAGTAGTGAGCAGCATCCAATGAGAGGCACAATGTGCTAAAATCTGCTGCTCCTACTCTCAATGCTCAAGATTGTAGCTTAGCTGGAAAAATGAGATTTGTATTGTTAGGTTTGAGTTTTGTACTGAGGGCACTGTCCCTTGCGCACACAAGGAACCAGTACAAATGAGCGCTGTGAAGGACCCAGGAATGGATCACTTGTTTTGCCTAAGAAGCTCCAACGAATTACCAATTTAATCATACGTTAAATTCATATTTTCATTCACCTATGGCTGTTCTTAATTTTGAAGAGGCTGTATTAATGTTAAATTTTTCTTTTGAAGGCTATTTTATGTATAGAAAGCAAATGTGTTGGTGTAGATTCTTTATGGTGGCTGTTGTACTACCAGTCATATGGCTGATAATACTACAGCCTCTATGCTCTTAATTAATGGTACTAAAGTTTACTTAAATTTCATTGTGTTCACTGCCAAACCCAAGTTTATGATTAGGCTGTTATATAGAAATGTAAAGTTTGAATGAACCGTTTGCCGGATTAACGACTCATTCATTATGTCTATTTTCCAGTGATTCTTAAAATAGCTTTGTATAAATCATAGAGGAAATATATTTGTTGTTGGCACTCAGAGCCACTATTTTGTAAAAAAAAGATTATTGAAAAGCTAATGTTAGAGTATGAAATTACTAAGAAAGGTGTATAAAATATTATTTTCCTCATCTATTAAAAATTGTGTACAGATAATATTGTCACGTGAAGTAAGTTTCATGGCAATTTGTTATTTTTGTTGTAATTCTCAAAACTTTGGCACAGTTATTTTAAAGACCCAAGTAAAAGTGTTTCACTGTTAATAGATATGCCTTCCACTCATTATACATCCTCATTTATTTGATCTGATTATAGCTATTTTTTTTTTTTTTAATTCATGGGACATTTCTTTCTTGAGCAAAAGTTTTCACTTTCTGAAGAGACATTTATGTTACCTCATTACAATCCACATCAAGTCACCTACACTCTGTGCTGTAATGTATATTGCTACATTTGTACAAAAAAAGCAGTATCCGTTGTAAGGATATCAACGTTTTAAATAAAAACATGTGTTGTTACATGTTGGATGTGATACAAACCCTCA

### Coleoptera (Tribolium castaneum)


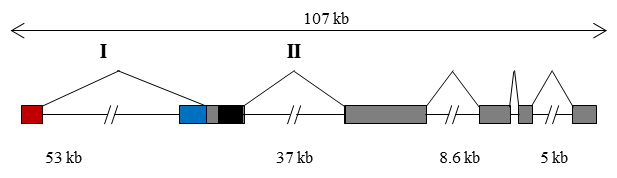


Read found in SRX6385151

READ ID: SRA:SRR9622227.2144583.1

Query 145 GGAGGCTCTGGAGCGTGCTGGAGGCGGTCATCATCGCGTTGTAAGTGCGTTTTCGGATAA 204

||||||||||||||||||||||||||||||||||||||||||||||||||||||||||||

Sbjct 76 GGAGGCTCTGGAGCGTGCTGGAGGCGGTCATCATCGCGTTGTAAGTGCGTTTTCGGATAA 17

Query 205 ACAATGCACGAAGAA 219

|||||||||||||||

Sbjct 16 ACAATGCACGAAGAA 2

##### >XM_008193153.2

TGAGTCGGCAGTCATGTCTGTCTGAATGCAAAGTGTAAGCGAAGTGGCCTACAGACCTACATTTCGTCAACGTTCGATTTTTAAATCGCTCAAAGAAATAGGTATTTGTGGAACCTTGTCGTGTGTTTGAAGGTCGGGGCTTCGGGAGGCTCTGGAGCGTGCTGGAGGCGGTCATCATCGCGTTGTAAGTGCGTTTTCGGATAAACAATGCACGAAGAAACGGCAAGTATGAGCGTTCCTGGCCCTGTTGCCGTGTCTGCGGGCCAGAGCGAGGGCAGCCCCACTCCGACGGCCGTCGTCGTCAACGCCGCCGAGGTGGACCTGGAGGTGGCGCCCGACGCCGACAACGTGGAGCTGCAAGTGCCTTTCGCGGCCAATTCCGGAGGGGCCAGCGCCATCTCCGGCGTCGACATGGGCGCCAGCTACCAGATTTCCGGGCCGGCGACGTCGCTCACAGCCGGCGACCTCCCAGACACGAAGGACGGCATCGAGGAGCTCTGTCCGGTCTGCGGGGATAAGGTCTCCGGGTACCACTACGGCCTCCTCACGTGTGAATCTTGCAAGGGATTTTTCAAACGAACTGTTCAAAATAAGAAAGTTTACACGTGCGTGGCGGAAAGAAGTTGTCACATAGACAAAACGCAAAGGAAAAGGTGTCCCTACTGCCGGTTTCAAAAGTGCTTAGAAGTCGGGATGAAACTTGAAGCTGTCCGAGCAGACCGAATGAGAGGCGGCAGGAACAAATTCGGTCCGATGTACAAACGCGACCGTGCCCGAAAACTGCAAATGATGCGACAACGACAGCTGGCGGCGCAAACGCTACGGGGAAGCTCTCTCGGTGACGCCATGTACAGCAGCCAGCCCGGAACATCACCCTTCGCCAACATCCACATCAAGCAAGAAATCCAAATTCCGCAAGTGTCATCTCTAACATCGTCGCCCGACTCGTCGCCGAGCCCCATCGCCGTCGCCCTGGGCCAGGTCAACACCTCCAACCTGGTGCAGCAGGCGTCCAGCCAGCAGCCGGCGCTGCAGATCGTCGGGGTGCCCGGGGGGCCCTCCTCCATGGTGATGGGGCCCGACAACAAGCTCTGGGGCTCCGCCAACTCCACCACCACCTCGCCCCACTCGCTCAGCCCCAAGGCGTTCCAGTTCGACACGGTGGTGCAAGGGGGCAGCGCGCCGCCCTCCAGTAAAGTGTCGCCGCTTATCAGGGACTTTGTGCAAGCCATCGACGATCGCGAGTGGCAAAACTCTTTGTATACTTTACTCCAGAACCAAACGTATAATCAGTGTGAAGTGGACTTATTTGAACTTATGTGTAAAGTTTTGGACCAAAATCTGTTCTCTCAAGTCGATTGGGCGAGGAATTCTGTGTTCTTTAAGGATCTCAAGGTGGATGACCAAATGAAACTACTCCAACATTCGTGGTCGGATATGCTGGTTCTGGATCACATGCACCAACGCATGCACAACAATCTTCCTGACGAAATGACCCTTCACAACGGTCAGAAGTTCGATTTGTTAAGTTTAGGACTCTTAGGCGTGCCCAGTTTAGCAGACCACTTCACTGACATCACCGCCAAACTGCAAGAACTCAAATTCGACGTTAGCGACTACATCTGTGTTAAATTTCTGTTGCTGCTTAACCCCGTATATTTTTTAGATGTGCGAGGAATCACAAACAAGAAACATGTACAAGAAGGTTACGAACAAGTTCAACAGGCTTTACTCGAATATACGGTGACGTGTTACCCACAAATCCAGGACAAGTTCAACAAGATGATGCAACTGCTCCCTGAGATTCACAGCTTGGCAACGCGCGGCGAAGAGCACCTCTACCACAAGCACTGCAGCGGTAGCGCGCCAACTCAGACGCTCCTGATGGAAATGCTCCACGCGAAAAGAAAATAACGGCCGTCCGCCTCTTCGTTTTCGTTACCATATCTGTGTGCAATACCAGCTTACAACACTAATGTACATAGGTTACTGGAAATCTCGTAGTTGTTGAAATTGACGATGCCTTCATGAAGTACAAAGAGCTAGGGCGGCCAGGCCGCACCGCCACCACATATTTTTATAAAATAGAGAAAAAGCGGCCACAGCACCCTGCTCCTCCGTTGTTTAATTTATGATAAATGTTACGTTGATTTAATGAGTTTTATAAGAACGACACACACTGACACACACACAAAACACATTCCAGATGTGCTCTGGAGGGATTCAAGGGCATCCTAGCGTAGGGTAGCGTAGCTTTTAAGCGCGAGTGACAAACAAACGAACGAAGCGAATGAGTGATTGGTTGGTTATTGCAGATCAAGTGCACTCTCTTCAACGTTTTAATCTTAGTACGTGTGGGGGTCGATTTGCCCCTTGTTAGCTTAACCAAGGTGATGGACTAACAGCTGTAATTTTATGTAATTATAGAGTTATACATACTATATATACACAGTTTATTACTAGTTATTAAGTTTAATTATTATATTATAAATATATTTGATTATGTTACATAATTATATGAATGTTAATATTATGTGAGAGCAAGGTACTGTGGTCTAACCTGTGTTATTGTTATCATTAAATTGGTAAATACTACTGATTGTTTGTGTTGTGCAGGCTACCTCTAAAGTCTCCTTCGCGTGGGGCCGCTGTGCGAGCCCCAATGCGCTGGTCAGCGGAGTCTATTTATTATCTGTTAGAGTATCTTGCCCTGCTGTGACATGCAAAAGGCCCTGAGCTTACTATTGATTGATCAGATATATATTTATATATAAAATACGTACACTTATTTAAGCAGTGTGATTGAGAAGATGTATATATAATTTATGTGGATCAAAGTGACTTGTTTGTAGAAATAAATCACAGTGAATTAAG

Read found in SRX6385151

READ ID: SRA:SRR9622227.11121124.1

Query 382 TTGTACTGTTTGTGAAATGTTACTGGACATGGAGAACCACTCGACGTTGTTATCTCTGAA 441

||||||||||||||||||||||||||||||||||||||||||||||||||||||||||||

Sbjct 76 TTGTACTGTTTGTGAAATGTTACTGGACATGGAGAACCACTCGACGTTGTTATCTCTGAA 17

Query 442 CATGTCCCCGTTCAA 456

|||||||||||||||

Sbjct 16 CATGTCCCCGTTCAA 2

##### >XM_008193151.2

TGTGTGTGTGTATGTATGACTTCACAACAGCACTAACATAGACCTGGTTCATGGGAAAGTGGGTCACCGGTCCCGAGTCACTTTCACTTATTACATGGGCTCCTAATCATTTTCCATTGTAACCCCGTGCTGCCAACACGTCTCTCGGAAGGGGATTCGCAGTTGAGGCGCTCTTGGACGGTCCGCGCTGGTCAGTTTGCTCACTGAAGTCCGTCGAGACGGAGGTGCCGTTGTTCCACTGGTGCGTGTGTGTTGTGTGCCTGCTCCACCGGATTACTGTGCTGTGCAGTGTGCTTGCTGATGCTCCACCATGCCCTTAAACTACTTCTCCTTCGTGGTGACCTAACCGAACGCTCGTCCCTCCGACCAGTCCTTACCCCGTTGTACTGTTTGTGAAATGTTACTGGACATGGAGAACCACTCGACGTTGTTATCTCTGAACATGTCCCCGTTCAACGCCAACTCGGGGGACTCGCCCGCCCAGGCCTCCAGCCCCATGCCCCAGTACGGCTCGCCCCCGGTGGCCTACAGCAGCTGCCAGCAGTCCATGGGGCTGCCGCACCACCAGCCCCCCATGATGATGTCCCAGAACATGAATAACCTAGACGCCTCTTATATCTTTTCTTCAGGGGCCAGCGCCATCTCCGGCGTCGACATGGGCGCCAGCTACCAGATTTCCGGGCCGGCGACGTCGCTCACAGCCGGCGACCTCCCAGACACGAAGGACGGCATCGAGGAGCTCTGTCCGGTCTGCGGGGATAAGGTCTCCGGGTACCACTACGGCCTCCTCACGTGTGAATCTTGCAAGGGATTTTTCAAACGAACTGTTCAAAATAAGAAAGTTTACACGTGCGTGGCGGAAAGAAGTTGTCACATAGACAAAACGCAAAGGAAAAGGTGTCCCTACTGCCGGTTTCAAAAGTGCTTAGAAGTCGGGATGAAACTTGAAGCTGTCCGAGCAGACCGAATGAGAGGCGGCAGGAACAAATTCGGTCCGATGTACAAACGCGACCGTGCCCGAAAACTGCAAATGATGCGACAACGACAGCTGGCGGCGCAAACGCTACGGGGAAGCTCTCTCGGTGACGCCATGTACAGCAGCCAGCCCGGAACATCACCCTTCGCCAACATCCACATCAAGCAAGAAATCCAAATTCCGCAAGTGTCATCTCTAACATCGTCGCCCGACTCGTCGCCGAGCCCCATCGCCGTCGCCCTGGGCCAGGTCAACACCTCCAACCTGGTGCAGCAGGCGTCCAGCCAGCAGCCGGCGCTGCAGATCGTCGGGGTGCCCGGGGGGCCCTCCTCCATGGTGATGGGGCCCGACAACAAGCTCTGGGGCTCCGCCAACTCCACCACCACCTCGCCCCACTCGCTCAGCCCCAAGGCGTTCCAGTTCGACACGGTGGTGCAAGGGGGCAGCGCGCCGCCCTCCAGTAAAGTGTCGCCGCTTATCAGGGACTTTGTGCAAGCCATCGACGATCGCGAGTGGCAAAACTCTTTGTATACTTTACTCCAGAACCAAACGTATAATCAGTGTGAAGTGGACTTATTTGAACTTATGTGTAAAGTTTTGGACCAAAATCTGTTCTCTCAAGTCGATTGGGCGAGGAATTCTGTGTTCTTTAAGGATCTCAAGGTGGATGACCAAATGAAACTACTCCAACATTCGTGGTCGGATATGCTGGTTCTGGATCACATGCACCAACGCATGCACAACAATCTTCCTGACGAAATGACCCTTCACAACGGTCAGAAGTTCGATTTGTTAAGTTTAGGACTCTTAGGCGTGCCCAGTTTAGCAGACCACTTCACTGACATCACCGCCAAACTGCAAGAACTCAAATTCGACGTTAGCGACTACATCTGTGTTAAATTTCTGTTGCTGCTTAACCCCGTATATTTTTTAGATGTGCGAGGAATCACAAACAAGAAACATGTACAAGAAGGTTACGAACAAGTTCAACAGGCTTTACTCGAATATACGGTGACGTGTTACCCACAAATCCAGGACAAGTTCAACAAGATGATGCAACTGCTCCCTGAGATTCACAGCTTGGCAACGCGCGGCGAAGAGCACCTCTACCACAAGCACTGCAGCGGTAGCGCGCCAACTCAGACGCTCCTGATGGAAATGCTCCACGCGAAAAGAAAATAACGGCCGTCCGCCTCTTCGTTTTCGTTACCATATCTGTGTGCAATACCAGCTTACAACACTAATGTACATAGGTTACTGGAAATCTCGTAGTTGTTGAAATTGACGATGCCTTCATGAAGTACAAAGAGCTAGGGCGGCCAGGCCGCACCGCCACCACATATTTTTATAAAATAGAGAAAAAGCGGCCACAGCACCCTGCTCCTCCGTTGTTTAATTTATGATAAATGTTACGTTGATTTAATGAGTTTTATAAGAACGACACACACTGACACACACACAAAACACATTCCAGATGTGCTCTGGAGGGATTCAAGGGCATCCTAGCGTAGGGTAGCGTAGCTTTTAAGCGCGAGTGACAAACAAACGAACGAAGCGAATGAGTGATTGGTTGGTTATTGCAGATCAAGTGCACTCTCTTCAACGTTTTAATCTTAGTACGTGTGGGGGTCGATTTGCCCCTTGTTAGCTTAACCAAGGTGATGGACTAACAGCTGTAATTTTATGTAATTATAGAGTTATACATACTATATATACACAGTTTATTACTAGTTATTAAGTTTAATTATTATATTATAAATATATTTGATTATGTTACATAATTATATGAATGTTAATATTATGTGAGAGCAAGGTACTGTGGTCTAACCTGTGTTATTGTTATCATTAAATTGGTAAATACTACTGATTGTTTGTGTTGTGCAGGCTACCTCTAAAGTCTCCTTCGCGTGGGGCCGCTGTGCGAGCCCCAATGCGCTGGTCAGCGGAGTCTATTTATTATCTGTTAGAGTATCTTGCCCTGCTGTGACATGCAAAAGGCCCTGAGCTTACTATTGATTGATCAGATATATATTTATATATAAAATACGTACACTTATTTAAGCAGTGTGATTGAGAAGATGTATATATAATTTATGTGGATCAAAGTGACTTGTTTGTAGAAATAAATCACAGTGAATTAAG

## Myriapoda

### Myriapoda (Strigamia maritima)


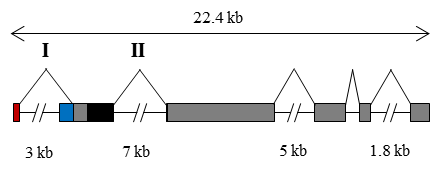


##### >Incomplete_alpha_myriapoda

GAAAGAGTTGTAGATTTGGAGCCAAGTAGTTTGCTGGAGAAAAGAGATCCTACTGCCGTCGCCGCTGCCGTGGCCGCCAGTGCAGCCGGGCCGCCGCCGCCGCCAACGCCGCCGCCTCCTCCGCAGCAGTCGACGTTGGTGATGTCCGAATACCAGGACCAGCCCGATACGAAAGAGGGCATCGAGGAACTGTGTCCCGTGTGCGGGGACAAAGTGTCCGGTTATCATTACGGGCTGCTCACGTGCGAGAGCTGTAAAGGTTTCTTCAAACGTACCGTACAGAATAAGAAGGTGTACACATGCGTCGCTGAAAGGAACTGCCATATTGATAAAAGTCAGAGGAAAAGATGTCCGTTTTGCAGGTTTCAAAAGTGTCTCGATGTTGGAATGAAATTAGAAGCTGTCCGTGCAGATCGTATGCGTGGCGGCCGAAACAAATTTGGACCGATGTACAAGAGGGACAGAGCGAGGAAACTACAACTCCTTCGACAGAAACAAATGCAGCAACACAATCGTTCAACCTGCCAACTCAACGCCGAGTTGTCGGGAGCTGGGGGTGGCAGTGGAAACAACGCCATGTCCACCTCGATGGCCAACAACATGGCATACGGACCTCCGTCGGGTCCACTGTACACTCAACAGGGGCTGCCAATAAAACAAGAGATACAAATTCCACAACTGAGTTCATCGACGGCCTCACCCGACTCGTCATCGTCGTCGCCGCTGCCCACCCAGAACCACCATCATGCCCATCATCATTCTCTACACCATCCACTCGTTATGGGCGGATCGATGAGCGCCCCTTCGCCGACGACCCTTCACTCTTCGCCGCCCAACTCGGCCGAACACCACAAAATGACAACGGCCTGGGTGGCGCCCGGCACGCCGACGTCCAGCACTCCGTCGCCGAACAACGGCAACGTGGTGGCGGCCACGAACGGCACGGGTTGTGGGGGCACGTCGGCCGCGTCGCCGTCGGACCCACATTCTCCGAAAGTGTTCCACTACGACGGGGCAGGTTCGGTGGTGCAGTTGAGTGCGGCGACTGCCAACGGGAATGGCAACGGCAACGGCACGACGCCGACAGCCACATCGATCGTGCCGGGCAAGATCCCGTCCATGATCCGGGACTTTCAGTACTCGTTGGACGACAAGGAATGGCAGAGTCAATTGTTTGGTTTGCTGCAAAGTCAAACGTACAATCAGTGCGAAGTGGACTTGTTCGAGTTGATGTGTAAAGTTTTGGACCAGTCGCTTTTTGCCCAAGTGGACTGGGCGCGGAACAGCACGTTTTTTAAGGATCTCAAAGTGGATGACCAGATGAAGTTGTTGCAGTACTCTTGGAGTGATATGCTCATCTTGGACCACATACATCACCGGCTGCACAATAGTTTACCTGAAGAAACGACGTTGGCTAACGGACAGAAATTCGACTTGCTCACGTTAGCCGTACTCGGACTGCCCAACTTGGTTGATCAGTTCACGGAATTAAACAGTAAAATGCAGGAATTGAAGCTGGACGGCAACGATTATGTGTGCCTCAAGTTCTTGCTGCTCCTCAATCCAGATGTACGGGGTTTGGTAAACCGGAAACACGTGCAAGAGGCCCATGAACAAGTAAAACAAGCACTCTTGGATTATTCTCTGAATTGCTACCAGCAAGATAAATTCAGCAAAATGCTTCGCCTGCTACCTGAAGTGCACATGTTGAGTTTACGCGGAGAAGAGTATCTTTATTACAAACACCTGAGTGGAAACGCACCTGTGCAGACACTCCTTATGGAAATGCTTCATGCAAAAAAAAATAAACTCAGACGAAGGTGAAATGAAAGGAAACGAGATGGAATTGAACCTCGGAAACGAAGAAATAACGGATCAATCCACTGCCCTCATTTTACCTTACAACTTCCATCCAATCGGCGAAGTTTGAAGGTCCTCTACACCCCGCAGAACTAAAAAGTGGCGAGTAGTGGAAGAAAGAATGAAGGAAGGCACTTTTTAAAATGGTTATGTCACACTAAACTCGTTGAAGATTCTTTTTGTTTTTGTTGATTTCTTCTGCTTTGAGGTGATGAATTCCATTGGTGCAAAACAGGAAATGGAAAATATTGAAAAATGGGCCCAAACTGCCATAACATTGTGTGACTATTTTGGTATTTTGATTATTGTTGTTTTTAACACCTGCTATTTTACACCTGGTGGTGCGATGAGCGAGCCAACCCCGCCCCTTGTTTAATTTTGTTTCTTCCCTTCATTTATGTACTGTTAAATTATCTTTTCATCATTTATTTTTATTTTTTTATTTTTCTTTGGAACAGATGTTTCAATTATGTTTTTTTAATTTGGTAATTAATTTTGAGAGAAAATTATTTTGTAGTACATTCCAATTTTGAGTTAAGTGTTACAAAATCCTTTCTATGTACAAAAAATTACATAAAAAAATAAGTTTTGTAGAAAAACAAAGGTTTTAAAAAAAAATATGATCATGATTAAGAAAATAAGCTTTTTTATATATTAATAAACAAAATACTATAACTTTTTGCCTGTATTATGATAAATTTAAAAACAAAAAAAGTATACTGTAGGTGATGGGAATTGAATTTATCTATATTAAAAAAAATAATAATAATAAACAAAGTATCCTTTTATGAGTTTTATGATGATATTTATTTAGTGTATAGGACCAGTTCATTTTTTTTTGTAAAAAATAATATTTTTTTAAAAAGGATAGTGAAATAGACAGGAATGATGAAAAAGTTGGACTTGCAAATACCTCACTGATAATCAGTGCAATGGATTTTTTTTCTTGTTTTTTTTTTTTTTTTTTTTTGTATTGAAAAATACAAAGTGTTCAAATCAAAATAACTGCACAACATGTGGCAATTTTTCTGCTTCTAAGCTATTATCTGAAATTTATGCTGCTTTAATTTCTACTTTTTCTGCTAACCATTACGGAGGTTGTTGAATTAGTTATTGAATTTACCACCAGACACTCACGAGGCAATTACACGTGGAAAATGCGAGGAAAAATGGCTGAAAATTGAAACATCGAGATCAAGTTAAGACCAATATAGAACAAATTGAATTAATAAGATTAAATTGGTTGATCTCATTACATAGTTTCTTGTAATTCGTCGATAATAATTCTTATTACTGTGTTTCTTGAAACGAAATGATGAAAGCGATTGACTTTGAGTATCTGGTGAGAGATTTGATAGGATGTTTACAATTTTTCAAAGTGTGTTGACACTTAACAGGTTTTCAAAAATGAAAGAACGGCAGGTGAATGGTGTTTATTTTTGTTTGTAATTGTCAAGAATTTATTTGCTACAAAAAGTGGCAGCTTTTGTGATTTTTTCTGTTTAAGTAATTAGTTCGTTTTGTTAGTGAGTTTAAAATTGGTTGTATTTTATTTTTCAAATATTTTTTAAAAATATTTTTTGTGTCAAGGTTGAATGATGTTTTCTAAATAGGAATTGGTATTATTACTGCCACTCATGCATTTCCTAGCTTTTATTTGTCTGTTTAGATAACTGTGTTTTTAAACAAACAAACAAAAATATTGAAAAGTTTACAAATTTTCAAGTTTAAAATTTTTGCAAACATTTTCTTTTCTTTGAGATATAAAAAAAGAGCAGGTTAAATCAAACTAAAACGTGTTTTGTTGACTAGAAACTACCGAAGTTAAATTTTTGCTTGGTTTTGTATGGATCAGTGGAATCCATTTGTCTGCCAAGAATGTTGGAAAACAAAAGAAATTAAAAAAGAACCAGTTATCCATTTGTTATTCTCTGCCACTGACTTATGCAATATAAACCACCTAGTACAAACATAGGAAATATTATTTAGGTACCAGAAACATAAATTTGATCATTTTCCCCTCATTTTTTCATATAACTCAAGGGCAATAATTGTTACTTAAAAAATAAATAGAAACTAGTCAATTTGAACTAGTGGCAATTTGACATAATTTTTTTAGTTTAATAAGAAGAAATAAATTTTTTTATTTTTAATAAAAATAATAATAATTGTTGAAATTTTAAAATTTTCAAAAAAATTGTTGGAAACATTACTAATGGCATTTGAAGCGTGATTAATAGTTTGTTACATTTTGTACCTTAAAAATAAATGCAATAATTCCTTCATGACAACAACATGACATTAATTAATGTTAAATTCTCAGAAAAATTTAATTATTTGAATGTTTAAAAGTGTCGTAACGACATGAATGAATTATTCCATGTATTTTCAAGGTAGAGTGAAATATTTTAGCAAAAAAAGTGATTTAACATTCCATCAAGGTTTGTGTCTCTGCTTAAACTTTGTAACCACCACGTTCTTTAACTGTTTTCTCTCTTATATTAACCGGCGGACAAATAAATGTTAACATTCCAGCGTTGGTTATTTGTTTTGCTACTTGAGTCTTCCCTCAAACTTATACTAATCATAAAACAAAAACTGTTTTTAATTTGTTTAACTCATCTTAACTAATTCACTCGTTTAATTCACCTCCTAACTGAGCAGTTGGTGCCACTGAATTTTGAACTGTAAAAATGAAAAATATATATTTAAAAGTTTTTAAAAAAAACAAAAAAAAAACTTGTTCTCTACAAAGGTTGTAAAATGTTTTCAGAAGTTTTAATCACAGATGGGGATTTATAAGTGTATTGTACAAGAGTTATACTTTTTCTATAGTTAATAGCACCACATTAATATTTGTATTAGAAAAAAAATGTTGCCTTCATTATTTGAAGTTGCTTGTGTCCATTGTACATAAATAAGTTCTTAGCTCTTTTTCAGTTGCTAACTATTGATATTTGAGTCTGACGTTTAATATTTTTTTAAGGAAAATGACGAGTCTATGAAAGAACAAAATGTTTTACTGCACAGATTCGGTTCTATGTAAACAATTATTGTTTGAAAATTATTACACGTAATTTGTTAATTTTTTTTATATATAAATGAGGTGACTGTTGTGTTTTTGTATAGATTTAAAAATGAAAACTGATTAGGATTTGTAGTTTGTTTTTAACCTTTTTTTTAAAAAAAATACAGTGTTATTCCAGGCAGAAATAAATACACTCCCTCCCAAAGGGGAATTTATTTATTTCTTTAAACACACAAAAAAGAGGAAAATAATCATGTATACCAGTAAATATATACTGTAGAAAAAACACTACTGTGAATATTGAGAAACAAATTTTATCACCAGATTTCATAGCATTGCAACTGATTCAAACTCTCTTTTATTAGTTTTAAGTGATATAAATAAATAAATCAATGTTTCTGATTTTTCACATTTTAATTAACCCTTTGAGACCCAAAAATGCCTTAATATTTTTAGTTAAATAACAAATATTTAGTCACAAAATCTTTTTGATCTCAAAGGGTCATGCAGCTAATAAAAAGCTTGTTCATTTATGATTTGAACAGTTTTTATCTTAATTAACTAATACACATACAAATCCTTGTGCCAACAAGCTTTGCTCTAATTGTATAATATTTTT

Found read in SRX4183681 male gonad RNAseq

READ ID: SRA:SRR7280106.135368945.2

Query 417 TGTGTGTGGTGCCGGCGCCGCCGCCACTGTCCCCTTACTCGGGCGGCCCCGTGCTCAGCC 476

||||||||||||||||||||||||||||||||||||||||||||||||||||||||||||

Sbjct 75 TGTGTGTGGTGCCGGCGCCGCCGCCACTGTCCCCTTACTCGGGCGGCCCCGTGCTCAGCC 16

Query 477 CGTCGGAAATCTCGA 491

|||||||||||||||

Sbjct 15 CGTCGGAAATCTCGA 1

##### >Strigamia_maritima_bFTZF1

GGACCGATCCACGATGTCGATTTTTAGAGGCAACCAAAAAAATGTACAGTTACAATCCTTTTTACCCCTTTGCTGAACGACTCGGGACGAGTTTGAAACGTCCCAGCGCGCACACGAGAATTTAGGCTTAAGCGAGTTTGTTGTAAAGTTTTACATCCCGGGTGTTTGTTGCTAGGTTGGCGGATCCCTCGCACTAAGCGTCACACTGCAAAGTGGCTGAGTGAGTAGAGTGTGAGTGGTAAACTCTTCTCGACTCGACCCAATCTAGGTCTATGTTAGAGAGAAAGCTTACCACGCACAGCTCTCACTATCAGTCCTACTGACCTACATTCCCACCACTGTGCAGAGAGAGAAACCAGCGAGGGAGTGGAGAGGGAGAACAACGGGGAGGATGGTGGCAGCATGGAAAAGCCAAATGTGTGTGGTGCCGGCGCCGCCGCCACTGTCCCCTTACTCGGGCGGCCCCGTGCTCAGCCCGTCGGAAATCTCGACCACTTTTCTCATGGATACTTCCACTTATTTCTTACCAGCTGCCGTCGCCGCTGCCGTGGCCGCCAGTGCAGCCGGGCCGCCGCCGCCGCCAACGCCGCCGCCTCCTCCGCAGCAGTCGACGTTGGTGATGTCCGAATACCAGGACCAGCCCGATACGAAAGAGGGCATCGAGGAACTGTGTCCCGTGTGCGGGGACAAAGTGTCCGGTTATCATTACGGGCTGCTCACGTGCGAGAGCTGTAAAGGTTTCTTCAAACGTACCGTACAGAATAAGAAGGTGTACACATGCGTCGCTGAAAGGAACTGCCATATTGATAAAAGTCAGAGGAAAAGATGTCCGTTTTGCAGGTTTCAAAAGTGTCTCGATGTTGGAATGAAATTAGAAGCTGTCCGTGCAGATCGTATGCGTGGCGGCCGAAACAAATTTGGACCGATGTACAAGAGGGACAGAGCGAGGAAACTACAACTCCTTCGACAGAAACAAATGCAGCAACACAATCGTTCAACCTGCCAACTCAACGCCGAGTTGTCGGGAGCTGGGGGTGGCAGTGGAAACAACGCCATGTCCACCTCGATGGCCAACAACATGGCATACGGACCTCCGTCGGGTCCACTGTACACTCAACAGGGGCTGCCAATAAAACAAGAGATACAAATTCCACAACTGAGTTCATCGACGGCCTCACCCGACTCGTCATCGTCGTCGCCGCTGCCCACCCAGAACCACCATCATGCCCATCATCATTCTCTACACCATCCACTCGTTATGGGCGGATCGATGAGCGCCCCTTCGCCGACGACCCTTCACTCTTCGCCGCCCAACTCGGCCGAACACCACAAAATGACAACGGCCTGGGTGGCGCCCGGCACGCCGACGTCCAGCACTCCGTCGCCGAACAACGGCAACGTGGTGGCGGCCACGAACGGCACGGGTTGTGGGGGCACGTCGGCCGCGTCGCCGTCGGACCCACATTCTCCGAAAGTGTTCCACTACGACGGGGCAGGTTCGGTGGTGCAGTTGAGTGCGGCGACTGCCAACGGGAATGGCAACGGCAACGGCACGACGCCGACAGCCACATCGATCGTGCCGGGCAAGATCCCGTCCATGATCCGGGACTTTCAGTACTCGTTGGACGACAAGGAATGGCAGAGTCAATTGTTTGGTTTGCTGCAAAGTCAAACGTACAATCAGTGCGAAGTGGACTTGTTCGAGTTGATGTGTAAAGTTTTGGACCAGTCGCTTTTTGCCCAAGTGGACTGGGCGCGGAACAGCACGTTTTTTAAGGATCTCAAAGTGGATGACCAGATGAAGTTGTTGCAGTACTCTTGGAGTGATATGCTCATCTTGGACCACATACATCACCGGCTGCACAATAGTTTACCTGAAGAAACGACGTTGGCTAACGGACAGAAATTCGACTTGCTCACGTTAGCCGTACTCGGACTGCCCAACTTGGTTGATCAGTTCACGGAATTAAACAGTAAAATGCAGGAATTGAAGCTGGACGGCAACGATTATGTGTGCCTCAAGTTCTTGCTGCTCCTCAATCCAGATGTACGGGGTTTGGTAAACCGGAAACACGTGCAAGAGGCCCATGAACAAGTAAAACAAGCACTCTTGGATTATTCTCTGAATTGCTACCAGCAAGATAAATTCAGCAAAATGCTTCGCCTGCTACCTGAAGTGCACATGTTGAGTTTACGCGGAGAAGAGTATCTTTATTACAAACACCTGAGTGGAAACGCACCTGTGCAGACACTCCTTATGGAAATGCTTCATGCAAAAAAAAATAAACTCAGACGAAGGTGAAATGAAAGGAAACGAGATGGAATTGAACCTCGGAAACGAAGAAATAACGGATCAATCCACTGCCCTCATTTTACCTTACAACTTCCATCCAATCGGCGAAGTTTGAAGGTCCTCTACACCCCGCAGAACTAAAAAGTGGCGAGTAGTGGAAGAAAGAATGAAGGAAGGCACTTTTTAAAATGGTTATGTCACACTAAACTCGTTGAAGATTCTTTTTGTTTTTGTTGATTTCTTCTGCTTTGAGGTGATGAATTCCATTGGTGCAAAACAGGAAATGGAAAATATTGAAAAATGGGCCCAAACTGCCATAACATTGTGTGACTATTTTGGTATTTTGATTATTGTTGTTTTTAACACCTGCTATTTTACACCTGGTGGTGCGATGAGCGAGCCAACCCCGCCCCTTGTTTAATTTTGTTTCTTCCCTTCATTTATGTACTGTTAAATTATCTTTTCATCATTTATTTTTATTTTTTTATTTTTCTTTGGAACAGATGTTTCAATTATGTTTTTTTAATTTGGTAATTAATTTTGAGAGAAAATTATTTTGTAGTACATTCCAATTTTGAGTTAAGTGTTACAAAATCCTTTCTATGTACAAAAAATTACATAAAAAAATAAGTTTTGTAGAAAAACAAAGGTTTTAAAAAAAAATATGATCATGATTAAGAAAATAAGCTTTTTTATATATTAATAAACAAAATACTATAACTTTTTGCCTGTATTATGATAAATTTAAAAACAAAAAAAGTATACTGTAGGTGATGGGAATTGAATTTATCTATATTAAAAAAAATAATAATAATAAACAAAGTATCCTTTTATGAGTTTTATGATGATATTTATTTAGTGTATAGGACCAGTTCATTTTTTTTTGTAAAAAATAATATTTTTTTAAAAAGGATAGTGAAATAGACAGGAATGATGAAAAAGTTGGACTTGCAAATACCTCACTGATAATCAGTGCAATGGATTTTTTTTCTTGTTTTTTTTTTTTTTTTTTTTTGTATTGAAAAATACAAAGTGTTCAAATCAAAATAACTGCACAACATGTGGCAATTTTTCTGCTTCTAAGCTATTATCTGAAATTTATGCTGCTTTAATTTCTACTTTTTCTGCTAACCATTACGGAGGTTGTTGAATTAGTTATTGAATTTACCACCAGACACTCACGAGGCAATTACACGTGGAAAATGCGAGGAAAAATGGCTGAAAATTGAAACATCGAGATCAAGTTAAGACCAATATAGAACAAATTGAATTAATAAGATTAAATTGGTTGATCTCATTACATAGTTTCTTGTAATTCGTCGATAATAATTCTTATTACTGTGTTTCTTGAAACGAAATGATGAAAGCGATTGACTTTGAGTATCTGGTGAGAGATTTGATAGGATGTTTACAATTTTTCAAAGTGTGTTGACACTTAACAGGTTTTCAAAAATGAAAGAACGGCAGGTGAATGGTGTTTATTTTTGTTTGTAATTGTCAAGAATTTATTTGCTACAAAAAGTGGCAGCTTTTGTGATTTTTTCTGTTTAAGTAATTAGTTCGTTTTGTTAGTGAGTTTAAAATTGGTTGTATTTTATTTTTCAAATATTTTTTAAAAATATTTTTTGTGTCAAGGTTGAATGATGTTTTCTAAATAGGAATTGGTATTATTACTGCCACTCATGCATTTCCTAGCTTTTATTTGTCTGTTTAGATAACTGTGTTTTTAAACAAACAAACAAAAATATTGAAAAGTTTACAAATTTTCAAGTTTAAAATTTTTGCAAACATTTTCTTTTCTTTGAGATATAAAAAAAGAGCAGGTTAAATCAAACTAAAACGTGTTTTGTTGACTAGAAACTACCGAAGTTAAATTTTTGCTTGGTTTTGTATGGATCAGTGGAATCCATTTGTCTGCCAAGAATGTTGGAAAACAAAAGAAATTAAAAAAGAACCAGTTATCCATTTGTTATTCTCTGCCACTGACTTATGCAATATAAACCACCTAGTACAAACATAGGAAATATTATTTAGGTACCAGAAACATAAATTTGATCATTTTCCCCTCATTTTTTCATATAACTCAAGGGCAATAATTGTTACTTAAAAAATAAATAGAAACTAGTCAATTTGAACTAGTGGCAATTTGACATAATTTTTTTAGTTTAATAAGAAGAAATAAATTTTTTTATTTTTAATAAAAATAATAATAATTGTTGAAATTTTAAAATTTTCAAAAAAATTGTTGGAAACATTACTAATGGCATTTGAAGCGTGATTAATAGTTTGTTACATTTTGTACCTTAAAAATAAATGCAATAATTCCTTCATGACAACAACATGACATTAATTAATGTTAAATTCTCAGAAAAATTTAATTATTTGAATGTTTAAAAGTGTCGTAACGACATGAATGAATTATTCCATGTATTTTCAAGGTAGAGTGAAATATTTTAGCAAAAAAAGTGATTTAACATTCCATCAAGGTTTGTGTCTCTGCTTAAACTTTGTAACCACCACGTTCTTTAACTGTTTTCTCTCTTATATTAACCGGCGGACAAATAAATGTTAACATTCCAGCGTTGGTTATTTGTTTTGCTACTTGAGTCTTCCCTCAAACTTATACTAATCATAAAACAAAAACTGTTTTTAATTTGTTTAACTCATCTTAACTAATTCACTCGTTTAATTCACCTCCTAACTGAGCAGTTGGTGCCACTGAATTTTGAACTGTAAAAATGAAAAATATATATTTAAAAGTTTTTAAAAAAAACAAAAAAAAAACTTGTTCTCTACAAAGGTTGTAAAATGTTTTCAGAAGTTTTAATCACAGATGGGGATTTATAAGTGTATTGTACAAGAGTTATACTTTTTCTATAGTTAATAGCACCACATTAATATTTGTATTAGAAAAAAAATGTTGCCTTCATTATTTGAAGTTGCTTGTGTCCATTGTACATAAATAAGTTCTTAGCTCTTTTTCAGTTGCTAACTATTGATATTTGAGTCTGACGTTTAATATTTTTTTAAGGAAAATGACGAGTCTATGAAAGAACAAAATGTTTTACTGCACAGATTCGGTTCTATGTAAACAATTATTGTTTGAAAATTATTACACGTAATTTGTTAATTTTTTTTATATATAAATGAGGTGACTGTTGTGTTTTTGTATAGATTTAAAAATGAAAACTGATTAGGATTTGTAGTTTGTTTTTAACCTTTTTTTTAAAAAAAATACAGTGTTATTCCAGGCAGAAATAAATACACTCCCTCCCAAAGGGGAATTTATTTATTTCTTTAAACACACAAAAAAGAGGAAAATAATCATGTATACCAGTAAATATATACTGTAGAAAAAACACTACTGTGAATATTGAGAAACAAATTTTATCACCAGATTTCATAGCATTGCAACTGATTCAAACTCTCTTTTATTAGTTTTAAGTGATATAAATAAATAAATCAATGTTTCTGATTTTTCACATTTTAATTAACCCTTTGAGACCCAAAAATGCCTTAATATTTTTAGTTAAATAACAAATATTTAGTCACAAAATCTTTTTGATCTCAAAGGGTCATGCAGCTAATAAAAAGCTTGTTCATTTATGATTTGAACAGTTTTTATCTTAATTAACTAATACACATACAAATCCTTGTGCCAACAAGCTTTGCTCTAATTGTATAATATTTTT

## Chelicerata

### Acariformes (Tetranychus urticae)


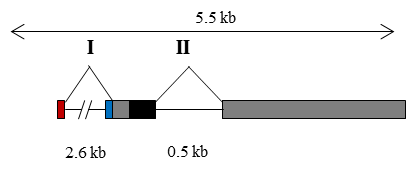


Reads found SRX7472641

Read ID: SRA:SRR10798718.21843491.2

Query 254 caacataaatacactgatatataaatatatatTACAACAGCATTAAACCCGTCCAATTAA 313

||||||||||||||||||||||||||||||||||||||||||||||||||||||||||||

Sbjct 1 CAACATAAATACACTGATATATAAATATATATTACAACAGCATTAAACCCGTCCAATTAA 60

Query 314 AGGAAACCTTGTCAACCCTATCAGATCCTAAAATGGCCCAG 354

|||||||||||||||||||||||||||||||||||||||||

Sbjct 61 AGGAAACCTTGTCAACCCTATCAGATCCTAAAATGGCCCAG 101

##### >XM_015929659.2

GTTTAGCGAGACCAGAGTTGAACATCTATTTTTTTTTCGTTCATAAAGTTCATATTTTAAACTAATCTATTGTATTATATTAGTGTTGTAATTTACTGTAATCTACTGTAATCTACTGTATTCTACTATACCGAATGTAACCTGTGTAATTGTTAACTCTATTGAATAGTGTTTATCATCTATATATACCTGTGCTATTAACGAGACCAACCATTAAATTATCAAACAGGAGATATATTTATATATACACAGACAACATAAATACACTGATATATAAATATATATTACAACAGCATTAAACCCGTCCAATTAAAGGAAACCTTGTCAACCCTATCAGATCCTAAAATGGCCCAGAATGATTTGGTGTCTTCTGGGTCTTCTTCAAATGAACCAAATGGACCTACCGGTTATGGACATAATCATTTACCTTCTGCTTTATCCTTGGCAGTCCCATCGATAACCAGTGCTGCTGGTCCACACTGTCCACCTGAAACACCTGATACCAAGGAAGGTATGGATGAATTGTGTCCCGTGTGTGGGGATAAAGTTTCAGGTTACCATTATGGACTATTAACCTGTGAATCCTGTAAAGGTTTCTTCAAACGGACTGTACAAAATAAAAAGGTTTATACCTGTGTTGCTGATCGTAGTTGTCACATTGACAAATCCCAGAGGAAACGATGTCCATTTTGTAGATTTCAAAAGTGCCTTGAAGTTGGAATGAAATTAGAAGCTGTACGTGCTGATCGTATGAGAGGAGGTCGTAATAAGTTTGGGCCAATGTACAAAAGAGATCGAGCTCGACGATTACAGTTGATGAGGCAACGTCAAATGGTGCGTGGCGGTTCACTTGGTGGTCATTCGGGTAACCATAGTCCTGGTGAAGTTGGTGGTTCACCATCACTTGCCATGGTAGCCTCCAACAGTAACGGTGGACTGGGAGGTGGTTCAATTTACACACCGGACGGGATCAAGCAAGAACTTATTCAAATTCCTCAACTATCATCATCAACCTCATCCCCCGACTCTTCACCAACCTCTGTACCTACCACTCTTACATCATCAGTTTTTTCTGTCCCAGGTCATCATTTTGGTTCATCCTCATCCCAAGGACATTCAGTCTCACATCCTGGCTCCATGGGTGGTGCCTCAAGTAACCCTGGAGGTCCAGCCGGTGGTGGATCACATGTTGACATGGTTAAATGGGTTCCTAATTCAAATGGTTCCCTTGCACCAACAGGATTACCCAAATCAGCTACTGGTAACTCATCATCCTCCTCGACTACCTCATCAACATCCTCACCATCCGGTCACAATGGTCCTGGAGGTCCAGGTGGCGGCGGTGGTGGCGGCGGTGGTAGTGGTGGTATCAATAATGGTACCGCAAATGGACCAAACAATGGATTACCATTCCATTATGGTAGTGGTCATGGTCCAGGAGGTAGTGAAAGTGGCGGCCAAGGGTCTTTAATGAACAATAATGGTACAAATAATGAAACAAGTGGCTCAGGTTTACTTGGTTTATCTCTTAATTCATCGTCAACTGGTCACGGCGGTGGTGGTCGTGATAAGATTCCCATAATTGTTCGTGAACTTCAAGCAACAGCTCCAGATGATAATGAATGGAGAAGCCAATTATTTGGACTTCTTAACAGTCAAACGTATAACCAATGTGAAGTTGATCTATTTGAATTGATGTGCAAAGTTATTGATCAATCACTTTTTGCTCAAGTCGATTGGGCAAGGAATAGTATTTTCTTTAAAGATCTAAAAGTGGACGATCAAATGAAACTGTTACAACACGCATGGAGTGATATGCTTGTACTAGACCACATTCATCAAAGGATGCACAATGATTTACCCGATGAAACAACATTACCCAATGGGCAAAAGTTTGATCTCTTAGGTTTAGCTTTGCTCGGTGTTCCAACCGCATCTGATAATTTGATTCAATGTCAAGCAAAATTACAGCAACTCAAATTTGATTCAGTTGACTATCTTTGTGTCAAATTTTTGTTACTCTTAAATCCTGAAGTACGAGGTCTATCAAATTATAAATTAGTTGCTGAAGCTCATGAGCAAACCCAACAAGCTCTACACCAATATTGTCTTGAAATATATCCTCAAATTAACGATAAATTTCACCAATTAATGGCACAATTACCCGCACTAAGACAATTAACAATACGAGGAGAGGAATTCCTTTATTATAAACACCTTAACGGAGACGCTCCATCGCAAACCTTATTAATGGAAATGCTTCACGCAAAACGAAAATAA

Reads found SRX7472641

Read ID: SRA:SRR10798718.23343510.2

Query 6 GATGTCCAATCTTAGTGATCATCACATTGAACCATATTTTATGCCACCAACAGGGTCTTC 65

||||||||||||||||||||||||||||||||||||||||||||||||||||||||||||

Sbjct 101 GATGTCCAATCTTAGTGATCATCACATTGAACCATATTTTATGCCACCAACAGGGTCTTC 42

Query 66 TTCAAATGAACCAAATGGACCTACCGGTTATGGACATAATC 106

|||||||||||||||||||||||||||||||||||||||||

Sbjct 41 TTCAAATGAACCAAATGGACCTACCGGTTATGGACATAATC 1

>Tetranychus_urticae_bFTZF1

ATGATGATGTCCAATCTTAGTGATCATCACATTGAACCATATTTTATGCCACCAACAGGGTCTTCTTCAAATGAACCAAATGGACCTACCGGTTATGGACATAATCATTTACCTTCTGCTTTATCCTTGGCAGTCCCATCGATAACCAGTGCTGCTGGTCCACACTGTCCACCTGAAACACCTGATACCAAGGAAGGTATGGATGAATTGTGTCCCGTGTGTGGGGATAAAGTTTCAGGTTACCATTATGGACTATTAACCTGTGAATCCTGTAAAGGTTTCTTCAAACGGACTGTACAAAATAAAAAGGTTTATACCTGTGTTGCTGATCGTAGTTGTCACATTGACAAATCCCAGAGGAAACGATGTCCATTTTGTAGATTTCAAAAGTGCCTTGAAGTTGGAATGAAATTAGAAGCTGTACGTGCTGATCGTATGAGAGGAGGTCGTAATAAGTTTGGGCCAATGTACAAAAGAGATCGAGCTCGACGATTACAGTTGATGAGGCAACGTCAAATGGTGCGTGGCGGTTCACTTGGTGGTCATTCGGGTAACCATAGTCCTGGTGAAGTTGGTGGTTCACCATCACTTGCCATGGTAGCCTCCAACAGTAACGGTGGACTGGGAGGTGGTTCAATTTACACACCGGACGGGATCAAGCAAGAACTTATTCAAATTCCTCAACTATCATCATCAACCTCATCCCCCGACTCTTCACCAACCTCTGTACCTACCACTCTTACATCATCAGTTTTTTCTGTCCCAGGTCATCATTTTGGTTCATCCTCATCCCAAGGACATTCAGTCTCACATCCTGGCTCCATGGGTGGTGCCTCAAGTAACCCTGGAGGTCCAGCCGGTGGTGGATCACATGTTGACATGGTTAAATGGGTTCCTAATTCAAATGGTTCCCTTGCACCAACAGGATTACCCAAATCAGCTACTGGTAACTCATCATCCTCCTCGACTACCTCATCAACATCCTCACCATCCGGTCACAATGGTCCTGGAGGTCCAGGTGGCGGCGGTGGTGGCGGCGGTGGTAGTGGTGGTATCAATAATGGTACCGCAAATGGACCAAACAATGGATTACCATTCCATTATGGTAGTGGTCATGGTCCAGGAGGTAGTGAAAGTGGCGGCCAAGGGTCTTTAATGAACAATAATGGTACAAATAATGAAACAAGTGGCTCAGGTTTACTTGGTTTATCTCTTAATTCATCGTCAACTGGTCACGGCGGTGGTGGTCGTGATAAGATTCCCATAATTGTTCGTGAACTTCAAGCAACAGCTCCAGATGATAATGAATGGAGAAGCCAATTATTTGGACTTCTTAACAGTCAAACGTATAACCAATGTGAAGTTGATCTATTTGAATTGATGTGCAAAGTTATTGATCAATCACTTTTTGCTCAAGTCGATTGGGCAAGGAATAGTATTTTCTTTAAAGATCTAAAAGTGGACGATCAAATGAAACTGTTACAACACGCATGGAGTGATATGCTTGTACTAGACCACATTCATCAAAGGATGCACAATGATTTACCCGATGAAACAACATTACCCAATGGGCAAAAGTTTGATCTCTTAGGTTTAGCTTTGCTCGGTGTTCCAACCGCATCTGATAATTTGATTCAATGTCAAGCAAAATTACAGCAACTCAAATTTGATTCAGTTGACTATCTTTGTGTCAAATTTTTGTTACTCTTAAATCCTGAAGTACGAGGTCTATCAAATTATAAATTAGTTGCTGAAGCTCATGAGCAAACCCAACAAGCTCTACACCAATATTGTCTTGAAATATATCCTCAAATTAACGATAAATTTCACCAATTAATGGCACAATTACCCGCACTAAGACAATTAACAATACGAGGAGAGGAATTCCTTTATTATAAACACCTTAACGGAGACGCTCCATCGCAAACCTTATTAATGGAAATGCTTCACGCAAAACGAAAATAA

### Araneae (Parasteatoda tepidarorum)


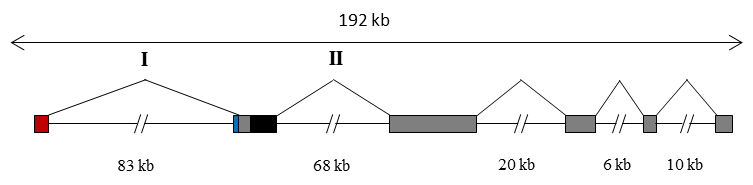


Confirmed reads SRX2855144 Male soma sample

Read ID:  SRA:SRR5602551.4827374.2

Query 592 GATGTTAGTTGACGGTGGACCCAATGTATCGTCCGCCAGCGATATGAACAGCGACTCGAC 651

||||||||||||||||||||||||||||||||||||||||||||||||||||||||||||

Sbjct 1 GATGTTAGTTGACGGTGGACCCAATGTATCGTCCGCCAGCGATATGAACAGCGACTCGAC 60

Query 652 CACCACCACGGTGGCGGCCGTGACACAAGGGCAAGATGACCATTCCCTTGAAATTACAGG 711

||||||||||||||||||||||||||||||||||||||||||||||||||||||||||||

Sbjct 61 CACCACCACGGTGGCGGCCGTGACACAAGGGCAAGATGACCATTCCCTTGAAATTACAGG 120

Query 712 GCCCT 716

|||||

Sbjct 121 GCCCT 125

##### >XM_016053025.2

TAGCGTTACCACGTGACTTCTGTGTTTATTGTCGGCAGTTACATCAACGGCGGCGGTTCGTGTAGTTAGGTTCCTTCTTCTATAATACTTTTCTTGCCTTTTGTTTTTAAAACATCCTATCCCTTTCTCTAACTGCTTGTAGAAGACTTGTGTGATTATGTCATAATGTGTACGTTTCATCATCGAGGGATTTCGTGTTATCACTAGCGAAAGCGACTTGGTAACACTAGTGACCTACTTTCGAGAAAGCTCGCCGTTTTAACCCACTTTTCTGCGCCTGCTCACTGCGTCTGAAAGTGAAGTTTTTTCAGGGCTGGCTTTACCTACCCTAAAACGCTACGGAAGGAAGAACGCTAAGCCTTGTCCTTGATCTCCACCGGCTATTCTGACCTGTGGTTCACAAAGTTGCGAAAATAAAGAAACGCAAAGATATTTTGTTGTGTGAAGGTTGACCGCTACGATTATTGATGTTAACGATTTCTGATGCATCTTATGGATGATCGTAATTGCTTCCGCGAGCAATAATTGCTGCCGGGTGGTGTTTTGTTATAGAAAGTGAATCGTGATCGGCCCAGGCGTGGAACGGGAAGTGATGTTAGTTGACGGTGGACCCAATGTATCGTCCGCCAGCGATATGAACAGCGACTCGACCACCACCACGGTGGCGGCCGTGACACAAGGGCAAGATGACCATTCCCTTGAAATTACAGGGCCCTCCAACATGCTCTCTGTGGTGCCCTCGCAAGTGGGGTCCCCGCAACAACAGTCCAGTGGCCCCCTGGTTGAGTATTCACATGACCTGCCGGACACCAAGGAGGGTATTGAGGAACTGTGCCCTGTTTGTGGGGACAAAGTGTCCGGATACCACTATGGTCTGCTCACGTGCGAGTCTTGCAAAGGATTTTTCAAGAGAACGGTACAGAATAAAAAAGTTTACACCTGCGTGGCCGACCGCAGTTGTCACATTGATAAAAGTCAAAGGAAAAGATGTCCCTTCTGCCGATTTCAAAAATGTCTGGACGTTGGAATGAAACTTGAAGCTGTACGAGCAGATCGAATGAGAGGAGGTAGGAACAAATTCGGACCTATGTACAAACGTGACAGAGCACGAAAGCTACAAATGATGAGACAAAAACAAATCCAGAGAGCCCAGTGTCTCGGTATCATGACCAACAACGAGGTCACCCCAGCCTTGCCTCCACCATTCGCACCCCCAGCAATGCCATCACCATCAGTCTACGACCCCCATCACATCAAACAAGAACTCATCCAAATACCCCAACTGAGCTCATCCACCAGCTCACCGGACTCATCTCCCTCTCCCCTCTGTACCTCAGCTCAGACCAACTCAACGATGGCGGCTCATTTTGCCCTGGCCGGTGGACCACCAGTCCTGTCTACCGTGCCAGAGCCAATGAAATCATGGCCTTCATCCACCAGGGCACCATCACCACCCCATCCGACTGGGTCGAATGGTGGTGCATCCTCTGGTTCACCCAAACCACCTTATCACTACGGAGACGTGAGTCCTGTACCACTTACAGTAAGAAGTCCATCACCTATTCTTATCGGAAGTGGTAAGATCCCTCACCTAATCAAAGATTTTCAGACGTCCATGCCCGACGACAAAGATTGGCAGTCTCAACTCTTTGGTCTTCTTCAAAATCAAACATACAACCAGTGTGAGGTGGATCTTTTTGAACTTATGTGTAAAGTTATTGACCAATCACTTTTCGCGCAAGTTGATTGGGCCCGCAATAGTGTCTTCTTCAAGGAACTGAAGGTGGATGATCAAATGAAACTGCTGCAGCACTCTTGGAGTGATATGTTGGTTTTAGATCATCTTCATCAGAGGCTTCACAACGGCCTTCCTGAAGAGATAACAATGCCCAATGGTCAAAAGTTCAGTATGGTTAATTTGGCTCTACTAGGGGTAGCTAGTATGACAGACCAGTTAAATCATGTGTCAGCCCGGTTACAAGAACTCAAATTTGATCCCCTAGATTATATATGTCTCAAGTTTCTACTACTACTCAATCCTGCAGAAGTAAGAACACTGGCGAATGTCAAGTTGGTGATTGATGCATATGAACGAACACAACAAGCCCTGGTTGAATATACACTCAGCTGTTACCCACAATTTCCAGATAAATTTAACAACTTAATGTCAATACTGCCAGAAATCCATGTGATCAGTGAACGGGGGGAAGATTTTCTACTCTACCGACATCTAAACGGAAGTGCACCCTCACAGACTCTACTCATGGAAATGTTACATGCGAAGAAAAAATAGCAGGACTTGAAAAATTTTGTTGTCTTTATATGTTGATAATATTTACAGTTGTAACTGCCATCAGTTCAATTCTTTCTATAACTGCCAGAAAGACTTTCGGATCTAACTCTGTACATTCCATTAGGAGGGGTCAGCCCACCCCTCAACCCCCTAAAGGGATGTTGGAATTTTTGAGATCCTCGCTAAAATGAACTTGATACCATTTGATGTTGTTATTCTCTAAGCACACAATTCTCTTGTACATTTAAAAATTGAAAGACTTCATGCATTCTACGTCAACTAACAAAGCCATTGTCCTGTTTTAAGAAAAATCATCTGTTCATTGTTATTGCTCCCTCCCCTTCTGTAGAAATTTTATTTCCTTATATTTGTACAGTTGTAAGAGGGAAGCTGTTTTTTTTCTTCTCTTTTTTGTGGTTCAAACTTGTTTATAATTATTAAGAAGAAGTGAAATATAAAAGCATTTGGGAGAGAGATGATTTCATTTAAATAGTTTAAATTATACCATAACTAACTGTTCTTAAACAAAACTCTTAATAATGTAGAGTAGACTCTTAAAAGTTTAAACTGTTCATTTTGTTAACCTCATGTATCTCAGGGAATTGATTGAATGTAATTTTTTTCTCCTTTTTTTAGAGTATTATAAGCAATAAGAATCTTTATTTCATTTCTTTATAGTGGCACAGTTGGTTTTGATTTTATTAGAATATCCTACAAAAGGACAAAATTATTTTTTAAATCTTTTATTAATTAATTATTATCTTTAAAAATATTACAATTTATAATATAAGCTTTCTGCAATTTTTTTATTTCTAAAAAAAATTTACCTTTTATTATATGTTCTATACTTTTTCCTTTACTGCTATAGACAAATTCTACTGCTTGTGAACTAGTCAGATATTTTTTATATATTATTATGTGTATAATTTTTCAAAATTTTGTAAATAAAATTATTTAAGCGTATCATATTTGTATTTTGTATATATTGTAATTTTTTTTTTTGCAACTTTTGAATGATAAGAAAAAATTATGTATCCCAACTGTGTCACACTCCAAAAAATTGTTTGCCCTAATTGAATTTTGACTGATTCACTATTATTTCAAAATCACCAGTGTTTATGATTCACTACTGCAAAATATGAGTTTTCAAAATGTAATTTTTCATTTTTTTTCTTCAAAATTGTGATTTGTTTTTGTTTGTAGATAACACATTCCTCTGTTCAATTTCTTCCCTATGCATTGTTTAAACTTACTCTAGTAATTGTGTCCTTTCTGCCTAACGTTTATGGTGTTGGTTTTGTTTCTTTTATAAAAAAAAAAATTCAATTTTTGATTTACTTATTTGTATAGGCTAGTAACTATGTGTAAAATTTAAATAACATAAATTGAAATTTTTAGGTTTATTTTTCCTTTTGTTATTATAATTTCCAAACATTATGTCTAAATATGTTTTAATTGTGTGGAAGTATACAGTTTGACTGAAACATTTTTTTAGTATTTTAATTATTTCAACTGAAGAGCTGTAATGGGAAAACATGTTAAATGACTCTTAAGCAAATTTGAGGAAAAAATCTTTTAAATAAATAAAAACATCTAGGTGCTATATAATGAACATCTTTTTTTTCTCCATTGCCTTATTTAAAAACCTGGCTGATTGGAAGAACGTATAAATCGTATAACATGTTTTTGCTGGAAAAGCAAGCCCCCCTTTTAGACCCCTTATTCTTGCCACAGTTCACATTCAAGTAGTGGAAAAACATGTAAATCCCTTAACATGTTCTCAATGAAAGTGCATGTAAATCCCTTCATATTTTCAATGGAAAGACATTTAACATGTTTTCAGTAGAAAAATATCTCAATCATTTAACATGATCTTCTATCAAGCTCTTCAATTACAGTGTGAGTGGTTACTAATATTTATTTTTATGAATAATACGAATTGCAATTAACTTCCTAAAGTATAGAAATAAAGTCGAACGTCTATTTAACAAATTTGCTAAATCCCGATAATGGAAAATTCATTAAATATAAAATCGCTTAAACAACACAAAACAGGTTTTAAATTCAT

SRA found in SRX2855144 Male soma sample

READ ID:  SRA:SRR5602551.8796090.1

SRA found in SRX2746609 stage 1-3 embryonic samples

Read ID: SRA:SRR5458595.93228699.1

Read ID:  SRA:SRR5458595.93228699.2

Query 5 TGGCTACATCGCATCTTATGGATACTTCTTTTTTGCTAGGGCCCTCCAACATGCTCTCTG 64

||||||||||||||||||||||||||||||||||||||||||||||||||||||||||||

Sbjct 100 TGGCTACATCGCATCTTATGGATACTTCTTTTTTGCTAGGGCCCTCCAACATGCTCTCTG 41

Query 65 TGGTGCCCTCGCAAGTGGGGTCCCCGCAACAACAGTCCAG 104

||||||||||||||||||||||||||||||||||||||||

Sbjct 40 TGGTGCCCTCGCAAGTGGGGTCCCCGCAACAACAGTCCAG 1

##### >Parasteatoda_tepidariorum_bFTZF1

ATGATGGCTACATCGCATCTTATGGATACTTCTTTTTTGCTAGGGCCCTCCAACATGCTCTCTGTGGTGCCCTCGCAAGTGGGGTCCCCGCAACAACAGTCCAGTGGCCCCCTGGTTGAGTATTCACATGACCTGCCGGACACCAAGGAGGGTATTGAGGAACTGTGCCCTGTTTGTGGGGACAAAGTGTCCGGATACCACTATGGTCTGCTCACGTGCGAGTCTTGCAAAGGATTTTTCAAGAGAACGGTACAGAATAAAAAAGTTTACACCTGCGTGGCCGACCGCAGTTGTCACATTGATAAAAGTCAAAGGAAAAGATGTCCCTTCTGCCGATTTCAAAAATGTCTGGACGTTGGAATGAAACTTGAAGCTGTACGAGCAGATCGAATGAGAGGAGGTAGGAACAAATTCGGACCTATGTACAAACGTGACAGAGCACGAAAGCTACAAATGATGAGACAAAAACAAATCCAGAGAGCCCAGTGTCTCGGTATCATGACCAACAACGAGGTCACCCCAGCCTTGCCTCCACCATTCGCACCCCCAGCAATGCCATCACCATCAGTCTACGACCCCCATCACATCAAACAAGAACTCATCCAAATACCCCAACTGAGCTCATCCACCAGCTCACCGGACTCATCTCCCTCTCCCCTCTGTACCTCAGCTCAGACCAACTCAACGATGGCGGCTCATTTTGCCCTGGCCGGTGGACCACCAGTCCTGTCTACCGTGCCAGAGCCAATGAAATCATGGCCTTCATCCACCAGGGCACCATCACCACCCCATCCGACTGGGTCGAATGGTGGTGCATCCTCTGGTTCACCCAAACCACCTTATCACTACGGAGACGTGAGTCCTGTACCACTTACAGTAAGAAGTCCATCACCTATTCTTATCGGAAGTGGTAAGATCCCTCACCTAATCAAAGATTTTCAGACGTCCATGCCCGACGACAAAGATTGGCAGTCTCAACTCTTTGGTCTTCTTCAAAATCAAACATACAACCAGTGTGAGGTGGATCTTTTTGAACTTATGTGTAAAGTTATTGACCAATCACTTTTCGCGCAAGTTGATTGGGCCCGCAATAGTGTCTTCTTCAAGGAACTGAAGGTGGATGATCAAATGAAACTGCTGCAGCACTCTTGGAGTGATATGTTGGTTTTAGATCATCTTCATCAGAGGCTTCACAACGGCCTTCCTGAAGAGATAACAATGCCCAATGGTCAAAAGTTCAGTATGGTTAATTTGGCTCTACTAGGGGTAGCTAGTATGACAGACCAGTTAAATCATGTGTCAGCCCGGTTACAAGAACTCAAATTTGATCCCCTAGATTATATATGTCTCAAGTTTCTACTACTACTCAATCCTGCAGAAGTAAGAACACTGGCGAATGTCAAGTTGGTGATTGATGCATATGAACGAACACAACAAGCCCTGGTTGAATATACACTCAGCTGTTACCCACAATTTCCAGATAAATTTAACAACTTAATGTCAATACTGCCAGAAATCCATGTGATCAGTGAACGGGGGGAAGATTTTCTACTCTACCGACATCTAAACGGAAGTGCACCCTCACAGACTCTACTCATGGAAATGTTACATGCGAAGAAAAAATAGCAGGACTTGAAAAATTTTGTTGTCTTTATATGTTGATAATATTTACAGTTGTAACTGCCATCAGTTCAATTCTTTCTATAACTGCCAGAAAGACTTTCGGATCTAACTCTGTACATTCCATTAGGAGGGGTCAGCCCACCCCTCAACCCCCTAAAGGGATGTTGGAATTTTTGAGATCCTCGCTAAAATGAACTTGATACCATTTGATGTTGTTATTCTCTAAGCACACAATTCTCTTGTACATTTAAAAATTGAAAGACTTCATGCATTCTACGTCAACTAACAAAGCCATTGTCCTGTTTTAAGAAAAATCATCTGTTCATTGTTATTGCTCCCTCCCCTTCTGTAGAAATTTTATTTCCTTATATTTGTACAGTTGTAAGAGGGAAGCTGTTTTTTTTCTTCTCTTTTTTGTGGTTCAAACTTGTTTATAATTATTAAGAAGAAGTGAAATATAAAAGCATTTGGGAGAGAGATGATTTCATTTAAATAGTTTAAATTATACCATAACTAACTGTTCTTAAACAAAACTCTTAATAATGTAGAGTAGACTCTTAAAAGTTTAAACTGTTCATTTTGTTAACCTCATGTATCTCAGGGAATTGATTGAATGTAATTTTTTTCTCCTTTTTTTAGAGTATTATAAGCAATAAGAATCTTTATTTCATTTCTTTATAGTGGCACAGTTGGTTTTGATTTTATTAGAATATCCTACAAAAGGACAAAATTATTTTTTAAATCTTTTATTAATTAATTATTATCTTTAAAAATATTACAATTTATAATATAAGCTTTCTGCAATTTTTTTATTTCTAAAAAAAATTTACCTTTTATTATATGTTCTATACTTTTTCCTTTACTGCTATAGACAAATTCTACTGCTTGTGAACTAGTCAGATATTTTTTATATATTATTATGTGTATAATTTTTCAAAATTTTGTAAATAAAATTATTTAAGCGTATCATATTTGTATTTTGTATATATTGTAATTTTTTTTTTTGCAACTTTTGAATGATAAGAAAAAATTATGTATCCCAACTGTGTCACACTCCAAAAAATTGTTTGCCCTAATTGAATTTTGACTGATTCACTATTATTTCAAAATCACCAGTGTTTATGATTCACTACTGCAAAATATGAGTTTTCAAAATGTAATTTTTCATTTTTTTTCTTCAAAATTGTGATTTGTTTTTGTTTGTAGATAACACATTCCTCTGTTCAATTTCTTCCCTATGCATTGTTTAAACTTACTCTAGTAATTGTGTCCTTTCTGCCTAACGTTTATGGTGTTGGTTTTGTTTCTTTTATAAAAAAAAAAATTCAATTTTTGATTTACTTATTTGTATAGGCTAGTAACTATGTGTAAAATTTAAATAACATAAATTGAAATTTTTAGGTTTATTTTTCCTTTTGTTATTATAATTTCCAAACATTATGTCTAAATATGTTTTAATTGTGTGGAAGTATACAGTTTGACTGAAACATTTTTTTAGTATTTTAATTATTTCAACTGAAGAGCTGTAATGGGAAAACATGTTAAATGACTCTTAAGCAAATTTGAGGAAAAAATCTTTTAAATAAATAAAAACATCTAGGTGCTATATAATGAACATCTTTTTTTTCTCCATTGCCTTATTTAAAAACCTGGCTGATTGGAAGAACGTATAAATCGTATAACATGTTTTTGCTGGAAAAGCAAGCCCCCCTTTTAGACCCCTTATTCTTGCCACAGTTCACATTCAAGTAGTGGAAAAACATGTAAATCCCTTAACATGTTCTCAATGAAAGTGCATGTAAATCCCTTCATATTTTCAATGGAAAGACATTTAACATGTTTTCAGTAGAAAAATATCTCAATCATTTAACATGATCTTCTATCAAGCTCTTCAATTACAGTGTGAGTGGTTACTAATATTTATTTTTATGAATAATACGAATTGCAATTAACTTCCTAAAGTATAGAAATAAAGTCGAACGTCTATTTAACAAATTTGCTAAATCCCGATAATGGAAAATTCATTAAATATAAAATCGCTTAAACAACACAAAACAGGTTTTAAATTCAT

### Scorpion (Centruroides sculpturatus)


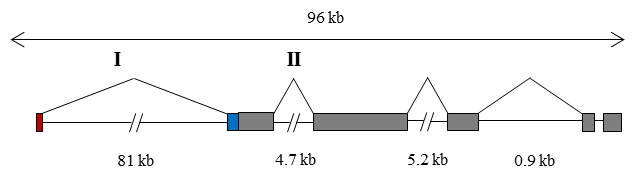


Reads found **SRX2855101**

Query 390 TGCGAACCTACCACCAGTACTGCAACTTCTACTGACGTATCAGCTACCCCATCGATGTTG 449

||||||||||||||||||||||||||||||||||||||||||||||||||||||||||||

Sbjct 1 TGCGAACCTACCACCAGTACTGCAACTTCTACTGACGTATCAGCTACCCCATCGATGTTG 60

Query 450 TCTGGACCGACTCCTACACCGACGGAGTATCATCACGACACTCCGGACACCAAAGAAGGG 509

||||||||||||||||||||||||||||||||||||||||||||||||||||||||||||

Sbjct 61 TCTGGACCGACTCCTACACCGACGGAGTATCATCACGACACTCCGGACACCAAAGAAGGG 120

Query 510 ATTGAAGAACTTTGTCCCGTTTGTGGCGAC 539

||||||||||||||||||||||||||||||

Sbjct 121 ATTGAAGAACTTTGTCCCGTTTGTGGCGAC 150

##### >aFTZF1_scorpion _XM_023378248.1

AACCCACTTTCGCGCTTGCTCACTGTGGACGAAAGTGAAGTTGGCCGAAGCCTGGAAGCCCTTGTCCTTGATTCCGCCGGTATAACGCGCGTGTAATGCTTTAGGATTTTCGTGTGTGGTCAACGTTCGAATGCCCACTCTCCCCTTCGACCCGTGGTTGCCCGACAGTGTGTTAACGAGACATTAAGAAGACGTCACACGGCGGACACGGCACATACAACACTACTCAATCACACCAAACTCGAATGTGTATACGGAGAAGGGGCGCAATGGAGACCGTGGCCGTACTCGAGTCTGTCAATGAAGTGTTGTAGCATCGTTTACTGTAAACTCCATTTTTTTACCGTTTCCAACAATTTCTACTATGTGATTTCGTATACAAGATGAGTTGCGAACCTACCACCAGTACTGCAACTTCTACTGACGTATCAGCTACCCCATCGATGTTGTCTGGACCGACTCCTACACCGACGGAGTATCATCACGACACTCCGGACACCAAAGAAGGGATTGAAGAACTTTGTCCCGTTTGTGGCGACACTGTGTCGGGTTATCATTATGGTCTGTTGACGTGTGAATCTTGTAAAGGTTTCTTCAAACGGACAGTGCAGAACAAGAAAGTGTACACGTGTGTCGCAGAAAGGAAATGTCATATAGATAAAAGTCAGAGGAAACGTTGTCCGTTTTGCAGATTTCAGAAATGTTTAGAAGTTGGCATGAAGCTCGAAGCTGTACGTGCAGACAGAATGAGGGGTGGAAGGAACAAATTCGGACCAATGTACAAAAGGGACAGAGCTAGGAAATTGCAGCTTCTGCGCCAAAAGCAATTAGCGCGTAACCAATGTCAAGGGGTCCCGGCGACGGAAGGAGGAATGAACAATCCTTTGCCTTCGCATCCTCCTTCATTCACAGCTTCGACAGGACCGGCGGGTGTTACGGCCCCTCAACAACTTTATTCTCCAGAAACCGTCTCTATGAAACAAGAACTTATTCAAATCCCTCACCTGAGTTCGTCTACGAATTCGCCCGAATCTTCTCCTTCACCTCTCTCTGCCATCATCCCTGTCACTACTCTAGGAAGTCACTATAGTGGGCCACAACCGACCAGAATTCATCCACAAGCACCAGTGGTACAAGAACCATCGAAATTAACTTGGGTCACGTCACACGCGCCACAAAACACAACACCGTCGCCTGGTCATGGTGCATCGCCGCCTGTTACTAATGCGGCACCCTCTCCCAAAACTTACCACTACGATGTTCCGTCCGCTGCAAATGTTCCACCTCCTTCGGTATCGTTAATGACAGGAATCGGAAAAATTCCGAATTTAATTCGGGAATTCCAATCCTGCATGGCCGACGATAAGGATTGGCAGAACCAAGTTTATAGTTTATTGCAAAGTCAGAGTTATAATCAGTGTGAAGTGGATCTGTTCGAACTGATGTGCAAAGTGATTGACCAATCGCTGTTCTTCCAAGTGGATTGGGCAAGGAACAGCATATTTTTTAAAGACCTAAAGGTTGACGACCAAATGAAATTATTACAACATGCATGGAGCGACATGTTGATCTTGGACCATCTTCATCAGAGACTCCATAACGGTTTGCCAGATGAAACTACTTTGCCAAACGGACAGAAATTTGATATGTTATGCCTAGCGTTATTAGGATTACCGGCAATGGCTGACCAGTTGCATCGTGTAGCTACTCGTCTTCAAACTTTAAAATTCGATCCTCTAGATTATATATGCCTCAAGTTCATTGTTCTGCTCAATCCAGAAATCCGGGGGCTGTCAAACATGAAGCTTGTCCACGAAGCTTACGAACAAACCCAGCAGGCGTTATTGGAGTACACCATGAACTGTTATCCACAAGTGACGGAGAAGTTTAATCAACTGATGCAGGTATTACCGGAGATCAAAGTGATCAGCATGAGAGGTGAGGAATATTTGTATTACAAACACATAAACAGCAGTATTCCGGCACAGACACTACTCATGGAAATGTTACACGCTAAGAAGAAATGACAACAGACACCTGTACTAACAGAAGAACTTTAAAGAAAAACGGTGAAAGCAACCATAATTATCCGAAACCAGACATCTTAGGCTGCACGAAGGGATTCAACGTAGTACCTCTGTCTACAAAGAGGGAACCTGTGAGCTCCCAATGCAAAACACTGCCGTATATATATAAATATATATATATATAAATTGTACACCATGTACTATTGCCAGCATTGATTCTTATTTTCCTTTTTTTTTCTTTTCCCATAAAGGCACTTTTCATTATTAGTTTTTCTTCAGTTCATATTTGTGATTATATTTGTTTGTACTGCCAAAATTGACACGGGTCTGTTCGTCAGTATATTCTGTACAGATATAGATAGAATGGAAAAAGATAAAGATGATAAGATATTAAAAAAACTCATACGACTCAGGGAATACGTCAAGCACAGCCACTCAAAAACAGCAAAAAAATAATAAATAATAATAATAATAAACTTTTTAAAGAGAGTATTCGAAATGAGCGGGTTGGAGTTTTAACCCATGTGTCAGGCAGTCCAAACTATAGATTTTTAGATTTAAATAATTATATATTATAAATATTATTATATTTTTTCGTTGTGTATACCGTTACAACAGAGATTCAATTGAAAACTGTTCTGTTCTTTTATTTTATTTTTTATAGCCAGGCCAAAATTTTAAGTCTGTTTCGATACTTATTTTCTGCTCTGCAACCTCTGGGTTAATTTTAAAAAAATTAATAATGATGATGATAATAAAAGTCTGTATGGTGTTCGTCGTGAAATTTTAACCCCCTTCTACTTATATACATGTGCTGGTGTAGGCTTCTGTCGCCGCCTCTGTACATAGATGTTGTAGTTAGTTGGTTGCGGGACACGTGTCAACGTAACATATATTTGGACACCACGCTCTCGGACTCGACAGAAAATGGTCTAGTGTTGAAAGTATAGATATATTTTCTACGATATACACGTGACATTATATTAAAAAAAACAAACGGCTTAGGAAGCACCATTATAAAAAGAGGAAAAATCATAATACTAATAATTCTTGTATATAGTTTTGACAGAAGTACAAATTCACTTCTCAGCTGTTGTATTTTGTGAATTGTTAATGGAGAGGTTACCTGTTAAGAGTTGTTTATAGTAAAAAAAAAAAAGGTGGTATATATATATATATTATATATATATTATATATTTTTTGATTGTAACGCTGATCTGGAAAGTGTGTGTGCCTTAATATACCAATAGAGATGCGTTTGTGAATAATCACTTATACATACTAATCGTTGTGTTTTTAAAATTATGATAAAATGAATTTGTTAAAATTATTATTAAGAAATCTTTTAATTTTGAAGCTATTTTACTGTATGTATAAATGTTTATTCACCACGTAATTTATTGAGGAATGTCTCATGAGCACAAACATACCATACATACATGGAAAACCCCCTAAAAGTTATTAATGGGCTGTTATCAGCATGAAAAATGGTGTCCGATTTAGCGATGTGTCCATCTTGTCATTGTCATCACACATTTTTTTTTCCATTATTATGTTTATCATCATGGAAATGGAGGGTGATGGACCAAAAACAACTGTTCACGAGATAAAAAAAAAATGGGGAAATCAAAAAAATTAATACATACATTTTTTAAAAAAGGGATAACTCGGGGAGAAATAAAATCAAAATTATTACTAAAGAATAAAGTTATAAGATTTTTATGCCCTTTGTCAAATTTTGAACTCGAGTGATAGTCGCACACAACAGAGTTCTTCAGGTGCCTTCCTATCTACCATTGTGTTTTGTTGATTGTTATTCCCTGAATGGTGATACATCTTTGAATGTGGAGGGCCACTGAATTAGAAAAAAAA

Read found SRX2855101

Read ID:SRA:SRR5602514.24772315.2

READ ID:SRA:SRR5602514.27432960.2

READ ID:SRA:SRR5602514.33015710.2

Query 422 TGTTGATAAACCCTCTTGATGATACAACGCGTTTGTCAACAATGATATCTTCAGCGTCTC 481

||||||||||||||||||||||||||||||||||||||||||||||||||||||||||||

Sbjct 1 TGTTGATAAACCCTCTTGATGATACAACGCGTTTGTCAACAATGATATCTTCAGCGTCTC 60

Query 482 TCATGGATACTTCTGCATGTTTTTTGCCAGCTACCCCATCGATGTTGTCTGGACCGACTC 541

||||||||||||||||||||||||||||||||||||||||||||||||||||||||||||

Sbjct 61 TCATGGATACTTCTGCATGTTTTTTGCCAGCTACCCCATCGATGTTGTCTGGACCGACTC 120

Query 542 CTACACCGACGGAGTATCATCACGACACTC 571

||||||||||||||||||||||||||||||

Sbjct 121 CTACACCGACGGAGTATCATCACGACACTC 150

##### >bFTZF1_scorpion_XM_023378247.1

TTGCTGACATAACATTGAAGCGATCCCTCCTTCCAATTACACAACTTTTCTCACTGAAACGAGATGTCGTCATTCGCAACGCGCTCGAAATCCGAACGTTTTTCGCATAAGAGAAAGTGAGAATTAAAAAAAAATAAATAAGAGTCGTTTTGTAACGACCGCTACGCCATCCAACCCACCCAGCTGGTTGTTAAGCTGCCATCAACCAGCCATTGGCTAGAAGTGGGTCAGGCTGGTTGCGCCGACTCAGTCGCTTCTGACATAGACCCACTGGGTCGGCTCTATACTTGTAGGTCGTATTAGTTGGTCGCTCACCATTCACGCCATCACCACCAGAAACGGCGTTCGCACTTTCGTTAACGGTATGAAATTAAATCGTTGAAAGTGTGTCTAGTATCTCCTGCGTCATGTACCGATTACATGTTGATAAACCCTCTTGATGATACAACGCGTTTGTCAACAATGATATCTTCAGCGTCTCTCATGGATACTTCTGCATGTTTTTTGCCAGCTACCCCATCGATGTTGTCTGGACCGACTCCTACACCGACGGAGTATCATCACGACACTCCGGACACCAAAGAAGGGATTGAAGAACTTTGTCCCGTTTGTGGCGACACTGTGTCGGGTTATCATTATGGTCTGTTGACGTGTGAATCTTGTAAAGGTTTCTTCAAACGGACAGTGCAGAACAAGAAAGTGTACACGTGTGTCGCAGAAAGGAAATGTCATATAGATAAAAGTCAGAGGAAACGTTGTCCGTTTTGCAGATTTCAGAAATGTTTAGAAGTTGGCATGAAGCTCGAAGCTGTACGTGCAGACAGAATGAGGGGTGGAAGGAACAAATTCGGACCAATGTACAAAAGGGACAGAGCTAGGAAATTGCAGCTTCTGCGCCAAAAGCAATTAGCGCGTAACCAATGTCAAGGGGTCCCGGCGACGGAAGGAGGAATGAACAATCCTTTGCCTTCGCATCCTCCTTCATTCACAGCTTCGACAGGACCGGCGGGTGTTACGGCCCCTCAACAACTTTATTCTCCAGAAACCGTCTCTATGAAACAAGAACTTATTCAAATCCCTCACCTGAGTTCGTCTACGAATTCGCCCGAATCTTCTCCTTCACCTCTCTCTGCCATCATCCCTGTCACTACTCTAGGAAGTCACTATAGTGGGCCACAACCGACCAGAATTCATCCACAAGCACCAGTGGTACAAGAACCATCGAAATTAACTTGGGTCACGTCACACGCGCCACAAAACACAACACCGTCGCCTGGTCATGGTGCATCGCCGCCTGTTACTAATGCGGCACCCTCTCCCAAAACTTACCACTACGATGTTCCGTCCGCTGCAAATGTTCCACCTCCTTCGGTATCGTTAATGACAGGAATCGGAAAAATTCCGAATTTAATTCGGGAATTCCAATCCTGCATGGCCGACGATAAGGATTGGCAGAACCAAGTTTATAGTTTATTGCAAAGTCAGAGTTATAATCAGTGTGAAGTGGATCTGTTCGAACTGATGTGCAAAGTGATTGACCAATCGCTGTTCTTCCAAGTGGATTGGGCAAGGAACAGCATATTTTTTAAAGACCTAAAGGTTGACGACCAAATGAAATTATTACAACATGCATGGAGCGACATGTTGATCTTGGACCATCTTCATCAGAGACTCCATAACGGTTTGCCAGATGAAACTACTTTGCCAAACGGACAGAAATTTGATATGTTATGCCTAGCGTTATTAGGATTACCGGCAATGGCTGACCAGTTGCATCGTGTAGCTACTCGTCTTCAAACTTTAAAATTCGATCCTCTAGATTATATATGCCTCAAGTTCATTGTTCTGCTCAATCCAGAAATCCGGGGGCTGTCAAACATGAAGCTTGTCCACGAAGCTTACGAACAAACCCAGCAGGCGTTATTGGAGTACACCATGAACTGTTATCCACAAGTGACGGAGAAGTTTAATCAACTGATGCAGGTATTACCGGAGATCAAAGTGATCAGCATGAGAGGTGAGGAATATTTGTATTACAAACACATAAACAGCAGTATTCCGGCACAGACACTACTCATGGAAATGTTACACGCTAAGAAGAAATGACAACAGACACCTGTACTAACAGAAGAACTTTAAAGAAAAACGGTGAAAGCAACCATAATTATCCGAAACCAGACATCTTAGGCTGCACGAAGGGATTCAACGTAGTACCTCTGTCTACAAAGAGGGAACCTGTGAGCTCCCAATGCAAAACACTGCCGTATATATATAAATATATATATATATAAATTGTACACCATGTACTATTGCCAGCATTGATTCTTATTTTCCTTTTTTTTTCTTTTCCCATAAAGGCACTTTTCATTATTAGTTTTTCTTCAGTTCATATTTGTGATTATATTTGTTTGTACTGCCAAAATTGACACGGGTCTGTTCGTCAGTATATTCTGTACAGATATAGATAGAATGGAAAAAGATAAAGATGATAAGATATTAAAAAAACTCATACGACTCAGGGAATACGTCAAGCACAGCCACTCAAAAACAGCAAAAAAATAATAAATAATAATAATAATAAACTTTTTAAAGAGAGTATTCGAAATGAGCGGGTTGGAGTTTTAACCCATGTGTCAGGCAGTCCAAACTATAGATTTTTAGATTTAAATAATTATATATTATAAATATTATTATATTTTTTCGTTGTGTATACCGTTACAACAGAGATTCAATTGAAAACTGTTCTGTTCTTTTATTTTATTTTTTATAGCCAGGCCAAAATTTTAAGTCTGTTTCGATACTTATTTTCTGCTCTGCAACCTCTGGGTTAATTTTAAAAAAATTAATAATGATGATGATAATAAAAGTCTGTATGGTGTTCGTCGTGAAATTTTAACCCCCTTCTACTTATATACATGTGCTGGTGTAGGCTTCTGTCGCCGCCTCTGTACATAGATGTTGTAGTTAGTTGGTTGCGGGACACGTGTCAACGTAACATATATTTGGACACCACGCTCTCGGACTCGACAGAAAATGGTCTAGTGTTGAAAGTATAGATATATTTTCTACGATATACACGTGACATTATATTAAAAAAAACAAACGGCTTAGGAAGCACCATTATAAAAAGAGGAAAAATCATAATACTAATAATTCTTGTATATAGTTTTGACAGAAGTACAAATTCACTTCTCAGCTGTTGTATTTTGTGAATTGTTAATGGAGAGGTTACCTGTTAAGAGTTGTTTATAGTAAAAAAAAAAAAGGTGGTATATATATATATATTATATATATATTATATATTTTTTGATTGTAACGCTGATCTGGAAAGTGTGTGTGCCTTAATATACCAATAGAGATGCGTTTGTGAATAATCACTTATACATACTAATCGTTGTGTTTTTAAAATTATGATAAAATGAATTTGTTAAAATTATTATTAAGAAATCTTTTAATTTTGAAGCTATTTTACTGTATGTATAAATGTTTATTCACCACGTAATTTATTGAGGAATGTCTCATGAGCACAAACATACCATACATACATGGAAAACCCCCTAAAAGTTATTAATGGGCTGTTATCAGCATGAAAAATGGTGTCCGATTTAGCGATGTGTCCATCTTGTCATTGTCATCACACATTTTTTTTTCCATTATTATGTTTATCATCATGGAAATGGAGGGTGATGGACCAAAAACAACTGTTCACGAGATAAAAAAAAAATGGGGAAATCAAAAAAATTAATACATACATTTTTTAAAAAAGGGATAACTCGGGGAGAAATAAAATCAAAATTATTACTAAAGAATAAAGTTATAAGATTTTTATGCCCTTTGTCAAATTTTGAACTCGAGTGATAGTCGCACACAACAGAGTTCTTCAGGTGCCTTCCTATCTACCATTGTGTTTTGTTGATTGTTATTCCCTGAATGGTGATACATCTTTGAATGTGGAGGGCCACTGAATTAGAAAAAAAA

### Acariformes (Varroa destructor)


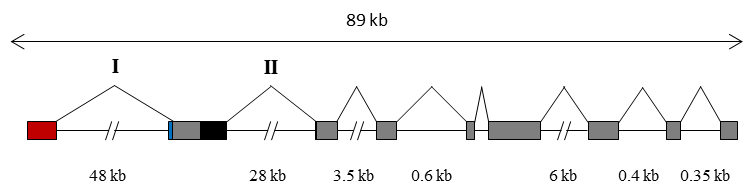


Reads found in SRX4926905

Query 1567 CCGACTGGAGTGCGGACTATGCTGGCTGTGGCCGGGGGCGAGCAGGCCAACTCGCCCGAT 1626

||||||||||||||||||||||||||||||||||||||||||||||||||||||||||||

Sbjct 150 CCGACTGGAGTGCGGACTATGCTGGCTGTGGCCGGGGGCGAGCAGGCCAACTCGCCCGAT 91

Query 1627 TCCCTTATGccccccccccACACCAATAATAGCAGTACTAATCAAAATAATAGCCTCGGA 1686

||||||||||||||||||||||||||||||||||||||||||||||||||||||||||||

Sbjct 90 TCCCTTATGCCCCCCCCCCACACCAATAATAGCAGTACTAATCAAAATAATAGCCTCGGA 31

Query 1687 AGACAGGACCAACAAAAGTCAACAACGAAT 1716

||||||||||||||||||||||||||||||

Sbjct 30 AGACAGGACCAACAAAAGTCAACAACGAAT 1

##### >XM_022789294.1

AGTTACCATTGTCTATTTGTCTGCGTATCGGTCGCCCTATGACTCTGCTGTAGCAAACGTAGTAGTGAGTGTCTGCTTAACGGCAGTGCCGGACGTAAAACTCGAAACATTTATTTAGCAGTGAGTGTCTGTGTCTGATTGCATCCAGTGGCAGTGGCGGTATCAGTACTGGCATCGGTTGCAAATGTTTTTCTTGTGGCTACCGCCATTGGCCCTAGCAACTGCAGTAGAGGCGATCATAGGCTAGCCGCCTGCCCCTTTCGTGGTCCATCCACTCCCCCCTGCCTCGACCCCTCCCCTTTCCCCCTAGATTTAGGCTGGACTGCTCTCACTCGGTAGACTAGGGGCCAACCCACGGACCACCGCCTGCCTGTCCGACCCCCACCTGTCAGCCCGTCGCAGCCCCCGGCGCAGCCGGGCCTGCTACCACTGCTGCCTGTCTGCCCGCGCGCCCCAGCCCAGTGACCTACTTTGAGGGCTGACTGGCAGGCTCGGTCGCTCAGCGTGGCTGCTGACCGGGGCCGTCTTCGAGACTGCGCCATCGTCCGCCGAAACCACCGCTGTATATCTTTCTTTCTCTTTTCCGGTCCGTCTGTGCCGTACTGTGTCGTTGAGTCGATCGTCCGTGCTGCTGTGCTGTGTTTTGTTTTGCTGCATGTGGTACGGTGCGTGCCATTGCCGTCGCGGCCCTTTTCAACGCTTGCCCCGTGGGCTGTTGTGTTGTACCCGCCGTTGTCGCGGCCTCGCCAGTCGTAATAGCAACAACAACAGGAATAGCGCCAACATTTCGTCGACAACAACAATAGCAACAACGGCGACGTCAACAACAGCTACCATTATGAGCAGAGGTAGCAACAAAAGCAGTGACACTGCTTACTAGTGATAGCGTTACGGCGAGCAATAGTCACAACAACAACGACAGAATCATCCGGCAACAAGCTATCGCGGCCGCCTTCTGGCGCGGTGCTCGACACAGCGCAGCTACAACGACAACCAACAACCAGCGATAGAAGCAGCTTCGAAGGACGAAGCGGCTCCAAAGGCAGCAGCAGTAACGCTAGCAGTACTGCTACCACTGCGATAGTTGTTGAATAGCGGCAGCAGGAGCCAGAAAACGATAGCAGACTGTCCAACCAGGCGTCCATGTAGCGCTCTCTCTCGAGGGATCCGTCCGACAAATGTCCGTCGATTCCTCGACGAGGAGTCGAGACTGCGGGTCTGGAAGATTCTGACGGCAGTGACGACGGCGCCAACAGCTGCGGCGATCATCCTTCACGCCGGCGATTTCTGTGAACACTACTATAACTGCAGCGGGTTCCAACAGTTATACTACAGTAGCGCGACGATAACGCGAAACTGTGCGAAGTGACTGTGCGACGGCTATCTTGAGTGTGTTAAGTGGTAATCCAGTCCTCACGGCTCGCATATACTTGTGTTGTCAGATGTGTAGTGCTTTGTGGTAAAAGTCGTGCAAATTTAATTCATCGACAGAAGTTTGCACTGCTACGTATATTAGTTCGCTCGTCTAACGTGAAAGCTCTGTGCGCGTGCAAGTAGTGAGTGAACCCGACTGGAGTGCGGACTATGCTGGCTGTGGCCGGGGGCGAGCAGGCCAACTCGCCCGATTCCCTTATGCCCCCCCCCCACACCAATAATAGCAGTACTAATCAAAATAATAGCCTCGGAAGACAGGACCAACAAAAGTCAACAACGAATACTACTAGCACCACGACTGCTCCGACGACAACGACGACGCTCACGTTAAAAAACGGCGCCAGTTGTGATTCTGAGAAGACCAACTCGAGCACCGCGTCAGCCTCGCAAGGGCCGAACCAGTACAGTGCGGATACGTATCAGCCTCCGGTCAACGTGATGGCAGTCAGTGGAGTTGCAGGGGTTACTGGTGTGTCAGTGGCACCGCCCCCTGGTGCCGTTGTTGGCCACGCTAATCCCTCCCTCGAACTTGCGGTCATTTCTCCGACCAACTCGGCCGCACTCGTCACCTCGGCGGATTCCAAGGAGGGCATCGAGGAACTGTGCCCAGTCTGTGGTGATAAAGTGTCTGGCTACCACTATGGCCTACTTACGTGTGAATCCTGCAAAGGCTTCTTCAAGAGGACGGTTCAGAACAAAAAGGTCTACACATGCGTGGCTGACAGATCGTGCCACATCGACAAGTCACAGAGGAAAAGATGCCCCTTCTGTCGCTTCCAGAAGTGCCTCGACGTCGGGATGAAACTCGAAGCGGTGCGAGCGGATCGGATGCGCGGCGGACGAAACAAATTCGGTCCAATGTACAAGCGGGATCGTGCGAGAAAGCTGCAGATGTTACGCCAGAAACAGCTGGCGTTACAATGCGCTGACTCCATGTCCCCCAGCACACTGGTCTCTATCGCCACCAACCCATACCCCATTCCCACTGCTTCGGTTTCATCGCCAGGTATGTATGATACGAACGGTGGTGGAACGGGTGGTGTTAAACAAGAGATTCAGATACCTCAGCTGAGCTCTTCCACCTCTTCACCAGATTCTTCGCCTTCTCCCCTGCAAACGACGACGACAACCACGAGTTTATCCGGCCTCACCAATACTACCACGACGGATTCGAGTATCTCCTCGCTGCTTGACATCTCACTCCGCCATCCATTCACCACAAAGGTAACTCCTCTAAACCCGCAGGGACACTTCGAGACGCCGAAAACATCAGCGCCTAGTAACAATAATAACAACAACGTGAGCTGGGTCCCGCAGGTTGCAGGCACGGGTGGTGGATCAGGGGGCGGAGGCGGTGCGAAACAAAACCTCGTTCCGTTCCAGAATTTCGAAAGTGGCTTATCTTCATCCCAATCGCCTCTGCCACTGGTGCTAACCTCACAAGCGCCGTCACCTCAATCGAAAATACCGCACCTCGTTCGAGAGCTCCAAAAGAATATGACAGACGACAAGGAGTGGCAGTCCCAACTATTCCAGCTACTACAATCGCAAACATACAACCAATGCGAAGTAGACCTCTTCGAGCTCATGTGCAAGGTTATTGATCAGGCACTGTTCGCTCAGGTCGATTGGGCACGCTCGACAATCTTCTTTAAGGAACTCAGGGTGGATGATCAAATGAAGCTATTGCAACAGTCCTGGTCGGATATGTTAATCCTTGATCATCTGCATCAGCGCATGCACCATTCGTTACCTGACGAAGCTGTACTACCTAATGGACAGAAGTTCGACCTTGTCTCATTATGTCTCCTCGGAGTTCCAGCTATGGCAGATCAGTTGAACACTGTGACCCATAAGTTGCTTGACCTACGATTTGATCAGATCGATTTCGCCTGTCTTAAATTCCTCATGCTACTTAATCCAGATCCCTCTTCAGCTGAGGTGCGCAGCCTATCGAACACACGGTTGGTTACGGAAGCCCAAGAACAGACGAAGCAAGTGCTTCTCGACTATTGCACGAGCAATTATCCCAGCGTCACTGATAAATTTGGTCAACTGATGGCGATCCTTCCCGAAATCAAGAGCATGTCAATGCGGGGTGAGGAGTTCCTATTCTTTAAGCACGTGAATGGAAATGCGCCGACCCAGACGTTGCTCATGGAGATGCTACATGCGAAACGGAAAGCCTAAGATAAGCCAGTGGGTTAATCCGTCCCCAAGTTCCCCTCACCTGCTATTATACTAGACGCCTTCCTTGGACGGGAAACCGTTTGAGGGACACGCAGACAGGCCGTCCTACTGCATCTCACGAAGTCTGGCGACCACACACCTGGAACCACGACAAGAACATCTCGATCATAATCGATGATGACTCTGATCCTGCCCCTTCATCCCTCAGTCTATTTGATAGGACAACTGTAGATAAACAACAGCTGGAATAGTGACAACAACACGGAGCCTTTGAGGTCGTTCCATAGTACGCAGTCGTTCCGAAAGAATTAAGAAGTATAGGATGAGAAGGCGGATATATTAAGATGAAGAAACAAGTTGTTCCCGAGTGATTTTTTTGTAGCTCGAATCTGTAGTAGTCGTCGCCGCCGTCGTCGTCGTCGTCGTCGTCGTCGT

Read found in **SRX4926906** and SRX4926905 (searched with 400 bp upstream of ATG start included)

READ ID:  **SRA:SRR8100122.75800737.2**

**READ ID:**  **SRA:SRR8100123.18219962.1**

Query 467 TTGTATTTCCTCAATATTTACAATTGCTTCGACACTGGACTATCTGAGCGCGCCCCTAGT 526

||||||||||||||||||||||||||||||||||||||||||||||||||||||||||||

Sbjct 150 TTGTATTTCCTCAATATTTACAATTGCTTCGACACTGGACTATCTGAGCGCGCCCCTAGT 91

Query 527 GGCGACCCCAGCCATTACAATGGCGTCATTCATGGATAATTTTTACTTTCCCACAGGGCC 586

||||||||||||||||||||||||||||||||||||||||||||||||||||||||||||

Sbjct 90 GGCGACCCCAGCCATTACAATGGCGTCATTCATGGATAATTTTTACTTTCCCACAGGGCC 31

Query 587 GAACCAGTACAGTGCGGATACGTATCAGCC 616

||||||||||||||||||||||||||||||

Sbjct 30 GAACCAGTACAGTGCGGATACGTATCAGCC 1

##### >FTZF1_Vdestructor_bFTZF1_Short

ATGGCGTCATTCATGGATAATTTTTACTTTCCCACAGGGCCGAACCAGTACAGTGCGGATACGTATCAGCCTCCGGTCAACGTGATGGCAGTCAGTGGAGTTGCAGGGGTTACTGGTGTGTCAGTGGCACCGCCCCCTGGTGCCGTTGTTGGCCACGCTAATCCCTCCCTCGAACTTGCGGTCATTTCTCCGACCAACTCGGCCGCACTCGTCACCTCGGCGGATTCCAAGGAGGGCATCGAGGAACTGTGCCCAGTCTGTGGTGATAAAGTGTCTGGCTACCACTATGGCCTACTTACGTGTGAATCCTGCAAAGGCTTCTTCAAGAGGACGGTTCAGAACAAAAAGGTCTACACATGCGTGGCTGACAGATCGTGCCACATCGACAAGTCACAGAGGAAAAGATGCCCCTTCTGTCGCTTCCAGAAGTGCCTCGACGTCGGGATGAAACTCGAAGCGGTGCGAGCGGATCGGATGCGCGGCGGACGAAACAAATTCGGTCCAATGTACAAGCGGGATCGTGCGAGAAAGCTGCAGATGTTACGCCAGAAACAGCTGGCGTTACAATGCGCTGACTCCATGTCCCCCAGCACACTGGTCTCTATCGCCACCAACCCATACCCCATTCCCACTGCTTCGGTTTCATCGCCAGGTATGTATGATACGAACGGTGGTGGAACGGGTGGTGTTAAACAAGAGATTCAGATACCTCAGCTGAGCTCTTCCACCTCTTCACCAGATTCTTCGCCTTCTCCCCTGCAAACGACGACGACAACCACGAGTTTATCCGGCCTCACCAATACTACCACGACGGATTCGAGTATCTCCTCGCTGCTTGACATCTCACTCCGCCATCCATTCACCACAAAGGTAACTCCTCTAAACCCGCAGGGACACTTCGAGACGCCGAAAACATCAGCGCCTAGTAACAATAATAACAACAACGTGAGCTGGGTCCCGCAGGTTGCAGGCACGGGTGGTGGATCAGGGGGCGGAGGCGGTGCGAAACAAAACCTCGTTCCGTTCCAGAATTTCGAAAGTGGCTTATCTTCATCCCAATCGCCTCTGCCACTGGTGCTAACCTCACAAGCGCCGTCACCTCAATCGAAAATACCGCACCTCGTTCGAGAGCTCCAAAAGAATATGACAGACGACAAGGAGTGGCAGTCCCAACTATTCCAGCTACTACAATCGCAAACATACAACCAATGCGAAGTAGACCTCTTCGAGCTCATGTGCAAGGTTATTGATCAGGCACTGTTCGCTCAGGTCGATTGGGCACGCTCGACAATCTTCTTTAAGGAACTCAGGGTGGATGATCAAATGAAGCTATTGCAACAGTCCTGGTCGGATATGTTAATCCTTGATCATCTGCATCAGCGCATGCACCATTCGTTACCTGACGAAGCTGTACTACCTAATGGACAGAAGTTCGACCTTGTCTCATTATGTCTCCTCGGAGTTCCAGCTATGGCAGATCAGTTGAACACTGTGACCCATAAGTTGCTTGACCTACGATTTGATCAGATCGATTTCGCCTGTCTTAAATTCCTCATGCTACTTAATCCAGATCCCTCTTCAGCTGAGGTGCGCAGCCTATCGAACACACGGTTGGTTACGGAAGCCCAAGAACAGACGAAGCAAGTGCTTCTCGACTATTGCACGAGCAATTATCCCAGCGTCACTGATAAATTTGGTCAACTGATGGCGATCCTTCCCGAAATCAAGAGCATGTCAATGCGGGGTGAGGAGTTCCTATTCTTTAAGCACGTGAATGGAAATGCGCCGACCCAGACGTTGCTCATGGAGATGCTACATGCGAAACGGAAAGCCTAAGATAAGCCAGTGGGTTAATCCGTCCCCAAGTTCCCCTCACCTGCTATTATACTAGACGCCTTCCTTGGACGGGAAACCGTTTGAGGGACACGCAGACAGGCCGTCCTACTGCATCTCACGAAGTCTGGCGACCACACACCTGGAACCACGACAAGAACATCTCGATCATAATCGATGATGACTCTGATCCTGCCCCTTCATCCCTCAGTCTATTTGATAGGACAACTGTAGATAAACAACAGCTGGAATAGTGACAACAACACGGAGCCTTTGAGGTCGTTCCATAGTACGCAGTCGTTCCGAAAGAATTAAGAAGTATAGGATGAGAAGGCGGATATATTAAGATGAAGAAACAAGTTGTTCCCGAGTGATTTTTTTGTAGCTCGAATCTGTAGTAGTCGTCGCCGCCGTCGTCGTCGTCGTCGTCGTCGTCGT

## Crustacea

### Branchiopoda (*Daphnia magna*)


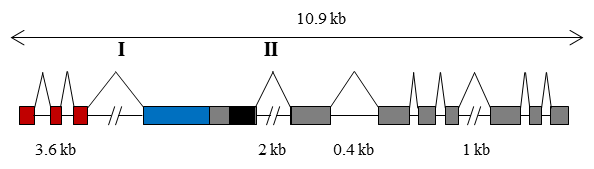


##### >LC105700.1

AGTCGTGCAGCAGTCCCGCACACACACGCCACCGTATTCGCAAGCTGCCAGTGAGATACATCCATCTGGGGAAAAAAAACGAAATACCCAAACTCTGCAACTGTGTGTGAAAACCGTGTGTGTGTGTGTGTGTGTGTGTATCTATTTTGATAACCAGTGTCAGCCGTCCCGCGTGAAATTTTCAGCAACGAGGTTTTCCGTCAATCATCATAACGTGGTTTAAATTGAAAATAAGGAATCAGTGCTTGGAAGCCAAACTTTGTCGAGTGATATTCGTGAAAGGGCAAAAAGTGAGCTGTGTTTATTTCGTTTTCGTCTCTTTTCTCTTTCAACAAGGGAGCGTGAATGGGATCCCCCACAAAGCTAAAAAGAAAATAATTCAAATTATCACGGCGAGACGGCTCTACTTGAAAGAGAGAAAACAAAAACACGAGAAAATGGAATGTCAAGCAGAAGCAGCAGCTTTGTTGCCTTCGTTCAGCGAGCTGACGTCGGCAGGAGTCGTCGGGCGTGACCGCCATGGATCACCGTCAGATGGCAGCACTGATGGGTCTCAGCAATCGCAGTTGTCGTCAACTTCGGAATCTTCAGCCGAATTGAGTAAAGAGCTCCGCTTGTCCTCGGCACACGCCATGATTTTCCTCTCACAACAGAAACGCGATTCCCTCCAACCTGACGAGCTATCCGCCCACGAAAGTGGGCCCACCTGCCTTCCAGCTGATTCAGATCACCTGATGCTCATCCAGCTCCAAGATTCACCACACGGCGAGTACGAAACGAGCAATGCGGCCAGCAGCAACGCGGCCCTCAGTCCATCACCTCTCATGGATCCTATCGTCATTGTTCCATCCATGTCTGGCCAATCGTCGGCCAGCTCTTTACCACCTGGAACGAGCTCGGCTTCGGGACGACACGACCTGCCCGATGCGAAAGAAGGCATCGAAGAATTGTGTCCGGTTTGCGGCGATAAAGTCTCTGGCTATCATTACGGTCTGCTCACCTGCGAATCGTGCAAGGGATTCTTCAAACGGACCGTTCAGAACAAAAAAGCCTATTCTTGCGTCGCTGATCGATCCTGCCACATTGACAAATCACAACGCAAGAGGTGCCCTTATTGCCGATTCCAAAAGTGCCTTGAAGTCGGCATGAAACTTGAAGCTGTGAGAGCGGACCGAATGCGTGGCGGTCGGAATAAATTTGGACCCATGTACAAACGAGATCGAGCGCGTAAATTGCAGGTGATGCGTGAAAGACAATTGACGACGCCTCGAGGTAGCAACGGTGGAGGTAGCAATCCGTCACCCAACAACAGCAACAGCGGAGCAGGGCAGTCTGGAATGTACACGGACATGGGCTATTCTCCGTCCGGCAGCAGCGTCTACGGTGGTGGCAGCAGTGGCGGTGGTGTCAAGCACGAAATTCAAATTGCCCAAGTTTCTTCGTTGACATCCAGTCCCGATTCCAGCCCGAGTCCACTTGCCGCATCACTCGGCTATCCAGGACCTTCTGGCCAGCCGTTGGGCTCGTTGAACGGTTCTACGGGACCATCCGGATCAGGCGGCCCGCGCAATAACAATAATACAAATAACCACAATAATAATAACATGGGCACGACATCGGGAGCGGGACCCTCTCATCCGCACACACCTTCTCCAAATAATCACCCGTCCGGCGGAGGAGGAGGAGGAGGCGCTCCTACACCCCAACAGCATTCCTCCACTTCATCCGTCTCACCGAAAACCTTCCATTTTGAGGGACTGTTGACTGGTAACACAATGCTGGGTAATAACAACGTTCCCAATGTCGGAGCCGGATCAGTTAGCGGGGCTAAAGTTCCGCCATTGATCCGCGAATTTGTCCAGTCTCTTGACGATAAAGAATGGCAAAGCGCTTTGTTTGGTCTTCTTCAGAGTCAAACCTACAATCAATGTGAAGTGGATTTGTTTGAACTGCTGTGTAAGGTGCTGGACCAAAACCTCTTTACCCAAGTGGACTGGGCACGCAATTCCTATTATTTCAAGGATCTAAAGGTTGATGATCAAATGAAGCTGCTCCAGCACGCCTGGTCGGATTTGTTAATTTTGGATCATTTGCATCAACGGTTGCACAATCACCTGCCTGACGAATCGTCACTGCCTAACGGACAGAAATTCGACCTTTTATCGCTATCGCTACTCGGTTGTCCATCACTGGCCGAACCCCTTCACGACGTTACTGCCCGGCTCACTGAAATACGTTTCGACGTGCCCGACTACGTCTGCCTCAAATTTCTCATGCTCCTCAATTCAGATGTCAAGGGATTGATGAATCGACGCCACGTGGTGGAAGCGCAGGAGCAAGTCCAACAAGCTCTTTTTGATTACACACTTAACTGCTACACTCATATCCCTGACAAGTTTGCTAAAATGCTGGCGATCTTACCGGACATTCACGCCATGTCGTCCCGCGGCGAAGAGTATCTCTATTTCAAGCATCTCAACGGCTGTGCGCCGACACAAACATTACTCATGGAAATGCTTCACGCCAAGCGGAAATGACGAGAGGCATTTTAAACAAAAAACCATCGCCAGGTAAGATGCATTTTATTTTTGCTCTCAAGTCAACTCATTTCTGTTCTGTATTTATTTATTTGTGAACGTTTTTCTTGGATTTTTTTTTTTTCTCTTAGATATGAGAATGTGAGTGTGATTGAATGTGTGTGAGTGCGTGTCTCTCTCGAATGAAATTCGGAGATATTGGGAGAAACTATTAAAAAAACAAAAC

##### >LC105701.1

CGGTCCGTCTCTCTGACTAGTCGGTGCTGCCCTTTTGTCCGACAATTAAAGTGCATATGATTCTCGTGTTGTTTTGTGCTGTACGACCGCCGTCGTTGTGTGTGTGAATTCTATTTATTTTTCCTATCGGTTGTCATCGCATGTGCCGCCCCTGTGTGTTTCGGAATTGCGGTCAACTTTCGCTTTCGGAGCGCCAACAACGCCATCAAGCGGGCAGGTGGTTTCTATAGTGTGTGACTTGTCCTCAATTTTTCCCTCTCTCTCTCTCTTGCTTCCAAACACACACACACATACAGACACGCACAGGAATCTTGTGTGTGAGGATTACAATTCTTTTTGAAAGACTTTTTTTTTTTCGTCTGTGCGTGTGCGTTCTCTTTCCATACCCATAGTGCAACAGGTGTGTGTGTAGCCCTCTGGTGAGCGTTTTTTCGTGCTGCGTATAGCCGCGTTGATTATCATTTTTTTCAAATCTTGTTTAAAAACAATTGTGTGAGATCTCATGTCTATTGGTTGAGATGCTAGTTGACATGGATCTCGCAGCTATTGCACCACTCGGAACCAACAGTTCGTCCGGTTTCATAAACTTGTCAGGCCCATCAGTCGATACGGACCAGGTCAGTGTCCAAATCGAACGTTTACACAGCCCCAACGGTGGTGGTGGTGGTGGTGGTGGTGGTTCAAACAGTGGCAATGGACCCAGTGGCCAGCATCATCACATGGTTGGCCATCCACAGCAACAACAACAACAACTCCATCATCATCACCATCACCACCACCACCTAGCGGGAGCTCAGCATTTGCATCATAGGACGCTGAGCTCTCCGAATTCTGAATTGTCGATTGCTTCTCTTTCAATGGCTTCATCCAGTCCCGCGTCTACCATGTCGCCGTTCAGTGCCAGTAGCAATAACAATAATAATAACAACAACAACAGTAGCTCGTCGGCGGCCATGAGCTATGCAATGAGTCACTTGAGCTCTGGTGGCGTCGAGGCCGGCGGCAATCTACTGGGTGTGGGCGTGACAGTCGGCCTGACTGGGCTCGATTCCTATTGCATTTACAATCCGAATAACGCAGGCGAGTACGAAACGAGCAATGCGGCCAGCAGCAACGCGGCCCTCAGTCCATCACCTCTCATGGATCCTATCGTCATTGTTCCATCCATGTCTGGCCAATCATCGGCCAGCTCTTTACCACCTGGAACGAGCTCGGCTTCGGGACGACACGACCTGCCCGATACGAAAGAAGGCATCGAAGAATTGTGTCCGGTTTGCGGCGATAAAGTCTCTGGCTATCATTACGGTCTGCTCACCTGCGAATCGTGCAAGGGATTCTTCAAACGGACCGTTCAGAACAAAAAAGCCTATTCTTGCGTCGCTGATCGATCCTGCCACATTGACAAATCACAACGCAAGAGGTGCCCTTATTGCCGATTCCAAAAGTGCCTTGAAGTCGGCATGAAACTTGAAGCTGTGAGAGCGGACCGAATGCGTGGCGGTCGGAATAAATTTGGACCCATGTACAAACGAGATCGAGCGCGTAAATTGCAGGTGATGCGTGAAAGACAATTGACGACGCCTCGAGGTAGCAACGGTGGAGGTAGCAATCCGTCACCCAACAACAGCAACAGCGGAGCAGGGCAGTCTGGAATGTACACGGACATGGGCTATTCTCCGTCCGGCAGCAGCGTCTACGGTGGTGGCAGCAGTGGCGGTGGTGTCAAGCACGAAATTCAAATTGCCCAAGTTTCTTCGTTGACATCCAGTCCCGATTCCAGCCCGAGTCCACTTGCCGCATCACTCGGCTATCCAGGACCTTCTGGCCAGCCGTTGGGCTCGTTGAACGGTTCTACGGGACCATCCGGATCAGGCGGCCCGCACAATAACAATAATACAAATAACCACAATAATAATAACATGGGCACGACATCGGGAGCGGGACCCTCTCATCCGCACACACCTTCTCCAAATAATCACCCGTCCGGCGGAGGAGGAGGAGGAGGCGCTCCTACACCCCAACAGCATTCCTCCACTTCATCCGTCTCACCGAAAACCTTCCATTTTGAGGGACTGTTGACTGGTAACACAATGCTGGGTAATAACAACGTTCCCAATGTCGGAGCCGGATCAGTTAGCGGGGCTAAAGTTCCGCCATTGATCCGCGAATTTGTCCAGTCTCTTGACGATAAAGAATGGCAAAGCGCTTTGTTTGGTCTTCTTCAGAGTCAAACCTACAATCAATGTGAAGTGGATTTGTTTGAACTGCTGTGTAAGGTGCTGGACCAAAACCTCTTTACCCAAGTGGACTGGGCACGCAATTCCTATTATTTCAAGGATCTAAAGGTTGATGATCAAATGAAGCTGCTCCAGCACGCCTGGTCGGATTTGTTAATTTTGGATCATTTGCATCAACGGTTGCACAATCACCTGCCTGACGAATCGTCACTGCCTAACGGACAGAAATTCGACCTTTTATCGCTATCGCTACTCGGTTGTCCATCACTGGCCGAACCCCTTCACGACGTTACTGCCCGGCTCACTGAAATACGTTTCGACGTGCCCGACTACGTCTGCCTCAAATTTCTCATGCTCCTCAATTCAGATGTCAAGGGATTGATGAATCGACGCCACGTGGTGGAAGCGCAGGAGCAAGTCCAACAAGCTCTTTTTGATTACACACTTAACTGCTACACTCATATCCCTGACAAGTTTGCTAAAATGCTGGCGATCTTACCGGACATTCACGCCATGTCGTCCCGCGGCGAAGAGTATCTCTATTTCAAGCATCTCAACGGCTGTGCGCCGACACAAACATTACTCATGGAAATGCTTCACGCCAAGCGGAAATGACGAGAGGCATTTTAAACAAAAAACCATCGCCAGGTAAGATGCATTTTATTTTTGCTCTCAAGTCAACTCATTTCTGTTCTGTATTTATTTATTTGTGAACGTTTTTCTTGGATTTTTTTTTTTTCTCTTAGATATGAGAATGTGAGTGTGATTGAATGTGTGTGAGTGCGTGTCTCTCTCGAATGAAATTCGGAGATATTGGGAGAAACTATTAAAAAAACAAAAC

### Maxillopoda (Lepeophtheirus salmonis)


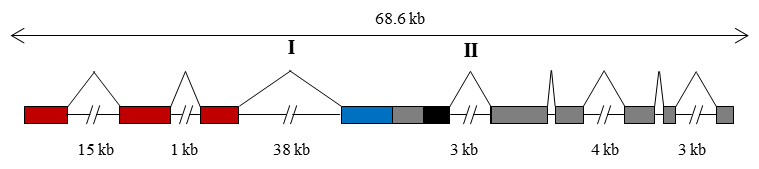


### Amphipoda (Hyalella azteca)


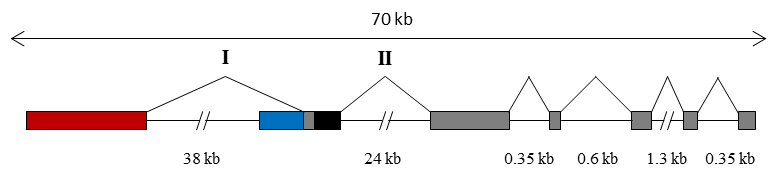


Many reads found in SRX685181

Read ID:  SRA:SRR1556043.212393440.2

Query 216 TTGCTTCCACCACCAGAAGTAACGATGTTATGCTGCTACTCGGACCTCTCATGTCTGGCG 275

||||||||||||||||||||||||||||||||||||||||||||||||||||||||||||

Sbjct 1 TTGCTTCCACCACCAGAAGTAACGATGTTATGCTGCTACTCGGACCTCTCATGTCTGGCG 60

Query 276 GAACCAATTCACGTAAAGATATATCCATTACGTCAAACGAT 316

|||||||||||||||||||||||||||||||||||||||||

Sbjct 61 GAACCAATTCACGTAAAGATATATCCATTACGTCAAACGAT 101

##### >XM_018152684.1

TGAAGGGTGGTGCGGTTGAGTTGCCCTAAAGACCTAGTTTTTACTTTCTACCTAACTTGGGGGCCTGCGCTCCCTCATGTCACTGAATTGTGCCGCAAGAGAAGACTCCTCGTGCTGTTGAAAGTGTTGTATCATGAGCGTTATACAGAAAGCCAAGGGAAGCAGGTATATTGAAGAGAAGGAGCCTAGCCGTGCTGCAACCTCGGCGACGCAAGTTGCTTCCACCACCAGAAGTAACGATGTTATGCTGCTACTCGGACCTCTCATGTCTGGCGGAACCAATTCACGTAAAGATATATCCATTACGTCAAACGATCATGTTAATGGCCATGAATATGCTCGGGAAATTAACGGTGTTTCGACTTTCCATTCATTGAGTTATTCGGATGAACCTCGCATTTCTGAACTAAAAAACGGTAACTGTTATACGCCTCGACATGGGATTGATACGAAGTACGAAACTAACAGTGTTCATAGTTCTCAAAGTGCAATCATTTATACGACAGAAAATGAAGTTTCTCGTCATTATCAATCTGAAGCTTCCACTAATAACATTGAAAAAGCTGATATATTGACCCACTCGTTGGTTACGGCAAATGCGCTAGACGATGATAACATGAACGCAAAAAATGATCCTGTTTTAGATCTGTCGACCCAAGAGTCTCATATATTTACTACTGACGGAATATCCATGAATCTTACTGACATGGTTGCTTTCGAGCTAAGCGCATCCGAGGACCATAATTCGAATCCTGACATAATGTCTGTTGTGACAACTCCAATTGCTGGACATGGACCAGACGCTTCCTTCAGTAATGTATCTGAAGATCACGTCTACTCCCCCATCATTGATCCCGTAGAAGCAGACAGAGTCTCTTTGTGTCATGAAGACTACTACCGTCATTCTCACGAACCTGCTTGTTGTACACCCTCTAGTTCTACGGCTCATTCCTCTCCTCTACCCATCCCTCTTCCCCGCGTTGCTAGCCGTGCTGAGTCTCTCACTCCTACCGACACTCCGGCTCATCGACAGACGGACTGCTCCTCATCCCTCCACCTCCGCACCCCTTCTCCACCCAGAGCCGCACCACCCCAGTCGCCTCTTAGTGACTCCTGCTCCCCACAACCTGCGGATGTGTCGCCAGATTCTCAAGGTGGAGGAAGCAGTGTTTCTGGTAACGCTACCGCCACCACTTCGCTCGACTACAGCAACGGATCAAGCATCGACATCCCTGACACAAAGGAAGGCATCGAAGAGCTCTGCCCAGTGTGTGGGGACAAAGTCTCAGGCTATCACTATGGCTTGCTTACGTGCGAGTCGTGCAAGGGGTTCTTCAAGAGAACCGTCCAGAACAAGAAGGTTTACACGTGCGTCGCTGATCGACAGTGCCAAATCGACAAGACCCAAAGGAAGAGGTGTCCTTATTGCAGATTCCAGAAGTGTATCGAGGTCGGCATGAAGCTTGAAGCTGTGCGGCAGGACCGGATGCGAGGAGGCAGGAACAAGTTCGGGCCCATGTACAAGCGTGACCGCGCGAGAAAGCTGCAGATGCTGCGTCAGCGTCAAATGACGCACCCAAATTTGTTGCCGTCTAGAAACGCGCCTGTGACATCAGGAGTCCAGTACACGTCACTCAGTTCTTATTCTACGGGTGGCTTACATATCAAAGAAGAGATCCAGAGTCCCTTCCTTTCTTCGTCTACCTCATCACCAGATTCCTCACCGGCACCTGTTAGGGTTCTTCAGAACTCCGGTGGACCGTCCCATCCCTCGCACAACTCCTCAGGACTCATTCTCGGCGCACCAGACAATATCGCTCTCTGGGTTGGTGCAGCTGGCAGTGGCGTAGGAGGTGGAGGTACAGGCGGAGGAGGAGGTGGAGGAAGCGGAGGTTCCCCGCAACAAGTCACTAGCAGATCTCTCAATAGAAACACCAATAGAATCCCCGACCTCGTTCGTGAATTATTGAATGCAGTTAACGATGCTGAATGGACCAATTCCCTCATGGGGCTTTTACAAAACCAAACTTATAATCAATGTGAAGTTGATCTCTTCGAGCTTATGTGCAAAGTCTTGGACCAGTCTTTGTTCGCCCAAGTTGACTGGGCAAGAAATTCCGTCTTCTTCAAAGATCTCAGGGTTGATGACCAAATGAAGTTGTTGCAAGAGTCTTGGTCGGATATGCTCCTTCTGGATCATCTCCATCAACGGTTACATAACAATATAGCCAACGACATGTCGCTCCCTAACGGCCAGAAGTTCAACCTGCTCAACTTAGCGCTGCTCGGCACTGATCAGTACGAGCAGAACTTCCATCAGCTGCTGTCAAAAATGGAAGACATGCAACTCGATGTTTCTGAATATATACTCATCAAATTTATTCTCCTACTTAACTCAGAGCCGTGCATCCGAGACAACAGACAGCTCACAGACCAGCAGAGCGTCGACGAGGCTGCCATGCAAGTGCGCAACGCTATGATGGAATATTGCGTCGTCAGCTACGGCGCCGCCACCGCCCAGGACCGGTACCAGAAGCTGCTTTCGCTCATATCGGACATTCATTTTATTGCGGACAGCGGCGAGAAGTTCCTGTACCTGAAGATGATGCACAGCGGCTCGTCCACTCAGACGCTGCTCATGGAAATGCTGCACACCAAACGCAGATAAAACCTGCCCCAGCAGCCACCTTCTCTCCTCTTTCTTTCATAAACGTCCATCAATCTCATGCACTTGCTCATATTTTATGTAAAAACTTCTGTTAATTGAGCAGTATTGTTAATGAAAACATCAGTCCGAATTTTTTGTTGATATTTTTTGTGCTCTGCGTTCCCGAAAGTTATTGAAAATTATTTTTATTTCCGTCACGTGTGCTTGACTTCATTAGGACTCTCATTTTGCAGAATTTCTTTCTTCAGCATCTATATTGACAACTACAAAACTGGAATCACAAATTGAGAAATGCTAGAGTAGTATAATAAATTACTAAATATTATAACTCCCGGAATAATATTACGAAATGAGAAAAAAAGTACGCGGAGGCGAGAGCAAAGAGACGCCCAAAATTGGACGTTGCGTCTCATTGAGAGCGTTACAGTTTATCCCCTTCTGTTGTGGATACAACGACTGTGGCGACTCTGTGTAATGTTTTAAGCTTATGATCTTAACACTGTAAATAAGTTTTGGTTTCTCACCATGCCCTGGTGGATCATTCACTTTACCAGAAATTATTTTAGTTAAGATTGACTCGATGTCACATCTTTTTTTTTTTTTTTGTGTGTGTGTTGCTTTGTGATCTTTTATTGTTTATTAAAAGTGCGATAACTAAGAAGTGAAATTCGCACGTTCTCTCAGCACAACAACAATATACTGTAAATAGTGAATCTGTAAGCCCATGATGATATTTGAAAGCTTTTATACTGTTGCCAAAAGGCTTCAAAATTTTGCCTTGTAGTTTTAAGGAATGAAATGAGTTACCTCCGGTAAAGTTTGAAACTGCTAGAACATTCAGCAATGTGTGTCACTCCTTGATCTCAGCGTGCACTTTATTACTATGTTAGGGTTAGAGATGACGGCTCTTCTTGGAAGGTTTGTTTTGACAACCGTTCAGTTATTCTCTACGGCCACATAGGCTCGGCGTTTGTTCTTGCTTTCGTCCGATTGGGTGATGAGGGTTGGTTTTGGTTATCGTCAATTACAATTGAAAAATTCCTTTTACCTTCAAGGAATAGTGGCGATTCTTCGTAGAAATGGAGGATAACACTCGGCATTTCTTCAGGCCTTTTCATGTCAATGATTATCTAATCTAATTCATGTTCATGCATCCATGAATAATAATTTTTTCTCATGTACAGTTGAAAATAAAATCTTTCGATTTGCTTTTCATAATTCGTTTTGAGAGATACACGAGTTTTAGCACTTAATTCAGATGAACGCTTCAATAATGGTGCCTTTGATTCTCAGTTGCTTTAGGAGATCTGCTCTCTTAGTTGTTACATTTCATGTTCAATCTCCTAGCGACCACTGGCAGTGAAGGAATACCTCGATCGCATCTAAAATCCTGTGAAAAAGAAGAGAATAATTCAGGGCCTGCATATACTCCAGTTGTTTGTTCTAAAATTGTTATAAGAAATTTATTGTTCATTAAATAAGGAGATTCCCCTGCTTTGGTTCGTTCTGATTAGGTTCTTATACAATGTGCAATATTATTGGCTACCTCGGAAGCCCTAATTTGTGTGATGGTCTAATTCTGTTGAGTCTATGAATTATTGAGAGAGAAAAAGAGACTTTATCAGAATTGTTTTACTGAAGCTTTTCTAGTTTGATAGTGAATCTACAAATTACGTTGAAGAAATTATAATTATATATAGATAGTTATTGTTATTATGTGCAATCTGGAATAAATTTGCTATTCCACTCTAGATGACACAAAATTACTTACTTAAATGTTATGACTAACTAAGCAGAATTTATTGACTTATGTATTTGGGATTATGTATTTTATGATGTAAAATTGTCTAAAATTCACTCAATGTTACTGGATTTTCAATCCTAGTGGATACCTAACATGTTGTTTTGTTATGATAAAATCCACAAAAATGACTAATCTGCGGAATTAGCTCGAGTATAATCTACAATGTGTGAAATAATTCATAAGTGGCATGTGCTGCTTACGTGTGTATTTTTCGACTTGGACCCTCAATACGTTAAATTAGACATTGAGTTTTCATCTCCTTGCACTTCATTAAGTTTCTGTGTGCAGAAATTCTGTATAGCCATGCTGTGACATCCCTCCCCGAGCGGCAGTTTGTTTGTTATTCATTCTAGGTTGTGTTTCCTCATGCAAGTAAAAATGTTGACAAGTCTCTATGTTTGTTGACGCTTAAATAGCTTTATTTCGTACACATCCATGCGATTCTGATAGGTCTGCATGTTTACATTCCAATTGTTTCACGAGTTGTCTTGCGGAGAGTTCTTTTCCACATATGAATATTTTATATTGTTCTATCTACAACTTAGTACTTACGCTTACCATTCTTCATCAATTGCTTGTCTAGCTGTGGTGAAGCCTTCTTCTCCTCATACTTTTGACTAAGAAATGCTTGAGAGTCGCAGTGTCTGCTCTCAAGTAAACGTTTAGCTTAATAATGATCCGTCTCGAGCTCCTTACACACTGTACATCGTTACCATAAGCTATAACAAAACTAAGAGATGCGTGCACCGCATTTAACCCTGTGGCCTGGCACATCTTTCATGCAAGTGCAAATTTCGATCCATTCTCATACGAAAAGATGGTCGCGATTTATAGTGTACAAGGAAAGACAAATTAATGTTCGCAAGTGCCTTTCGCGTTATATTGTGACTTAGCATATAGCTATTTAGGCTTATTCTATCTTTCCTCTACTTTATCTTACGTTCAGGTGTAGTTGTTGTGAGTGTAAACAAGAAAAATTGATTTGAACTAAAGATTTTTTGACCGATATTAGTATATATTAAATTGTATATAAATTCAGGTATGAAACGGTAGGTTAATTAAACCATGATCCTTTGACGTACAAACTGGCTATTGCAAACATTCATTGTTATACAATATCACTGAACATTGGAAAATTTACATGTTTCTAGAAGCGAGTTTAGATGCATTTTCAATTATTACATATGTCCAAAAGCTAAAAAAATCTTTATTCGAAACTACATGTTCAAAAATCTCTATTAGGAACATATTTTTATCAGAACTTAACTATCGCTCCACGTCCCACTACTAACCTAAGGTCCTATAATTCTATTGCAGCTAACTATCTTTCTTCTGATGTATTAAAAAGATGAGTGAACGTTGCAACGTAGACGCTACATGAGCCGAGTGATAAGTTCCTTGGATGTCATACTGCTATGTACAGACGTTGAGTTGGAGTAGCAAATTTATATTTTTTGATAATGGAATGTGTTATCTTAATCGGTAGTTTAATAATGTAGAAGAGTAAAGTTAAGCACAAGTTGTCAATAATTAACTTCATAAAATATGCGTACGTTGGCCCTCTATTGGAGACGGTCCCGCTGTTCACCTAACCTCCTCAAAATCACCAGCTATTAAAATCACTATTCAGCTGGTATATTCATACGGAACGGAATAAAAAACCTCAAATCCTAAAGAATGTTTGCTTTATTATCAGCGTTTCAATTAGAAGTTGCCCGTCTCTTCCATTAGGTTTAAATTACAGTGGCAAATTTTATGTCGTCCTCGACATTTTTAATCAGGACTTCTATTGGAACATCGGATTCGTTTCCCTTGATATTGAAAGCATCTGATTTAATGCAGTCATTAATTGTTACATTTCAAAGTTAAATGCAAATTTTTGCACCATCAGGCTTGCAAGCAATGCAAATCGACTATTCGTATAATAATTAGTTTAATAATTCGAGTGTTCGTATTCGCGACATAGCAGGAGCGTTGCATTCATTAGTGTCTAGCATCACAACGGGTAATATTTCTGCAATCTTAAATCTTAGTTTCGTACTCCTTCTATTTGAACAAGCGATATTTGGTCCATTTGTGAGATGCAGTTTTTGATTGTTACCTCTGCCTTGTGTCCTGTAGAAGTTATGACTGCTTATTTAATCCGATCGAATTGGTACATCTTTCATAAATCATGGCTGACCTGCATCATTTACTGTTTACTGTATTATTCATTTCCATGAATCTTTAATGAGAATTATATTCTCTTCTATACTTAGATTTATTTCTGTATTTTCATGCTGCAAAAGATATCTCTTAAAATTCCCTGCGTCATTTTCTCTTACTTTATAGAATAATGTCGAGCATTTACCACCGCAGCCCACCACGAGTGACATTTGATAACAATGCAGGATGCCGCGTGGAGTGACAACATCCAGGCTTTAACAAAATAAATGAGTTTATTTCTCCCCGTGAGTGACCACTCGCTGATAATACATGTAAATATTAATAGCTTGTAAGCCAAAATCCTTTCGAAATGTTCAACTGTAAAAAAATCTTTAACGTTAAGAAGAAACGCTCTATAGCTGCGTGGACATGTATGTTTTTGACCGCCTGAAAATCCATTTCTAAAATTGATATGTATATTTGAGTGACAAGTAAGGCTACCATAGAGTACATTATGAATGTCCATTGAAATAAAAGTGTTTTATTTAGATAA

Reads found in SRX685181

READ ID:  **SRA:SRR1556043.153512737.2**

READ ID: **SRA:SRR1556043.179380194.2**

READ ID:  **SRA:SRR1556043.149685312.2**

Query 8 GAGGCATGGAGCTCCCAAATCCGGTCCACAATTATAATCTTTCACCTCAATTCGGACAAT 67

||||||||||||||||||||||||||||||||||||||||||||||||||||||||||||

Sbjct 1 GAGGCATGGAGCTCCCAAATCCGGTCCACAATTATAATCTTTCACCTCAATTCGGACAAT 60

Query 68 TGACCATTACTTCACCAATGAATATAGACATGAATTCACTA 108

|||||||||||||||||||||||||||||||||||||||||

Sbjct 61 TGACCATTACTTCACCAATGAATATAGACATGAATTCACTA 101

##### >bFTZF1_putative

ATGCTCGGAGGCATGGAGCTCCCAAATCCGGTCCACAATTATAATCTTTCACCTCAATTCGGACAATTGACCATTACTTCACCAATGAATATAGACATGAATTCACTAGGCATCACAACAACCGCTATATCGCCATCTTTTGGTCACATTACTACATCAATGGGTGGGCAGGTACACTCCGTTGATTCGGCTAACACGATCTCACTCAACAACTCATCTCCACATCACCAACACCCTGTCGTAGCTGTTGCTTCGGCCTTACACCATCAGCAACATCACCAACAACAACACCAACATCAGCAGCAACAACAACATGTAGGTGGTTTACTAAGCCCTGGTCTAATGGATACTTCTTCTGGCATGTTTGCTGGCGCGTCAGGTGGAGGAAGCAGTGTTTCTGGTAACGCTACCGCCACCACTTCGCTCGACTACAGCAACGGATCAAGCATCGACATCCCTGACACAAAGGAAGGCATCGAAGAGCTCTGCCCAGTGTGTGGGGACAAAGTCTCAGGCTATCACTATGGCTTGCTTACGTGCGAGTCGTGCAAGGGGTTCTTCAAGAGAACCGTCCAGAACAAGAAGGTTTACACGTGCGTCGCTGATCGACAGTGCCAAATCGACAAGACCCAAAGGAAGAGGTGTCCTTATTGCAGATTCCAGAAGTGTATCGAGGTCGGCATGAAGCTTGAAGCTGTGCGGCAGGACCGGATGCGAGGAGGCAGGAACAAGTTCGGGCCCATGTACAAGCGTGACCGCGCGAGAAAGCTGCAGATGCTGCGTCAGCGTCAAATGACGCACCCAAATTTGTTGCCGTCTAGAAACGCGCCTGTGACATCAGGAGTCCAGTACACGTCACTCAGTTCTTATTCTACGGGTGGCTTACATATCAAAGAAGAGATCCAGAGTCCCTTCCTTTCTTCGTCTACCTCATCACCAGATTCCTCACCGGCACCTGTTAGGGTTCTTCAGAACTCCGGTGGACCGTCCCATCCCTCGCACAACTCCTCAGGACTCATTCTCGGCGCACCAGACAATATCGCTCTCTGGGTTGGTGCAGCTGGCAGTGGCGTAGGAGGTGGAGGTACAGGCGGAGGAGGAGGTGGAGGAAGCGGAGGTTCCCCGCAACAAGTCACTAGCAGATCTCTCAATAGAAACACCAATAGAATCCCCGACCTCGTTCGTGAATTATTGAATGCAGTTAACGATGCTGAATGGACCAATTCCCTCATGGGGCTTTTACAAAACCAAACTTATAATCAATGTGAAGTTGATCTCTTCGAGCTTATGTGCAAAGTCTTGGACCAGTCTTTGTTCGCCCAAGTTGACTGGGCAAGAAATTCCGTCTTCTTCAAAGATCTCAGGGTTGATGACCAAATGAAGTTGTTGCAAGAGTCTTGGTCGGATATGCTCCTTCTGGATCATCTCCATCAACGGTTACATAACAATATAGCCAACGACATGTCGCTCCCTAACGGCCAGAAGTTCAACCTGCTCAACTTAGCGCTGCTCGGCACTGATCAGTACGAGCAGAACTTCCATCAGCTGCTGTCAAAAATGGAAGACATGCAACTCGATGTTTCTGAATATATACTCATCAAATTTATTCTCCTACTTAACTCAGAGCCGTGCATCCGAGACAACAGACAGCTCACAGACCAGCAGAGCGTCGACGAGGCTGCCATGCAAGTGCGCAACGCTATGATGGAATATTGCGTCGTCAGCTACGGCGCCGCCACCGCCCAGGACCGGTACCAGAAGCTGCTTTCGCTCATATCGGACATTCATTTTATTGCGGACAGCGGCGAGAAGTTCCTGTACCTGAAGATGATGCACAGCGGCTCGTCCACTCAGACGCTGCTCATGGAAATGCTGCACACCAAACGCAGATAAAACCTGCCCCAGCAGCCACCTTCTCTCCTCTTTCTTTCATAAACGTCCATCAATCTCATGCACTTGCTCATATTTTATGTAAAAACTTCTGTTAATTGAGCAGTATTGTTAATGAAAACATCAGTCCGAATTTTTTGTTGATATTTTTTGTGCTCTGCGTTCCCGAAAGTTATTGAAAATTATTTTTATTTCCGTCACGTGTGCTTGACTTCATTAGGACTCTCATTTTGCAGAATTTCTTTCTTCAGCATCTATATTGACAACTACAAAACTGGAATCACAAATTGAGAAATGCTAGAGTAGTATAATAAATTACTAAATATTATAACTCCCGGAATAATATTACGAAATGAGAAAAAAAGTACGCGGAGGCGAGAGCAAAGAGACGCCCAAAATTGGACGTTGCGTCTCATTGAGAGCGTTACAGTTTATCCCCTTCTGTTGTGGATACAACGACTGTGGCGACTCTGTGTAATGTTTTAAGCTTATGATCTTAACACTGTAAATAAGTTTTGGTTTCTCACCATGCCCTGGTGGATCATTCACTTTACCAGAAATTATTTTAGTTAAGATTGACTCGATGTCACATCTTTTTTTTTTTTTTTGTGTGTGTGTTGCTTTGTGATCTTTTATTGTTTATTAAAAGTGCGATAACTAAGAAGTGAAATTCGCACGTTCTCTCAGCACAACAACAATATACTGTAAATAGTGAATCTGTAAGCCCATGATGATATTTGAAAGCTTTTATACTGTTGCCAAAAGGCTTCAAAATTTTGCCTTGTAGTTTTAAGGAATGAAATGAGTTACCTCCGGTAAAGTTTGAAACTGCTAGAACATTCAGCAATGTGTGTCACTCCTTGATCTCAGCGTGCACTTTATTACTATGTTAGGGTTAGAGATGACGGCTCTTCTTGGAAGGTTTGTTTTGACAACCGTTCAGTTATTCTCTACGGCCACATAGGCTCGGCGTTTGTTCTTGCTTTCGTCCGATTGGGTGATGAGGGTTGGTTTTGGTTATCGTCAATTACAATTGAAAAATTCCTTTTACCTTCAAGGAATAGTGGCGATTCTTCGTAGAAATGGAGGATAACACTCGGCATTTCTTCAGGCCTTTTCATGTCAATGATTATCTAATCTAATTCATGTTCATGCATCCATGAATAATAATTTTTTCTCATGTACAGTTGAAAATAAAATCTTTCGATTTGCTTTTCATAATTCGTTTTGAGAGATACACGAGTTTTAGCACTTAATTCAGATGAACGCTTCAATAATGGTGCCTTTGATTCTCAGTTGCTTTAGGAGATCTGCTCTCTTAGTTGTTACATTTCATGTTCAATCTCCTAGCGACCACTGGCAGTGAAGGAATACCTCGATCGCATCTAAAATCCTGTGAAAAAGAAGAGAATAATTCAGGGCCTGCATATACTCCAGTTGTTTGTTCTAAAATTGTTATAAGAAATTTATTGTTCATTAAATAAGGAGATTCCCCTGCTTTGGTTCGTTCTGATTAGGTTCTTATACAATGTGCAATATTATTGGCTACCTCGGAAGCCCTAATTTGTGTGATGGTCTAATTCTGTTGAGTCTATGAATTATTGAGAGAGAAAAAGAGACTTTATCAGAATTGTTTTACTGAAGCTTTTCTAGTTTGATAGTGAATCTACAAATTACGTTGAAGAAATTATAATTATATATAGATAGTTATTGTTATTATGTGCAATCTGGAATAAATTTGCTATTCCACTCTAGATGACACAAAATTACTTACTTAAATGTTATGACTAACTAAGCAGAATTTATTGACTTATGTATTTGGGATTATGTATTTTATGATGTAAAATTGTCTAAAATTCACTCAATGTTACTGGATTTTCAATCCTAGTGGATACCTAACATGTTGTTTTGTTATGATAAAATCCACAAAAATGACTAATCTGCGGAATTAGCTCGAGTATAATCTACAATGTGTGAAATAATTCATAAGTGGCATGTGCTGCTTACGTGTGTATTTTTCGACTTGGACCCTCAATACGTTAAATTAGACATTGAGTTTTCATCTCCTTGCACTTCATTAAGTTTCTGTGTGCAGAAATTCTGTATAGCCATGCTGTGACATCCCTCCCCGAGCGGCAGTTTGTTTGTTATTCATTCTAGGTTGTGTTTCCTCATGCAAGTAAAAATGTTGACAAGTCTCTATGTTTGTTGACGCTTAAATAGCTTTATTTCGTACACATCCATGCGATTCTGATAGGTCTGCATGTTTACATTCCAATTGTTTCACGAGTTGTCTTGCGGAGAGTTCTTTTCCACATATGAATATTTTATATTGTTCTATCTACAACTTAGTACTTACGCTTACCATTCTTCATCAATTGCTTGTCTAGCTGTGGTGAAGCCTTCTTCTCCTCATACTTTTGACTAAGAAATGCTTGAGAGTCGCAGTGTCTGCTCTCAAGTAAACGTTTAGCTTAATAATGATCCGTCTCGAGCTCCTTACACACTGTACATCGTTACCATAAGCTATAACAAAACTAAGAGATGCGTGCACCGCATTTAACCCTGTGGCCTGGCACATCTTTCATGCAAGTGCAAATTTCGATCCATTCTCATACGAAAAGATGGTCGCGATTTATAGTGTACAAGGAAAGACAAATTAATGTTCGCAAGTGCCTTTCGCGTTATATTGTGACTTAGCATATAGCTATTTAGGCTTATTCTATCTTTCCTCTACTTTATCTTACGTTCAGGTGTAGTTGTTGTGAGTGTAAACAAGAAAAATTGATTTGAACTAAAGATTTTTTGACCGATATTAGTATATATTAAATTGTATATAAATTCAGGTATGAAACGGTAGGTTAATTAAACCATGATCCTTTGACGTACAAACTGGCTATTGCAAACATTCATTGTTATACAATATCACTGAACATTGGAAAATTTACATGTTTCTAGAAGCGAGTTTAGATGCATTTTCAATTATTACATATGTCCAAAAGCTAAAAAAATCTTTATTCGAAACTACATGTTCAAAAATCTCTATTAGGAACATATTTTTATCAGAACTTAACTATCGCTCCACGTCCCACTACTAACCTAAGGTCCTATAATTCTATTGCAGCTAACTATCTTTCTTCTGATGTATTAAAAAGATGAGTGAACGTTGCAACGTAGACGCTACATGAGCCGAGTGATAAGTTCCTTGGATGTCATACTGCTATGTACAGACGTTGAGTTGGAGTAGCAAATTTATATTTTTTGATAATGGAATGTGTTATCTTAATCGGTAGTTTAATAATGTAGAAGAGTAAAGTTAAGCACAAGTTGTCAATAATTAACTTCATAAAATATGCGTACGTTGGCCCTCTATTGGAGACGGTCCCGCTGTTCACCTAACCTCCTCAAAATCACCAGCTATTAAAATCACTATTCAGCTGGTATATTCATACGGAACGGAATAAAAAACCTCAAATCCTAAAGAATGTTTGCTTTATTATCAGCGTTTCAATTAGAAGTTGCCCGTCTCTTCCATTAGGTTTAAATTACAGTGGCAAATTTTATGTCGTCCTCGACATTTTTAATCAGGACTTCTATTGGAACATCGGATTCGTTTCCCTTGATATTGAAAGCATCTGATTTAATGCAGTCATTAATTGTTACATTTCAAAGTTAAATGCAAATTTTTGCACCATCAGGCTTGCAAGCAATGCAAATCGACTATTCGTATAATAATTAGTTTAATAATTCGAGTGTTCGTATTCGCGACATAGCAGGAGCGTTGCATTCATTAGTGTCTAGCATCACAACGGGTAATATTTCTGCAATCTTAAATCTTAGTTTCGTACTCCTTCTATTTGAACAAGCGATATTTGGTCCATTTGTGAGATGCAGTTTTTGATTGTTACCTCTGCCTTGTGTCCTGTAGAAGTTATGACTGCTTATTTAATCCGATCGAATTGGTACATCTTTCATAAATCATGGCTGACCTGCATCATTTACTGTTTACTGTATTATTCATTTCCATGAATCTTTAATGAGAATTATATTCTCTTCTATACTTAGATTTATTTCTGTATTTTCATGCTGCAAAAGATATCTCTTAAAATTCCCTGCGTCATTTTCTCTTACTTTATAGAATAATGTCGAGCATTTACCACCGCAGCCCACCACGAGTGACATTTGATAACAATGCAGGATGCCGCGTGGAGTGACAACATCCAGGCTTTAACAAAATAAATGAGTTTATTTCTCCCCGTGAGTGACCACTCGCTGATAATACATGTAAATATTAATAGCTTGTAAGCCAAAATCCTTTCGAAATGTTCAACTGTAAAAAAATCTTTAACGTTAAGAAGAAACGCTCTATAGCTGCGTGGACATGTATGTTTTTGACCGCCTGAAAATCCATTTCTAAAATTGATATGTATATTTGAGTGACAAGTAAGGCTACCATAGAGTACATTATGAATGTCCATTGAAATAAAAGTGTTTTATTTAGATAA

## Priapulida

### Priapulida (Priapulus Caudatus)


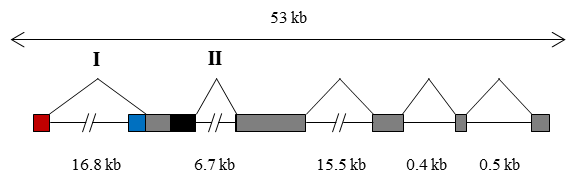


##### >FTZF1_priapulida_variantX2_XM_014816471.1

GAATTTGACGATTTCTTACTATTTCAGACATGACATTTCAATATAGTAAATGATGGAGACGTCCCCTGGGGATGAGTCAACGAGAGGAAGCCCACTTTCGCCACTGCTGGCTGGAGGCGTGTCCACCAGCCTCACCAGATCTGGAGATAACCCGATGCTGTTTCAGGACCACACAGACCAGTGCGGCAGCGATCCGGACGGGGAGAGCGTCTCCGACCACCAGCCGCAGCAGCAGCAGCCGCAACAGGCGCAGCAGCAGCAGCAGCTAGCGCTGCAGCAGCACTTCCCCGGCCAuCACCAGTACCCGTTGTCTGGCTTCAGCCCCGGCATGGCTTCGGCGGCGTCGGACTTTACGGACGCGCCCGACACGAAGGAGGACGTTGAAGAGGCGTGCCCCGTGTGCGGCGATCGCGTGTCCGGCTACCACTACGGACTACTCACCTGCGAGTCGTGCAAGGGATTCTTTAAGCGGACTGTGCAGAATAAGAAAGTGTACACGTGTGTCGCCGAGAAAAAGTGCTTGATAGACAAGACGCAGCGGAAACGTTGCCCCTACTGCCGCTTCCAGAAGTGCCTCGAGGTCGGGATGAAGCTGGAGGCCGTTCGTCCCGACCGCATGCGCGGAGGCCGAAACAAGTTCGGCCCGATGTACAAGCGCGAGCGGGCGCTGAAGCTGCAGGCGCGGCGCATGGCTGTTGCTGCACAGATGGGCGCCACCTGCCAGCTGAACGGCGTCGTCAAGTCGCTCGCCGCGTTCCCGTCCTTCCAGCAGGACAGCAAGTCGGACATTCAGATACCGCAGCTGAGCTCGTCAACGAACTCGCCGGACTCGTCCGCGCTCGATGACGCGCACAACAACAACATCAAGGAGGAGGGCGCCGGTGGTGGTGGCGGCGGAGGAGGCGCGTCTGCTGGCAGCGGGGCGGCGCGCGAGAGCAAGCTGTGGCAGTACTATCCGACCGTCACCAAGGTGTTGAACAACGCACCGCAGAAGGTGCCGCAGGTGATCCGCGAGCTGTCGGCGAGCACGATCGACGACAAGACCTGGCAGGAGTCGCTCTTCTCGCTCATCAACTCGCAGACATACAACCAGTGCGAGGTGGACCTGTTCGAACTGATGTGCAAAGTGCTCGACCACAGCCTTTTCTCGCTCGTCGAGTGGGCGCGGCGCTCCTACTTCTTCAAGGATCTAAAGGTTGAGGACCAGATGAAGTTGCTGCAGTTGTCGTGGTCCGAGTTGCTTATCCTCGACATGGTCTACCATCAGCTGCACAACTCGCTGCCCAATGAGACGCTGCTGCCCAACGGTCAGAAGTTCTCGCTGCTCAACCTGACGCTGTTTGGATTTTCCAACATGGCGGAGCGACATCACGAGCTGCTACAGCGGCTGCGACAGATCAAGTTCGATCGCAACGAGTATCTGTGCATCAAGTTCATCATCCTGCTGAATCCAGACGTGAAGGGGCTGACAAATAGCAAGCTGGTCGAAGAAGCTCAGGAGAAGGTCAACAATGCGCTCATGGAGTATTGCTCCAGCTTCTATCCAGAAGTGGAAGACAAGTTCGGACAGATCTTGCTGCGTCTGCCGGAGCTGAGGCTCATCAGCATTCGTGGCGAGGAGTTCCTTCACTTCAAGCACCTCAGTGGCGGCCTGCCCACCCAGACCCTCCTCATGGAGATGCTTCACGCCAAGCGGCGGCTCTGAGGGTGCACACAAGCGCACCCGAGAGGTGTCAGACTCATGGTGTCCTGTGGGCGCATCGTGGCGCCACCTAGGCACGCAGCCTACTCTGCGGAAGTGTATCCCGCCGAGTTGGGTGACGTCCGTGTCGCTGGGGTCGCTGCTCGCGGGCGTGACTCGATAGCTAGAACAACTCTGGCCTTCACCACAGCAGCAGGTGACGGCAGCAGAAGCCGCTACTCGCCCTGGTAGTTGCGTGGAACCGCGGGAGAACACGGTGCCACTGTGCTGGAGGTTTGCTGGACACGAGCCACCACCGAGTTTAGCCGCGTGCTTTGTGCGTGCGTCGGAGTAAATTCGCGTGCTGGTAATTTTGTTGGCAGAGGTTTTTAGTACTGACAAGATAACGATTAGTGAGAATTTGTGAGGGATGGTGAACTTCCCGTGGGCTGGTGATCGCAATCAAATAAACTGAACATTTGCGCTGCCAGCATTTCTTATGTTGGACTTGGAAGGTTCTGCAATCGACATGATTATCGATGGTTGAGGCCAGACACTCTGCGTAAGGCAACAAGCAACAAGCTAGACAGAGAGAGAAAATGAGGGGAGTGTCTGTGGCAGCGACCAGCAGCTTGTGCATAGCCAATCACGCAACACTTTTACATTTGTTTATTTTGAAATGGAGGTTCAAAATGAACTATAGAGTGTTGCGAAAAATGATTTTTAGTAGTTAGGTAATGAACAAATGTCTTAGGAAAGATGTCGATGGAATGATGTGTGGGATGGCAGTTGGTCTCAGCATTGCATCCAGGTGAGAATCTAAGCCAGTTATCCAGAGCCATTGATTAGTTGTGCCTGGTAGGTGGAGCTGAGAACGTGACAGGTACGTGCGTGAGAGCTCAGCCAACCAAAATCTTTTGATACCAAAAATAAATAGCGCCTAATCGACACAACATCCACTCCACATTCACAGAGATTTATTTTTAATAGTGTTATATCTACAATACGTATATCACTCAGCTTGTACGAAATTCACTCATCACTTGAATTCATCCTGGCTGGCTATAGACTCGAATATGATATACCCCTTACACATGAATGTAAAATAAACCATACTACTGCAGTAGGTCTCAAACTATTTCATTACTATCGTATATGTGCAGCATGTACATATCGAGTGTATATTAGACTATTGCCTCATATACACTCGAACTTACGTAGATGTGGGTTCATGCCTGCGCCAATTATTCTCTAGTGATGACCCGGCTGTATTAGTGGTGTGGTAGGTTCCATCCAGGTAGATATTCTGTTTTCTCAGAGCATGCATACCAAACTGTGTGTGTTTGTGTATGGTCGACTTAAGGGTATGGAGTGGGGTGGGTTGGAAGGGAAAGGGAACAGAGTCAGAGCAAGACATTGCAGTTGCGGTGCAGTTAAGCACACAGAGTGACTGAGGTGGCGGCTTTCATCGGGGTACGTTTACAATAGATAGCTCTGGGTGTGTGTATGTGTGTGTGTGTGTGTGTGTGGGTGGGTGTGTGTGTGTGGGTGGGTGTGTGTATGTGTGTGTGTGTGAACGTGCGTGTGCGTGCGTGCGTGCGTGCGTGCGTGCGTGTGTTATTCACTTGGTTTTGTACTGTGATATTACGTTGTGTGATTAGAAATGATTCTGAAAAACTCGGGAACTGCTTCCTGCATATGAATGGGGGAGATGAGCCGCACCAGTCTCGTTGCATGTCGCCTGAGAACAGAATATGTAGCGAGGATTAAAAACTTTGTTTCTTGTACTGCTACTGTGACTGCCTATCGCAGTGGCTGTTCCATTTGATCGCCTCTGTCCTCGTTAGCCAACCTTCGTACGGTTCCTGTAGTGGCGAACGTACCAGAACTCTTATTATTTAATACAACTGTAAGGTTTTATTACAAAATATACCACACTGTACCCAGCCTGTAGTATCAAACATACCACACTCCACGCCAGCCCATGGTATGACACTTCACCCCAACCTATAGTATAGAACATACCACACCGCAAACTGTAGTATAGAACATACCACACTGCACTCAACATATAGTATAGTACATACGACACTCCACCCAGCCTATAGTATAGTGCATACCACTACCCAAACCGTAGTATAGAACACACCACAATCCGACCAGGAGTAGGACATATATATATACAACACTCCACCCCAGCCTGTAGTATAGAACGTAGAGAACATACCACGCCGTAATCTTTAATAATAACTAATAAGGCAACCCTTGTCAGCTGTGCTGGTATTCTGCCTTGTATATATTGATGTAATAACGCGGTTAAACATATGGTATTCTGTTTTCTGTGACCATTAGCGGAATTTAGCCAGTGATGAATAATTCATAATTTAAGCCTGTGTAGATATTATCAAAGTTGGCTGGAGCACTATTTTTGTGATCGGAAAATGTAGTACTATGTGAACGCAATTGTTAACGGTGTTAGTATATCGACCACTGGTGGATGACGTGATGCCTTTACATGGTTTTAGGATGGTAATGTTAGTTTGACAACTTGTGTTAGTTTCGCACTGACAGATTATTTGTCGTCCGTGTTGCGTGGACGACTTAATGCACGTGTAGCTAATTTAGGATCTGGGATAGCATGAAATTTGCATGAGTGGATTAATACAGAAACTGCATGGTCATTCATTAATTACCACGTTTCACTGGATTAATCAAAACCCATTTCCTGTGTCCTTTATCGCCATCTCGCTGTATGTAGTACATTTTTACATAGCTGAGGCACAAGGCAAACTTTATAGATTGATATTTGTATCTAGGCTGCAGGCTGGCCTATTATTGTTTCCCACAGTGCTTCAGATCCGTGGCCACCGTACAAACACAGTGCTAGCTGGCTGATCTAGAAGACGCGGTCGTGTTTGTAAATCAATTGGACAGTTGTAAAGATTTATGATAACGTTCGAAGTTCATGGTACGACTTGTGTGGCAATTCGCATAGACGTCATTCGCGTTACCGAAATAAGGAGATGTATATTTTTAGAATCGAACGACGCGTGCGTGCCCGAATTCTCGCGCAGACGATAAAGTGCCAGTATGTCATGTGATGTATCGCGACATTTACGGTCCTGTCGCTGCGTGCGACCAGCTGACAGCATATTGCTACGAGTTTTAGAACAGTATCTTGCAGCGACTCGCACGTTTCAAATCGACAACGGATCATATCATAGGCATAGTGATTGCTGAGTTTATATTGTCATTTTGTTCATGTGTTCGTGTATGAAGTGATTCCACAAACACCGGTGGATCTCCGCATTGTACATTTTTTGTTGGAATGATTTATGAAAATGTACATTTTCATATTATTTGAATCGTTTATAGCCAGTGCTGTATATAATTTACAGATATATACTGTACACACAATTGTTTAGTCATTTATCATGTTTTCACTATTTTAAAGTATATTTCCTTTAATGACTTAGTAGTTCACAGCTAGAGCGGTCTGTAGAATAGATCTATCATAGATTGTAGATAAGAACATTATTTTGTGTTACTTTATATTAGCAGATGGCACGTGACAGTGCATCTGTCATCCATTCTCAATTCTCTGCGAGTCGTTTATAATGGTGTACAGACATGTCAACAGAGAAATAAATAGGCATTACTAACAGAAA

##### >bFTZF1_priapulida

ATGGATTACAACTCTAGCGTCATGGGCCACGCCGGCTCGTCGAGTGATAACGGCACTATGGTTACATACCCGTACCAAGACATAGACTTCTTTAGTCACGATCACCACCATGCCGCGCTCAATCACGGCATGTTTGTTATTCCTGCAGGCGGCAGCGATCCGGACGGGGAGAGCGTCTCCGACCACCAGCCGCAGCAGCAGCAGCCGCAACAGGCGCAGCAGCAGCAGCAGCTAGCGCTGCAGCAGCACTTCCCCGGCCAGTACCACAACCACCAGTACCCGTTGTCTGGCTTCAGCCCCGGCATGGCTTCGGCGGCGTCGGACTTTACGGACGCGCCCGACACGAAGGAGGACGTTGAAGAGGCGTGCCCCGTGTGCGGCGATCGCGTGTCCGGCTACCACTACGGACTACTCACCTGCGAGTCGTGCAAGGGATTCTTTAAGCGGACTGTGCAGAATAAGAAAGTGTACACGTGTGTCGCCGAGAAAAAGTGCTTGATAGACAAGACGCAGCGGAAACGTTGCCCCTACTGCCGCTTCCAGAAGTGCCTCGAGGTCGGGATGAAGCTGGAGGCCGTTCGTCCCGACCGCATGCGCGGAGGCCGAAACAAGTTCGGCCCGATGTACAAGCGCGAGCGGGCGCTGAAGCTGCAGGCGCGGCGCATGGCTGTTGCTGCACAGATGGGCGCCACCTGCCAGCTGAACGGCGTCGTCAAGTCGCTCGCCGCGTTCCCGTCCTTCCAGCAGGACAGCAAGTCGGACATTCAGATACCGCAGCTGAGCTCGTCAACGAACTCGCCGGACTCGTCCGCGCTCGATGACGCGCACAACAACAACATCAAGGAGGAGGGCGCCGGTGGTGGTGGCGGCGGAGGAGGCGCGTCTGCTGGCAGCGGGGCGGCGCGCGAGAGCAAGCTGTGGCAGTACTATCCGACCGTCACCAAGGTGTTGAACAACGCACCGCAGAAGGTGCCGCAGGTGATCCGCGAGCTGTCGGCGAGCACGATCGACGACAAGACCTGGCAGGAGTCGCTCTTCTCGCTCATCAACTCGCAGACATACAACCAGTGCGAGGTGGACCTGTTCGAACTGATGTGCAAAGTGCTCGACCACAGCCTTTTCTCGCTCGTCGAGTGGGCGCGGCGCTCCTACTTCTTCAAGGATCTAAAGGTTGAGGACCAGATGAAGTTGCTGCAGTTGTCGTGGTCCGAGTTGCTTATCCTCGACATGGTCTACCATCAGCTGCACAACTCGCTGCCCAATGAGACGCTGCTGCCCAACGGTCAGAAGTTCTCGCTGCTCAACCTGACGCTGTTTGGATTTTCCAACATGGCGGAGCGACATCACGAGCTGCTACAGCGGCTGCGACAGATCAAGTTCGATCGCAACGAGTATCTGTGCATCAAGTTCATCATCCTGCTGAATCCAGACGTGAAGGGGCTGACAAATAGCAAGCTGGTCGAAGAAGCTCAGGAGAAGGTCAACAATGCGCTCATGGAGTATTGCTCCAGCTTCTATCCAGAAGTGGAAGACAAGTTCGGACAGATCTTGCTGCGTCTGCCGGAGCTGAGGCTCATCAGCATTCGTGGCGAGGAGTTCCTTCACTTCAAGCACCTCAGTGGCGGCCTGCCCACCCAGACCCTCCTCATGGAGATGCTTCACGCCAAGCGGCGGCTCTGAGGGTGCACACAAGCGCACCCGAGAGGTGTCAGACTCATGGTGTCCTGTGGGCGCATCGTGGCGCCACCTAGGCACGCAGCCTACTCTGCGGAAGTGTATCCCGCCGAGTTGGGTGACGTCCGTGTCGCTGGGGTCGCTGCTCGCGGGCGTGACTCGATAGCTAGAACAACTCTGGCCTTCACCACAGCAGCAGGTGACGGCAGCAGAAGCCGCTACTCGCCCTGGTAGTTGCGTGGAACCGCGGGAGAACACGGTGCCACTGTGCTGGAGGTTTGCTGGACACGAGCCACCACCGAGTTTAGCCGCGTGCTTTGTGCGTGCGTCGGAGTAAATTCGCGTGCTGGTAATTTTGTTGGCAGAGGTTTTTAGTACTGACAAGATAACGATTAGTGAGAATTTGTGAGGGATGGTGAACTTCCCGTGGGCTGGTGATCGCAATCAAATAAACTGAACATTTGCGCTGCCAGCATTTCTTATGTTGGACTTGGAAGGTTCTGCAATCGACATGATTATCGATGGTTGAGGCCAGACACTCTGCGTAAGGCAACAAGCAACAAGCTAGACAGAGAGAGAAAATGAGGGGAGTGTCTGTGGCAGCGACCAGCAGCTTGTGCATAGCCAATCACGCAACACTTTTACATTTGTTTATTTTGAAATGGAGGTTCAAAATGAACTATAGAGTGTTGCGAAAAATGATTTTTAGTAGTTAGGTAATGAACAAATGTCTTAGGAAAGATGTCGATGGAATGATGTGTGGGATGGCAGTTGGTCTCAGCATTGCATCCAGGTGAGAATCTAAGCCAGTTATCCAGAGCCATTGATTAGTTGTGCCTGGTAGGTGGAGCTGAGAACGTGACAGGTACGTGCGTGAGAGCTCAGCCAACCAAAATCTTTTGATACCAAAAATAAATAGCGCCTAATCGACACAACATCCACTCCACATTCACAGAGATTTATTTTTAATAGTGTTATATCTACAATACGTATATCACTCAGCTTGTACGAAATTCACTCATCACTTGAATTCATCCTGGCTGGCTATAGACTCGAATATGATATACCCCTTACACATGAATGTAAAATAAACCATACTACTGCAGTAGGTCTCAAACTATTTCATTACTATCGTATATGTGCAGCATGTACATATCGAGTGTATATTAGACTATTGCCTCATATACACTCGAACTTACGTAGATGTGGGTTCATGCCTGCGCCAATTATTCTCTAGTGATGACCCGGCTGTATTAGTGGTGTGGTAGGTTCCATCCAGGTAGATATTCTGTTTTCTCAGAGCATGCATACCAAACTGTGTGTGTTTGTGTATGGTCGACTTAAGGGTATGGAGTGGGGTGGGTTGGAAGGGAAAGGGAACAGAGTCAGAGCAAGACATTGCAGTTGCGGTGCAGTTAAGCACACAGAGTGACTGAGGTGGCGGCTTTCATCGGGGTACGTTTACAATAGATAGCTCTGGGTGTGTGTATGTGTGTGTGTGTGTGTGTGTGGGTGGGTGTGTGTGTGTGGGTGGGTGTGTGTATGTGTGTGTGTGTGAACGTGCGTGTGCGTGCGTGCGTGCGTGCGTGCGTGCGTGTGTTATTCACTTGGTTTTGTACTGTGATATTACGTTGTGTGATTAGAAATGATTCTGAAAAACTCGGGAACTGCTTCCTGCATATGAATGGGGGAGATGAGCCGCACCAGTCTCGTTGCATGTCGCCTGAGAACAGAATATGTAGCGAGGATTAAAAACTTTGTTTCTTGTACTGCTACTGTGACTGCCTATCGCAGTGGCTGTTCCATTTGATCGCCTCTGTCCTCGTTAGCCAACCTTCGTACGGTTCCTGTAGTGGCGAACGTACCAGAACTCTTATTATTTAATACAACTGTAAGGTTTTATTACAAAATATACCACACTGTACCCAGCCTGTAGTATCAAACATACCACACTCCACGCCAGCCCATGGTATGACACTTCACCCCAACCTATAGTATAGAACATACCACACCGCAAACTGTAGTATAGAACATACCACACTGCACTCAACATATAGTATAGTACATACGACACTCCACCCAGCCTATAGTATAGTGCATACCACTACCCAAACCGTAGTATAGAACACACCACAATCCGACCAGGAGTAGGACATATATATATACAACACTCCACCCCAGCCTGTAGTATAGAACGTAGAGAACATACCACGCCGTAATCTTTAATAATAACTAATAAGGCAACCCTTGTCAGCTGTGCTGGTATTCTGCCTTGTATATATTGATGTAATAACGCGGTTAAACATATGGTATTCTGTTTTCTGTGACCATTAGCGGAATTTAGCCAGTGATGAATAATTCATAATTTAAGCCTGTGTAGATATTATCAAAGTTGGCTGGAGCACTATTTTTGTGATCGGAAAATGTAGTACTATGTGAACGCAATTGTTAACGGTGTTAGTATATCGACCACTGGTGGATGACGTGATGCCTTTACATGGTTTTAGGATGGTAATGTTAGTTTGACAACTTGTGTTAGTTTCGCACTGACAGATTATTTGTCGTCCGTGTTGCGTGGACGACTTAATGCACGTGTAGCTAATTTAGGATCTGGGATAGCATGAAATTTGCATGAGTGGATTAATACAGAAACTGCATGGTCATTCATTAATTACCACGTTTCACTGGATTAATCAAAACCCATTTCCTGTGTCCTTTATCGCCATCTCGCTGTATGTAGTACATTTTTACATAGCTGAGGCACAAGGCAAACTTTATAGATTGATATTTGTATCTAGGCTGCAGGCTGGCCTATTATTGTTTCCCACAGTGCTTCAGATCCGTGGCCACCGTACAAACACAGTGCTAGCTGGCTGATCTAGAAGACGCGGTCGTGTTTGTAAATCAATTGGACAGTTGTAAAGATTTATGATAACGTTCGAAGTTCATGGTACGACTTGTGTGGCAATTCGCATAGACGTCATTCGCGTTACCGAAATAAGGAGATGTATATTTTTAGAATCGAACGACGCGTGCGTGCCCGAATTCTCGCGCAGACGATAAAGTGCCAGTATGTCATGTGATGTATCGCGACATTTACGGTCCTGTCGCTGCGTGCGACCAGCTGACAGCATATTGCTACGAGTTTTAGAACAGTATCTTGCAGCGACTCGCACGTTTCAAATCGACAACGGATCATATCATAGGCATAGTGATTGCTGAGTTTATATTGTCATTTTGTTCATGTGTTCGTGTATGAAGTGATTCCACAAACACCGGTGGATCTCCGCATTGTACATTTTTTGTTGGAATGATTTATGAAAATGTACATTTTCATATTATTTGAATCGTTTATAGCCAGTGCTGTATATAATTTACAGATATATACTGTACACACAATTGTTTAGTCATTTATCATGTTTTCACTATTTTAAAGTATATTTCCTTTAATGACTTAGTAGTTCACAGCTAGAGCGGTCTGTAGAATAGATCTATCATAGATTGTAGATAAGAACATTATTTTGTGTTACTTTATATTAGCAGATGGCACGTGACAGTGCATCTGTCATCCATTCTCAATTCTCTGCGAGTCGTTTATAATGGTGTACAGACATGTCAACAGAGAAATAAATAGGCATTACTAACAGAAA

## Tardigrade

### Tardigrada (Hypsibius djuradini)


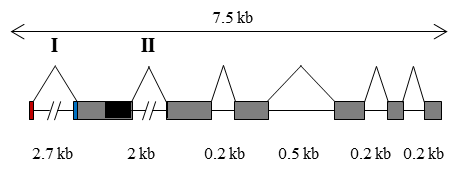


Read found in **SRX2528376**

Read ID: **SRA:SRR5218246.8855189.1**

Query 177 CGAAGAGAAAGGAATCGGAGCGGAAACATGGGAAGACGGCGTGCTAAAGCAGCTAAACGG 236

||||||||||||||||||||||||||||||||||||||||||||||||||||||||||||

Sbjct 1 CGAAGAGAAAGGAATCGGAGCGGAAACATGGGAAGACGGCGTGCTAAAGCAGCTAAACGG 60

Query 237 CGAAAAGGATCCGGTG 252

||||||||||||||||

Sbjct 61 CGAAAAGGATCCGGTG 76

##### >MTYJ01000049.1_hypsibius_djuradini_FTZF1

ATGGGAAGACGGCGTGCTAAAGCAGCTAAACGGCGAAAAGGATCCGGTGGAAGCCACGCCTTAACGGAACTGTCCTCTATCAACGGCGGCGGCGGGATGACACCCAGTCACCATCATCACTCCATCCCCTTCGGCCTGTCTTCCCTCTACAGCAACAGTTCACCCATTGGAGGAGGAACTGCTACCGGGTCGTCCTCCATATCGGGCGGGGACCTCTCAGACGAAAGCATCGCCACCAATCAACTTCAGCAGCAGCAACAGCAGCAGCAGCCTGACTCGGAAGTCGATATGGACCTGGAAGGGTGTCCGGTGTGTGGGGACAAGGTGTCCGGTTATCATTACGGCCTGTTGACCTGTGAGAGCTGTAAGGGATTCTTCAAGCGCACCGTCCAGAATAAGAAGGTCTACACGTGCAACGGGGAACGAGGCTGTCATATTGATAAGAGCAGCCGGAAGCGGTGTCCGTGTTGTCGCTATGAGAAGTGCATGCGGGTCGGAATGAAGCTGGAAGCGGTTCGCGAAGATCGGATGCGCGGAGGGCGGAACAAATTTGGCCCAATGTACAAACGTGACCGAGCACGGAAGCAGCAGCAACAACGAGTCCGCCACGTGAGTCCCCACCAGAGTTACCAGAGCCGGTATTACAAGAACTCCAACGGATGCAAAACGGAGAACGCCGGCTCATCCAGCATGTACATGTCCCCACCGCCCAGTTCGCTCTACGAGCCGGCCTTCCTTAAGACTGAGATGATTCAGATTCCACATTTAAGTTCGACGAATTCGCCGGAAAGTTTACAAATGTCCGGCGGCGAATCGAGCAGCAGCACCGTCAAGTCATGGTCTAACTCATCCTACTCACACTCACCACCTTTACAGTACAATCAGAACGGCATGATTGTGGACTTGATGAGCGGTATCCCACCACCCATCAGCTACTCCCAAGAGAACTTGAACGAGCCGGGTCTTCAGCCGTTTCCCGGCACCGTGGAGGAGTTGCGAGCCACCCTGGTGGACGAAGGTTTCTGGCAGCAGCGCCTGTTTGAGCTCCTCAAAAGCCAGACCTACAACCAGTGCTCGGTGGACCTGTTCGAGCTGCTGACCAAAGTTCTGGACCAGTGCCTCTTCTCGCAGGTGGAATGGGCACGCAACTCTGCATTCTTCAAGGATCTCAAACTGGAAGACCAAATGACTCTACTGCAGCATTCCTGGAGCGATTTGCTAATGCTTGATTTTATCCACCACCGCTTGAACAACGGCCTGCAAAGTACATTCAAACTTCCAAACGGCCAGTTTTTCCACCTGTGGGATCTGGCGCTTCTCGGCAACAACAGCAGCATGGACCATTTGCAGCGGTTGCTGGCGCGTTTTCAAGATCTTGACATGGATCGAGACGACTATATCTGTCTGAAGTTCATACTTCTGCTGGACCATGCTACACCTGGACTGAACGATCAACTGCACGTGCTAACTGGCCAGAACCGCGTGCAAAGCATCCTTCTCAGTCACTGCCGAGCAAAGTGTCCGGGCATGCCAGACCGGTACAGTCAGTTGATTCTTCAACTTCCGGATCTCCGCCTTTTGGCCAATCAGTGCGAAGAGTCGCTCTACCACAAACACATGACCGCTTCGGCCATATCCGACACGAGCCTCCTCATGGAGCTGCTCCTGTCCAAGCGAAAGAGTTTGCTGGAGCTGTCGACCGAATAA

Read found in **SRX2528376**

**Read ID:**  **SRA:SRR5218246.3998962.1**

Query 367 CAAAATGAACGATGCCTATTATTCCTTTCCCCCAGGATCCGGTGGAAGCCACGCCTTAAC 426

||||||||||||||||||||||||||||||||||||||||||||||||||||||||||||

Sbjct 1 CAAAATGAACGATGCCTATTATTCCTTTCCCCCAGGATCCGGTGGAAGCCACGCCTTAAC 60

Query 427 GGAACTGTCCTCTATC 442

||||||||||||||||

Sbjct 61 GGAACTGTCCTCTATC 76

##### >MTYJ01000049.1_hypsibius_djuradini_bFTZF1

ATGAACGATGCCTATTATTCCTTTCCCCCAGGATCCGGTGGAAGCCACGCCTTAACGGAACTGTCCTCTATCAACGGCGGCGGCGGGATGACACCCAGTCACCATCATCACTCCATCCCCTTCGGCCTGTCTTCCCTCTACAGCAACAGTTCACCCATTGGAGGAGGAACTGCTACCGGGTCGTCCTCCATATCGGGCGGGGACCTCTCAGACGAAAGCATCGCCACCAATCAACTTCAGCAGCAGCAACAGCAGCAGCAGCCTGACTCGGAAGTCGATATGGACCTGGAAGGGTGTCCGGTGTGTGGGGACAAGGTGTCCGGTTATCATTACGGCCTGTTGACCTGTGAGAGCTGTAAGGGATTCTTCAAGCGCACCGTCCAGAATAAGAAGGTCTACACGTGCAACGGGGAACGAGGCTGTCATATTGATAAGAGCAGCCGGAAGCGGTGTCCGTGTTGTCGCTATGAGAAGTGCATGCGGGTCGGAATGAAGCTGGAAGCGGTTCGCGAAGATCGGATGCGCGGAGGGCGGAACAAATTTGGCCCAATGTACAAACGTGACCGAGCACGGAAGCAGCAGCAACAACGAGTCCGCCACGTGAGTCCCCACCAGAGTTACCAGAGCCGGTATTACAAGAACTCCAACGGATGCAAAACGGAGAACGCCGGCTCATCCAGCATGTACATGTCCCCACCGCCCAGTTCGCTCTACGAGCCGGCCTTCCTTAAGACTGAGATGATTCAGATTCCACATTTAAGTTCGACGAATTCGCCGGAAAGTTTACAAATGTCCGGCGGCGAATCGAGCAGCAGCACCGTCAAGTCATGGTCTAACTCATCCTACTCACACTCACCACCTTTACAGTACAATCAGAACGGCATGATTGTGGACTTGATGAGCGGTATCCCACCACCCATCAGCTACTCCCAAGAGAACTTGAACGAGCCGGGTCTTCAGCCGTTTCCCGGCACCGTGGAGGAGTTGCGAGCCACCCTGGTGGACGAAGGTTTCTGGCAGCAGCGCCTGTTTGAGCTCCTCAAAAGCCAGACCTACAACCAGTGCTCGGTGGACCTGTTCGAGCTGCTGACCAAAGTTCTGGACCAGTGCCTCTTCTCGCAGGTGGAATGGGCACGCAACTCTGCATTCTTCAAGGATCTCAAACTGGAAGACCAAATGACTCTACTGCAGCATTCCTGGAGCGATTTGCTAATGCTTGATTTTATCCACCACCGCTTGAACAACGGCCTGCAAAGTACATTCAAACTTCCAAACGGCCAGTTTTTCCACCTGTGGGATCTGGCGCTTCTCGGCAACAACAGCAGCATGGACCATTTGCAGCGGTTGCTGGCGCGTTTTCAAGATCTTGACATGGATCGAGACGACTATATCTGTCTGAAGTTCATACTTCTGCTGGACCATGCTACACCTGGACTGAACGATCAACTGCACGTGCTAACTGGCCAGAACCGCGTGCAAAGCATCCTTCTCAGTCACTGCCGAGCAAAGTGTCCGGGCATGCCAGACCGGTACAGTCAGTTGATTCTTCAACTTCCGGATCTCCGCCTTTTGGCCAATCAGTGCGAAGAGTCGCTCTACCACAAACACATGACCGCTTCGGCCATATCCGACACGAGCCTCCTCATGGAGCTGCTCCTGTCCAAGCGAAAGAGTTTGCTGGAGCTGTCGACCGAATAA

## Nematoda

### Nematoda (Caenorhabditis elegans)


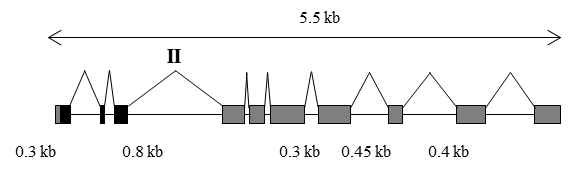


### Nematoda (Trichuris trichiura (CDW52832.1)


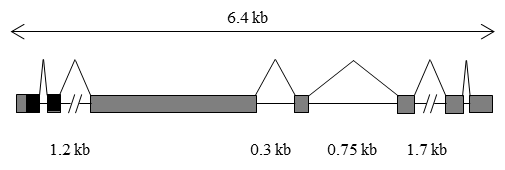


### Nematoda (Steinernema carpocapsae (TKR80278.1)


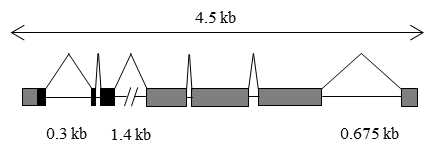

Supplement: S1 File — (DOCX) [file pone.0251575.s005.docx]
