# Supplementary material for: The FTZ-F1 gene encodes two functionally distinct nuclear receptor isoforms in the ectoparasitic copepod salmon louse (Lepeophtheirus salmonis)
Source: PLoS One. 2021 May 20;16(5):e0251575. doi: 10.1371/journal.pone.0251575 (PMC8136749; doi:10.1371/journal.pone.0251575)
Supplement: S1 Table — (DOCX) [file pone.0251575.s006.docx]

# **S1 Table. RT-qPCR primers used in this study.**

Sequences of all primers used in the study for rapid amplification of cDNA ends (RACE), Colony PCR, RNA interference (RNAi) and real time PCR (RT-qPCR) with their melting point (Tm), product length in base pairs (bp) and efficiency (E) where relevant. Fwd= forward, Rev= reverse. T7 promotor sites in the primers are underlined. + = to determine insert size in cloning vector, * product size of the transcript specific part, excluding T7 sites, ¤ = primers are specific for pBlueScript II SK (+) vector sequence containing the cod trypsin CPY185 sequence (Accession: XM_030370867.1)

| **Name** | **Sequence 5’ 🡪 3’** | **Method** | **Tm (℃)** | **Product (bp)** | **E** |
| --- | --- | --- | --- | --- | --- |
| FTZF1 3RACE1 | CGGTCCTGGAGTTGTGGGTCAATC | RACE | 73.4 |  |  |
| FTZF1 3RACE2 | CGGTATGGAAGAATTGTGCCCTGT | RACE | 70.5 |  |  |
| FTZF1 5RACE1 | GATGACACAGCTTCGATCCGCAAC | RACE | 72.7 |  |  |
| FTZF1 5RACE2 | AGGGCATCTTTTCCGTTGCGTTTT | RACE | 72.5 |  |  |
| MOD M13 Fwd | ATGACCATGATTACGCCAAG | Colony PCR | 62.3 | + |  |
| MOD M13 Rev | AACGACGGCCAGTGAATTGT | Colony PCR | 66.7 | + |  |
| αFTZF1 T7 Fwd | GAAATTAATACGACTCACTATAGGGTCACTCAACCCTACCAGTATTGA | RNAi | 76.1 | 408* |  |
| αFTZF1 T7 Rev | GAAATTAATACGACTCACTATAGGGTAAGTGTCCTCCAGCCTCTT | RNAi | 75.7 | 408* |  |
| βFTZF1 T7 Fwd | GAAATTAATACGACTCACTATAGGGTTGAGGACAACAACGACGAC | RNAi | 77.6 | 469* |  |
| βFTZF1 T7 Rev | GAAATTAATACGACTCACTATAGGGTTGGATGGACTACACTGCTCA | RNAi | 77.2 | 469* |  |
| Cod-specific T7 Fwd ¤ | GAAATTAATACGACTCACTATAGGGATAGGGCGAATTGGGTACCG | RNAi | 78.5 | 849 |  |
| Cod-specific T7 Rev ¤ | GAAATTAATACGACTCACTATAGGGAAAGGGAACAAAAGCTGGAGC | RNAi | 77.8 | 849 |  |
| αFTZF1 Fwd | CTCATTCAAAGTTCGGACGGA | RT-qPCR | 66.2 | 88 | 1.94 |
| αFTZF1 Rev | GAGGCGAGGAAGGGAAATTG | RT-qPCR | 66.9 | 88 | 1.94 |
| βFTZF1 Fwd | GCTGCGTCTTCTGCTATTACT | RT-qPCR | 60.3 | 112 | 1.95 |
| βFTZF1 Rev | GTCGTCGTTGTTGTCCTCAA | RT-qPCR | 63.9 | 112 | 1.95 |
| EF-1α Fwd | GGTCGACAGACGTACTGGTAAATCC | RT-qPCR | 67.4 | 229 | 1.89 |
| EF-1α Rev | TGCGGCCTTGGTGGTGGTTC | RT-qPCR | 74.7 | 229 | 1.89 |
| EMLSAG00000008331 Fwd | TCCTGATAAGGGTGGAGATCC | RT-qPCR | 64.0 | 66 | 1.91 |
| EMLSAG00000008331 Rev | TGAATGAGTCGGTTATACGCA | RT-qPCR | 63.0 | 66 | 1.91 |
| EMLSAG00000011833 Fwd | TGGGTTAGTTGGTGACGGAC | RT-qPCR | 64.8 | 115 | 1.89 |
| EMLSAG00000011833 Rev | CCCTATTGCCCACCGTTTAA | RT-qPCR | 65.5 | 115 | 1.89 |
| EMLSAG00000007107 Fwd | GGGTCATCGTCATCTGTAACC | RT-qPCR | 63.2 | 80 | 1.93 |
| EMLSAG00000007107 Rev | ACCAAAGGACTTTCTACTCCGA | RT-qPCR | 62.8 | 80 | 1.93 |
| EMLSAG00000010679 Fwd | AGCCCTAGAAATCGAGCCTG | RT-qPCR | 64.5 | 127 | 1.91 |
| EMLSAG00000010679 Rev | CGCATTCATTCCAGCTCCAA | RT-qPCR | 68.3 | 127 | 1.91 |
